# Highly Efficient Production of Heteroarene Phosphonates by Dichromatic Photoredox Catalysis

**DOI:** 10.1021/acsami.1c14497

**Published:** 2021-10-07

**Authors:** Jorge C. Herrera-Luna, David Díaz Díaz, M. Consuelo Jiménez, Raúl Pérez-Ruiz

**Affiliations:** † Departamento de Química, Universitat Politècnica de València (UPV), Camino de Vera S/N, 46022 Valencia, Spain; ‡ Departamento de Química Orgánica and Instituto de Bio-Orgánica Antonio González, Universidad de La Laguna, Avda. Astrofísico Francisco Sánchez 3, 38206 La Laguna, Spain; $ Institut für Organische Chemie, Universität Regensburg, 93053 Regensburg, Germany

**Keywords:** dichromatic photocatalysis, visible light, gel nanoreactor, heteroarene halides, phosphorylation

## Abstract

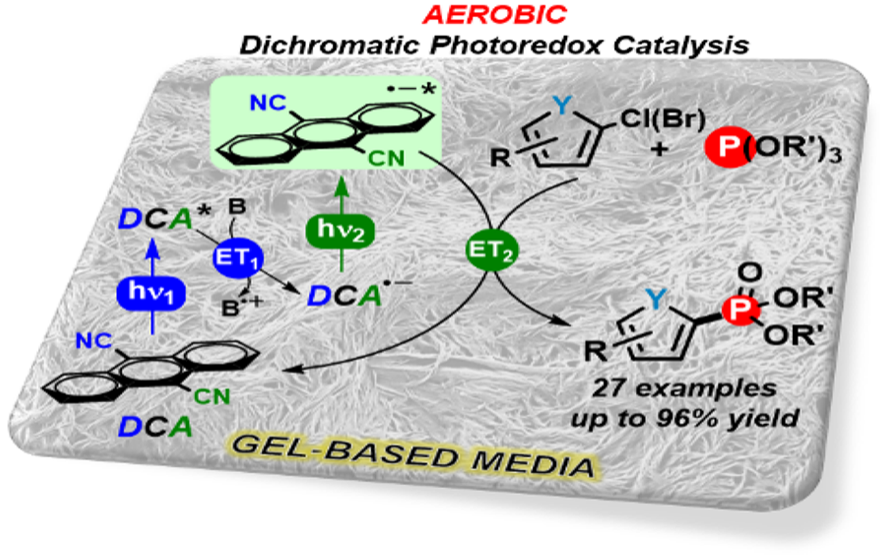

A new strategy to achieve efficient aerobic phosphorylation of five-membered heteraroenes with excellent yields using dichromatic photoredox catalysis in a gel-based nanoreactor is described here. The procedure involves visible aerobic irradiation (cold white LEDs) of a mixture containing the heteroarene halide, trisubstituted phospite, *N*,*N*-diisopropylethylamine (DIPEA) as sacrificial agent, and catalytic amounts of 9,10-dicyanoanthracene (**DCA**) in the presence of an adequate gelator, which permits a faster process than at the homogeneous phase. The methodology, which operates by a consecutive photoinduced electron transfer (ConPET) mechanism, has been successfully applied to the straightforward and clean synthesis of a number of different heteroarene (furan, thiophene, selenophene, pyrrole, oxazole, or thioxazole) phosphonates, extending to the late-stage phosphonylation of the anticoagulant rivaroxaban. Strategically, employment of cold white light is critical since it provides both selective wavelengths for exciting first **DCA** (blue region) and subsequently its corresponding radical anion **DCA**^•–^ (green region). The resultant strongly reducing excited agent **DCA**^•–^* is capable of even activate five-membered heteroarene halides (Br, Cl) with high reduction potentials (∼−2.7 V) to effect the C(sp^2^)–P bond formation. Spectroscopic and thermodynamic studies have supported the proposed reaction mechanism. Interestingly, the rate of product formation has been clearly enhanced in gel media because reactants can be presumably localized not only in the solvent pools but also through to the fibers of the viscoelastic gel network. This has been confirmed by field-emission scanning electron microscopy images where a marked densification of the network has been observed, modifying its fibrillary morphology. Finally, rheological measurements have shown the resistance of the gel network to the incorporation of the reactants and the formation of the desired products.

## Introduction

Aryl phosphonates and their derivatives are very important entities which exhibit a widespread applicability in diverse scientific fields such as life science,^1−3^ materials,^4−9^ or even acting as ligands in catalysis.^10−15^ In general, C(sp^2^)–P bonds are traditionally accessible by transition-metal-catalyzed coupling processes, and well-established methods using palladium,^16−19^ nickel,^20,21^ or copper^22,23^ catalysts have been reported. However, required functionalized reactants that consequently reduce the substrate scope, expensive metal complexes, or harsh conditions have limited the development of novel synthetic metal-catalyzed protocols. Visible-light photoredox catalysis has been postulated as an alternative and powerful strategy to forge new C(sp^2^)–P bonds under milder conditions.^24−33^ Generation of highly reactive aryl radical intermediates by direct single-electron transfer (SET) from the excited photocatalyst to the organic substrate has permitted the phosphorylation of aryl moieties with phosphorus-based nucleophiles to lead to the desired aryl phosphonates. For instance, six-membered (hetero)arene halides (Br, I) have been typically employed as precursors for the investigations of these cross-coupling reactions. Despite the fact that recent metal-catalyzed methods have been published,^34,35^ little attention has been paid to the fabrication of a broad scope of five-membered heteroarene phosphonates directly between furan, thiophene, selenophene, or pyrrole halides (Cl, Br) with phosphites by visible-light photoredox catalysis.

**Scheme 1:**
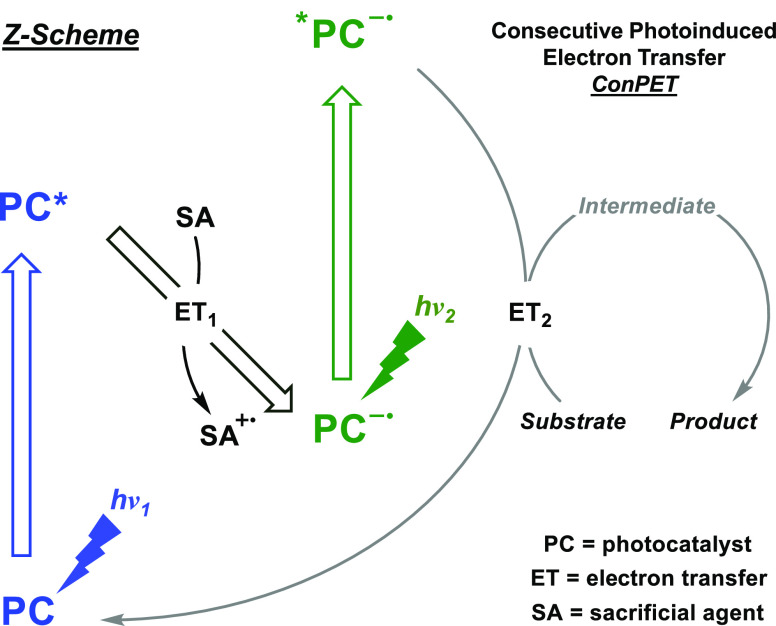
Z-Scheme Adaptation to Organic Transformations through the ConPET Concept

Biphotonic technology has emerged as a valuable tool for organic synthesis, and a recent revision of this research field can be found in literature.^36,37^ This accumulative two-photon approach allows us to tackle not only important bond activations but also SET processes under mild conditions and using lower energy visible light. Among others,^38,39^ the consecutive photoinduced electron transfer (ConPET) mechanism is of great interest and mirrors the Z-scheme in biological photosynthesis,^40^ and several organic dyes have been utilized as photocatalysts (Scheme 1).^41−46^ Briefly, upon selective excitation (*h*ν_1_) of the photocatalyst (PC), the resulting excited PC (PC*) is quenched by a sacrificial agent (SA) through single-electron transfer, giving rise to the radical ion pair (the PC anion radical, PC^•–^ and the SA cation radical, SA^•+^). Then the PC^•–^ can absorb another photon (*h*ν_2_) to yield its electronically excited anion radical (*PC^•–^) which is found to be sufficiently reactive for high energy demanding bond activation.

As a simple, low-weight molecule, soluble, photostable, and commercially available dye, the 9,10-dicyanoanthracene (**DCA**) has been recently found to be an ideal reducing photocatalyst for operating via a ConPET mechanism.^47^ Interestingly, the management of a simpler setup with low-power cold white LEDs is crucial since this energy source provides the simultaneous dichromatic light for exciting both the **DCA** ground state (near-UV-to-blue region) and the **DCA** radical anion (green region). From a mechanistic perspective, inert atmosphere conditions are mandatory to avoid quenching of **DCA** radical anion by oxygen. This differs from nature where visible light and oxygen are both abundant, playing a key role in aerobic photochemical reactions. Smartly, nature has normalized confined and compartmentalized environments for addressing efficiently photochemical transformations under air conditions. In this sense, research groups have been devoted to developing artificial photonanoreactors based on low-molecular-weight (LMW) molecules self-assembled by noncovalent interactions (e.g., hydrogen bonding, van de Waals, charge transfer, dipolar, π–π stacking)^48,49^ in order to access otherwise slow or forbidden pathways and achieve high selectivity under mild conditions. Supramolecular viscoelastic gels^50^ fulfill properly the requirements to act as a photonanoreactor-like system, and investigations of air-sensitive photochemical reactions have been successfully achieved in such microenvironments.^51,52^

**Scheme 2:**
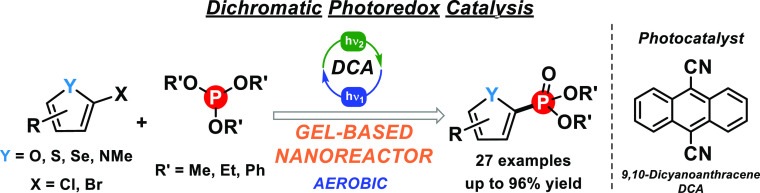
Formation of Five-Membered Heteroarene Phosphonates via Dichromatic Photoredox Catalysis in Gel Nanoreactor

With this background, we describe herein our endeavors toward the aerobic visible-light-mediated phosphorylation of five-membered heteroarenes in gel-based nanoreactor that, as far as we are aware, remains unexplored (Scheme 2).

Thus, we propose a catalytic strategy for the activation of unreactive heteroarene halides via dichromatic photoredox catalysis, leading to heteroarene radical intermediates which further react with the corresponding phosphorus-type nucleophiles. The employed conditions which include visible light, room temperature, aerobic atmosphere, and supramolecular gels as confined media would reflect a similar scenario than in nature, becoming an efficient and attractive strategy for organic synthesis. Moreover, utilization of **DCA** as a ConPET photocatalyst has satisfactorily extended the scope of starting materials to heteroarene bromides or heteroarene chlorides, whereas the LMW gel nanoreactor has provided an adequate environment for substantially accelerating the reactivity in comparison with solution phase.

## Results and Discussion

### Optimization

We first tested the irradiation between 5-chloro-2-thiophenecarbonitrile (**1a**) and triethyl phosphite (**2a**) as model reactants in anhydrous acetonitrile (ACN) with cold white light LEDs in the presence of *N*,*N*-diisopropylethylamine (DIPEA), as sacrificial electron donor agent, and catalytic amounts of **DCA** under aerobic atmosphere. The desired heteroarene phosphonate (**3a**) was obtained in poor yields together with a low conversion of the starting material (Table 1, entry 1). This fact was, as expected, due to poisoning by oxygen exposure that dropped drastically down the effectiveness of the photoreaction. In sharp contrast, employment of the aerated physical gel built from **G1** (*N*,*N*′-bis(octadecyl)-L-boc-glutamic diamide, see the molecular structure in Table 1) as confined medium at same reaction conditions resulted not only in excellent conversion and yield but also in higher selectivity toward the coupled product **3a** (Table 1, entry 2), confirming the efficient compartmentalization provoked by the gel network for this visible-light-induced synthetic procedure. In addition, the optimal concentration of **G1** was found to be at 10 mg/mL; for instance, changes in the **G1** amount did not give better outputs (Table 1, entries 3 and 4). This could be explained in terms of diffusion rates: (i) at above optimal **G1** concentration (15 mg/mL) the movement of the reactants could be restricted inside the solvent pools and (ii) the oxygen-diffused degree could be higher at below optimal **G1** concentration (5 mg/mL), affecting the photochemical mechanism. Likewise, the light scattering occasioned by these materials could also alter the overall reactivity, which could be minimized by adapting the solvent volume (see Table S1, entries 17 and 18); as a matter of fact, the lower the volume the higher the process efficiency.

**Table 1:**
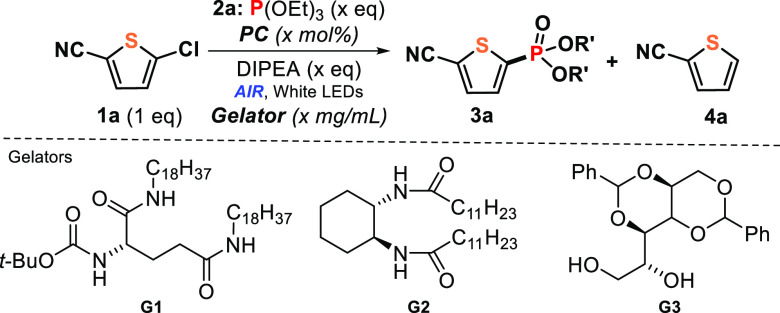
Optimization of the Reaction Conditions^a^

entry	**2a** (equiv)	DIPEA (equiv)	**PC**^b^ (mol %)	**G**^c^ (mg/mL)	conversion^d^ (%)	yield^d^ (%)	**3a/4a**^d^ ratio
1	5	1.2	10		14	10	71/29
**2**	**5**	**1.2**	**10**	**10**	**100**	**90**	90/10
3	5	1.2	10	15	85	74	87/13
4	5	1.2	10	5	87	75	86/14
5	20^e^	1.2	10	10	100	92	92/8
6	5	1.5	10	10	92	80	87/13
7	5	1	10	10	77	66	85/15
8	5	1.2	20	10	100	84	84/16
9	5	1.2	5	10	57	50	87/13
10	5	1.2	10^f^	10	40	32	80/20
11	5	1.2	10^g^	10	50	42	84/16
12	5	1.2	10^h^	10	55	48	87/13
13	5	1.2	10	10^i^	81	70	86/14
14	5	1.2	10	40^j^	73	56	76/24
15	5		10	10	0	0	0
16^k^	5	1.2	10	10	0	0	0

a **1a** (7.2 mg, 0.05 mmol) with G1 in 1 mL of anhydrous ACN; irradiation with cold white-light (410–700 nm) LEDs at 23 °C for 4 h unless otherwise indicated.

b DCA as photocatalyst unless otherwise indicated.

c G: gelator. H-bonding and van der Waals forces trigger the self-assembly process of gelators in organic solvent, affording tangled fibrillar nanostructures over a wide concentration range.^53−55^

d Conversions, yields, and ratios were calculated from quantitative GC analysis vs internal standard 1-dodecanenitrile.

e 2 h of irradiation.

f *N*,*N*-Bis(2,6-diisopropylphenyl)perylene-3,4,9,10-bis(dicarboximide) (PDI).

g Rhodamine 6G (Rh6G).

h Sulforhodamine B (SRhB).

i G2.

j G3.

k Using blue (420 nm) lamps or green (520 nm) LEDs.

In an attempt to turn the reaction into quantitative conditions, incremental changes in the amount of **2a** did show a slight improvement in the results (Table 1, entry 5), whereas variation at the DIPEA equivalents or different loading of the photocatalyst **DCA** gave lower yields of **3a** (Table 1, entries 6–9). Employment of other bases and solvents (see Table S1, entries 19–37) as well as other ConPET photocatalysts (Table 1, entries 10–12) showed less efficient reactions. Control experiments clearly demonstrated that the presence of DIPEA was essential for this photochemical protocol (Table 1, entry 15) and the negligible reactivity by photolysis with either blue (420 nm) lamps or green (520 nm) LEDs (Table 1, entry 16) reinforced the concept of a dichromatic excitation source for activating first the **DCA** and then its radical anion. The question then arose whether the versatility of this photoredox catalytic process was only associated to the self-assembled matrix of **G1** or if other gel-based materials containing different assembly patterns could also act as photonanoreactors. Thus, the model reaction was carried out in gels **G2** (*N*,*N*′-((1S,2S)-cyclohexane-1,2-diyl)didodecanamide; see the molecular structure in Table 1) and **G3** (1,3:2,4-dibenzylidene-d-sorbitol; see the molecular structure in Table 1) as confined media. The desired **3a** was successfully obtained in 70% and 56% yield, respectively (Table 1, entries 13 and 14), indicating that supramolecular viscoelastic gels proposed a suitable microenvironment for this type of photoinduced process. It is worth mentioning that gelators could be easily separated by filtration and reused in subsequent procedures without detriment to its gelation properties (see details in the Supporting Information).

Therefore, the optimal conditions implied acceptable reagent loadings (5 equiv of **2a**, 1.2 equiv of DIPEA and 10 mol % of **DCA**), visible-light irradiation using cold white LEDs (410–700 nm) as the energy source in **G1** confined medium for 4 h under air atmosphere.

### Scope

With the standardized conditions, we sought to examine the scope of this dichromatic photocatalyzed phoshorylation of thiophenes further (Table 2). In general, 10–20 mol % of **DCA** was necessary to observe a complete conversion of the starting materials. Although phosphite derivative **2a** was used as a representative coupling partner, other phosphites such as P(OMe)_3_ or P(OPh)_3_ were successfully coupled with **1a** (Table 2, entries **3b**–**c**) with special attention in product **3b** that was obtained almost in quantitative amount (97% yield). Commercially available thiophene chloride (or bromide) precursors containing acetyl, trifluoromethyl, or methyl group afforded the corresponding phosphonylated product from moderate-to-excellent yields (Table 2, entries **3d**–**3f**), signaling that the reaction proceeded with an acceptable functional group tolerance and was clearly favored to thiophene halides bearing electron-acceptor groups. Interestingly, a diphoshonate thiophene (Table 2, entry **3g**) was quantitatively obtained after a one-pot full conversion of the 2,5-dibromothiophene, and the reaction was found to be tolerant in terms of regioselectivity. For instance, phosphorylation of 2-thiophenecarbonitrile halides containing the halogen atom in position 3 or 4 provided the desired products in excellent yields (Table 2, entries **3h**,**i**); however, the observed lower production when the nitrile group was in position 3 (Table 2, entries **3j**,**k**) could be correlated with the high reduction potentials of the precursors (vide infra). Other regioisomers employing acetylthiophene-type halides were also obtained from moderate to excellent yields (Table 2, entries **3l**–**n**).

**Table 2:**
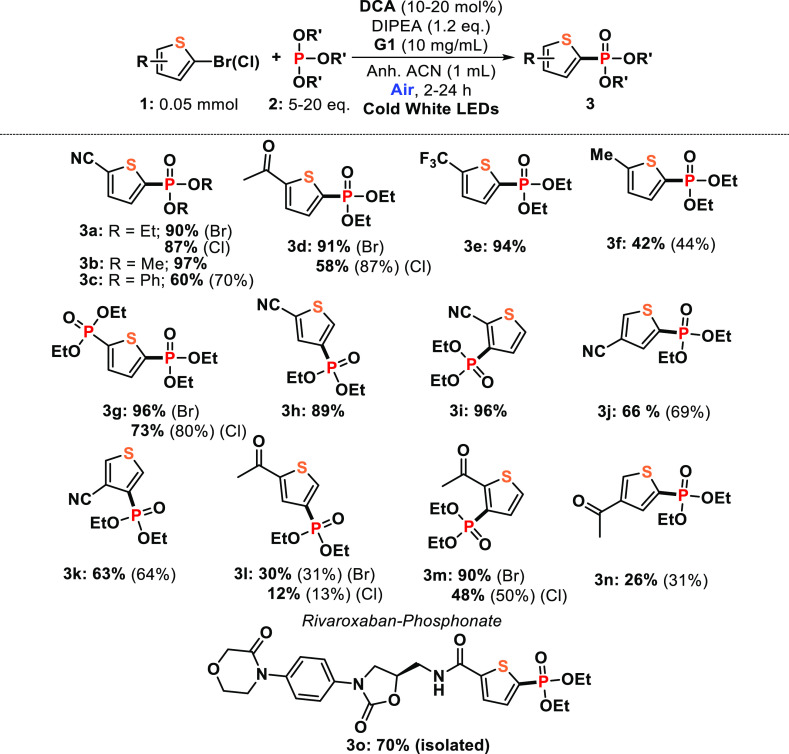
Substrate Scope of Thiophenes^a^

a For detailed information on the reaction conditions, see the Supporting Information. Full conversion of compound **1** in all cases unless otherwise indicated in brackets.

To demonstrate the synthetic potential of the developed dichromatic photocatalyzed transformation, this method was applied to the late-stage phosphonylation of rivaroxaban, an oral anticoagulant agent for the prevention and treatment of thromboembolic disorders.^56^ Thus, the subsequent phosphonylated product (Table 2, entry **3o**) was obtained in very good, isolated yields (70%). In addition, the model reaction was submitted at higher scales (from 0.05 to 1 mmol), producing a 66% isolated yield of **3a** (86% GC yield) and under outdoor sunlight generating **3a** after 8 h in an excellent 94% yield (see all details in the Supporting Information).

Next, we explored the feasibility of this procedure using other five-membered haloheterocycles such as furan, pyrrole, selenophene, oxazole, or thioxazole halides (Table 3, entries **10a**–**c**, **11a**, **12a**, **13a**, and **14a**). The results indicated that the coupling reaction of **2a** with the corresponding heteroarenes brilliantly succeeded, with almost quantitative yields in some cases (for instance, 96% for **10b**). Finally, we expanded the investigations to the phosphorylation of (hetero)aryl halides and **2a** to check the generality of our procedure where the corresponding products were obtained in high-to-excellent yields (Table 4, entries **15a**–**e**). Moreover, some of these findings were found to be comparable with that from example of rhodamine 6G as ConPET photocatalyst (i.e., 93%, 79% and 70% yields for **15a**, **15b** and **15e**, respectively),^46^ despite the lower addition of **2a** or DIPEA, aerobic gel media, or shortener irradiation times.

**Table 3:**
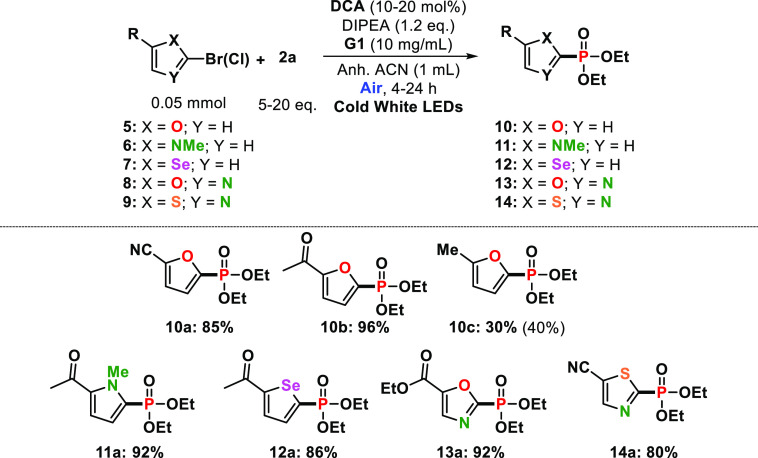
Phosphonylation of Other Five-Membered Heteroarene Halides^a^

a For detailed information on the reaction conditions, see the Supporting Information. Full conversion of starting materialsin all cases unless otherwise indicated in brackets.

**Table 4:**
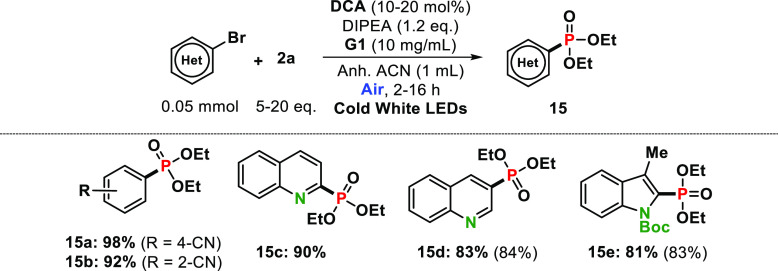
Coupling Reaction of (Hetero)aryl Halides with Triethyl Phosphite **2a**^a^

a For detailed information on the reaction conditions, see the Supporting Information. Full conversion of starting materials in all cases unless otherwise indicated in parentheses.

### Mechanism

**Figure 1:**
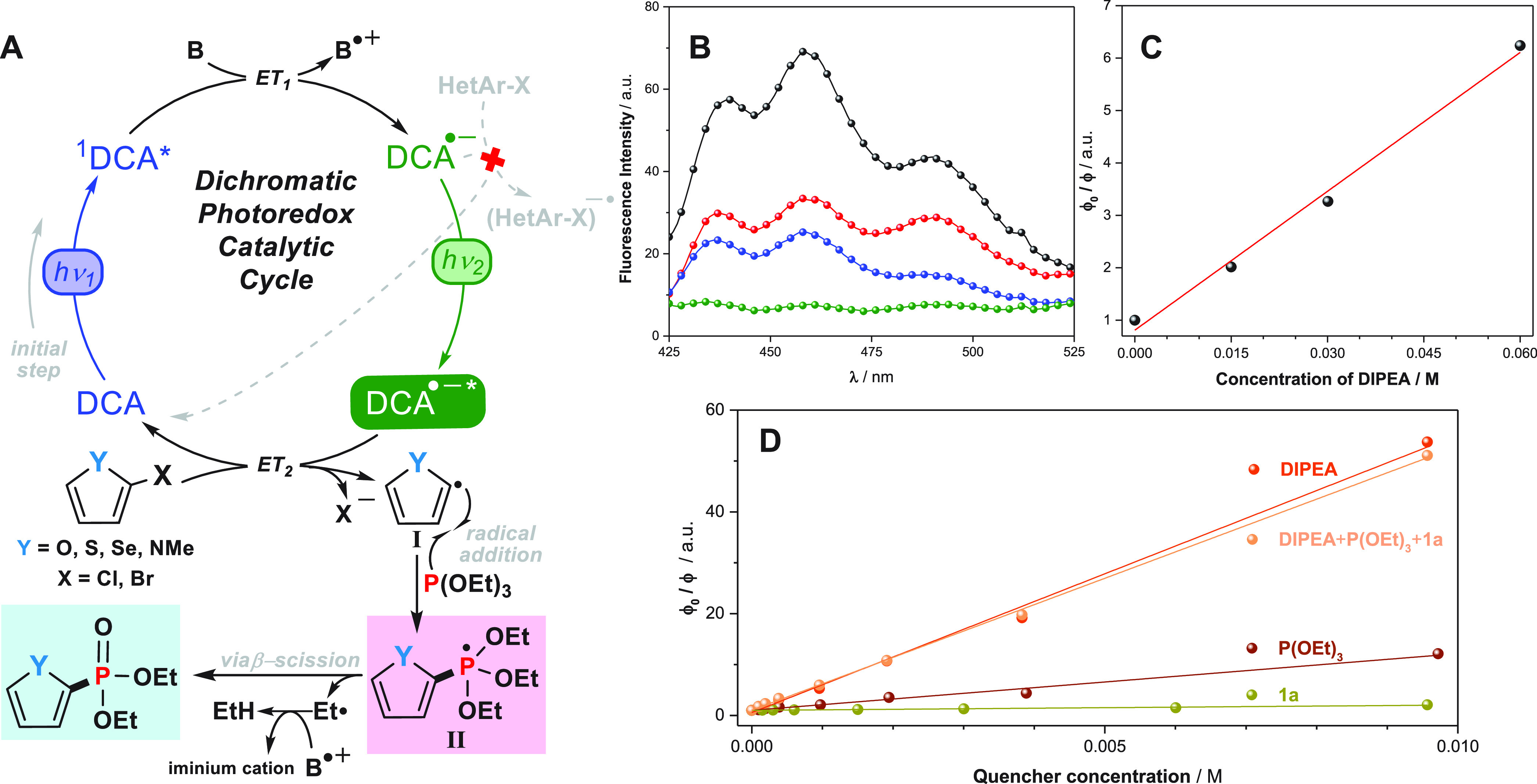
(A) Proposed reaction mechanism for the phosphonylation of five-membered heteroarenes dichromatic photoredox catalysis in gel nanoreactor. (B) Quenching of **DCA** fluorescence in the presence of increasing amounts of DIPEA in **G1** medium. [**DCA**] = 5 mM; [DIPEA] = 0 mM (black), 15 mM (red), 30 mM (blue) and 60 mM (green). (C) Stern–Volmer plot. (D) Stern–Volmer plots of **1a**, DIPEA, and P(OEt)_3_ quench of **DCA**.

Once product studies were established, we proposed the reaction mechanism for the dichromatic photoredox catalysis phosphonylation of five-membered heteroarene halides which is outlined in Figure 1A.

To check whether the **DCA** properties may or may not be similar in gel medium to those for the homogeneous phase, excitation and emission measurements were carried out employing an integrating sphere spectrofluorometer. As depicted in Figure S4, the results revealed no medium dependent. Hence, the singlet excited state of the ConPET photocatalyst **DCA** (^1^DCA*), generated upon blue-region light irradiation, oxidized DIPEA affording the corresponding radical ion pairs. This was confirmed by emission measurements in gel medium where fluorescence intensity of **DCA** gradually decreased in the presence of increasing amounts of DIPEA, indicating completely quenching at reaction conditions (Figure 1B). From the ^1^DCA* lifetime (15.9 ns in ACN)^57^ and the obtained Stern–Volmer constant (88.3 M^–1^, Figure 1C), the rate constant *k*_q_ (S_1_) for this pathway was found to be 5.5 × 10^9^ M^–1^ s^–1^. Additionally, quenching experiments showed that neither the heteroarene halide nor the phosphite-like derivative interacted individually with the ^1^DCA* and did not influence the deactivation by DIPEA (Figure 1D). Although the **DCA** radical anion (**DCA**^•–^) was efficiently generated, it possessed insufficient reducing power (−0.6 to −0.89 V vs SCE)^13^ to reductively activate the heteroarene halides. The reduction potentials of the heteroarene halides were measured by cyclic voltammetry in ACN under argon, ranging from −1.66 to −2.72 V (Table 5). Thus, these processes did not appear to be thermodynamically feasible where the free energy changes (Δ*G*_1_) associated with the electron transfer were estimated to be above zero (Table 5). This issue was fully supported by negligible formation of product in the photoreaction when using only blue lamps (Table 1, entry 16). In addition, the α-aminoalkyl radical could be generated from DIPEA radical cation, and it may react with the heteroarene halides through the halogen atom transfer mechanism;^58,59^ however, this mechanistic scenario appeared to be unlikely at these conditions due to lack of product formation.

**Table 5:**
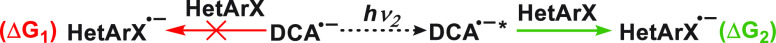

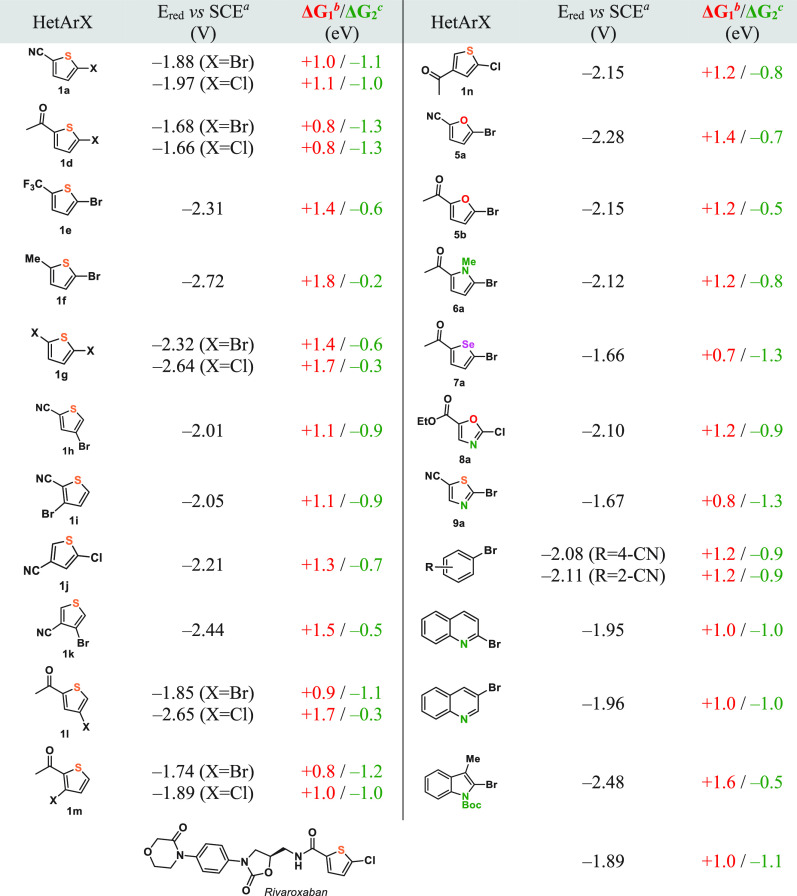
Thermodynamic Data of the SET Processes from DCA^•–^* and DCA^•–^ to HetArX

a *E*_1/2_^red^(vs SCE) in ACN was calculated from *E*_1/2_^red^ (vs SCE) = *E*_1/2_^red^ (vs ferrocene) + 0.38 V (see the Supporting Information for details).

b Free energy changes were estimated as follows:Δ*G*_1_ = *E*_1/2_^red^ (**DCA**) – *E*_1/2_^red^ (HetArX); Δ*G*_2_ = *E*_1/2_^red^ (**DCA**) – *E*_1/2_^red^ (HetArX) – *E*_ex,_ where *E*_ex_ is the photoelectron ejection energy estimated as 2.08 V.^70^

Then **DCA**^•–^ was excited by the green-light region, giving rise to its excited state, **DCA**^•–^*, a highly reactive species with an estimated reducing potential of −3.2 V (vs SCE)^60^ and a lifetime of 13.5 ns^61^ that allowed it to engage in reductive activation of heteroarene halides. In fact, the electron transfer from **DCA**^•–^* would be an exergonic process in all cases, and the trend of substrate reactivity was very accurately mirrored by these thermodynamic data (Table 5). The radical anion of the heteroarene halide then suffered rapid mesolytic scission of the C–X bond, leading to the corresponding halide anion (X^–^) and the heteroarene radical **I** which could be added to the phosphite-like derivative via radical addition to afford the phosphoranyl radical intermediate **II**.^62,63^ The involvement of intermediate **I** was confirmed by a trapping experiment employing diphenyl disulfide where efficient formation of 2-carbonitrile-5-phenylthiothiophene was detected (see the Supporting Information for details). Intermediate **II** could then undergo a β-scission fragmentation, giving rise to the desired heteroarene phosphonate and the ethyl radical which evolved to ethane by hydrogen-atom transfer.^25,26,64−69^

### Gel-Based Nanoreactor

**Figure 2:**
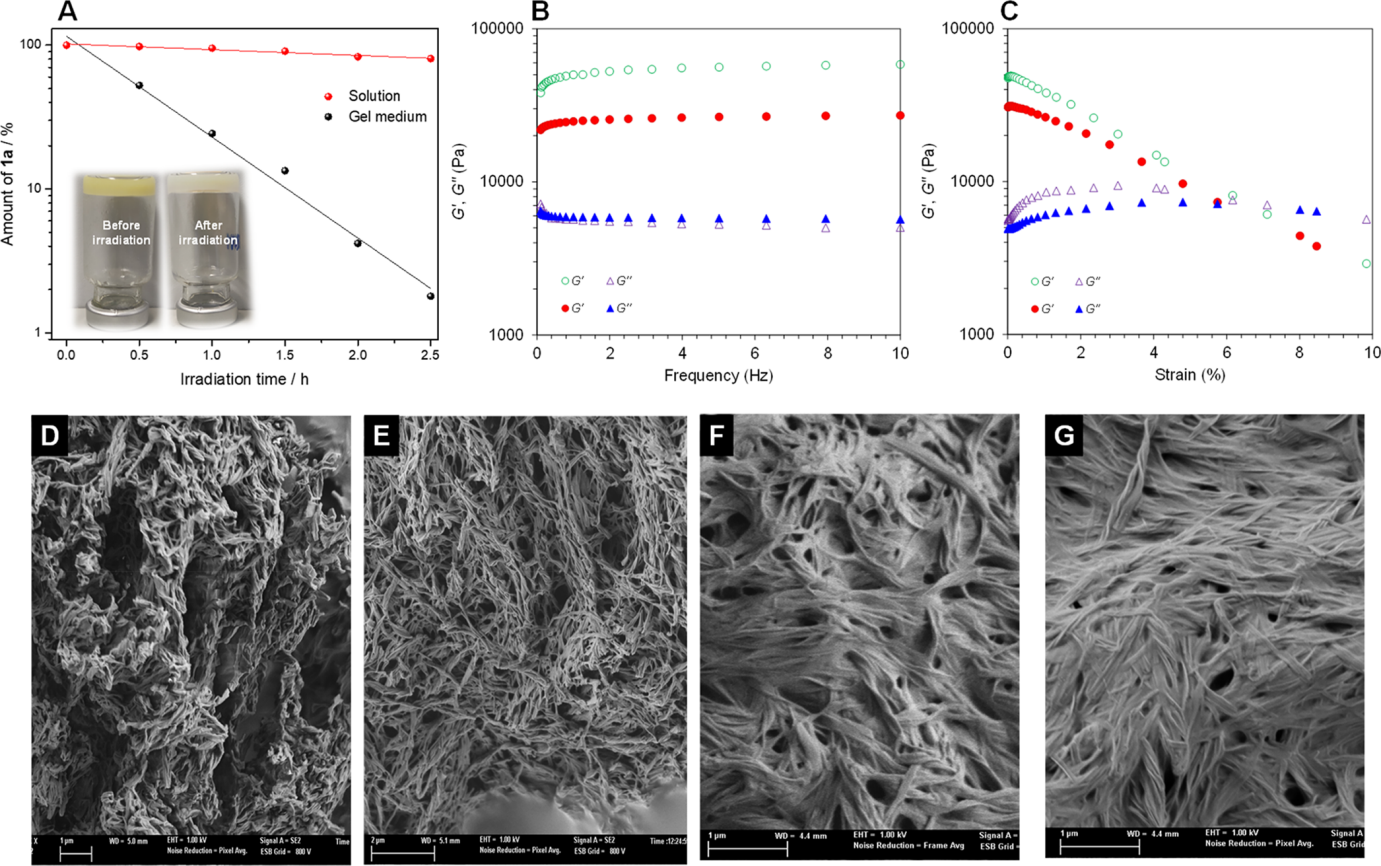
(A) Conversion of **1a** vs irradiation time at optimal reaction conditions in solution or gel medium. Inset: photograph of the loaded **G1** before/after irradiation. (B) Dynamic frequency sweep (DFS) plots: variation of *G′* and *G′′* with frequency (from 0.1 to 10 Hz at 0.1% strain), loaded **G1** before (unfilled) and after (filled) irradiation. (C) Dynamic strain sweep (DSS) plots: variation of *G′* and *G′′* with strain (from 0.01 to 100%), loaded **G1** before (unfilled) and after (filled) irradiation. Representative field-emission scanning electron microscopy (FESEM) images of **G1** prepared by freeze-drying the gel: (D) unloaded **G1** before irradiation; (E) unloaded **G1** after irradiation; (F) loaded **G1** before irradiation; (G) loaded **G1** after irradiation.

As stated above, the potential advantage of carrying out the dichromatic photocatalyzed phosphorylation of heteroarenes in gels as confined media has been well-established by steady-state irradiations. To further support the role of viscoelastic gel networks as successful nanoreactors, combination of several experimental investigations which comprises kinetic studies, field-scanning electron microscopy (FESEM), and oscillatory rheological measurements were performed. First, the kinetic of **1a** conversion was investigated under both solution and gel media (Figure 2A). The result clearly revealed that **1a** converted remarkably faster in gel medium than in inert solution for the same irradiation time;^71^ likewise, a similar trend was observed with the formation of product **3a** (see details in the Supporting Information). Along this vein, the model reaction was submitted to 30 min irradiation under frozen (193 K) aerated anhydrous ACN solution, affording negligible production of **3a** that would indicate a molecular diffusion restriction. On the contrary, product **3a** was successfully obtained in 41% yield at identical conditions in the presence of **G1** (see the Supporting Information for details). This might be hypothesized as the reactants may be not only localized in the solvent pools between the fibers but also widespread through the fibers, allowing the photochemical process in a confined but dynamic space. In addition, the aspect of the material to the naked eyes showed that the viscoelastic properties of the gels after irradiation did not vary in a critical manner; in other words, there was no gravitational flow (inset of Figure 2A).

To endorse these findings, field-emission scanning electron microscopy (FESEM) images of the unloaded **G1** and **G1** loaded with the photocatalyst, and substrates were recorded (Figure 2D–G). A marked densification of the network was clearly observed at the loaded gel, which could be attributed to the inclusion of the reactants within the supramolecular gel and therefore apparently triggering an impact on the fibrillar morphology of the gel network. Interestingly, the gel phase did not appear to suffer dramatic morphological changes after the irradiation period, as can be seen from the FESEM images, indicating that the gel matrix only played the role of nanoreactor and did not interfere in the dichromatic photocatalyzed reaction.

When using a gel system as compartmented medium is important to ensure that, at least, its viscoelastic nature remains through the entire experiment. This was demonstrated by rheological experiments before and after irradiation (Figure 2B,C). These measurements indicated that the storage modulus *G′* was always 1 order of magnitude higher than the loss modulus *G″* during both dynamic frequency and strain sweeps (DFS and DSS, respectively). Analyzing in detail the plots, DFS (Figure 2B) data suggested that the gel strength decreased during the experiment as shown by the corresponding dissipation factor (i.e., tan δ (*G″*/*G′*) = 0.1158 (before irradiation) vs 0.2402 (after irradiation)). This was not totally unexpected as new species were formed during the irradiation, which could also destabilize the supramolecular network. Moreover, DSS (Figure 2C) measurements showed that the gel phase turned into liquid at 9.8% strain before irradiation, while this transition took place at 8% strain after the experiment, which was also in agreement with the DFS profiles. Despite the observed weakening, the gel network resisted during the entire experiment providing the unique environment to facilitate the discussed chemical transformations.

## Conclusions

In conclusion, supramolecular gels made in acetonitrile can be used as reaction media to carry out aerobic visible-light-mediated phosphorylation of five-membered heteroarenes affording the corresponding aryl phosphonates. Optimized conditions include the use of white light LEDs (410–700 nm), DIPEA as sacrificial electron donor agent, catalytic amounts of **DCA** (10 mol %), and *N*,*N*′-bis(octadecyl)-l-Boc-glutamic diamide as gelator whose noncovalent nature of the supramolecular network enables its easy separation and reutilization. Interestingly, the gel media significantly enhanced the reaction rate in some cases compared to those in homogeneous solution. In terms of scope, commercially available five-membered heteroarene chloride (or bromide) precursors containing an acetyl, ester, cyano, trifluoromethyl, or methyl group afforded the corresponding phosphonylated products in moderate to excellent yields within 4 h. The synthetic potential of this dichromatic photocatalyzed transformation was demonstrated by its application in the late-stage phosphonylation of the anticoagulant rivaroxaban. Spectroscopic and thermodynamic studies supported the involvement of the strongly reducing excited radical anion **DCA**^•–^* in the proposed reaction mechanism. This species was found to be capable to activate heteroarene halides with high reduction potentials. Finally, FESEM and rheological measurements suggested that the gel network resisted the incorporation of the reactants and the formation of the desired products. Although certain detriment in the mechanical properties of the gels was observed during the reactions, it apparently did not harm their efficiency as confined reaction media. We believe this methodology could become a general approach to facilitate photoredox catalysis under aerobic conditions.

## Experimental Section

### Materials and Methods

All reagents (≥97% purity) and solvents (≥99% purity) were purchased from commercial suppliers (Merck, TCI, Apollo Scientific, Fluorochem, Scharlab) and used as received unless otherwise indicated. Reactions were carried out in a Metria-Crimp Headspace clear vial flat bottom (10 mL, Ø 20 mm) sealed with a Metria-aluminum crimp cap with molded septum butyl/natural PTFE (Ø 20 mm). Irradiation was performed with a cool white LED (LED Cree MK-R, cold-white, 11.6 V, 700 mA, P = 8.5 W). TLC was performed on commercial SiO_2_-coated aluminum and plastic sheets (DC60 F254, Merck). Visualization was done by UV light (254 nm). Products were isolated materials after column flash chromatography or TLC on silica gel (Merck, mesh 35–70, 60 Å pore size), and their corresponding yields were determined by quantitative GC-FID measurements on an Agilent 8860 GC-System with N2 as carrier gas. Dodecanenitrile was used as an internal standard in the GC-FID quantitative measurements; yield products were estimated as [conversion × selectivity]/mass balance. Determination of purity and structure confirmation of the literature known products was performed by ^1^H, ^13^C, ^19^F, and ^31^P NMR and high-resolution mass spectrometry (HRMS) in the case of unknown products. NMR spectral data were collected on a Bruker Advance 400 (400 MHz for ^1^H, 101 MHz for ^13^C, 376 MHz for ^19^F, and 162 MHz for ^31^P) spectrometer at 20 °C. Chemical shifts are reported in δ/ppm, and coupling constants *J* are given in hertz. Solvent residual peaks were used as internal standard for all NMR measurements. The quantification of ^1^H cores was obtained from integrations of appropriate resonance signals. Abbreviations used in NMR spectra: s, singlet; d, doublet; t, triplet; q, quartet; m, multiplet; dd, doublet of doublet; ddd, doublet of doublet of doublet; td, triplet of doublet; and dq, doublet of quartet. HRMS was performed in the mass facility of SCSIE University of Valencia.

### General Procedure for the Phosphorylation of Heteroarenes Halides

A vial (10 mL) was charged with 9,10-anthracenedicarbonitrile (1.2 mg, 5 μmol, 10 mol % and the correspondent gelator (G1, 10 mg/mL). Anhydrous acetonitrile (1.0 mL) was poured into the vial, and 5-chloro-2-thiophenecarbonitrile (5.4 μL, 50 μmol, 1.0 equiv) and triethyl phosphite (45 μL, 250 μmol, 5.0 equiv) were added. Then DIPEA (10.5 μL, 60 μmol, 1.2 equiv) and dodecanenitrile (12.0 μL, 50 μmol, 1.0 equiv) were added with a 25 μL Hamilton syringe. The vial was sealed with a septum. It was heated to 150 °C with a heatgun for 1.5 min with manual stirring until a clear solution was obtained. The vial was cooled to room temperature until gel formation was observed. The reaction was irradiated with an external LED through the plain bottom side of the vial at 23 °C during the corresponding time. Then brine (2 mL) was added, and the aqueous phase was extracted with ethyl acetate (1 mL). The reaction was monitored by GC-FID analysis. The organic phase was dried over anhydrous sodium sulfate, filtered from the drying agent, and concentrated in vacuo. The crude was purified via a TLC plastic sheet or flash column chromatography using a hexane/ethyl acetate mixture as the mobile phase.

### Spectroscopic Measurements

The absorption spectra were recorded on a JASCO V-630 spectrophotometer.

The fluorescence spectra were recorded on an FS5 Edinburgh instrument spectrofluorometer with a SC-05 standard cuvette holder module and an SC-30 integrating sphere module.

### Cyclic voltammetry

The redox potentials were measured by cyclic voltammetry with an Solartron 1284 potentiostat. All measurements were made in deaerated acetonitrile containing tetrabutylammonium tetrafluoroborate (0.1 M) as supporting electrolyte, a glassy carbon as working electrode, a platinum wire as counter electrode, a silver wire as pseudoreference, and ferrocene (0.01 M) as internal standard. The scan rate was 100 mV·s^–1^. Potentials are reported with respect to the saturated calomel electrode (SCE) as reference.

### Oscillatory Rheology

Oscillatory rheology was performed with an AR 2000 Advanced rheometer (TA Instruments) equipped with a Julabo C cooling system. A 1000 μm gap setting, and a torque setting of 40000 dyn cm^–2^ at 25 °C were used for the measurements in a plain-plate (40 mm, stainless steel).

The following experiments were performed using 2 mL total gel volume: (a) dynamic strain sweep (DSS), variation of *G*′ and *G*′’ with strain (from 0.01 to 100%); (b) dynamic frequency sweep (DFS), variation of *G*′ and *G*′’ with frequency (from 0.1 to 10 Hz ar 0.1% strain).

### Field-Emission Scanning Electron Microscopy

The equipment in operation in the UPV Microscopy Service is the ZEISS ULTRA 55 model, incorporating the following detectors:

- A secondary electron detector (SE2), which provides an SEM topography image of the sample surface with a large depth of field
- A secondary electron in-lens detector located inside the electron column, which works with low energy secondary electrons and provides images with a higher resolution
- A backscattered electron detector (AsB), which is sensitive to the variation of atomic number in the elements present in the sample; therefore, it is used to observe changes in the chemical composition of the specimen
- A backscattered electron In-lens detector (EsB), independent of the secondary In-lens detector, which provides a pure backscattered signal with no secondary electron contamination and very low acceleration potential
- An X-ray dispersive energy detector (EDS, Oxford Instruments), which receives X-rays from each surface point the electron beam passes over

## Supporting Information Available

Materials and methods, general procedures, table which includes optimizing conditions, emission measurements, GC chromatograms, characterization of products and spectroscopic data of all compounds (PDF)
