# Supplementary material for: Highly Efficient Production of Heteroarene Phosphonates by Dichromatic Photoredox Catalysis
Source: ACS Appl Mater Interfaces. 2021 Oct 7;13(41):48784–94. doi: 10.1021/acsami.1c14497 (PMC8630706; doi:10.1021/acsami.1c14497)
Supplement: Supplementary file 1 — am1c14497_si_001.pdf [file am1c14497_si_001.pdf]

Supplementary information

Highly Efficient Production of Heteroarene Phosphonates by Dichromatic Photoredox Catalysis

Jorge C. Herrera-Luna,[a] David Díaz Díaz,[b,c] M. Consuelo Jiménez,*[a] and Raúl Pérez-Ruiz*[a]

[a] Departamento de Química, Universitat Politècnica de València (UPV), Camino de Vera S/N, 46022, Valencia, Spain.

[b] Departamento de Química Orgánica and instituto de Bio-Orgánica Antonio González, Universidad de La Laguna, Avda. Astrofísico Francisco Sánchez 3, 38206, La Laguna, Spain.

[c] Institut für Organische Chemie, Universität Regensburg, Universitätsstr. 31, 93053, Regensburg, Germany.

mcjimene@qim.upv.es; raupreru@qim.upv.es

TABLE OF CONTENTS

➢ **Materials and methods** **S2**

➢ **General procedure** **S3**

➢ **Synthesis of LMW Gelators** **S4**

➢ **Searching the optimal conditions** **S5**

➢ **Cyclic Voltammetry** **S7**

➢ **Kinetic studies** **S11**

➢ **Frozen model reaction procedure** **S12**

➢ **UV-vis absorption spectra** **S13**

➢ **Trapping reaction** **S16**

➢ **Sunlight irradiation and 1 mmol scale** **S17**

➢ **Oscillatory rheology** **S19**

➢ **Field-emission scanning electron microscopy (FESEM)** **S20**

➢ **Characterization of compounds** **S24**

➢ **NMR spectra** **S35**

Materials and methods

All reagents (≥97% purity) and solvents (≥99% purity) were purchased from commercial suppliers (Merck, TCI, Apollo Scientific, Fluorochem, Scharlab) and used as received unless otherwise indicated. Reactions were carried out in Metria®-Crimp Headspace clear vial flat bottom (10 mL, Ø 20 mm) sealed with Metria®-aluminium crimp cap with moulded septum butyl/natural PTFE (Ø 20 mm). Irradiation was performed with a cool white LED (LED Cree MK-R, cold-white, 11.6 V, 700 mA, P = 8.5 W). TLC was performed on commercial SiO_2-coated aluminium and plastic sheets (DC60 F254, Merck). Visualization was done by UV-light (254nm). Product were isolated materials after column flash chromatography or TLC on silica gel (Merck, mesh 35-70, 60 Å pore size) and their corresponding yields were determined by quantitative GC-FID measurements on an Agilent 8860 GC-System with N_2 as carrier gas. Dodecanenitrile was used as an internal standard in the GC-FID quantitative measurements; yield products were estimated as: [conversion × selectivity]/mass balance. Determination of purity and structure confirmation of the literature known products was performed by ^{1}H NMR, ^{13}C NMR, ^{19}F, ^{31}P and high-resolution mass spectrometry (HRMS) in case of unknown products. NMR spectral data were collected on a Bruker Advance 400 (400 MHz for ^{1}H, 101 MHz for ^{13}C, 376 MHz for ^{19}F and 162 MHz for ^{31}P) spectrometer at 20 °C. Chemical shifts are reported in δ/ppm, coupling constants J are given in Hertz. Solvent residual peaks were used as internal standard for all NMR measurements. The quantification of ^{1}H cores was obtained from integrations of appropriate resonance signals. Abbreviations used in NMR spectra: s – singlet, d – doublet, t – triplet, q – quartet, m – multiplet, dd – doublet of doublet, ddd – doublet of doublet of doublet, td – triplet of doublet and dq – doublet of quartet. HRMS was carried out was performed in the mass facility of SCSIE University of Valencia. Absorption spectra were recorded on a JASCO V-630 spectrophotometer. The fluorescence spectra were recorded on an FS5 Edinburgh instrument spectrofluorometer with a SC-05 standard cuvette holder module and an SC-30 integrating sphere module.

General procedure

A vial (10 mL) was charged with 9,10-anthracenedicarbonitrile (1.2 mg, 5 μmol, 10 mol% and the correspondent gelator (G1, 10 mg/mL). Anhydrous acetonitrile (1.0 mL) was poured and 5-chloro-2-thiophenecarbonitrile (5.4 μL, 50 μmol, 1.0 equiv.) and triethylphosphite (45 μL, 250 μmol, 5.0 equiv.) were added. Then, DIPEA (10.5 μL, 60 μmol, 1.2 equiv.) and dodecanenitrile (12.0 μL, 50 μmol, 1.0 equiv.) were added with 25 μL Hamilton syringe. The vial was sealed with a septum. It was heated to 150 °C with heatgun for 1.5 minutes with manual stirring until complete clear solution. The vial cooled to room temperature until gel formation was observed. The reaction was irradiated with an external LED through the plain bottom side of the vial at 23 °C during the corresponding time. Then, brine (2 mL) was added, and the aqueous phase was extracted with ethyl acetate (1 mL). The reaction was monitored by GC-FID analysis. The organic phase was dried over anhydrous sodium sulfate, filtered from the drying agent, and concentrated in vacuo. The crude was purified via TLC plastic sheet or flash column chromatography using a hexane/ethyl acetate mixture as the mobile phase. Note: The gelator can be easily separated by filtration and reused in subsequent experiments without any detriment of its gelation properties.

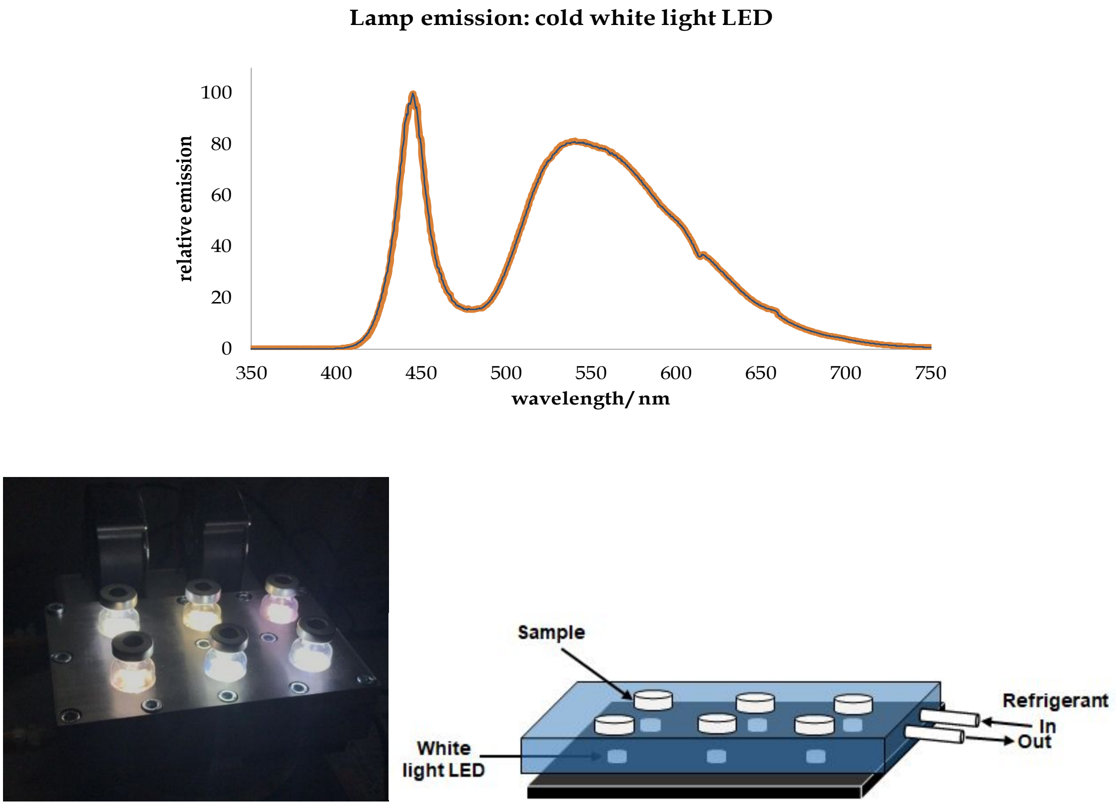

Chart S1. Lamp emission spectrum of the white light LEDs and irradiation setup.

Synthesis of Low Molecular Weight Gelators (LMW Gelators)

*N,N'-Bis(octadecyl)-L-Boc-glutamic Diamide (**G1**).*

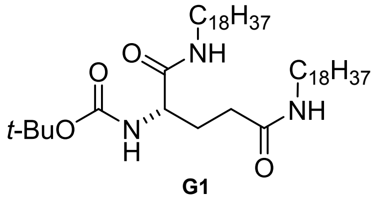

G1

Boc-glutamic acid (0.01 mol, 1.0 equiv) and octadecylamine (0.02 mol, 2.0 equiv) in dichloromethane (200 mL) were mixed. Then, 1-ethyl-3-(3-(dimethylamino)propyl)carbodiimide hydrochloride (EDC·HCl) (0.022 mol, 2.2 equiv) was added to the mixture and stirred at RT for 72 h. The obtained white solid was isolated by filtration and washed three times with dichloromethane. The crude product was dissolved in THF and precipitated by water. A fine white solid was obtained (80%). Following the procedure reported previously *Soft Matter* **2007**, *3*, 1312−1317.

^{1}H NMR (400 MHz $CDCl_3$) δ 6.69 (br, 1H), 6.32 (br, 1H), 5.77 (br, 1H), 4.08 (br, 1H), 3.26−3.22 (m, 4H), 2.41−2.27 (m, 2H), 2.06−1.93 (m, 2H), 1.58−1.46 (m, 4H), 1.43 (s, 9H), 1.25 (s, 60H), 0.87 (t, 6H) ppm.

(S,S)-Dodecyl-3-[2(3-dodecyl-ureido)cyclohexyl]urea (***G2***).

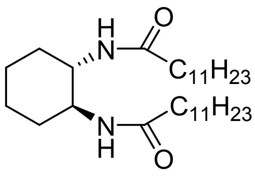

G2

A solution of dodecylisocyanate (15 mmol, 2.0 equiv) in toluene (20 mL) was slowly added to a solution of (*S,S*)-1,2-cyclohexyldiamine (7 mmol, 1.0 equiv) in toluene (100 mL). The reaction mixture was stirred for 16 h at RT and 2 h at 100 °C. After cooling to RT, the gel-like reaction mixture was filtered to give a white waxy solid. The waxy solid was further stirred for 16 h with dichloromethane (50 mL) and collected by filtration. This procedure was repeated with diethyl ether. After drying, a white solid was obtained (70%). Following the procedure reported previously *Chem. - Eur. J.* **1999**, *5*, 937−950.

^{1}H NMR (400 MHz, $CDCl_3$) δ 5.18 (br, 2H), 4.67 (br, 2H), 3.42 (m, 2H), 3.08 (m, 4H), 2.03 (d, 2H), 1.72 (m, 6H), 1.45 (m, 4H), 1.25 (s, 36H), 0.87 (t, 6H) ppm.

1,3,2,4-Dibenzylidene-D-sorbitol (***G3***).

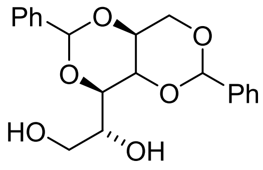

G3

Commercially available.

Searching the optimal conditions

Table S1. Optimizing the reaction conditions[a]

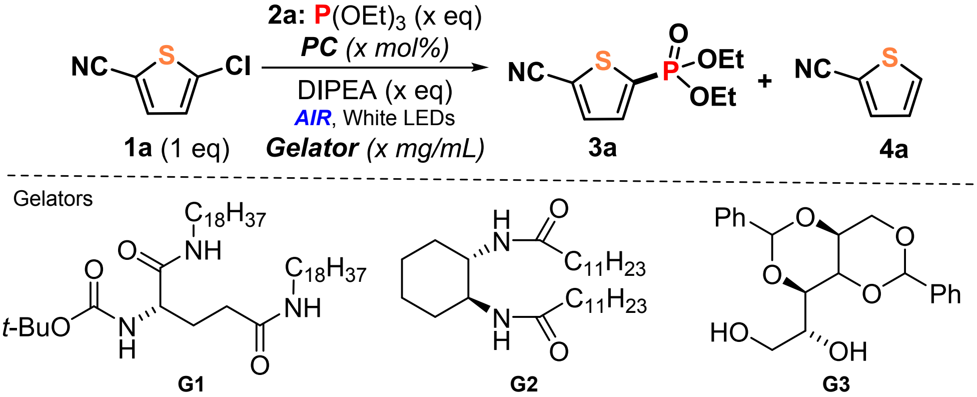

Entry	2a (eq)	DIPEA (eq)	PC[b] (mol%)	G[c] (mg/mL)	Conversion[d] (%)	Yield[d] (%)	3a/4a[d] ratio
1	5	1.2	10	-	14	10	71/29
**2**	**5**	**1.2**	**10**	**10**	**100**	**90**	**90/10**
3	5	1.2	10	15	85	74	87/13
4	5	1.2	10	5	87	75	86/14
5	20[e]	1.2	10	10	100	92	92/8
6	5	1.5	10	10	92	80	87/13
7	5	1	10	10	77	66	85/15
8	5	1.2	20	10	100	84	84/16
9	5	1.2	5	10	57	50	87/13
10	5	1.2	10[e]	10	40	32	80/20
11	5	1.2	10[f]	10	50	42	84/16
12	5	1.2	10[g]	10	55	48	87/13
13	5	1.2	10	10[h]	81	70	86/14
14	5	1.2	10	40[i]	73	56	76/24
15	5	-	10	10	0	0	0
16[j]	5	1.2	10	10	0	0	0
17	5	1.2	10	22 in 2mL	55	51	93/7
18	5	1.2	10	45 in 4mL	24	22	92/8
19[k]	5	1.2	10	10	73	23	32/68
20[l]	5	1.2	10	10	63	17	27/73
21[m]	5	1.2	10	10	10	6	73/27
22[n]	5	1.2	10	10	46	12	27/73
23[o]	5	1.2	10	10	18	6	35/65
24[p]	5	1.2	10	10	12	0	0
25[q]	5	1.2	10	10	34	20	61/39
26[r]	5	1.2	10	10	94	80	86/14
27[s]	5	1.2	10	10	11	7	64/36
28[t]	5	1.2	10	10	8	3	42/58
29[u]	5	1.2	10	10	57	15	27/63

30[v]	5	1.2	10	10	90	79	88/12
31[w]	5	1.2	10	10	67	60	89/11
32	5	Et_3N 1.2	10	10	63	57	91/9
33	5	DIPA 1.2	10	10	35	31	89/11
34	5	DBU 1.2	10	10	93	74	80/20
35	5	DABCO 1.2	10	10	7	5	73/27
36	5	K_2CO_3 1.2	10	10	26	20	79/21
37	5	Et_3N 1.2	10	10	63	57	91/9
38[x]	5	1.2	10	-	37	33	90/10

[a] **1a** (7.2 mg, 0.05 mmol) with **G1** in 1 mL of anhydrous ACN; irradiation with cold white-light (410-700 nm) LEDs at 23 ºC for 4 hours unless otherwise indicated. [b] **DCA** as photocatalyst unless otherwise indicated. [c] **G**: gelator. H-bonding and van der Waals forces trigger the self-assembly process of gelators in organic solvent, affording tangled fibrillar nanostructures over a wide concentration range.[*] [d] Conversions, yields and ratios were calculated from quantitative GC analysis *vs.* internal 1-dodecanonitrile. [e]2 hours of irradiation. [f] *N*,*N*-bis(2,6-diisopropylphenyl)perylene-3,4,9,10-bis(dicarboximide) (PDI). [f] Rhodamine 6G (Rh6G). [g] Sulforhodamine B (SRhB). [h] **G2**. [i] **G3**. [j] Using Blue (420 nm) lamps or Green (520 nm) LEDs. [k]DMF, [l]DMA, [m]AcOEt, [n]EtOH, [o]Dioxane, [p]Toluene, [k]DCM, [r]Acetone, [s]Ether, [t]Hexane, [u]DMSO, [v]ACN, [w]ACN/H_2O (9/1, v/v), [x]under anaerobic conditions.

[*] (a) Li, Y.; Wang, T.; Liu, M. Gelating-induced supramolecular chirality of achiral porphyrins: chiroptical switch between achiral molecules and chiral assemblies. Soft Matter 2007, 3, 1312−1317. (b) Hanabusa, K.; Yamada, M.; Kimura, M.; Shirai, H. Prominent Gelation and Chiral Aggregation of Alkylamides Derived from trans- 1,2-Diaminocyclohexane. Angew. Chem., Int. Ed. Engl. 1996, 35, 1949−1951. (c) Watase, M.; Nakatani, Y.; Itagaki, H. On the Origin of the Formation and Stability of Physical Gels of Di-*O*-benzylidene-ᴅ-sorbitol. J. Phys. Chem. B 1999, 103, 13, 2366–2373

Cyclic Voltammetry

The redox potentials were measured by cyclic voltammetry with an Solartron 1284 potentiostat. All measurements were made in deaerated acetonitrile containing tetrabutylammonium tetrafluoroborate (0.1 M) as supporting electrolyte, a glassy carbon as working electrode, a platinum wire as counter electrode, a silver wire as pseudo reference and ferrocene (0.01 M) as internal standard. The scan rate was 100 mV·s^{-1}. Potentials are reported with respect to the saturated calomel electrode (SCE) as reference.

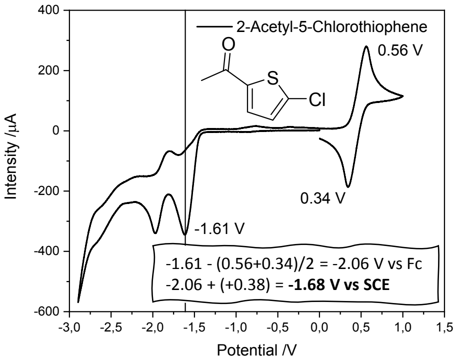

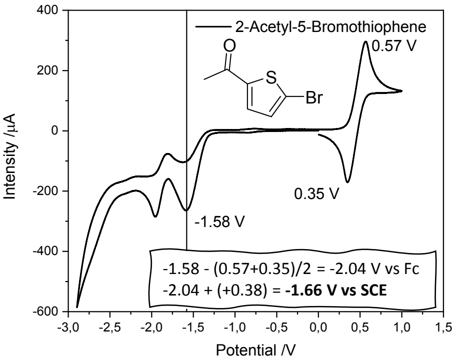

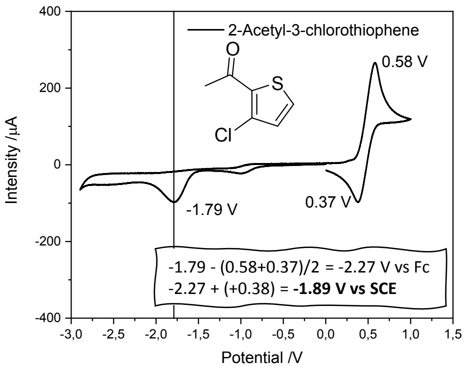

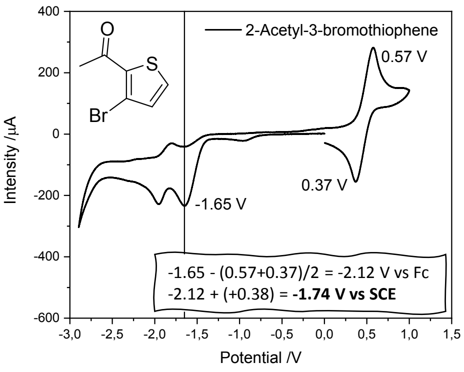

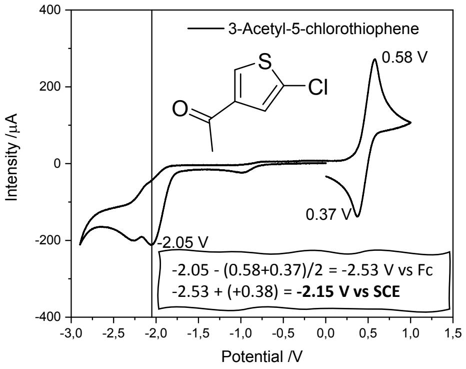

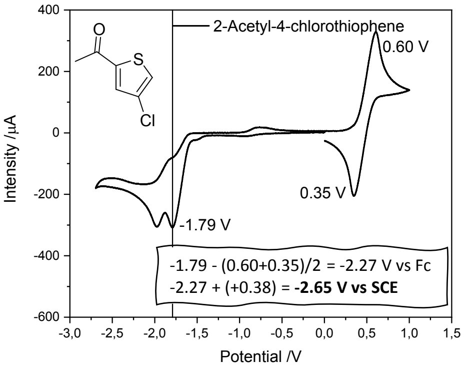

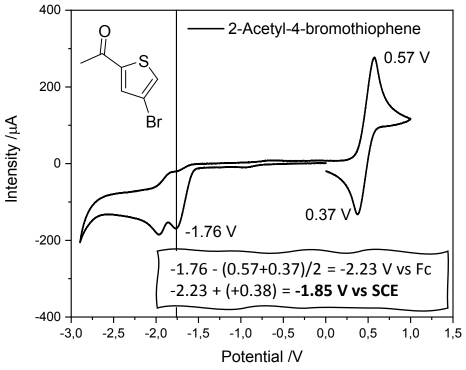
2-Acetyl-4-bromothiophene
0.57 V
0.37 V
-1.76 V
-1.76 - (0.57+0.37)/2 = -2.23 V vs Fc
-2.23 + (+0.38) = -1.85 V vs SCE
Intensity /µA
Potential /V

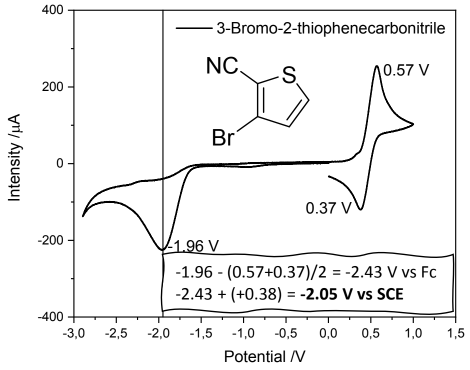
3-Bromo-2-thiophenecarbonitrile
0.57 V
0.37 V
-1.96 V
-1.96 - (0.57+0.37)/2 = -2.43 V vs Fc
-2.43 + (+0.38) = -2.05 V vs SCE
Intensity /µA
Potential /V

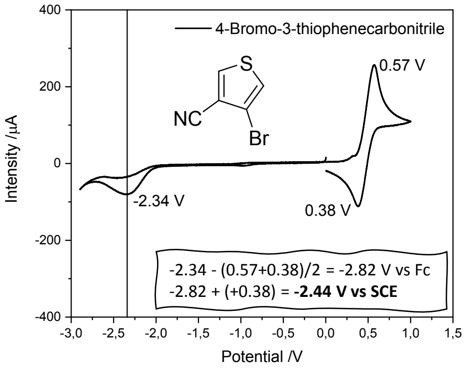
4-Bromo-3-thiophenecarbonitrile
0.57 V
0.38 V
-2.34 V
-2.34 - (0.57+0.38)/2 = -2.82 V vs Fc
-2.82 + (+0.38) = -2.44 V vs SCE
Intensity /µA
Potential /V

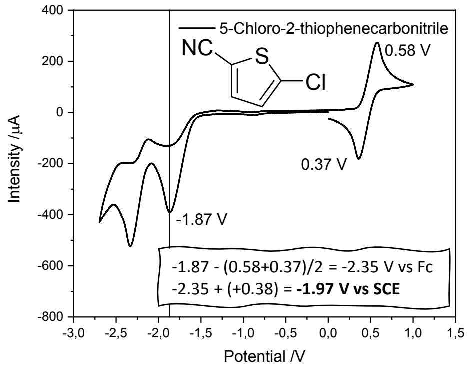
5-Chloro-2-thiophenecarbonitrile
0.58 V
0.37 V
-1.87 V
-1.87 - (0.58+0.37)/2 = -2.35 V vs Fc
-2.35 + (+0.38) = -1.97 V vs SCE
Intensity /µA
Potential /V

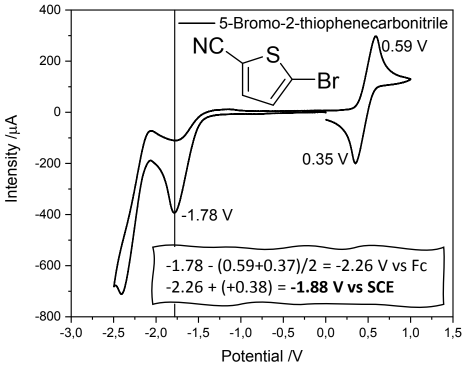
5-Bromo-2-thiophenecarbonitrile
0.59 V
0.35 V
-1.78 V
-1.78 - (0.59+0.37)/2 = -2.26 V vs Fc
-2.26 + (+0.38) = -1.88 V vs SCE
Intensity /µA
Potential /V

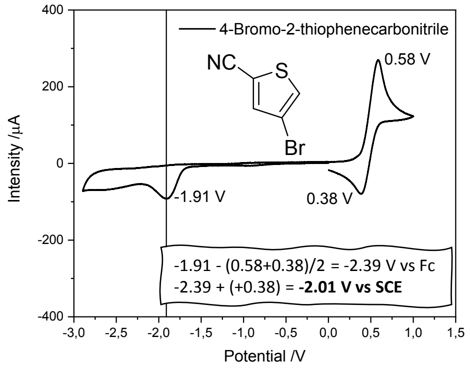
4-Bromo-2-thiophenecarbonitrile
0.58 V
0.38 V
-1.91 V
-1.91 - (0.58+0.38)/2 = -2.39 V vs Fc
-2.39 + (+0.38) = -2.01 V vs SCE
Intensity /µA
Potential /V

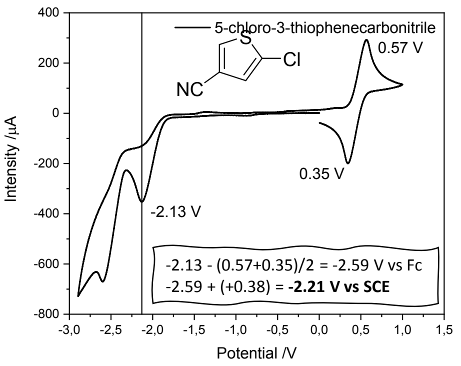
5-chloro-3-thiophenecarbonitrile
0.57 V
0.35 V
-2.13 V
-2.13 - (0.57+0.35)/2 = -2.59 V vs Fc
-2.59 + (+0.38) = -2.21 V vs SCE
Intensity /µA
Potential /V

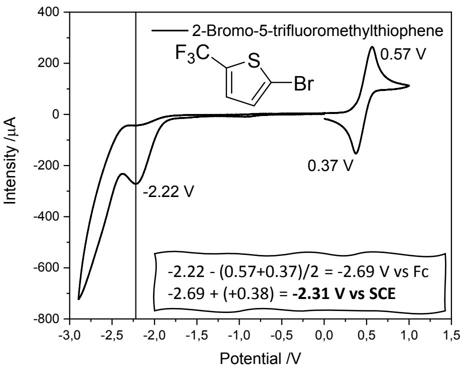
2-Bromo-5-trifluoromethylthiophene
0.57 V
0.37 V
-2.22 V
-2.22 - (0.57+0.37)/2 = -2.69 V vs Fc
-2.69 + (+0.38) = -2.31 V vs SCE
Intensity /µA
Potential /V

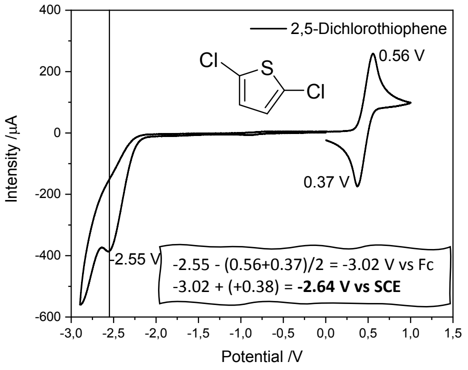
2,5-Dichlorothiophene
0.56 V
0.37 V
-2.55 V
-2.55 - (0.56+0.37)/2 = -3.02 V vs Fc
-3.02 + (+0.38) = **-2.64 V vs SCE**
Intensity /μA
Potential /V

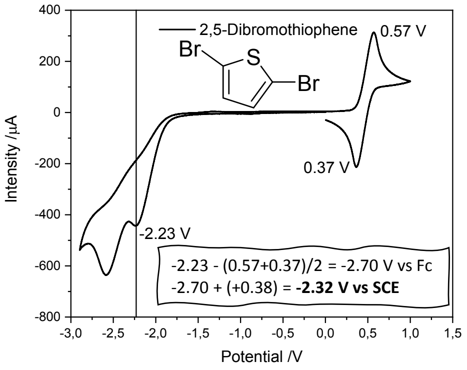
2,5-Dibromothiophene
0.57 V
0.37 V
-2.23 V
-2.23 - (0.57+0.37)/2 = -2.70 V vs Fc
-2.70 + (+0.38) = **-2.32 V vs SCE**
Intensity /μA
Potential /V

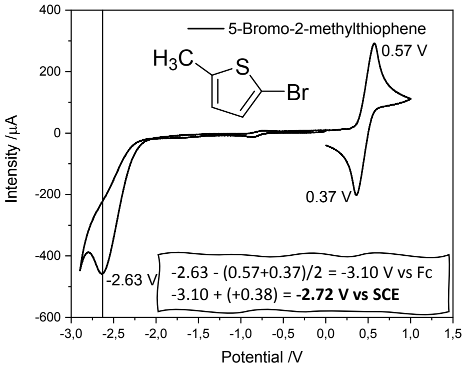
5-Bromo-2-methylthiophene
0.57 V
0.37 V
-2.63 V
-2.63 - (0.57+0.37)/2 = -3.10 V vs Fc
-3.10 + (+0.38) = **-2.72 V vs SCE**
Intensity /μA
Potential /V

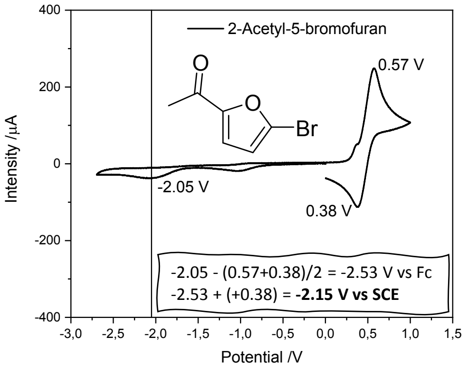
2-Acetyl-5-bromofuran
0.57 V
0.38 V
-2.05 V
-2.05 - (0.57+0.38)/2 = -2.53 V vs Fc
-2.53 + (+0.38) = **-2.15 V vs SCE**
Intensity /μA
Potential /V

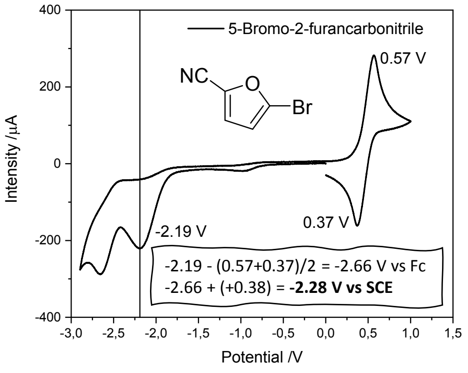
5-Bromo-2-furancarbonitrile
0.57 V
0.37 V
-2.19 V
-2.19 - (0.57+0.37)/2 = -2.66 V vs Fc
-2.66 + (+0.38) = **-2.28 V vs SCE**
Intensity /μA
Potential /V

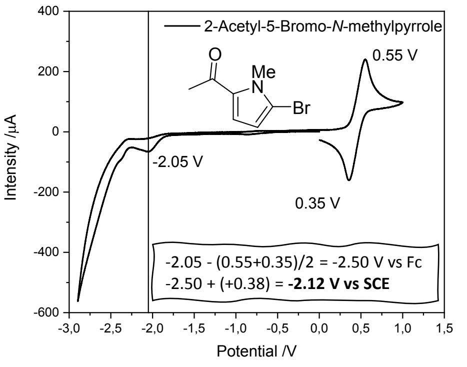
2-Acetyl-5-Bromo-*N*-methylpyrrole
0.55 V
0.35 V
-2.05 V
-2.05 - (0.55+0.35)/2 = -2.50 V vs Fc
-2.50 + (+0.38) = **-2.12 V vs SCE**
Intensity /μA
Potential /V

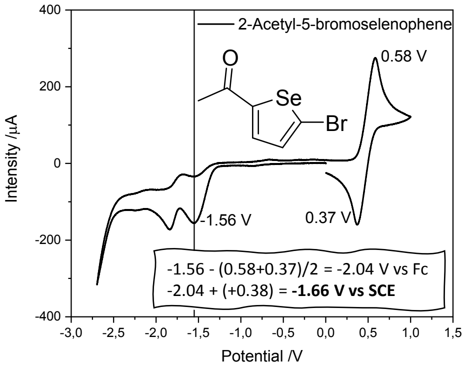
2-Acetyl-5-bromoselenophene
0.58 V
0.37 V
-1.56 V
-1.56 - (0.58+0.37)/2 = -2.04 V vs Fc
-2.04 + (+0.38) = **-1.66 V vs SCE**
Intensity /μA
Potential /V

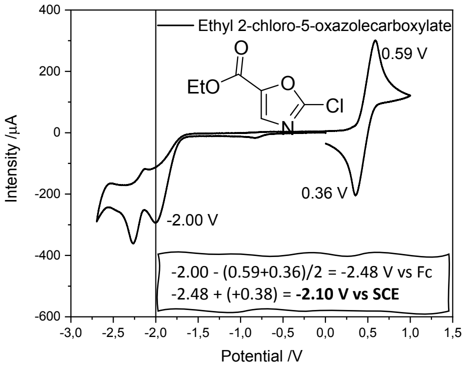
Ethyl 2-chloro-5-oxazolecarboxylate
0.59 V
0.36 V
-2.00 V
-2.00 - (0.59+0.36)/2 = -2.48 V vs Fc
-2.48 + (+0.38) = **-2.10 V vs SCE**
Intensity /μA
Potential /V

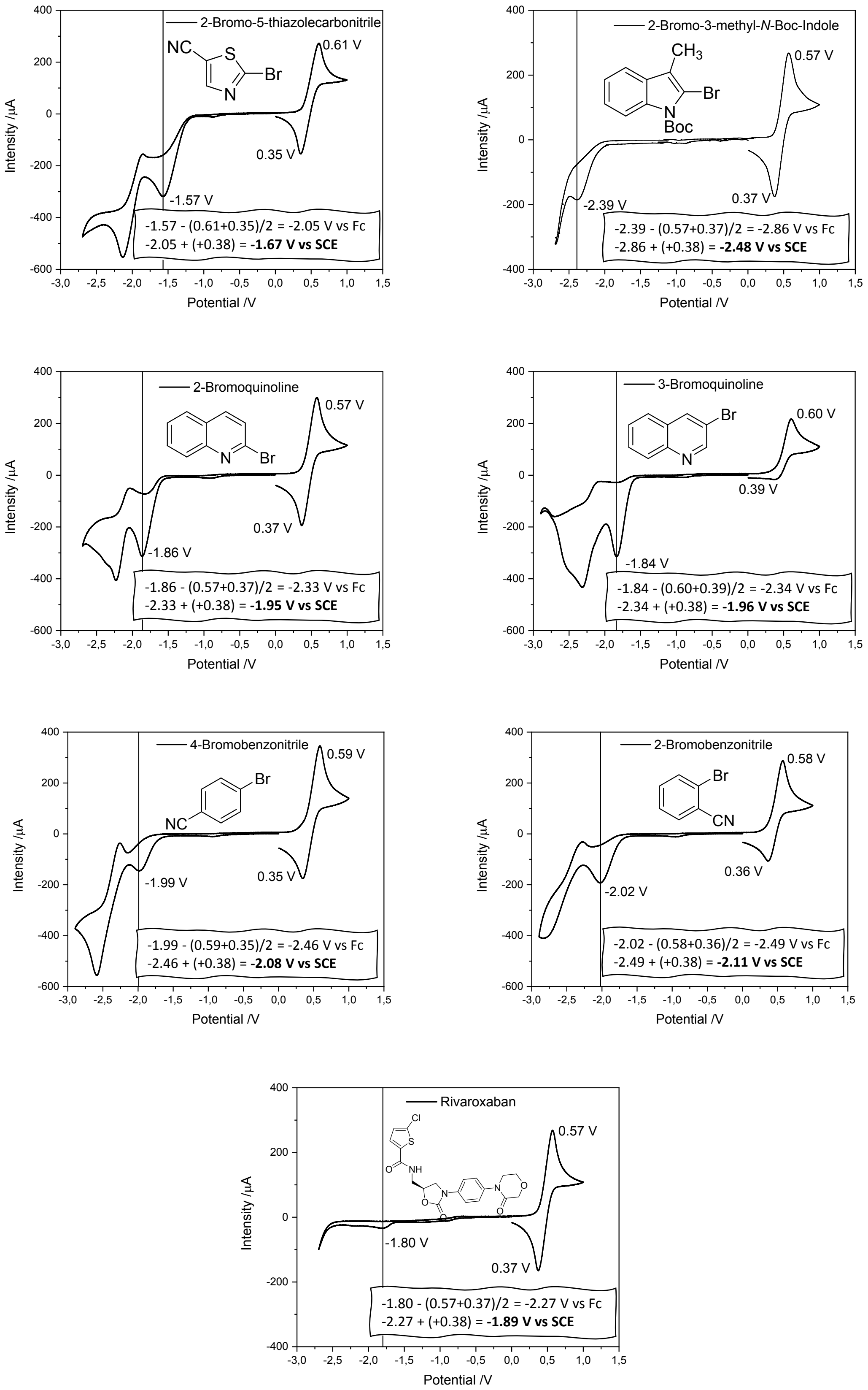

Figure S1. Cyclic voltammetry spectra of the corresponding (hetero)arene halides.

Kinetic studies

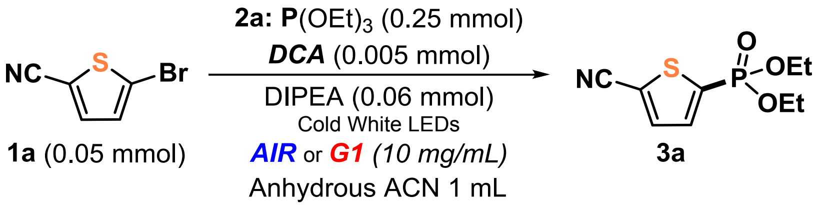

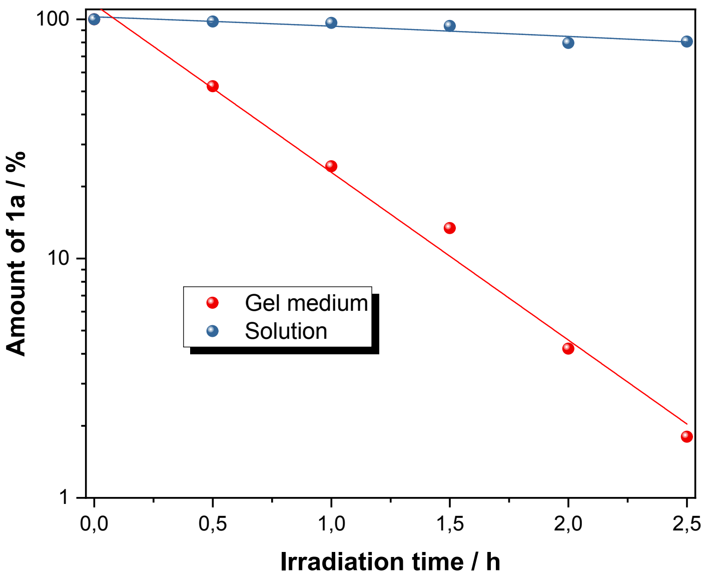

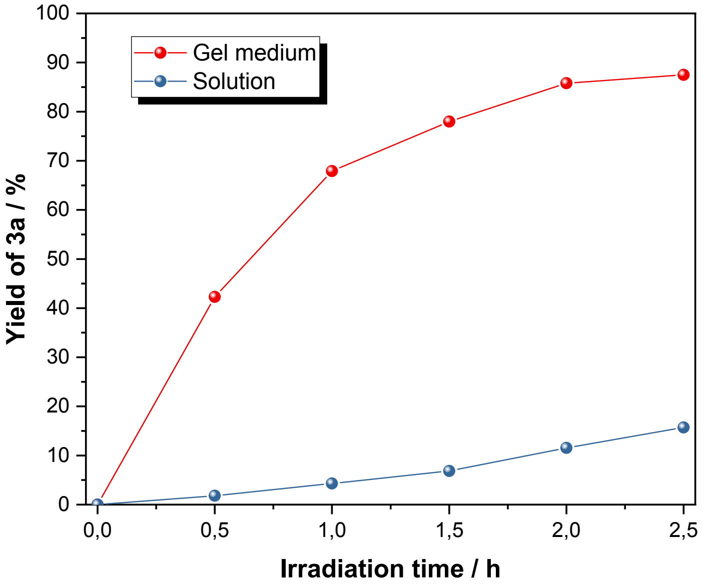

Figure S2. *Top:* Relationship between conversion of **1a** versus irradiation time. *Bottom:* Relationship between production of **3a** versus irradiation time.

Frozen model reaction procedure

Scheme S1. Frozen reaction conditions for the phosphorylation of **1a**.

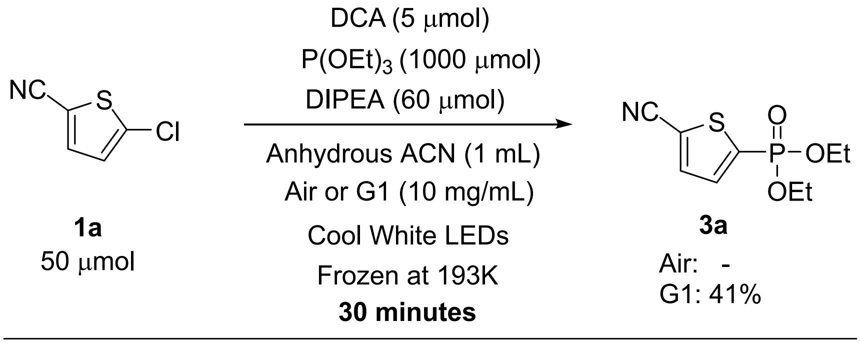

A vial (10 mL) was charged with 9,10-anthracenedicarbonitrile (1.2 mg, 5 μmol, 10 mol% and the correspondent gelator (G1, 10 mg). Anhydrous acetonitrile (1.0 mL) was poured and 5-chloro-2-thiophenecarbonitrile (5.4 μL, 50 μmol, 1.0 equiv.) and triethylphosphite (180 μL, 1000 μmol, 20.0 equiv.) were added. Then, DIPEA (10.5 μL, 60 μmol, 1.2 equiv.) and dodecanenitrile (12.0 μL, 50 μmol, 1.0 equiv.) were added with 25 μL Hamilton syringe. Quickly, the vial was sealed with a septum. It was heated to 150 °C with heatgun for 1.5 minutes with manual stirring until complete clear solution. The vial cooled to room temperature until gel formation was observed. The vial was frozen at 193K for 2 hours. Later, the frozen vials were irradiated with an external LED through the plain bottom side of the vial at 23 °C for 30 minutes. Finally, the reactions were monitored by GC-FID analysis. The gel was broken with ethyl acetate (1 mL), and it was clean with brine (2 mL). The organic phase was dried over anhydrous sodium sulfate, filtered from the drying agent, and concentrated in vacuo.

UV-vis absorption spectra and DCA fluorescence quenching

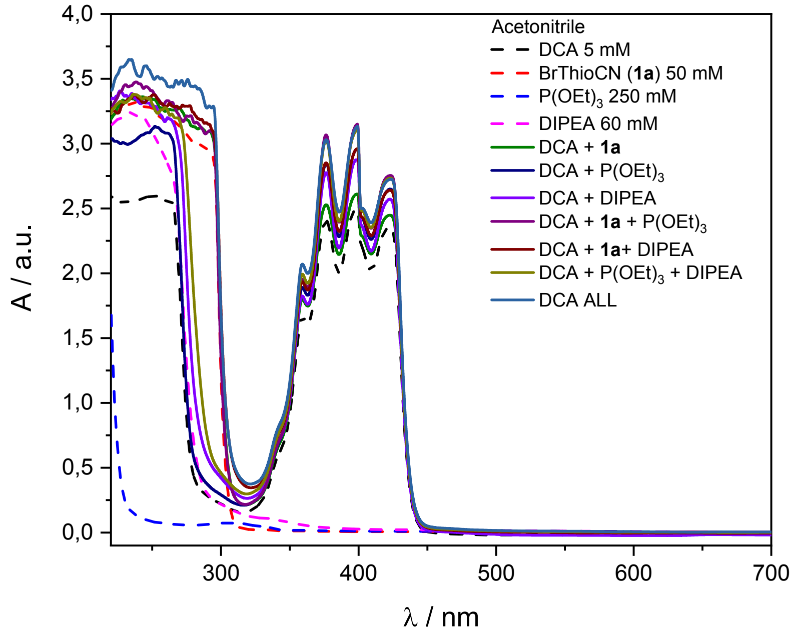

Figure S3. Absorption spectra of the corresponding photocatalyst and substrates in acetonitrile.

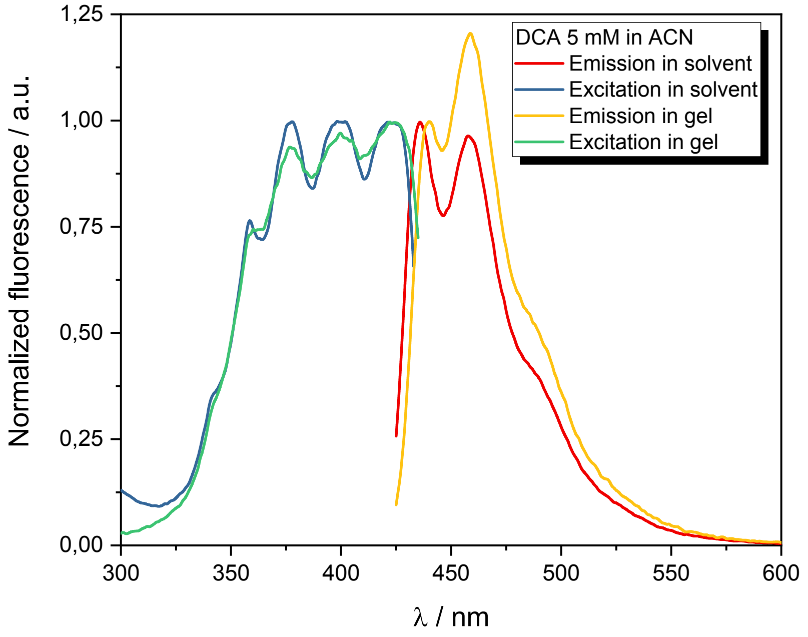

Figure S4. Emission and excitation spectra of DCA.

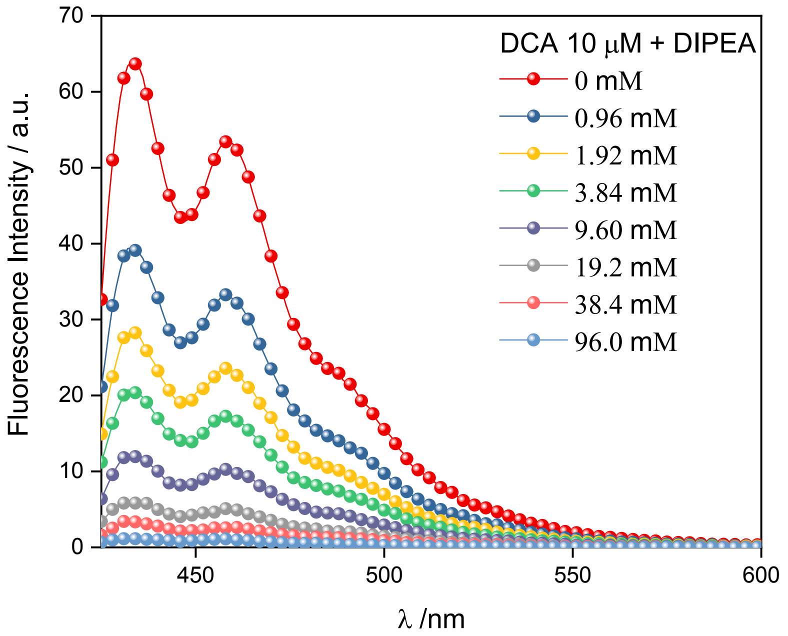

Figure S5. Emission spectra of DCA (10 μM) in the presence of increasing amounts of DIPEA in acetonitrile under aerobic conditions.

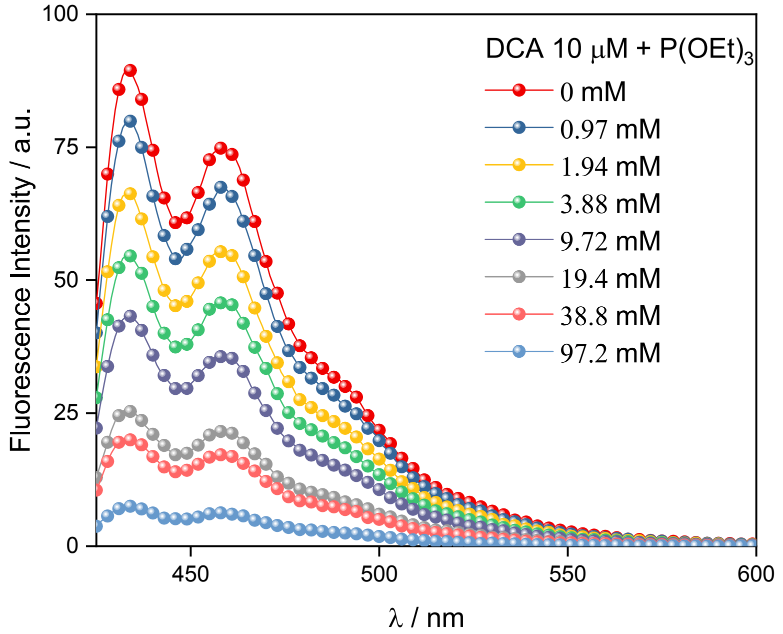

Figure S6. Emission spectra of DCA (10 μM) in the presence of increasing amounts of triethyl phosphite in acetonitrile under aerobic conditions.

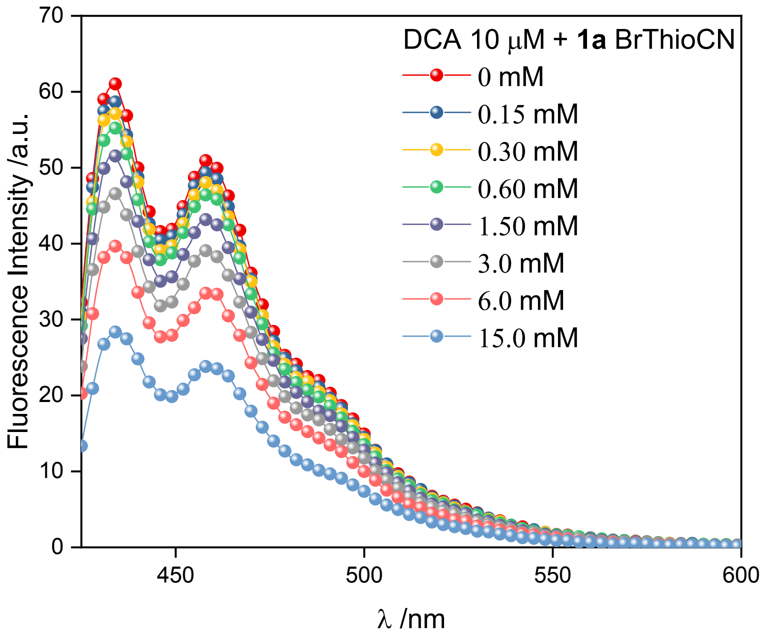

Figure S7. Emission spectra of DCA (10 μM) in the presence of increasing amounts of 2-bromo-5-thiophenecarbonitrile in acetonitrile under aerobic conditions.

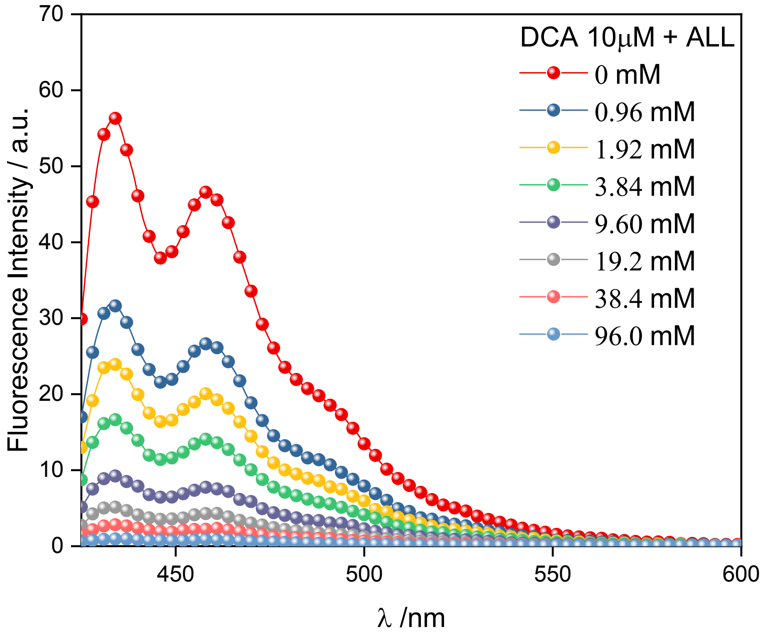

Figure S8. Emission spectra of DCA (10 μM) in the presence of increasing amounts of DIPEA + triethyl phosphite + 2-bromo-5-thiophenecarbonitrile in acetonitrile under aerobic conditions.

Trapping reaction

Scheme S2. Trapping reaction of **1a** by diphenyl sulfide.

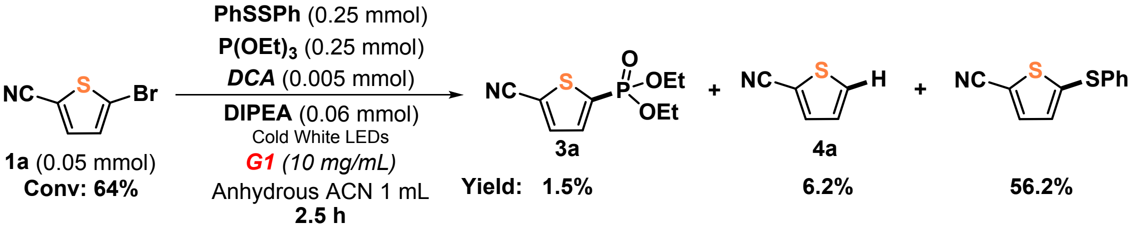

A vial (10 mL) was charged with 9,10-anthracenedicarbonitrile (1.2 mg, 5 μmol, 10 mol%), diphenyl sulfide (47.5 mg, 250 μmol, 5.0 equiv.) and the correspondent gelator (G1, 10 mg). Anhydrous acetonitrile (1.0 mL) was poured and 5-bromo-2-thiophenecarbonitrile (5.7 μL, 50 μmol, 1.0 equiv.) and triethylphosphite (45 μL, 250 μmol, 5.0 equiv.) were added. Then, DIPEA (10.5 μL, 60 μmol, 1.2 equiv.) and dodecanenitrile (12.0 μL, 50 μmol, 1.0 equiv.) were added with 25 μL Hamilton syringe. Quickly, the vial was sealed with a septum. It was heated to 150 °C with heatgun for 1.5 minutes with manual stirring until complete clear solution. The vial cooled to room temperature until gel formation was observed. The reaction was irradiated with an external LED through the plain bottom side of the vial at 23 °C for 2.5 hours. Then, brine (2 mL) was added, and the aqueous phase was extracted with ethyl acetate (1 mL). The reaction was monitored by GC-FID analysis obtaining. The organic phase was dried over anhydrous sodium sulfate, filtered from the drying agent, and concentrated in vacuo. The crude was purified via flash column chromatography using a hexane/ethyl acetate mixture as the mobile phase.

Sunlight irradiation and 1 mmol scale

Sunlight irradiation

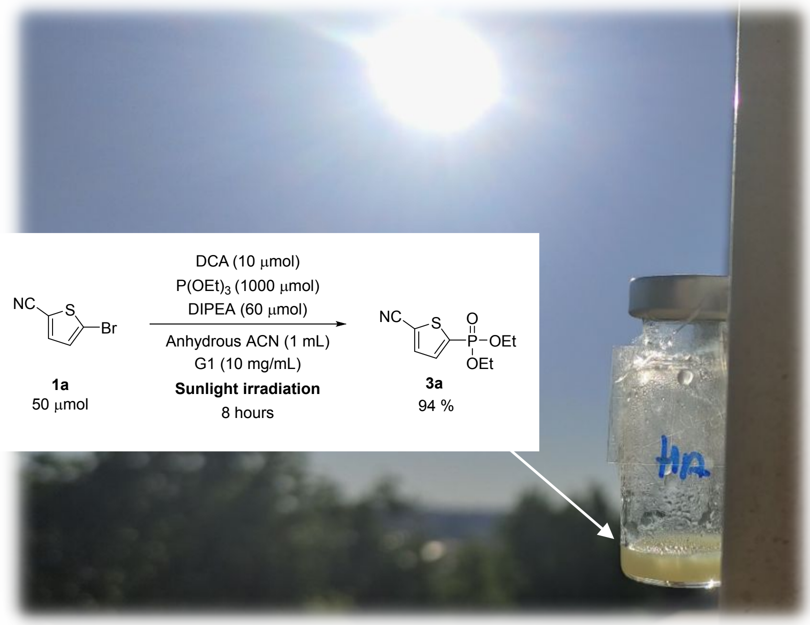

Figure S9. Photograph of the reaction performed by external sunlight irradiation.

The compound **3a** was prepared according to the general procedure using 9-10-dicyanoanthracene (2.4 mg, 10 µmol, 20 mol%) as photocatalyst, 5-bromo-2-thiophenecarbonitrile (5.7 µL, 50 µmol, 1.0 equiv.), triethylphosphite (180 µL, 1000 µmol, 20.0 equiv.), dodecanenitrile (12.0 µL, 50 µmol, 1.0 equiv.) as internal standard and DIPEA (10.5 µL, 60 µmol, 1.2 equiv.) and G1 (10 mg/mL). The gel mixture was irradiated with sunlight for 8 hours, obtaining 94% product yield according to GC-FID analysis.
Location: Camí de Vera S/N, Chemistry Department, Universitat Politècnica de València (UPV), Valencia, Spain (coordinate: 39.482917, -0.341642), temperature: 16-23 °C, from 9:00 to 17:00. Date: 24/05/2021

1 mmol scale

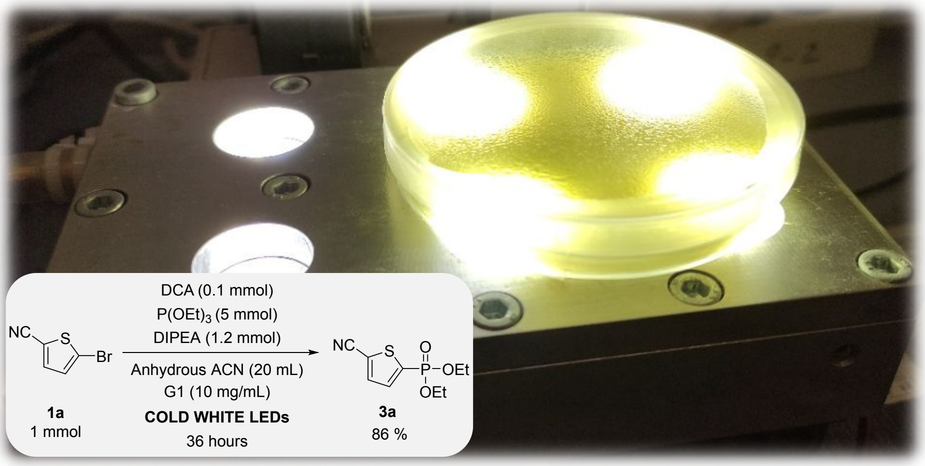

Figure S10. Photograph of the reaction performed by white light LEDs for the scaling-up conditions.

To scale-up the procedure (from 0.05 mmol to 1 mmol), we performed the reaction following the general procedure inside a covered petri dish using 9-10-dicyanoanthracene (24 mg, 0.1 mmol, 10 mol%) as photocatalyst, 5-bromo-2-thiophenecarbonitrile (114 µL, 1 mmol, 1.0 equiv.), triethylphosphite (900 µL, 5 mmol, 5.0 equiv.), dodecanenitrile (240 µL, 1 mmol, 1.0 equiv.) as internal standard and DIPEA (209 µL, 1.2 mmol, 1.2 equiv.) and G1 (200 mg/mL) in 20 mL of anhydrous acetonitrile. The reaction mixture was irradiated for 36 hours with cold white LEDs. Then, brine (30 mL) was added, and the aqueous phase was extracted with ethyl acetate (3 x 30 mL). The reaction was monitored by GC-FID analysis obtaining 86% product yield. The organic phase was dried over anhydrous sodium sulfate, filtered from the drying agent, and concentrated in vacuo. The crude was purified via flash column chromatography using a hexane/ethyl acetate mixture as the mobile phase obtaining 161.7 mg, 66% isolated yield.

Oscillatory rheology

Oscillatory rheology was performed with an AR 2000 Advanced rheometer (TA Instruments) equipped with a Julabo C cooling system. A 1000 µm gap setting and a torque setting of 40,000 dynes cm^{-2} at 25 ºC were used for the measurements in a plain-plate (40 mm, stainless steel).

The following experiments were performed using 2 mL total gel volume: a) Dynamic strain sweep (DSS): variation of G' and G'' with strain (from 0.01 to 100%); b) dynamic frequency sweep (DFS): variation of G' and G'' with frequency (from 0.1 to 10 Hz ar 0.1% strain).

Field-emission scanning electron microscopy (FESEM)

The equipment in operation in the UPV Microscopy Service is the ZEISS ULTRA 55 model, incorporating the following detectors:
·A Secondary Electron Detector (SE2), which provides an SEM topography image of the sample surface with a large depth of field.
· A Secondary Electron In-Lens Detector located inside the electron column, which works with low energy secondary electrons and provides images with a higher resolution.
· A Backscattered Electron Detector (AsB) which is sensitive to the variation of atomic number in the elements present in the sample; therefore, it is used to observe changes in the chemical composition of the specimen.
· A Backscattered Electron In-lens Detector (EsB), independent of the secondary In-lens detector, which provides a pure backscattered signal with no secondary electron contamination and very low acceleration potential.
· An X-Ray Dispersive Energy Detector, EDS, (Oxford Instruments) which receives x-rays from each surface point the electron beam passes over.

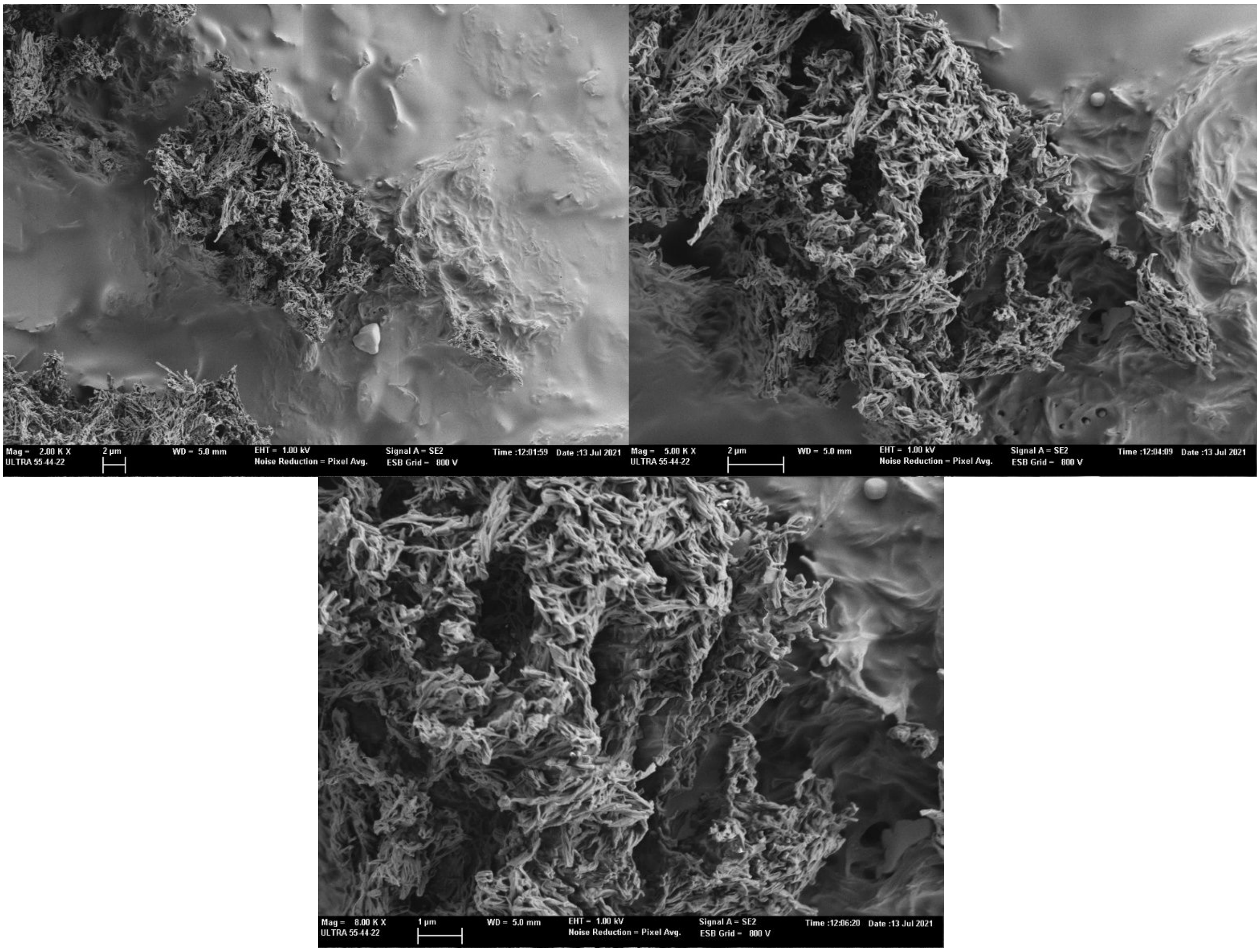

Figure S11. Representative images of FESEM of the unloaded **G1** before irradiation.

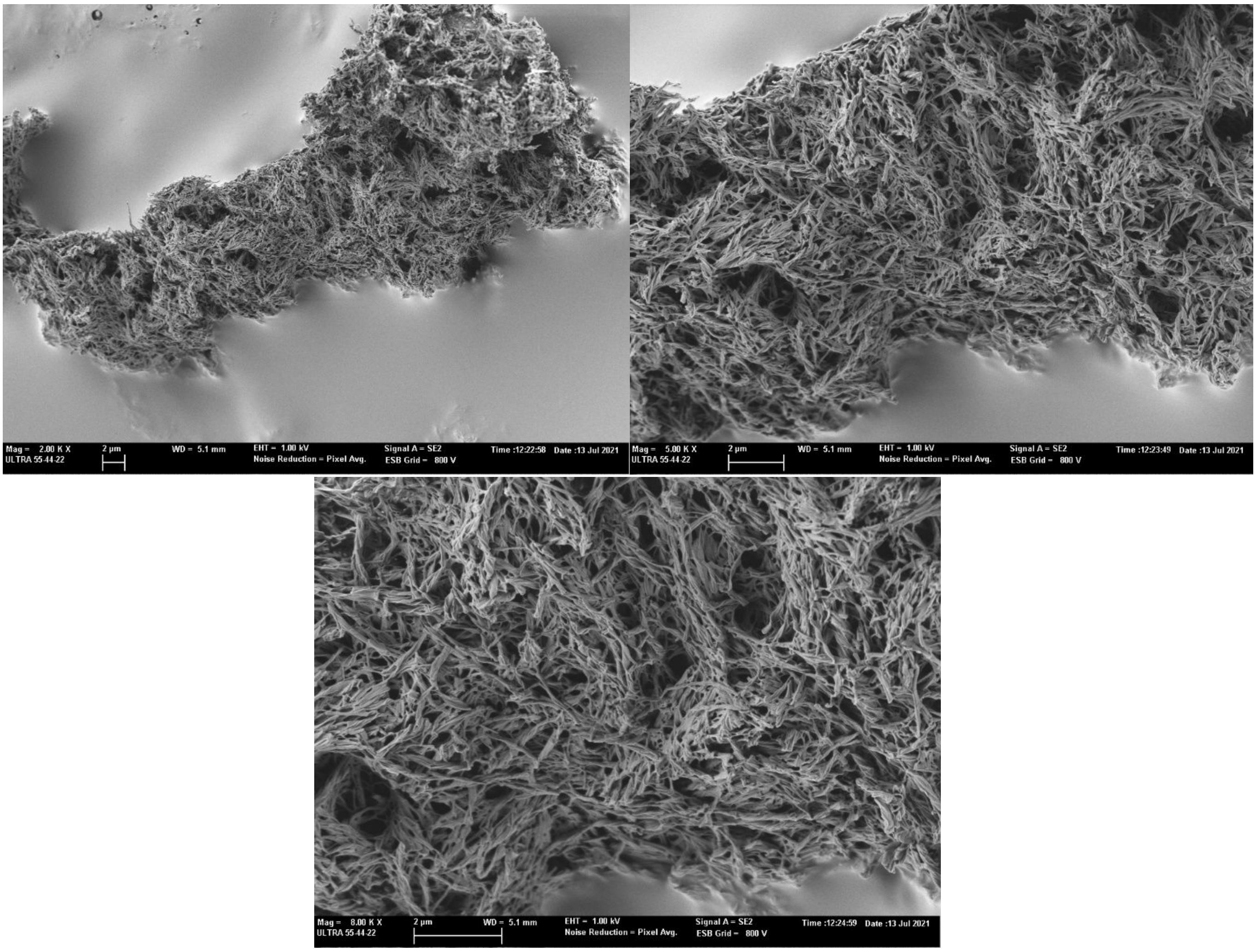

Figure S12. Representative images of FESEM of the unloaded **G1** after 2.5 hours of irradiation.

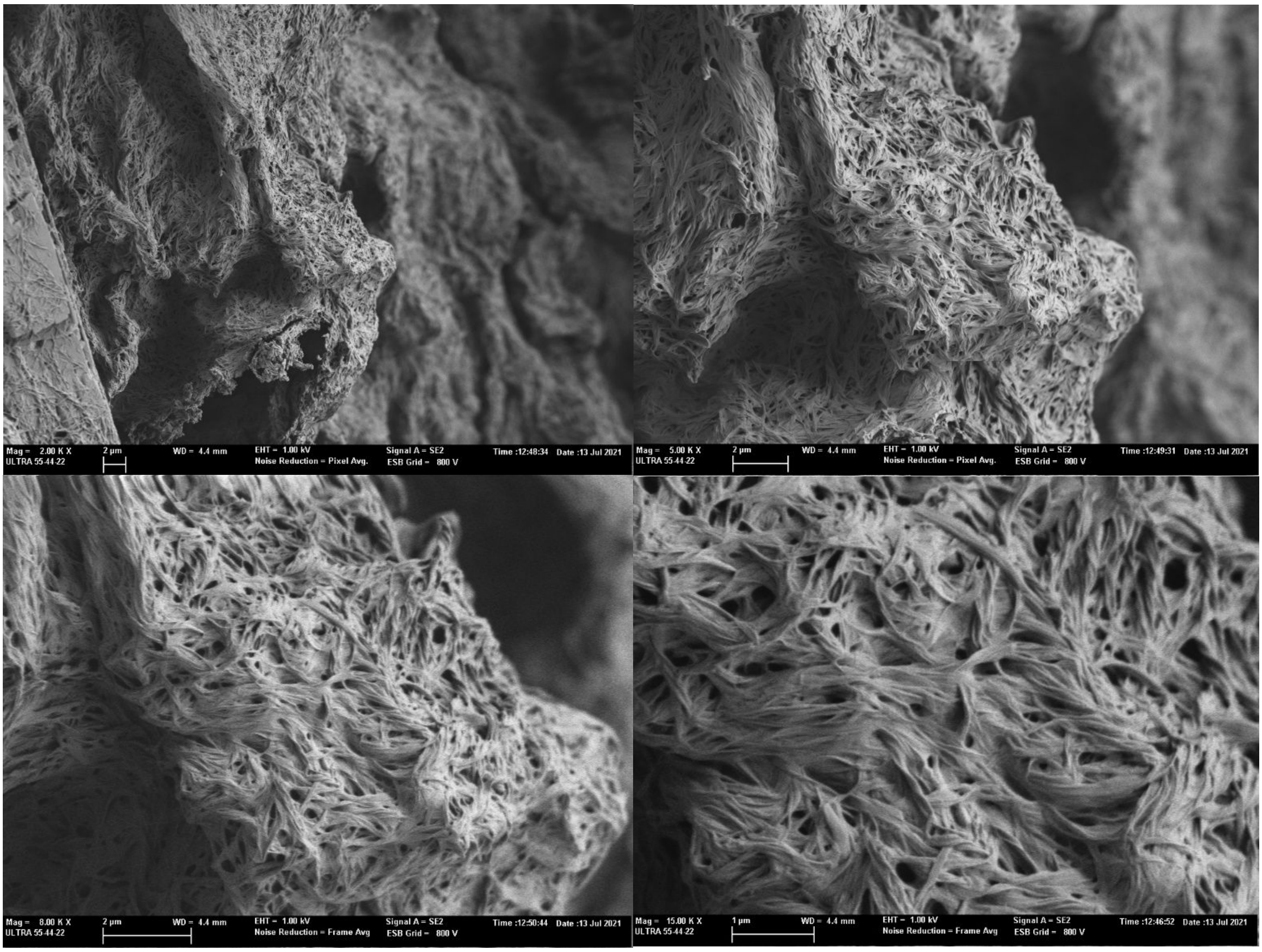

Figure S13. Representative images of FESEM of the loaded **G1** before irradiation.

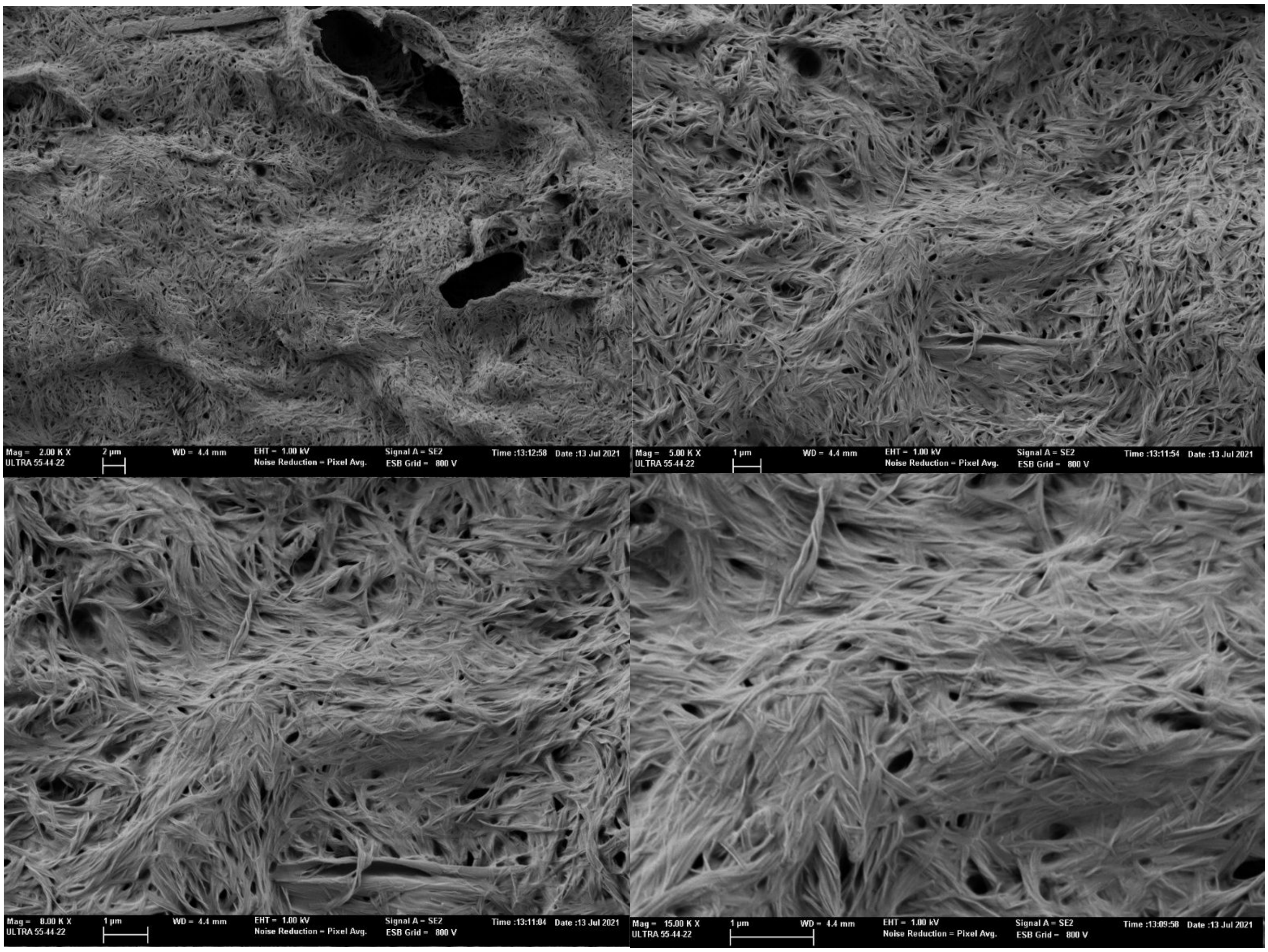

Figure S14. Representative images of FESEM of the loaded **G1** after 2.5 hours of irradiation.

Characterization of compounds

THIOPHENES

3a. Diethyl (5-cyanothiophen-2-yl)phosphonate

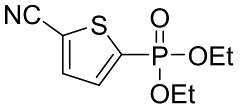

The compound was prepared according to the general procedure using 9-10-dicyanoanthracene (1.2 mg, 5 µmol, 0.1 equiv.), 5-bromo-2-thiophenecarbonitrile (5.7 µL, 50 µmol, 1.0 equiv.), triethylphosphite (45 µL, 250 µmol, 5 equiv.), dodecanenitrile (12 µL, 50 µmol, 1.0 equiv.) as internal standard and DIPEA (10.5 µL, 60 µmol, 1.2 equiv.) and G1 (10 mg/mL). The gel mixture was irradiated for 2.5 hours, obtaining **90%** product yield according to GC-FID analysis (75% isolated yield as yellow oil). Using 5-chloro-2-thiophenecarbonitrile, the gel mixture was irradiated for 4 hours, obtaining 87% product yield according to GC-FID analysis.

^{1}H NMR (400 MHz, $CDCl_3$) δ 7.64 (t, *J* = 3.5 Hz, 1H), 7.59 (dd, *J* = 8.1, 3.8 Hz, 1H), 4.26 – 4.07 (m, 4H), 1.35 (t, *J* = 7.1 Hz, 6H) ppm.
^{13}C NMR (101 MHz, $CDCl_3$) δ 137.6 (CH, d, *J* = 16.8 Hz), 136.8 (C, d, *J* = 204.5 Hz), 135.7 (CH, d, *J* = 10.7 Hz), 116.8 (C, d, *J* = 10.0 Hz), 113.1 (C, d, *J* = 3.0 Hz), 63.6 (CH_2, d, *J* = 5.6 Hz), 16.4 (CH_3, d, *J* = 6.5 Hz) ppm.
^{31}P NMR (162 MHz, $CDCl_3$) δ 7.48 (s) ppm.
HRMS (EI): m/z $(M+H)^+$ = calcd. for $C_9H_{12}NO_3PS$: 246.0348, found: 246.0347.

3b. Dimethyl (5-cyanothiophen-2-yl)phosphonate

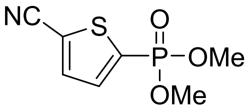

The compound was prepared according to the general procedure using 9-10-dicyanoanthracene (1.2 mg, 5 µmol, 0.1 equiv.), 5-bromo-2-thiophenecarbonitrile (5.7 µL, 50 µmol, 1.0 equiv.), trimethylphosphite (30.4 µL, 250 µmol, 5 equiv.), dodecanenitrile (12 µL, 50 µmol, 1.0 equiv.) as internal standard and DIPEA (10.5 µL, 60 µmol, 1.2 equiv.) and G1 (10 mg/mL). The gel mixture was irradiated for 2 hours, obtaining **97**% product yield according to GC-FID analysis (80% isolated yield as yellow oil).

^{1}H NMR (400 MHz, $CDCl_3$) δ 7.68 – 7.64 (m, 1H), 7.61 (dd, *J* = 8.1, 3.8 Hz, 1H), 3.84 (s, 3H), 3.81 (s, 3H) ppm.
^{13}C NMR (101 MHz, $CDCl_3$) δ 137.7 (CH, d, *J* = 17.0 Hz), 136.2 (CH, d, *J* = 10.8 Hz), 135.0 (C, d, *J* = 206.0 Hz), 53.7 (CH_3, dd, *J* = 5.5, 2.2 Hz) ppm.
^{31}P NMR (162 MHz, $CDCl_3$) δ 10.47 (s) ppm.
HRMS (EI): m/z $(M+H)^+$ = calcd. for $C_7H_8NO_3PS$: 218.0035, found: 218.0036.

3c. Diphenyl (5-cyanothiophen-2-yl)phosphonate

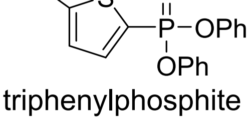

The compound was prepared according to the general procedure using 9-10-dicyanoanthracene (1.2 mg, 5 µmol, 0.1 equiv.), 5-bromo-2-thiophenecarbonitrile (5.7 µL, 50 µmol, 1.0 equiv.), triphenylphosphite (67.5 µL, 250 µmol, 5 equiv.), dodecanenitrile (12 µL, 50 µmol, 1.0 equiv.) as internal standard and DIPEA (10.5 µL, 60 µmol, 1.2 equiv.) and G1 (10 mg/mL). The gel mixture was irradiated for 10 hours, obtaining **60%** product yield according to GC-FID analysis (45% isolated yield as yellow powder).

^{1}H NMR (400 MHz, $CDCl_3$) δ 7.71 (dd, *J* = 8.4, 3.8 Hz, 1H), 7.64 (t, *J* = 3.7 Hz, 1H), 7.35 (dd, *J* = 11.1, 4.7 Hz, 4H), 7.21 (dd, *J* = 11.4, 4.0 Hz, 6H) ppm.

^{13}C NMR (101 MHz, $CDCl_3$) δ 149.9 (C, d, J = 9.7 Hz), 137.6 (CH, d, J = 15.6 Hz), 137.3 (CH, d, J = 11.3 Hz), 134.2 (C, d, J = 212.8 Hz), 130.2 (CH, s), 126.1 (CH, d, J = 1.2 Hz), 120.57 (CH, d, J = 4.7 Hz), 118.2 (C, d, J = 10.7 Hz), 112 (C, d, J = 3.0 Hz) ppm.
^{31}P NMR (162 MHz, $CDCl_3$) δ -0.18 (s) ppm.
HRMS (EI): m/z $(M+H)^+$ = calcd. for $C_{17}H_{12}NO_3PS$: 342.0348, found: 342.0344.

3d. Diphenyl (5-acetylthiophen-2-yl)phosphonate

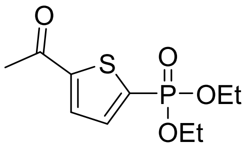

The compound (CAS: 1119779-20-2) was prepared according to the general procedure using 9-10-dicyanoanthracene (1.2 mg, 5 µmol, 0.1 equiv.), 2-acetyl-5-bromothiophene (4 mg, 50 µmol, 1.0 equiv.), triethylphosphite (45 µL, 250 µmol, 5 equiv.), dodecanenitrile (12 µL, 50 µmol, 1.0 equiv.) as internal standard and DIPEA (10.5 µL, 60 µmol, 1.2 equiv.) and G1 (10 mg/mL). The gel mixture was irradiated for 4 hours, obtaining **91%** product yield according to GC-FID analysis (78% isolated yield as yellow oil). Using 2-acetyl-5-chlorothiophene and 20 equivalents of triethylphosphite, the gel mixture was irradiated for 24 hours, obtaining 58% product yield according to GC-FID analysis.

^{1}H NMR (400 MHz, $CDCl_3$) δ 7.68 (t, J = 3.3 Hz, 1H), 7.63 (dd, J = 7.9, 3.7 Hz, 1H), 4.27 – 4.06 (m, 4H), 2.59 (s, 3H), 1.35 (t, J = 7.1 Hz, 6H) ppm.
^{13}C NMR (101 MHz, $CDCl_3$) δ 190.6 (C, d, J = 1.6 Hz), 150.6 (C, d, J = 7.2 Hz), 136.7 (CH, d, J = 11.5 Hz), 136.4 (C, d, J = 203.1 Hz), 132.1 (CH, d, J = 17.0 Hz), 63.2 (CH_2, d, J = 5.5 Hz), 27.3 (CH_3, s), 16.4 (CH_3, d, J = 6.6 Hz) ppm.
^{31}P NMR (162 MHz, $CDCl_3$) δ 9.61 (s) ppm.
HRMS (EI): m/z $(M+H)^+$ = calcd. for $C_{10}H_{15}O_4PS$: 263.0501, found: 263.0505.

3e. Diethyl (5-(trifluoromethyl)thiophen-2-yl)phosphonate

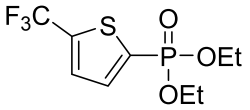

The compound was prepared according to the general procedure using 9-10-dicyanoanthracene (1.2 mg, 5 µmol, 0.1 equiv.), 2-bromo-5-(trifluoromethyl)thiophene (7.2 µL, 50 µmol, 1.0 equiv.), triethylphosphite (45 µL, 250 µmol, 5 equiv.), dodecanenitrile (12 µL, 50 µmol, 1.0 equiv.) as internal standard and DIPEA (10.5 µL, 60 µmol, 1.2 equiv.) and G1 (10 mg/mL). The gel mixture was irradiated for 4 hours, obtaining **94%** product yield according to GC-FID analysis (71% isolated yield as yellow oil).

^{1}H NMR (400 MHz, $CDCl_3$) δ 7.58 (ddd, J = 8.1, 3.7, 1.2 Hz, 1H), 7.48 (td, J = 3.6, 1.0 Hz, 1H), 4.26 – 4.07 (m, 4H), 1.35 (td, J = 7.1, 0.4 Hz, 6H) ppm.
^{13}C NMR (101 MHz, $CDCl_3$) δ 138.6 (C, dq, J = 38.6, 8.5 Hz), 135.9 (CH, d, J = 11.3 Hz), 132.9 (C, dd, J = 206.1, 1.0 Hz), 129.7 – 128.9 (CH, m), 122.0 (CF_3, dq, J = 269.8, 2.9 Hz), 63.3 (d, J = 5.5 Hz), 16.4 (d, J = 6.6 Hz) ppm.
^{19}F NMR (376 MHz, $CDCl_3$) δ -55.65 (s) ppm.
^{31}P NMR (162 MHz, $CDCl_3$) δ 8.98 (s) ppm.
HRMS (EI): m/z $(M+H)^+$ = calcd. for $C_9H_{12}F_3O_3PS$: 289.0270, found: 289.0271.

3f. Diethyl (5-methylthiophen-2-yl)phosphonate

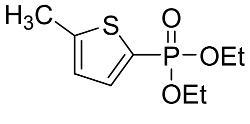

The compound was prepared according to the general procedure using 9-10-dicyanoanthracene (2.4 mg, 10 µmol, 0.2 equiv.), 2-bromo-5-methylthiophene (6.0 µL, 50 µmol, 1.0 equiv.), triethylphosphite (180 µL, 1000 µmol, 20 equiv.), dodecanenitrile (12 µL, 50 µmol, 1.0 equiv.) as internal standard and DIPEA (10.5 µL, 60 µmol, 1.2

equiv.) and G1 (10 mg/mL). The gel mixture was irradiated for 24 hours, obtaining **42%** product yield according to GC-FID analysis (25% isolated yield as yellow oil).

^{1}H NMR (400 MHz, $CDCl_3$) δ 7.47 (dd, *J* = 8.5, 3.5 Hz, 1H), 6.83 (td, *J* = 3.5, 1.0 Hz, 1H), 4.21 – 4.03 (m, 4H), 2.54 (d, *J* = 0.8 Hz, 3H), 1.33 (t, *J* = 7.1 Hz, 6H) ppm.
^{13}C NMR (101 MHz, $CDCl_3$) δ 149.1 (C, d, *J* = 7.1 Hz), 137.5 (CH, d, *J* = 11.6 Hz), 126.8 (CH, d, *J* = 17.1 Hz), 124.1 (C, d, *J* = 213.1 Hz), 62.6 (CH_2, d, *J* = 5.3 Hz), 16.4 (CH_3, d, *J* = 6.7 Hz), 15.5 (CH_3, s).
^{31}P NMR (162 MHz, $CDCl_3$) δ 12.09 (s) ppm.
HRMS (EI): m/z $(M+H)^+$ = calcd. for $C_9H_{15}O_3PS$: 235.0552, found: 235.0554.

3g. Tetraethyl thiophene-2,5-diylbis(phosphonate)

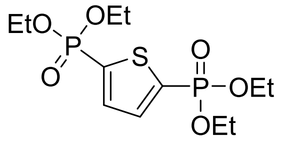

The compound (CAS: 100651-98-7) was prepared according to the general procedure using 9-10-dicyanoanthracene (1.2 mg, 5 µmol, 0.1 equiv.), 2,5-dibromothiophene (5.9 µL, 50 µmol, 1.0 equiv.), triethylphosphite (180 µL, 1000 µmol, 20 equiv.), dodecanenitrile (12 µL, 50 µmol, 1.0 equiv.) as internal standard and DIPEA (10.5 µL, 60 µmol, 1.2 equiv.) and G1 (10 mg/mL). The gel mixture was irradiated for 10 hours, obtaining 96% product yield according to GC-FID analysis (81% isolated yield as colorless oil). Using 2,5-dichlorothiophene (5.4 µL, 50 µmol, 1.0 equiv.) and 9-10-dicyanoanthracene (2.4 mg, 10 µmol, 0.2 equiv.), the gel mixture was irradiated for 10 hours, obtaining **73%** product yield according to GC-FID analysis.

^{1}H NMR (400 MHz, $CDCl_3$) δ 7.65 (d, *J* = 4.8 Hz, 1H), 7.64 (d, *J* = 4.8 Hz, 1H), 4.27 – 4.06 (m, 8H), 1.35 (t, *J* = 7.1 Hz, 12H) ppm.
^{13}C NMR (101 MHz, $CDCl_3$) δ 136.8 – 136.4 (CH, m), 136.5 (C, dd, *J* = 203.2, 6.2 Hz), 63.3 (CH_2, dd, *J* = 4.1, 1.0 Hz), 16.4 (CH_3, dd, *J* = 4.8, 1.7 Hz) ppm.
^{31}P NMR (162 MHz, $CDCl_3$) δ 9.55 (s) ppm.
HRMS (EI): m/z $(M+H)^+$ = calcd. for $C_{12}H_{22}O_6P_2S$: 357.0685, found: 357.0683.

3h. Diethyl (5-cyanothiophen-3-yl)phosphonate

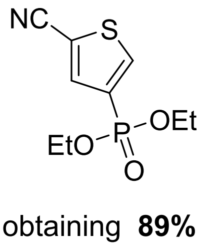

The compound was prepared according to the general procedure using 9-10-dicyanoanthracene (2.4 mg, 10 µmol, 0.2 equiv.), 4-bromo-2-thiophenecarbonitrile (9.4 mg, 50 µmol, 1.0 equiv.), triethylphosphite (180 µL, 1000 µmol, 20 equiv.), dodecanenitrile (12 µL, 50 µmol, 1.0 equiv.) as internal standard and DIPEA (10.5 µL, 60 µmol, 1.2 equiv.) and G1 (10 mg/mL). The gel mixture was irradiated for 21 hours, obtaining **89%** product yield according to GC-FID analysis (72% isolated yield as colorless oil).

^{1}H NMR (400 MHz, $CDCl_3$) δ 8.16 (dd, *J* = 8.3, 1.3 Hz, 1H), 7.81 (dd, *J* = 4.5, 1.3 Hz, 1H), 4.25 – 4.04 (m, 4H), 1.39 – 1.29 (m, 6H) ppm.
^{13}C NMR (101 MHz, $CDCl_3$) δ 140.7 (CH, d, *J* = 16.7 Hz), 139.2 (CH, d, *J* = 16.3 Hz), 132.0 (C, d, *J* = 199.5 Hz), 113.1 (C, s), 112.3 (C, d, *J* = 21.9 Hz), 63.0 (CH_2, d, *J* = 5.6 Hz), 16.5 (CH_3, d, *J* = 6.4 Hz) ppm.
^{31}P NMR (162 MHz, $CDCl_3$) δ 9.25 (s) ppm.
HRMS (EI): m/z $(M+H)^+$ = calcd. for $C_9H_{12}NO_3PS$: 246.0348, found: 246.0344.

3i. Diethyl (5-cyanothiophen-3-yl)phosphonate

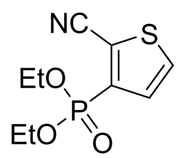

The compound (CAS: 1605306-51-1) was prepared according to the general procedure using 9-10-dicyanoanthracene (2.4 mg, 10 µmol, 0.2

equiv.), 3-bromo-2-thiophenecarbonitrile (5.7 µL, 50 µmol, 1.0 equiv.), triethylphosphite (180 µL, 1000 µmol, 20 equiv.), dodecanenitrile (12 µL, 50 µmol, 1.0 equiv.) as internal standard and DIPEA (10.5 µL, 60 µmol, 1.2 equiv.) and G1 (10 mg/mL). The gel mixture was irradiated for 2.5 hours, obtaining **96%** product yield according to GC-FID analysis (81% isolated yield as pale-yellow oil).

^{1}H NMR (400 MHz, $CDCl_3$) δ 7.64 (dd, *J* = 5.1, 3.0 Hz, 1H), 7.48 (t, *J* = 5.0 Hz, 1H), 4.31 – 4.08 (m, 4H), 1.38 (td, *J* = 7.1, 0.5 Hz, 6H) ppm.
^{13}C NMR (101 MHz, $CDCl_3$) δ 139.7 (C, d, *J* = 193.4 Hz), 132.4 (CH, d, *J* = 19.3 Hz), 132.0 (C, d, *J* = 14.6 Hz), 116.1 (C, d, *J* = 11.3 Hz), 112.6 (C, d, *J* = 3.8 Hz), 63.4 (CH_2, d, *J* = 5.9 Hz), 16.4 (CH_3, d, *J* = 6.4 Hz) ppm.
^{31}P NMR (162 MHz, $CDCl_3$) δ 7.14 (s) ppm.
HRMS (EI): m/z $(M+H)^+$ = calcd. for $C_9H_{12}NO_3PS$: 246.0348, found: 246.0344.

3j. Diethyl (4-cyanothiophen-2-yl)phosphonate

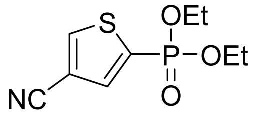

The compound was prepared according to the general procedure using 9-10-dicyanoanthracene (1.2 mg, 5 µmol, 0.1 equiv.), 5-chloro-3-thiophenecarbonitrile (7.6 mg, 50 µmol, 1.0 equiv.), triethylphosphite (45 µL, 250 µmol, 5 equiv.), dodecanenitrile (12 µL, 50 µmol, 1.0 equiv.) as internal standard and DIPEA (10.5 µL, 60 µmol, 1.2 equiv.) and G1 (10 mg/mL). The gel mixture was irradiated for 24 hours, obtaining **66%** product yield according to GC-FID analysis (47% isolated yield as colorless oil).

^{1}H NMR (400 MHz, $CDCl_3$) δ 8.21 (dd, *J* = 5.6, 1.2 Hz, 1H), 7.77 (dd, *J* = 8.1, 1.2 Hz, 1H), 4.30 – 4.08 (m, 4H), 1.36 (td, *J* = 7.1, 0.5 Hz, 6H) ppm.
^{13}C NMR (101 MHz, $CDCl_3$) δ 141.6 (CH, d, *J* = 6.4 Hz), 137.1 (CH, d, *J* = 12.4 Hz), 132.2 (C, d, *J* = 208.5 Hz), 114.1 (C, d, *J* = 1.3 Hz), 112.2 (C, d, *J* = 21.2 Hz), 63.5 (CH_2, d, *J* = 5.6 Hz), 16.4 (CH_3, d, *J* = 6.4 Hz) ppm.
^{31}P NMR (162 MHz, $CDCl_3$) δ 7.87 (s) ppm.
HRMS (EI): m/z $(M+H)^+$ = calcd. for $C_9H_{12}NO_3PS$: 246.0348, found: 246.0344.

3k. Diethyl (4-cyanothiophen-3-yl)phosphonate

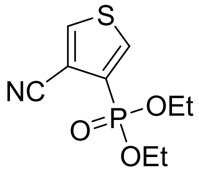

The compound was prepared according to the general procedure using 9-10-dicyanoanthracene (1.2 mg, 5 µmol, 0.1 equiv.), 4-bromo-3-thiophenecarbonitrile (9.7 mg, 50 µmol, 1.0 equiv.), triethylphosphite (180 µL, 250 µmol, 5 equiv.), dodecanenitrile (12 µL, 50 µmol, 1.0 equiv.) as internal standard and DIPEA (10.5 µL, 60 µmol, 1.2 equiv.) and G1 (10 mg/mL). The gel mixture was irradiated for 8 hours, obtaining **63%** product yield according to GC-FID analysis (51% isolated yield as colorless oil).

^{1}H NMR (400 MHz, $CDCl_3$) δ 8.13 (dd, *J* = 8.5, 3.1 Hz, 1H), 8.05 (t, *J* = 3.1 Hz, 1H), 4.32 – 4.06 (m, 4H), 1.37 (td, *J* = 7.1, 0.4 Hz, 6H) ppm.
^{13}C NMR (101 MHz, $CDCl_3$) δ 138.3 (CH, d, *J* = 15.2 Hz), 138.0 (CH, d, *J* = 16.2 Hz), 132.08 (C, d, *J* = 197.8 Hz), 113.67 (C, s), 112.55 (C, d, *J* = 12.4 Hz), 63.27 (CH_2, d, *J* = 5.9 Hz), 16.38 (CH_3, d, *J* = 6.4 Hz).
^{31}P NMR (162 MHz, $CDCl_3$) δ 7.14 (s) ppm.
HRMS (EI): m/z $(M+H)^+$ = calcd. for $C_9H_{12}NO_3PS$: 246.0348, found: 246.0346.

3l. Diethyl (5-acetylthiophen-3-yl)phosphonate

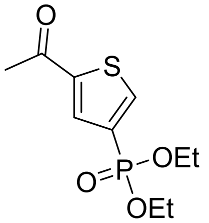

The compound was prepared according to the general procedure using 9-10-dicyanoanthracene (2.4 mg, 10 µmol, 0.2 equiv.), 2-acetyl-4-

bromothiophene (6.4 µL, 50 µmol, 1.0 equiv.), triethylphosphite (180 µL, 1000 µmol, 20 equiv.), dodecanenitrile (12 µL, 50 µmol, 1.0 equiv.) as internal standard and DIPEA (10.5 µL, 60 µmol, 1.2 equiv.) and G1 (10 mg/mL). The gel mixture was irradiated for 21 hours, obtaining **29%** product yield according to GC-FID analysis (22% isolated yield as white powder). Using 2-acetyl-4-chlorothiophene, the gel mixture was irradiated for 24 hours, obtaining **12%** product yield according to GC-FID analysis.

^{1}H NMR (400 MHz, $CDCl_3$) δ 8.32 (dd, *J* = 5.7, 1.3 Hz, 1H), 8.02 (dd, *J* = 8.2, 1.3 Hz, 1H), 4.25 – 4.10 (m, 4H), 2.55 (s, 3H), 1.35 (td, *J* = 7.1, 0.5 Hz, 6H) ppm.
^{13}C NMR (101 MHz, $CDCl_3$) δ 190.6 (C, s), 146.7 (C, d, *J* = 15.6 Hz), 141.8 (CH, d, *J* = 16.2 Hz), 134.0 (CH, d, *J* = 15.8 Hz), 131.4 (C, d, *J* = 197.0 Hz), 62.7 (CH_2, d, *J* = 5.6 Hz), 27.0 (CH_3, s), 16.5 (CH_3, d, *J* = 6.5 Hz) ppm.
^{31}P NMR (162 MHz, $CDCl_3$) δ 9.80 (s) ppm.
HRMS (EI): m/z $(M+H)^+$ = calcd. for $C_{10}H_{15}O_4PS$: 263.0501, found: 263.0497.

3m. Diethyl (2-acetylthiophen-3-yl)phosphonate

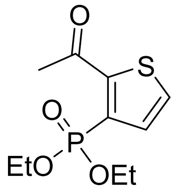

The compound was prepared according to the general procedure using 9-10-dicyanoanthracene (1.2 mg, 5 µmol, 0.1 equiv.), 2-acetyl-3-bromothiophene (10.6 mg, 50 µmol, 1.0 equiv.), triethylphosphite (45 µL, 250 µmol, 5 equiv.), dodecanenitrile (12 µL, 50 µmol, 1.0 equiv.) as internal standard and DIPEA (10.5 µL, 60 µmol, 1.2 equiv.) and G1 (10 mg/mL). The gel mixture was irradiated for 8 hours, obtaining **89%** product yield according to GC-FID analysis (70% isolated yield as yellow oil). Using 2-acetyl-3-chlorothiophene, the gel mixture was irradiated for 24 hours, obtaining **48%** product yield according to GC-FID analysis.

^{1}H NMR (400 MHz, $CDCl_3$) δ 7.54 (dd, *J* = 5.0, 2.8 Hz, 1H), 7.51 (t, *J* = 4.9 Hz, 1H), 4.32 – 4.19 (m, 4H), 2.71 (s, 3H), 1.36 (td, *J* = 7.1, 0.5 Hz, 6H) ppm.
^{13}C NMR (101 MHz, $CDCl_3$) δ 190.4 (C, d, *J* = 2.6 Hz), 149.1 (C, d, *J* = 15.9 Hz), 134.9 (CH, d, *J* = 14.2 Hz), 132.2 (C, d, *J* = 194.0 Hz), 130.31 (CH, d, *J* = 19.6 Hz), 63.02 (CH_2, d, *J* = 6.0 Hz), 29.4 (CH_3, d, *J* = 1.6 Hz),16.5 (CH_3, d, *J* = 6.5 Hz) ppm.
^{31}P NMR (162 MHz, $CDCl_3$) δ 7.14 (s) ppm.
HRMS (EI): m/z $(M+H)^+$ = calcd. for $C_{10}H_{15}O_4PS$: 263.0501, found: 263.0498.

3n. Diethyl (4-acetylthiophen-2-yl)phosphonate

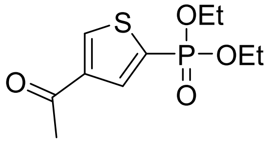

The compound was prepared according to the general procedure using 9-10-dicyanoanthracene (1.2 mg, 5 µmol, 0.1 equiv.), 3-acetyl-5-chlorothiophene (8.0 mg, 50 µmol, 1.0 equiv.), triethylphosphite (180 µL, 1000 µmol, 20 equiv.), dodecanenitrile (12 µL, 50 µmol, 1.0 equiv.) as internal standard and DIPEA (10.5 µL, 60 µmol, 1.2 equiv.) and G1 (10 mg/mL). The gel mixture was irradiated for 24 hours, obtaining **26%** product yield according to GC-FID analysis (19% isolated yield as white powder).

^{1}H NMR (400 MHz, $CDCl_3$) δ 8.17 (dd, J = 8.0, 1.2 Hz, 1H), 7.87 (dd, J = 4.5, 1.2 Hz, 1H), 4.21 – 4.06 (m, 4H), 2.58 (s, 3H), 1.35 (t, J = 7.2 Hz, 6H) ppm.
^{13}C NMR (101 MHz, $CDCl_3$) δ 191.7 (C, s), 143.4 (C, d, *J* = 17.4 Hz), 138.7 (CH, d, *J* = 6.8 Hz), 136.0 (CH, d, *J* = 12.0 Hz), 129.1 (C, d, *J* = 211.2 Hz), 63.2 (CH_2, d, *J* = 5.4 Hz), 27.9 (CH_3, s), 16.4 (CH_3, d, *J* = 6.6 Hz) ppm.
^{31}P NMR (162 MHz, $CDCl_3$) δ 10.98 (s) ppm.
HRMS (EI): m/z $(M+H)^+$ = calcd. for $C_{10}H_{15}O_4PS$: 263.0501, found: 263.0502

3o. Diethyl (R)-(5-(((2-oxo-3-(4-(3-oxomorpholino)phenyl)oxazolidin-5-yl)methyl)carbamoyl)thiophen-2-yl)phosphonate

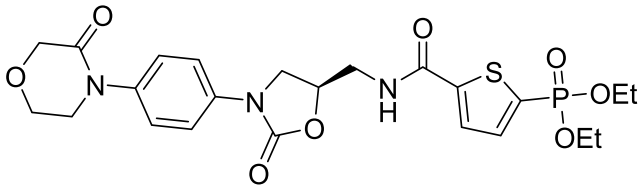

The compound was prepared according to the general procedure using 9-10-dicyanoanthracene (2.4 mg, 10 μmol, 0.2 equiv.), rivaroxaban (21.8 mg, 50 μmol, 1.0 equiv.), triethylphosphite (180 μL, 1000 μmol, 20 equiv.), DIPEA (10.5 μL, 60 μmol, 1.2 equiv.) and G1 (10 mg/mL). The gel mixture was irradiated for 24 hours, obtaining **70%** (18.9 mg) isolated product yield as pale-yellow oil.

^{1}H NMR (400 MHz, $CDCl_3$) δ 7.72 (t, *J* = 6.0 Hz, 1H), 7.58 (t, *J* = 3.5 Hz, 1H), 7.55 – 7.50 (m, 2H), 7.48 (dd, *J* = 8.1, 3.8 Hz, 1H), 7.33 – 7.28 (m, 2H), 4.83 (td, *J* = 11.5, 5.1 Hz, 1H), 4.32 (s, 2H), 4.23 – 4.06 (m, 4H), 4.06 – 3.98 (m, 3H), 3.83 (dd, *J* = 9.2, 6.6 Hz, 1H), 3.79 – 3.66 (m, 4H), 1.38 – 1.25 (m, 6H) ppm.
^{13}C NMR (101 MHz, $CDCl_3$) δ 167.2 (C, s), 162.1 (C, d, *J* = 2.2 Hz), 154.6 (C, s), 145.8 (C, d, *J* = 8.0 Hz), 137.4 (C, s), 136.9 (C, s),136.6 (CH, d, *J* = 11.4 Hz), 132.9 (C, d, *J* = 205.3 Hz), 129.0 (CH, d, *J* = 17.2 Hz), 126.5 (CH, s), 119.2 (CH, s), 71.7 (CH, s), 68.6 (CH_2, s), 64.2 (CH_2, s), 63.3 (CH_2, dd, *J* = 5.4, 2.4 Hz), 49.9 (CH_2, s), 47.8 (CH_2, s), 42.6 (CH_2, s), 16.4 (CH_3, d, *J* = 6.7 Hz) ppm.
^{31}P NMR (162 MHz, $CDCl_3$) δ 9.80 (s) ppm.
HRMS (EI): m/z $(M+H)^+$ = calcd. for $C_{23}H_{28}N_3O_8PS$: 538.1374, found: 538.1375.

FURANS

10a. Diethyl (5-cyanofuran-2-yl)phosphonate

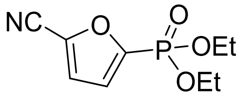

The compound was prepared according to the general procedure using 9-10-dicyanoanthracene (1.2 mg, 5 μmol, 0.1 equiv.), 5-bromo-2-furancarbonitrile (5 μL, 50 μmol, 1.0 equiv.), triethylphosphite (45 μL, 250 μmol, 5 equiv.), dodecanenitrile (12 μL, 50 μmol, 1.0 equiv.) as internal standard, DIPEA (10.5 μL, 60 μmol, 1.2 equiv.) and G1 (10 mg/mL). The gel mixture was irradiated for 3 hours, obtaining **85%** product yield according to GC-FID analysis (77% isolated yield as yellow oil).

^{1}H NMR (400 MHz, $CDCl_3$) δ 7.17 (dd, *J* = 3.6, 2.0 Hz, 1H), 7.14 (dd, *J* = 3.6, 2.2 Hz, 1H), 4.32 – 4.10 (m, 4H), 1.37 (td, *J* = 7.1, 0.5 Hz, 6H) ppm.
^{13}C NMR (101 MHz, $CDCl_3$) δ 150.4 (C, d, *J* = 232.0 Hz), 130.4 (C, d, *J* = 13.4 Hz), 122.4 (d, *J* = 23.2 Hz), 121.8 (d, *J* = 10.4 Hz), 110.5 (C, d, *J* = 2.5 Hz), 63.9 (d, *J* = 5.7 Hz), 16.4 (d, *J* = 6.3 Hz) ppm.
^{31}P NMR (162 MHz, $CDCl_3$) δ 0.19 (s) ppm.
HRMS (EI): m/z $(M+H)^+$ = calcd. for $C_9H_{12}NO_4P$: 230.0577, found: 230.0574.

10b. Diethyl (5-acetylfuran-2-yl)phosphonate

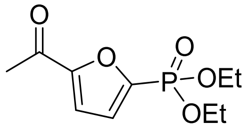

The compound (CAS: 261365-06-4) was prepared according to the general procedure using 9-10-dicyanoanthracene (1.2 mg, 5 μmol, 0.1 equiv.), 2-acetyl-5-bromofuran (9.9 mg, 50 μmol, 1.0 equiv.), triethylphosphite (45 μL, 250 μmol, 5 equiv.), dodecanenitrile (12 μL, 50 μmol, 1.0 equiv.) as internal standard, DIPEA (10.5 μL, 60 μmol, 1.2 equiv.) and G1 (10 mg/mL). The gel mixture was irradiated for 4 hours, obtaining **96%** product yield according to GC-FID analysis (82% isolated yield as pale-yellow oil).

^{1}H NMR (400 MHz, $CDCl_3$) δ 7.16 (dd, *J* = 3.6, 2.4 Hz, 1H), 7.14 (dd, *J* = 3.6, 1.8 Hz, 1H), 4.26 – 4.10 (m, 4H), 2.51 (s, 3H), 1.34 (q, *J* = 6.9 Hz, 6H) ppm.
^{13}C NMR (101 MHz, $CDCl_3$) δ 187.3 (C, d, *J* = 1.0 Hz), 156.4 (C, d, *J* = 9.5 Hz), 147.9 (C, d, *J* = 236.5 Hz), 123.3 (CH, d, *J* = 24.0 Hz), 116.0 (CH, d, *J* = 10.7 Hz), 63.5 (CH_2, d, *J* = 5.6 Hz), 26.5 (CH_3, d, *J* = 1.6 Hz), 16.4 (CH_3, d, *J* = 6.2 Hz) ppm.
^{31}P NMR (162 MHz, $CDCl_3$) δ 2.15 (s) ppm.
HRMS (EI): m/z $(M+H)^+$ = calcd. for $C_{10}H_{15}O_5P$: 247.0730, found: 247.0729.
Spectral data were consistent with the literature.[1]

10c. Diethyl (5-methylfuran-2-yl)phosphonate

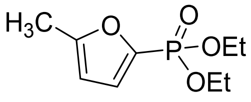

The compound (CAS: 70519-53-8) was prepared according to the general procedure using 9-10-dicyanoanthracene (2.4 mg, 10 µmol, 0.2 equiv.), 2-bromo-5-methylfuran (5.5 µL, 50 µmol, 1.0 equiv.), triethylphosphite (180 µL, 1000 µmol, 20 equiv.), dodecanenitrile (12 µL, 50 µmol, 1.0 equiv.) as internal standard, DIPEA (10.5 µL, 60 µmol, 1.2 equiv.) and G1 (10 mg/mL). The gel mixture was irradiated for 24 hours, obtaining **30%** product yield according to GC-FID analysis (19% isolated yield as white powder).

^{1}H NMR (400 MHz, $CDCl_3$) δ 7.05 (dd, *J* = 2.9, 2.2 Hz, 1H), 6.08 (ddd, *J* = 3.4, 2.7, 0.9 Hz, 1H), 4.26 – 4.16 (m, 4H), 2.36 (s, 3H), 1.34 (td, *J* = 7.1, 0.5 Hz, 6H) ppm.
^{13}C NMR (101 MHz, $CDCl_3$) δ 161.8 (C, d, *J* = 242.8 Hz), 143.2 (C, s), 124.4 (d, *J* = 26.2 Hz), 107.2 (CH, d, *J* = 11.8 Hz), 62.8 (CH_2, d, *J* = 3.0 Hz), 16.4 (CH_3, d, *J* = 7.04 Hz), 14.2 (CH_3, s) ppm.
^{31}P NMR (162 MHz, $CDCl_3$) δ 5.02 (s) ppm.
HRMS (EI): m/z $(M+H)^+$ = calcd. for $C_9H_{15}O_4P$: 219.0781, found 219.0779.

PYRROLE

11a. Diethyl (5-acetyl-1-methyl-1H-pyrrol-2-yl)phosphonate

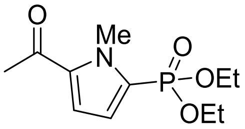

The compound (CAS: 1394991-86-6) was prepared according to the general procedure using 9-10-dicyanoanthracene (1.2 mg, 5 µmol, 0.1 equiv.), 2-acetyl-5-bromo-*N*-methylpyrrole (10.1 mg, 50 µmol, 1.0 equiv.), triethylphosphite (45 µL, 250 µmol, 5 equiv.), dodecanenitrile (12 µL, 50 µmol, 1.0 equiv.) as internal standard, DIPEA (10.5 µL, 60 µmol, 1.2 equiv.) and G1 (10 mg/mL). The gel mixture was irradiated for 4 hours, obtaining **92%** product yield according to GC-FID analysis (76% isolated yield as pale-yellow oil).

^{1}H NMR (400 MHz, $CDCl_3$) δ 6.91 (t, *J* = 4.1 Hz, 1H), 6.76 (dd, *J* = 4.1, 3.2 Hz, 1H), 4.21 – 4.10 (m, 4H), 4.09 (d, *J* = 0.8 Hz, 3H), 2.48 (s, 3H), 1.34 (t, *J* = 7.1 Hz, 6H) ppm.
^{13}C NMR (101 MHz, $CDCl_3$) δ 190.0 (C, d, *J* = 1.3 Hz), 135.7 (C, d, *J* = 11.5 Hz), 128.2 (C, d, *J* = 219.2 Hz), 119.6 (CH, d, *J* = 17.0 Hz), 118.1 (CH, d, *J* = 13.9 Hz), 62.8 (CH_2, dd, *J* = 5.3, 2.1 Hz), 36.1 – 36.0 (CH_3, m), 28.6 – 28.1 (CH_3, m), 16.4 (CH_3, d, *J* = 6.5 Hz) ppm.
^{31}P NMR (162 MHz, $CDCl_3$) δ 8.33 (s) ppm.
HRMS (EI): m/z $(M+H)^+$ = calcd. for $C_{11}H_{18}NO_4P$: 260.1046, found: 260.1046.

[1] Qun Dang, Srinivas Rao Kasibhatla, K. Raja Reddy, Tao Jiang, M. Rami Reddy, Scott C. Potter, James M. Fujitaki, Paul D. van Poelje, Jingwei Huang, William N. Lipscomb, and Mark D. Erion. *J. Am. Chem. Soc.* **2007**, 129, *50*, 15491–15502

Spectral data were consistent with the literature.[2]

SELENOPHENE

12a. Diethyl (5-acetylselenophen-2-yl)phosphonate

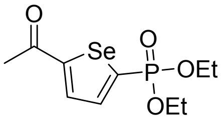

The compound was prepared according to the general procedure using 9-10-dicyanoanthracene (1.2 mg, 5 µmol, 0.1 equiv.), 2-acetyl-5-bromoselenophene (12.6 mg, 50 µmol, 1.0 equiv.), triethylphosphite (45 µL, 250 µmol, 5 equiv.), dodecanenitrile (12 µL, 50 µmol, 1.0 equiv.) as internal standard, DIPEA (10.5 µL, 60 µmol, 1.2 equiv.) and G1 (10 mg/mL). The gel mixture was irradiated for 4 hours, obtaining **86%** product yield according to GC-FID analysis (71% isolated yield as yellow oil).

^{1}H NMR (400 MHz, $CDCl_3$) δ 7.92 (dd, *J* = 8.4, 3.0 Hz, 1H), 7.89 (t, *J* = 2.9 Hz, 1H), 4.24 – 4.08 (m, 4H), 2.59 (s, 3H), 1.34 (td, *J* = 7.1, 0.5 Hz, 6H) ppm.
^{13}C NMR (101 MHz, $CDCl_3$) δ 192.0 (C, d, *J* = 1.2 Hz), 158.4 (C, d, *J* = 5.5 Hz), 142.4 (C, d, *J* = 197.7 Hz), 139.2 (CH, d, *J* = 10.5 Hz), 134.5 (CH, d, *J* = 18.7 Hz), 63.2 (CH_2, dd, *J* = 5.1, 2.2 Hz), 26.5 (CH_3, d, *J* = 2.1 Hz), 16.4 (CH_3, d, *J* = 6.6 Hz) ppm.
^{31}P NMR (162 MHz, $CDCl_3$) δ 12.02 (s) ppm.
HRMS (EI): m/z $(M+H)^+$ = calcd. for $C_{10}H_{15}O_4PSe$: 310.9946, found: 310.9944.

OXAZOLE

13a. Ethyl 2-(diethoxyphosphoryl)oxazole-5-carboxylate

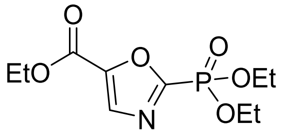

The compound was prepared according to the general procedure using 9-10-dicyanoanthracene (1.2 mg, 5 µmol, 0.1 equiv.), ethyl 2-chloro-1,3-oxazole-5-carboxylate (6.6 µL, 50 µmol, 1.0 equiv.), triethylphosphite (45 µL, 250 µmol, 5 equiv.), dodecanenitrile (12 µL, 50 µmol, 1.0 equiv.) as internal standard, DIPEA (10.5 µL, 60 µmol, 1.2 equiv.) and G1 (10 mg/mL). The gel mixture was irradiated for 15 hours, obtaining **91%** product yield according to GC-FID analysis (76% isolated yield as pale-yellow oil).

^{1}H NMR (400 MHz, $CDCl_3$) δ 7.84 (d, *J* = 1.2 Hz, 1H), 4.41 (q, *J* = 7.1 Hz, 2H), 4.38 – 4.30 (m, 4H), 1.42 (td, *J* = 7.1, 0.7 Hz, 6H), 1.40 (t, *J* = 7.1 Hz, 3H) ppm.
^{13}C NMR (101 MHz, $CDCl_3$) δ 158.4 (C, d, *J* = 266.2 Hz), 157.2 (C, d, *J* = 0.5 Hz), 145.0 (C, d, *J* = 5.6 Hz), 133.9 (CH, d, *J* = 14.6 Hz), 64.9 (CH_2, d, *J* = 5.9 Hz), 62.2 (CH_2, s), 16.4 (CH_3, d, *J* = 6.3 Hz), 14.3 (CH_3, s) ppm.
^{31}P NMR (162 MHz, $CDCl_3$) δ -4.19 (s) ppm.
HRMS (EI): m/z $(M+H)^+$ = calcd. for $C_{10}H_{16}NO_6P$: 278.0788, found: 278.0787.

THIAZOLE

14a. Diethyl (5-cyanothiazol-2-yl)phosphonate

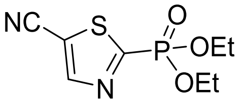

The compound was prepared according to the general procedure using 9-10-dicyanoanthracene (1.2 mg, 5 µmol, 0.1 equiv.), ethyl 2-bromo-1,3-thiazole-5-carbonitrile (9.5 mg, 50 µmol, 1.0 equiv.), triethylphosphite (45 µL, 250 µmol, 5 equiv.), dodecanenitrile (12 µL, 50 µmol, 1.0 equiv.) as internal standard, DIPEA (10.5 µL, 60 µmol, 1.2 equiv.) and

[2] Chang-Bing Xiang, Yong-Jun Bian, Xue-Rong Mao, and Zhi-Zhen Huang. *J. Org. Chem.* **2012**, 77, *17*, 7706–7710

G1 (10 mg/mL). The reaction mixture was irradiated for 15 hours, obtaining **80%** product yield according to GC-FID analysis (72% isolated yield as pale-yellow oil).

^{1}H NMR (400 MHz, $CDCl_3$) δ 8.49 (d, J = 1.5 Hz, 1H), 4.39 – 4.22 (m, 4H), 1.39 (td, J = 7.1, 0.7 Hz, 6H) ppm.
^{13}C NMR (101 MHz, $CDCl_3$) δ 165.6 (C, d, *J* = 237.3 Hz), 153.8 (CH, d, *J* = 26.0 Hz), 111.5 (C, d, *J* = 1.7 Hz), 111.0 (C, d, *J* = 1.5 Hz), 64.9 (CH_2, d, *J* = 6.1 Hz), 16.4 (CH_3, d, *J* = 6.1 Hz) ppm.
^{31}P NMR (162 MHz, $CDCl_3$) δ 0.51 (s) ppm.
HRMS (EI): m/z $(M+H)^+$ = calcd. for $C_8H_{11}N_2O_3PS$: 247.0301, found: 247.0300.

<u>6-MEMBERS HETEROARENES</u>

15a. Diethyl (4-cyanophenyl)phosphonate

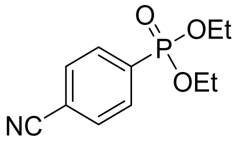

The compound (CAS: 28255-72-3) was prepared according to the general procedure using 9-10-dicyanoanthracene (1.2 mg, 5 μmol, 0.1 equiv.), 4-bromobenzonitrile (9.2 mg, 50 μmol, 1.0 equiv.), triethylphosphite (45 μL, 250 μmol, 5 equiv.), dodecanenitrile (12 μL, 50 μmol, 1.0 equiv.) as internal standard, DIPEA (10.5 μL, 60 μmol, 1.2 equiv.) and G1 (10 mg/mL). The reaction mixture was irradiated for 2 hours, obtaining **98%** product yield according to GC-FID analysis (90% isolated yield as colorless oil).

^{1}H NMR (400 MHz, $CDCl_3$) δ 7.92 (dd, J = 13.1, 8.5 Hz, 1H), 7.75 (dd, J = 8.5, 3.6 Hz, 1H), 4.25 – 4.01 (m, 4H), 1.34 (t, J = 7.1 Hz, 6H) ppm.
^{13}C NMR (101 MHz, $CDCl_3$) δ 134.1 (C, d, J = 187.8 Hz), 132.4 (CH, d, J = 9.8 Hz), 132.1 (CH, d, J = 15.0 Hz), 118.0 (C, s), 116.2 (C, d, J = 3.6 Hz), 62.8 (CH_2, d, J = 5.6 Hz), 16.5 (CH_3, d, J = 6.3 Hz) ppm.
Spectral data were consistent with the literature.[3]

15b. Diethyl (2-cyanophenyl)phosphonate

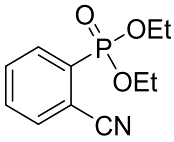

The compound (CAS: 34595-07-8) was prepared according to the general procedure using 9-10-dicyanoanthracene (1.2 mg, 5 μmol, 0.1 equiv.), 2-bromobenzonitrile (9.2 mg, 50 μmol, 1.0 equiv.), triethylphosphite (45 μL, 250 μmol, 5 equiv.), dodecanenitrile (12 μL, 50 μmol, 1.0 equiv.) as internal standard, DIPEA (10.5 μL, 60 μmol, 1.2 equiv.) and G1 (10 mg/mL). The reaction mixture was irradiated for 2 hours, obtaining **92%** product yield according to GC-FID analysis (83% isolated yield as colorless oil).

^{1}H NMR (400 MHz, $CDCl_3$) δ 8.16 – 8.07 (m, 1H), 7.83 – 7.77 (m, 1H), 7.74 – 7.61 (m, 2H), 4.39 – 4.03 (m, 4H), 1.48 – 1.31 (m, 6H) ppm.
^{13}C NMR (101 MHz, $CDCl_3$) δ 134.7 (CH, d, J = 6.6 Hz), 134.6 (CH, d, J = 9.2 Hz), 132.5 (CH, d, J = 2.6 Hz), 132.5 (C, d, J = 188.0 Hz), 132.3 (CH, d, J = 14.0 Hz), 117.2 (C, d, J = 5.7 Hz), 114.8 (C, d, J = 5.0 Hz), 63.3 (CH_2, d, J = 6.0 Hz), 16.4 (CH_3, d, J = 6.3 Hz) ppm.
Spectral data were consistent with the literature.[4]

---

[3] Rongqiang Zhuang, Jian Xu, Zhenshi Cai, Guo Tang, Meijuan Fang, and Yufen Zhao. *Org. Lett.* **2011**, 13, *8*, 2110–2113.
[4] I. Ghosh, R. S. Shaikh, B. König. *Angew. Chem. Int. Ed.* **2017**, *56*, 8544.
[5] Ya Bai, Nian Liu, Shutao Wang, Siyu Wang, Shulin Ning, Lingling Shi, Lili Cui, Zhuoqi Zhang, and Jinbao Xiang. *Organic Letters* **2019** *21* (17), 6835-6838

15c. diethyl quinolin-2-ylphosphonate

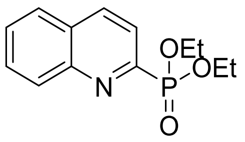

The compound (CAS: 37175-36-3) was prepared according to the general procedure using 9-10-dicyanoanthracene (2.4 mg, 10 μmol, 0.2 equiv.), 2-bromoquinoline (10.4 mg, 50 μmol, 1.0 equiv.), triethylphosphite (180 μL, 1000 μmol, 20 equiv.), dodecanenitrile (12 μL, 50 μmol, 1.0 equiv.) as internal standard, DIPEA (10.5 μL, 60 μmol, 1.2 equiv.) and G1 (10 mg/mL). The reaction mixture was irradiated for 10 hours, obtaining **89%** product yield according to GC-FID analysis (74% isolated yield as colorless oil).

^{1}H NMR (400 MHz, $CDCl_3$) δ 8.27 (dd, J = 7.9, 6.3 Hz, 2H), 8.00 (dd, J = 8.4, 4.7 Hz, 1H), 7.87 (dd, J = 8.2, 1.1 Hz, 1H), 7.78 (ddd, J = 8.5, 6.9, 1.4 Hz, 1H), 7.64 (dd, J = 11.1, 4.1 Hz, 1H), 4.39 – 4.22 (m, 4H), 1.38 (t, J = 7.1 Hz, 6H) ppm.
Spectral data were consistent with the literature.[5]

15d. Diethyl quinolin-3-ylphosphonate

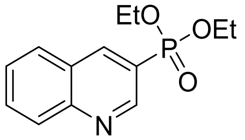

The compound (CAS: 82594-50-1) was prepared according to the general procedure using 9-10-dicyanoanthracene (2.4 mg, 10 μmol, 0.2 equiv.), 3-bromoquinoline (6.9 μL, 50 μmol, 1.0 equiv.), triethylphosphite (180 μL, 1000 μmol, 20 equiv.), dodecanenitrile (12 μL, 50 μmol, 1.0 equiv.) as internal standard, DIPEA (10.5 μL, 60 μmol, 1.2 equiv.) and G1 (10 mg/mL). The reaction mixture was irradiated for 10 hours, obtaining **83%** product yield according to GC-FID analysis (69% isolated yield as pale-yellow oil).

^{1}H NMR (400 MHz, $CDCl_3$) δ 9.15 (dd, J = 4.5, 2.0 Hz, 1H), 8.71 (dd, J = 15.2, 1.3 Hz, 1H), 8.16 (d, J = 8.0 Hz, 1H), 7.92 (dd, *J* = 8.2, 0.9 Hz, 1H), 7.85 (ddd, J = 8.4, 7.0, 1.3 Hz, 1H), 7.64 (ddd, J = 8.1, 7.0, 1.0 Hz, 1H), 4.37 – 4.06 (m, 4H), 1.36 (td, J = 7.1, 0.4 Hz, 6H) ppm.
^{13}C NMR (101 MHz, $CDCl_3$) δ 150.7 (CH, d, J = 12.2 Hz), 149.5 (C, d, J = 1.2 Hz), 142.2 (CH, d, J = 8.6 Hz), 132.0 (CH, s), 129.7 (CH, d, J = 1.1 Hz), 128.8 (CH, s), 127.8 (CH, d, J = 1.1 Hz), 126.9 (C, d, J = 13.5 Hz), 122.1 (C, d, J = 189.3 Hz), 62.8 (CH_2, d, J = 5.5 Hz), 16.5 (CH_3, d, J = 6.4 Hz) ppm.
Spectral data were consistent with the literature.[4]

15e. Tert-butyl 2-(diethoxyphosphoryl)-3-methyl-1H-indole-1-carboxylate

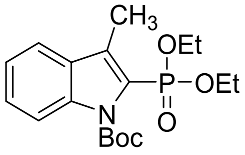

The compound (CAS: 2035049-93-3) was prepared according to the general procedure using 9-10-dicyanoanthracene (1.2 mg, 5 μmol, 0.1 equiv.), *N*-Boc-2-bromo-3-methylindole (16.0 mg, 50 μmol, 1.0 equiv.), triethylphosphite (45 μL, 250 μmol, 5 equiv.), dodecanenitrile (12 μL, 50 μmol, 1.0 equiv.) as internal standard, DIPEA (10.5 μL, 60 μmol, 1.2 equiv.) and G1 (10 mg/mL). The reaction mixture was irradiated for 16 hours, obtaining **81%** product yield according to GC-FID analysis (65% isolated yield as yellow oil).

^{1}H NMR (400 MHz, $CDCl_3$) δ 8.07 (d, J = 8.5 Hz, 1H), 7.61 (d, J = 7.8 Hz, 1H), 7.42 (dd, J = 8.2, 7.4 Hz, 1H), 7.30 – 7.25 (m, 1H), 4.32 – 4.01 (m, 4H), 2.60 (d, J = 2.2 Hz, 3H), 1.69 (s, 9H), 1.38 – 1.33 (m, 6H) ppm.
^{13}C NMR (101 MHz, $CDCl_3$) δ 149.9 (C, s), 138.1 (C, d, J = 8.8 Hz), 132.1 (C, d, J = 16.9 Hz), 130.0 (C, d, J = 17.0 Hz), 127.3 (CH, s), 122.8 (CH, s), 122.3 (C, d, J = 220.5 Hz),

120.2 (CH, s), 115.4 (CH, s), 84.9 (C, s), 62.5 (CH_2, d, J = 5.5 Hz), 28.2 (CH_3, s), 16.5 (CH_3, d, J = 6.8 Hz), 11.1 (CH_3, s) ppm.
Spectral data were consistent with the literature.[6]

RADICAL TRAPPING

Tert-butyl 2-(diethoxyphosphoryl)-3-methyl-1H-indole-1-carboxylate

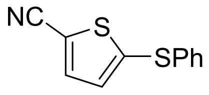

The compound (CAS: 1300106-13-1) was prepared according to the general procedure using 9-10-dicyanoanthracene (1.2 mg, 5 µmol, 0.1 equiv.), *N*-Boc-2-bromo-3-methylindole (16.0 mg, 50 µmol, 1.0 equiv.), triethylphosphite (45 µL, 250 µmol, 5 equiv.), diphenyl disulfide (47.5 mg, 250 µmol, 5 equiv.) as trapping agent, dodecanenitrile (12 µL, 50 µmol, 1.0 equiv.) as internal standard, DIPEA (10.5 µL, 60 µmol, 1.2 equiv.) and G1 (10 mg/mL). The reaction mixture was irradiated for 2.5 hours, obtaining **56%** product yield according to GC-FID analysis (41% isolated yield as colorless oil).

^{1}H NMR (400 MHz, $CDCl_3$) δ 7.50 (d, J = 3.9 Hz, 1H), 7.41 – 7.31 (m, 5H), 7.10 (d, J = 3.9 Hz, 1H) ppm.
^{13}C NMR (101 MHz, $CDCl_3$) δ 144.4 (C, s), 137.9 (CH, s), 134.8 (C, s), 131.9 (CH, s), 130.8 (CH, s), 129.8 (CH, s), 128.5 (CH, s), 113.7 (C, s), 112.1 (C, s) ppm.
HRMS (EI): m/z $(M+H)^+$ = calcd. for $C_{11}H_7NS_2$: 218.0093, found: 218.0089.
Spectral data were consistent with the literature.[7]

---

[4] I. Ghosh, R. S. Shaikh, B. König. *Angew. Chem. Int. Ed.* **2017**, *56*, 8544.
[6] Shaikh, Rizwan S.; Duesel, Simon J. S.; Koenig, Burkhard. *ACS Catalysis* **2016**, *6*(12), 8410-8414.
[7] Mieko Arisawaa, Fumihiko Toriyama, Masahiko Yamaguchi. *Tetrahedron Letters* 52, *18*, 2344-2347.

NMR Spectra

^{1}H 400MHz, $CDCl_3$

3a

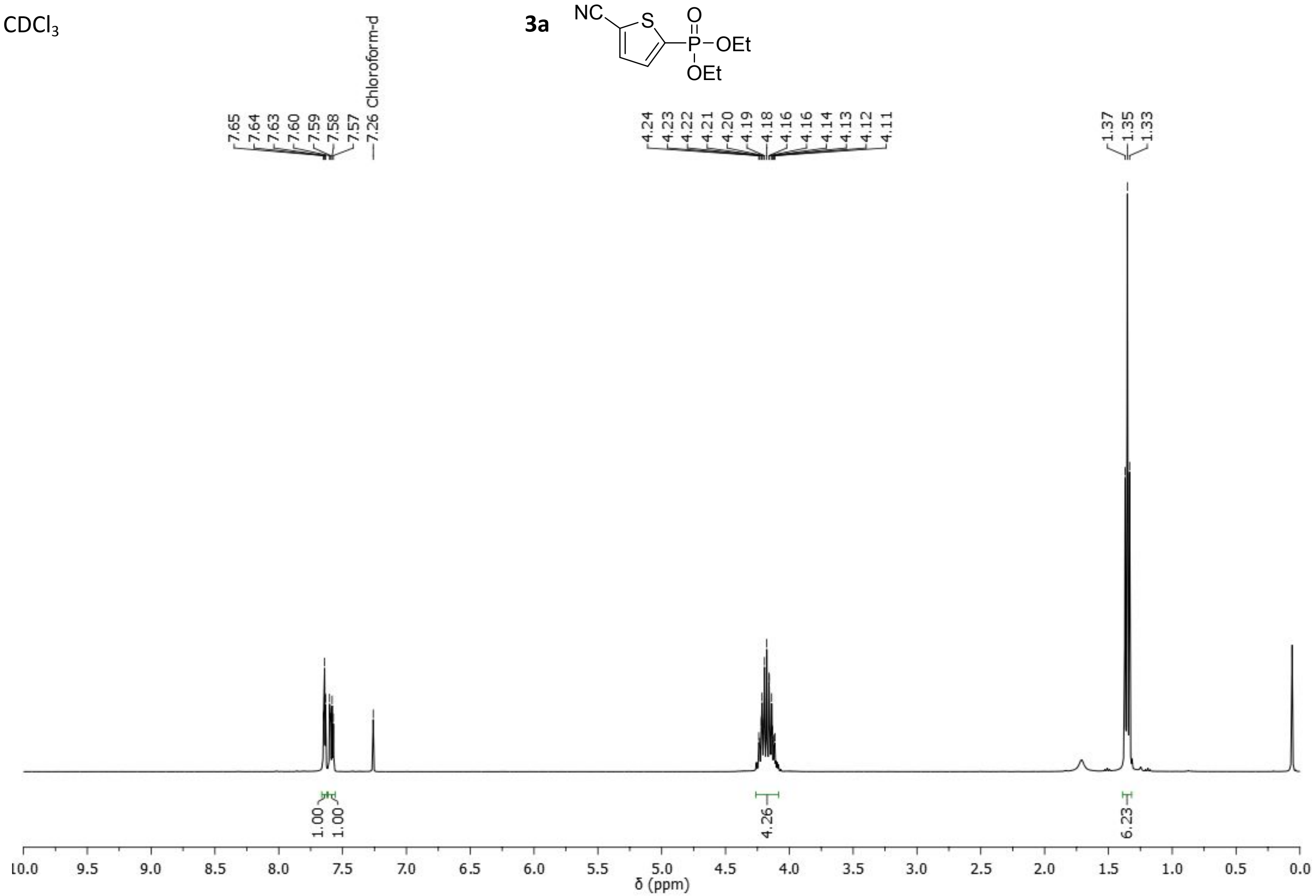

Figure S15. ^{1}H NMR spectrum of **3a**.

^{13}C 101MHz, $CDCl_3$

3a 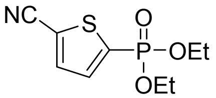

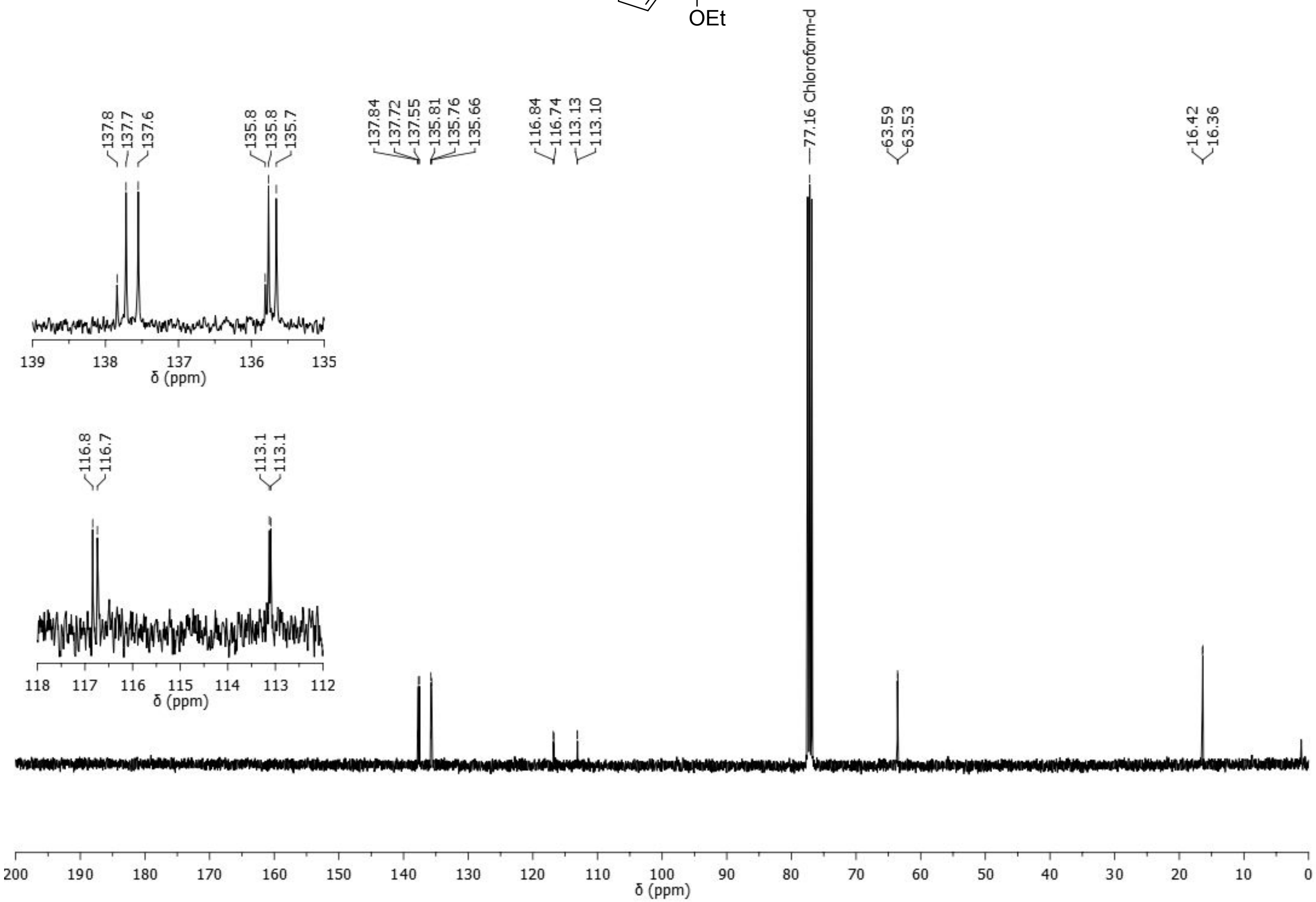

Figure S16. ^{13}C NMR spectrum of **3a**.

^{31}P 162MHz, $CDCl_3$

3a

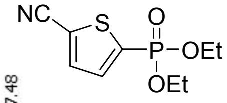

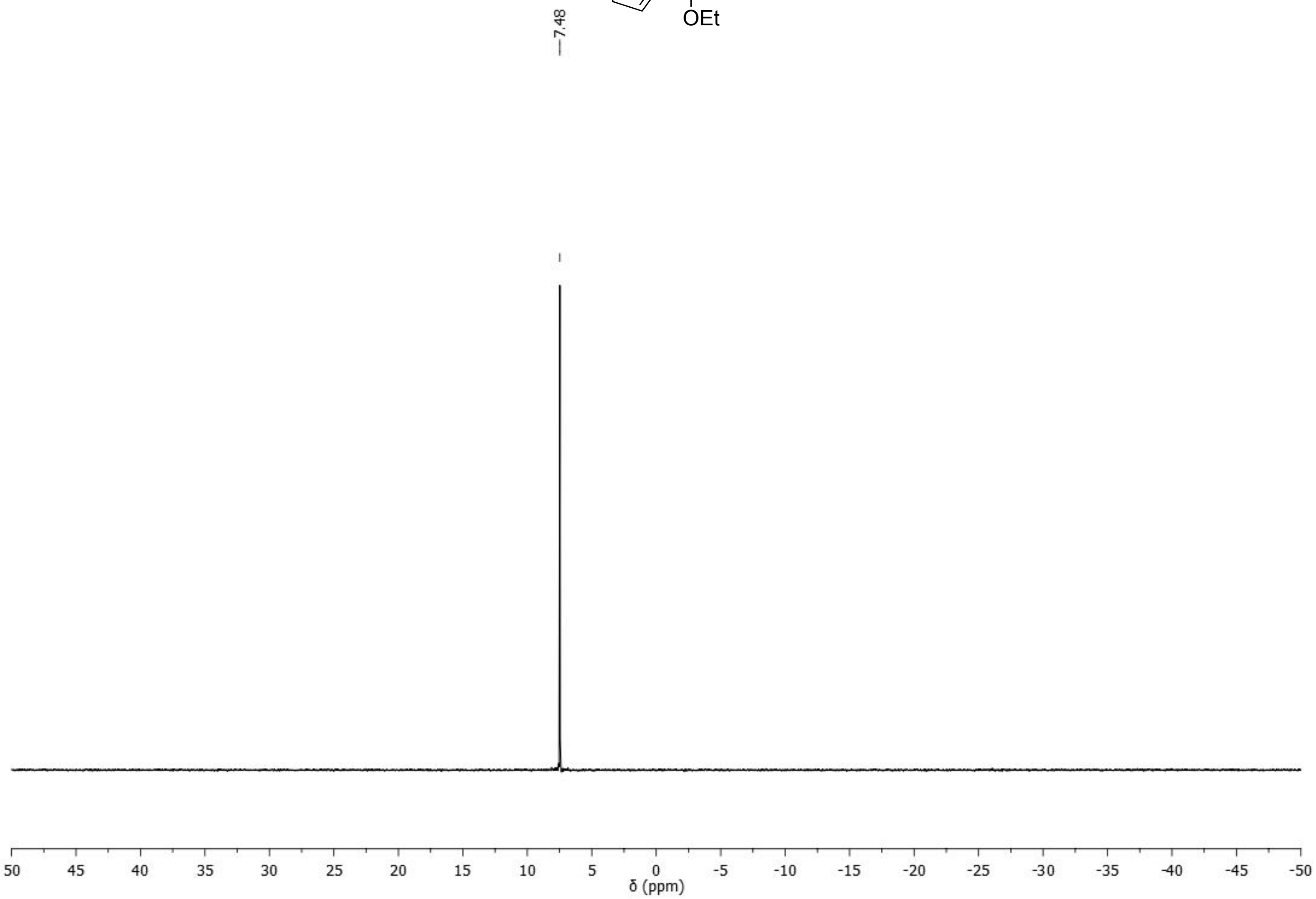

Figure S17. ^{31}P NMR spectrum of **3a**.

^1H 400MHz, $CDCl_3$

3b

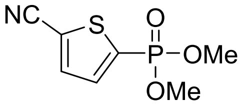

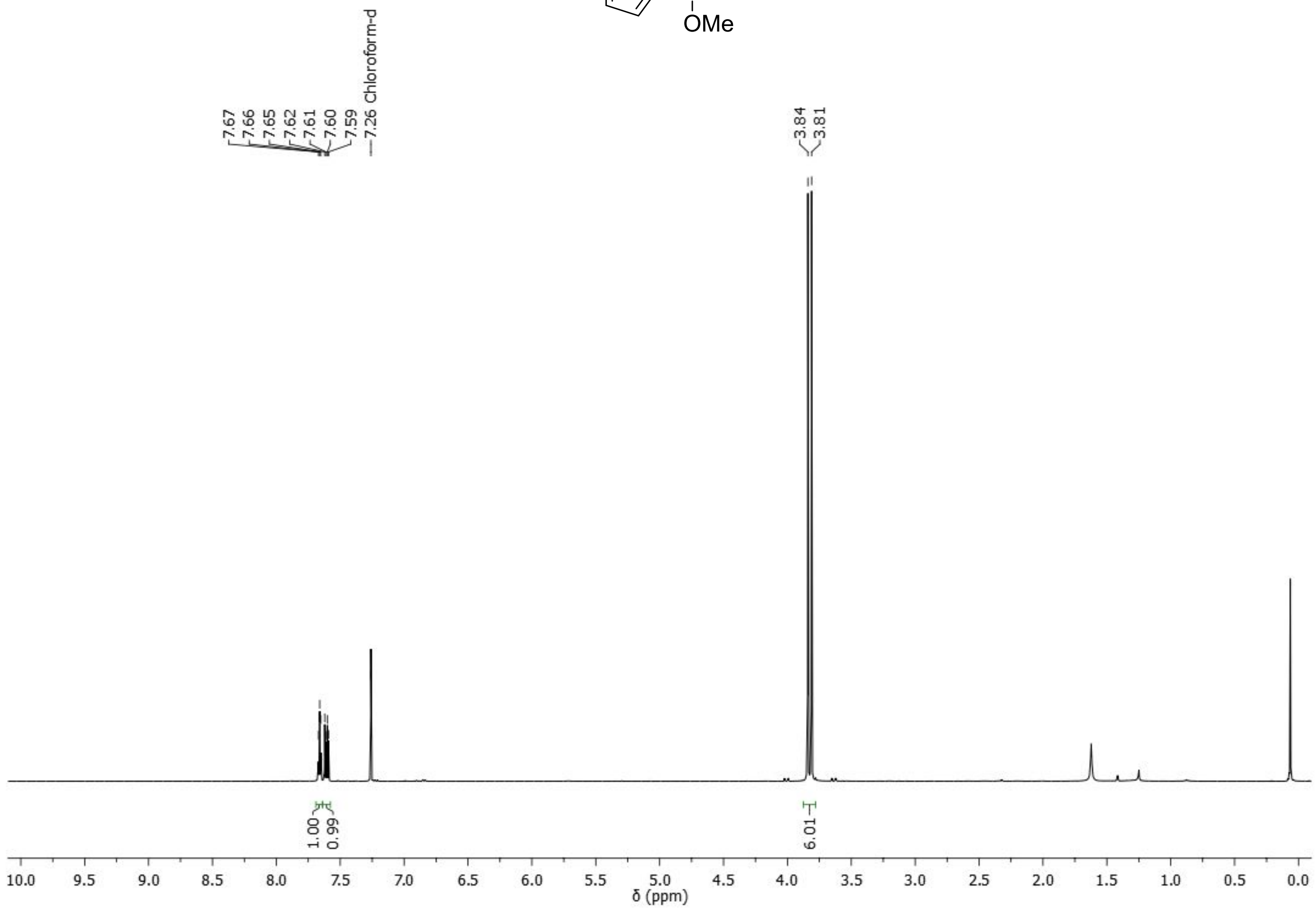

Figure S18. ^1H NMR spectrum of **3b**.

^{13}C 101MHz, $CDCl_3$

3b

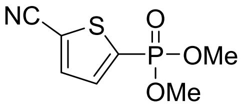

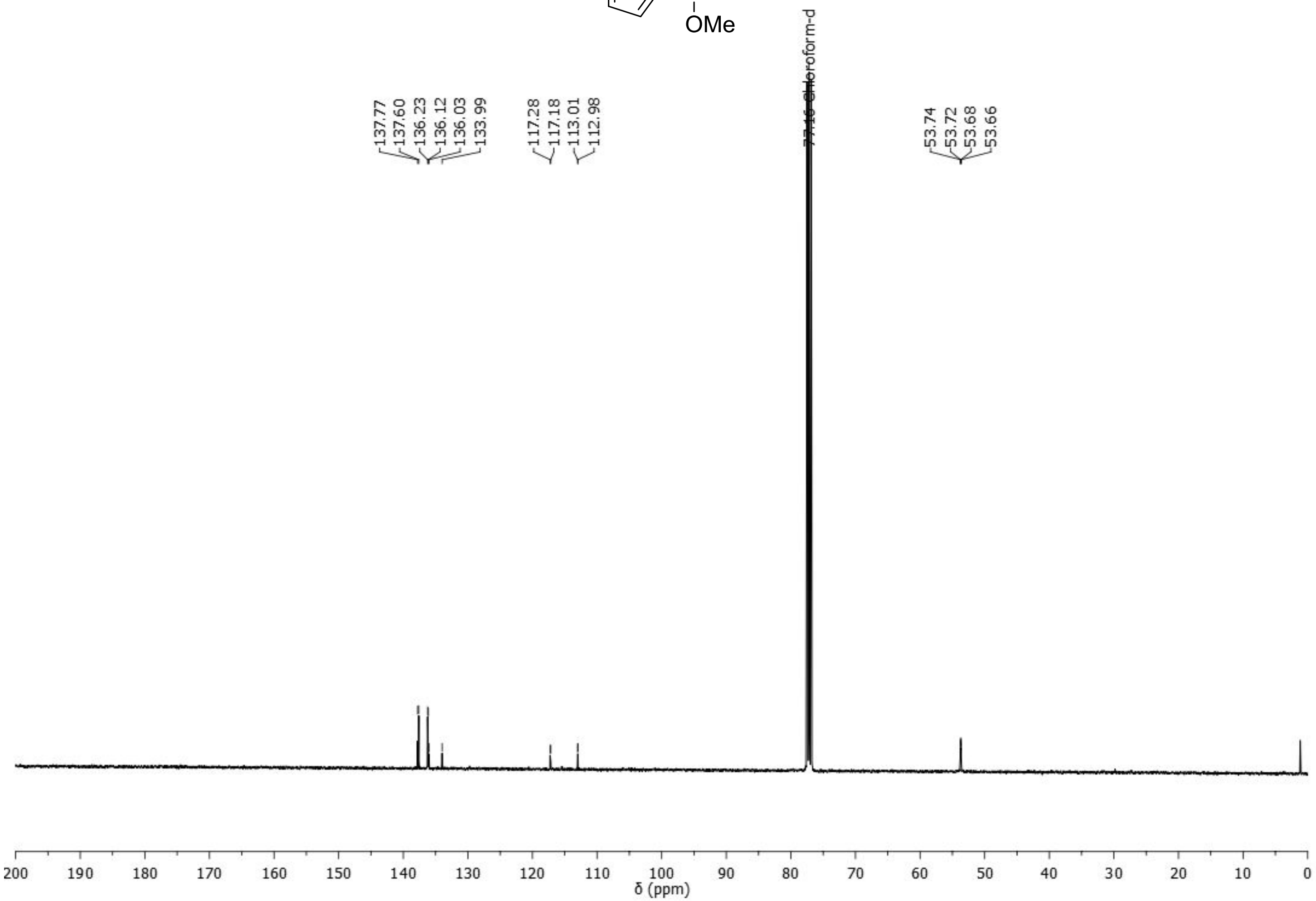

Figure S19. ^{13}C NMR spectrum of **3b**.

^{31}P 162MHz, $CDCl_3$

3b

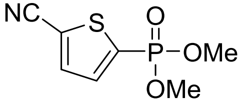

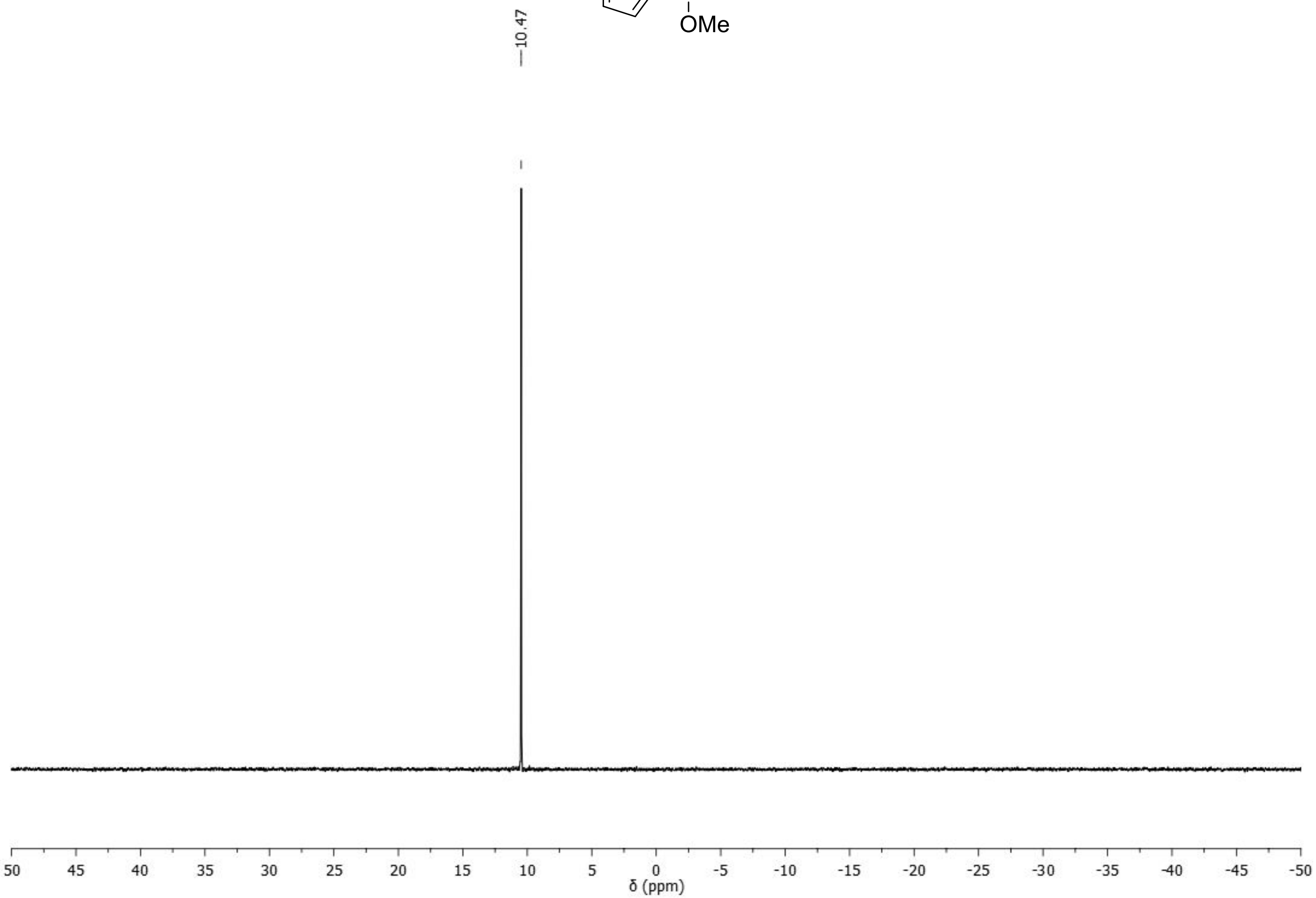

Figure S20. ^{31}P NMR spectrum of **3b**.

^{1}H 400MHz, $CDCl_3$

3c 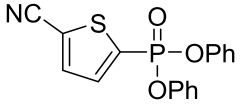

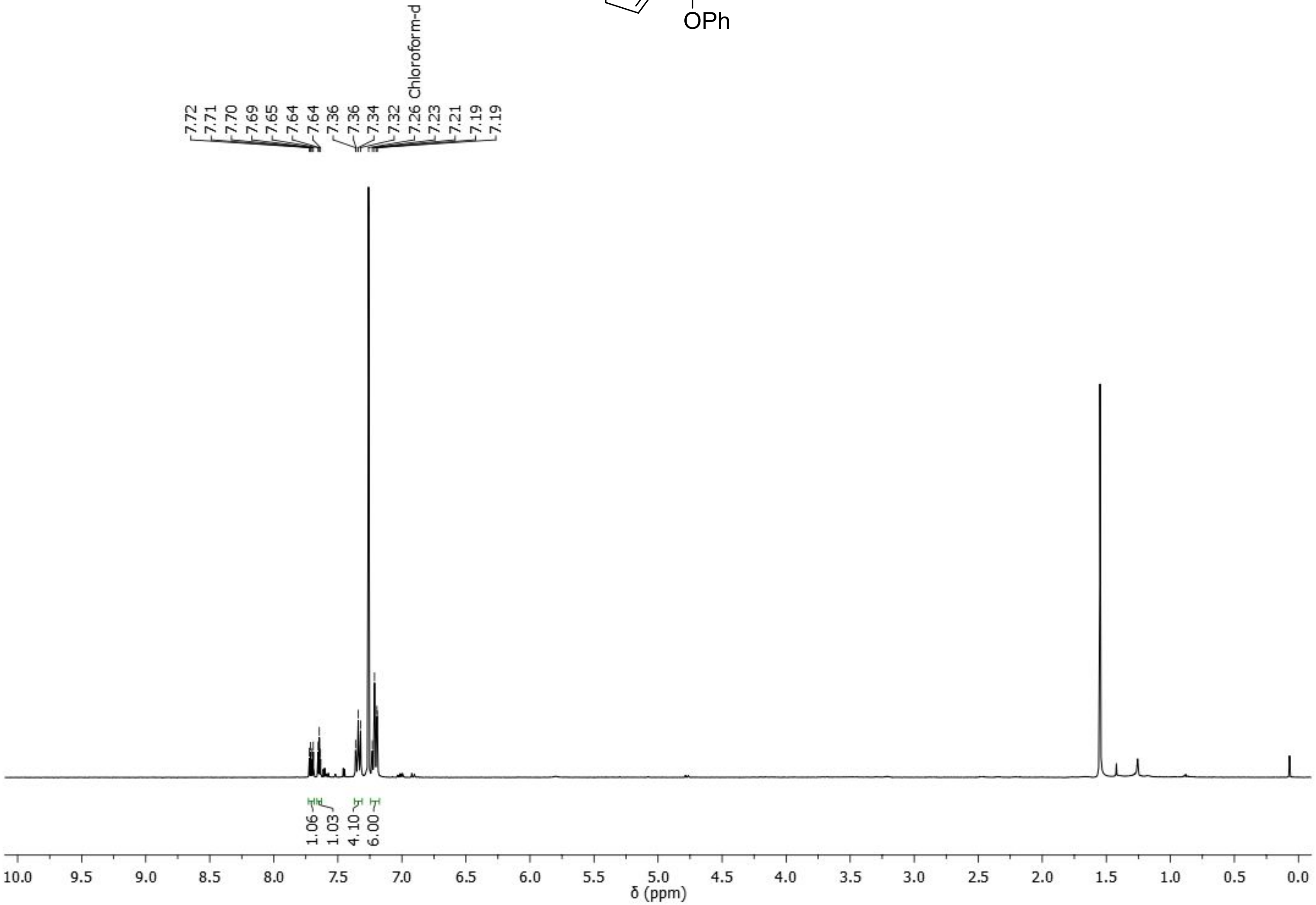

Figure S21. ^{1}H NMR spectrum of **3c**.

^{13}C 101MHz, $CDCl_3$

3c

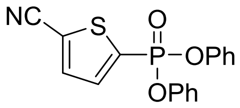

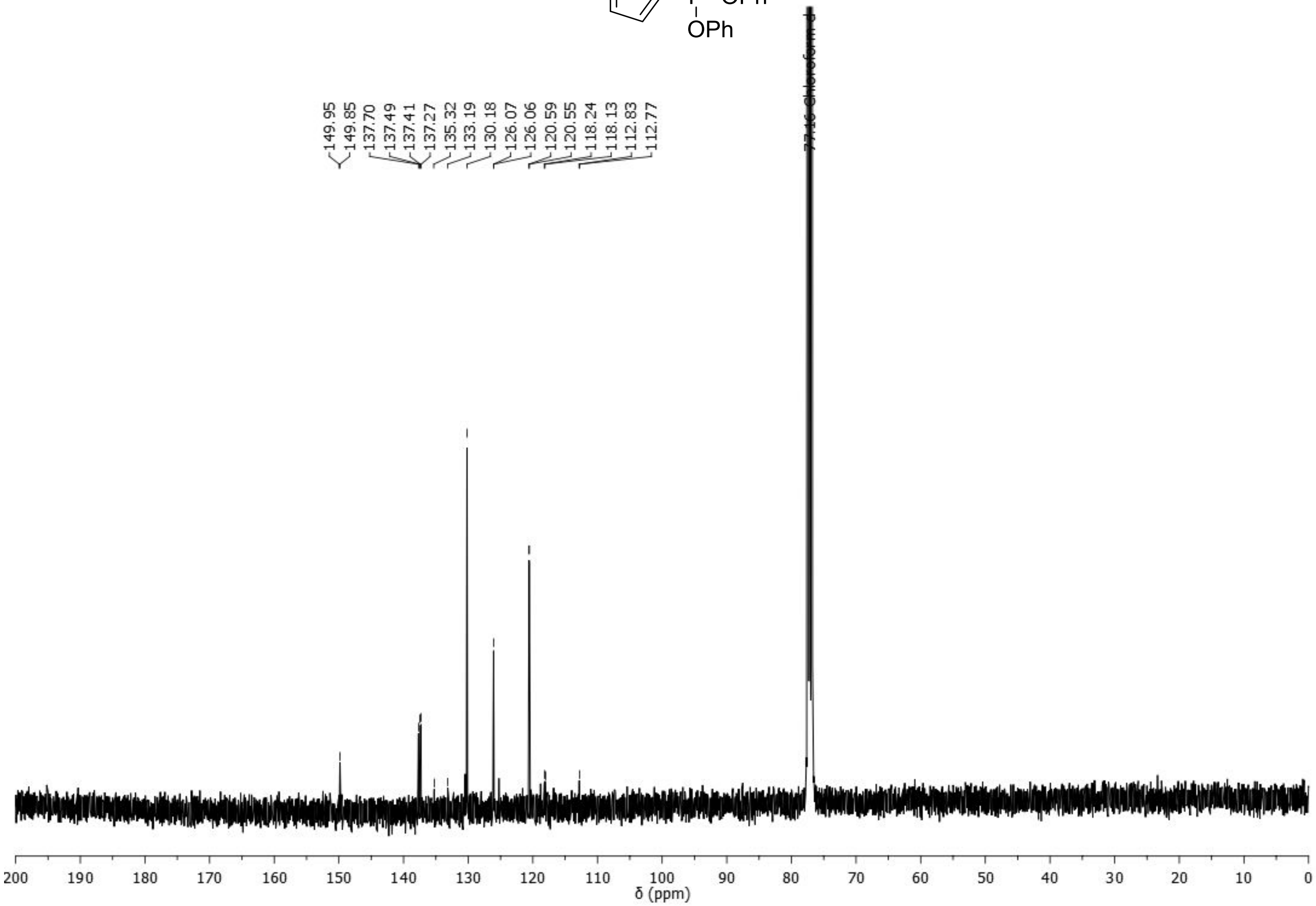

Figure S22. ^{13}C NMR spectrum of **3c**.

^{31}P 162MHz, $CDCl_3$

3c

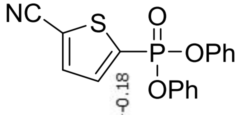

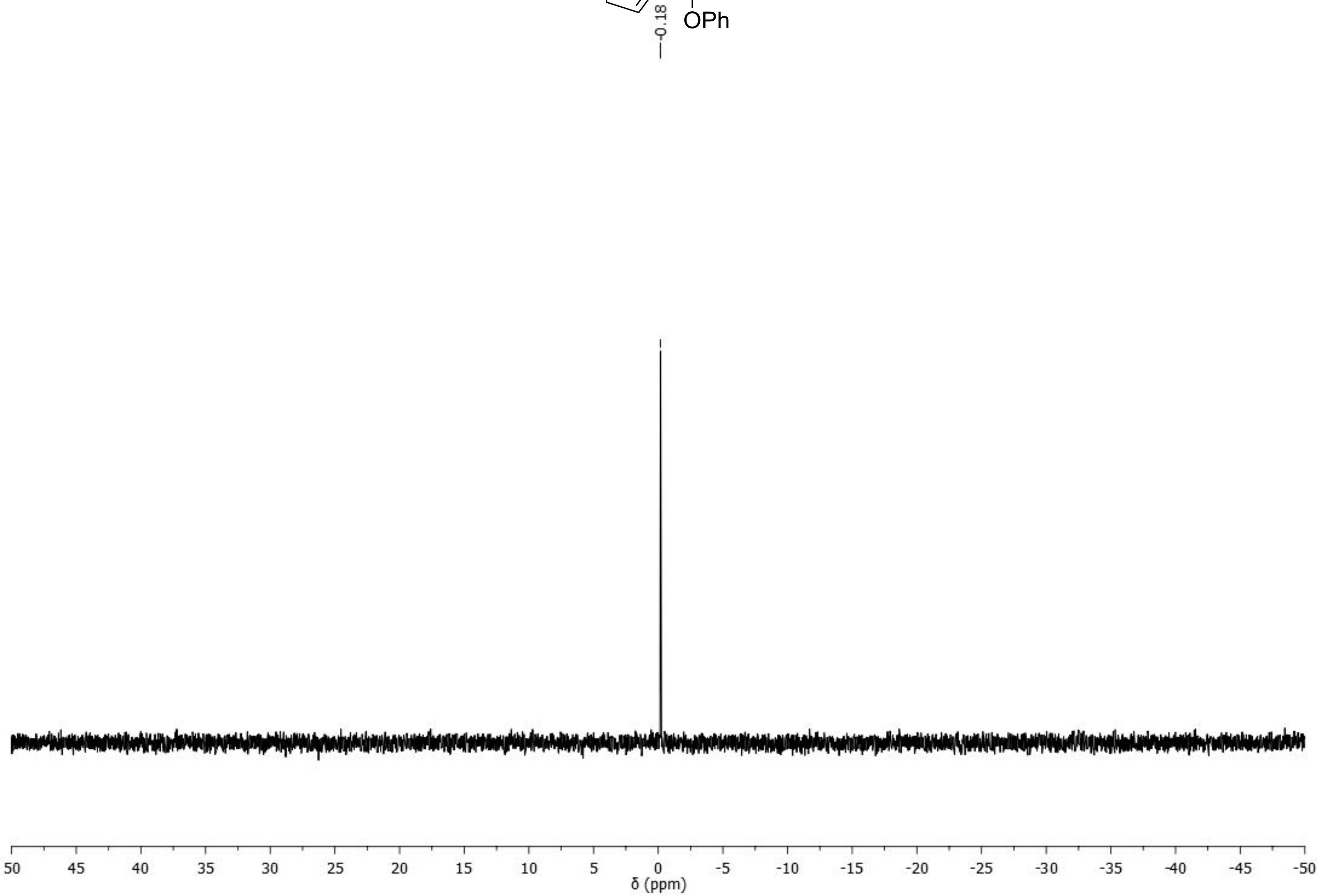

Figure S23. ^{31}P NMR spectrum of **3c**.

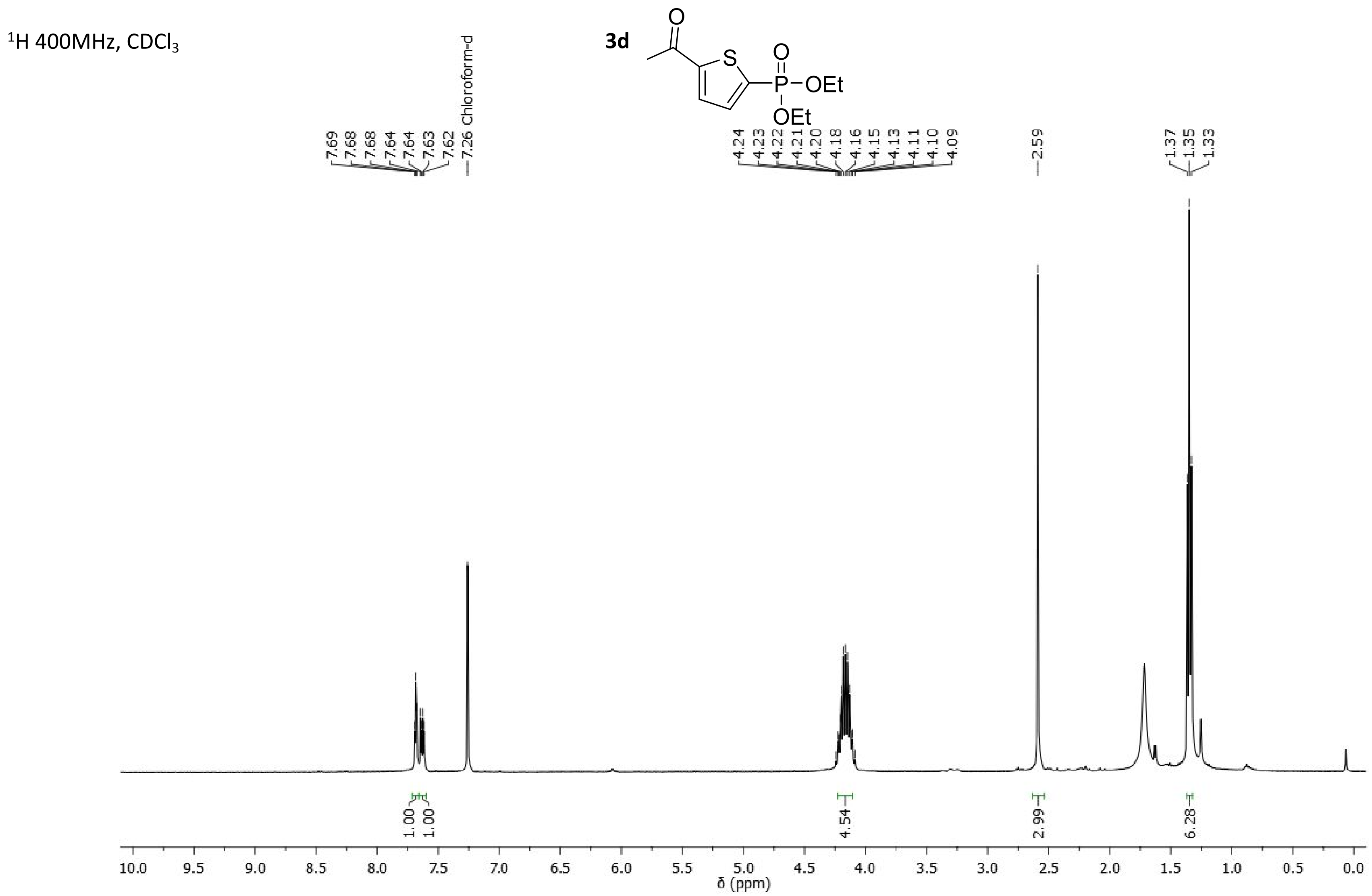

Figure S24. ^{1}H NMR spectrum of **3d**.

^{13}C 101MHz, $CDCl_3$

3d

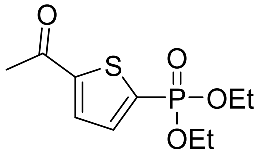

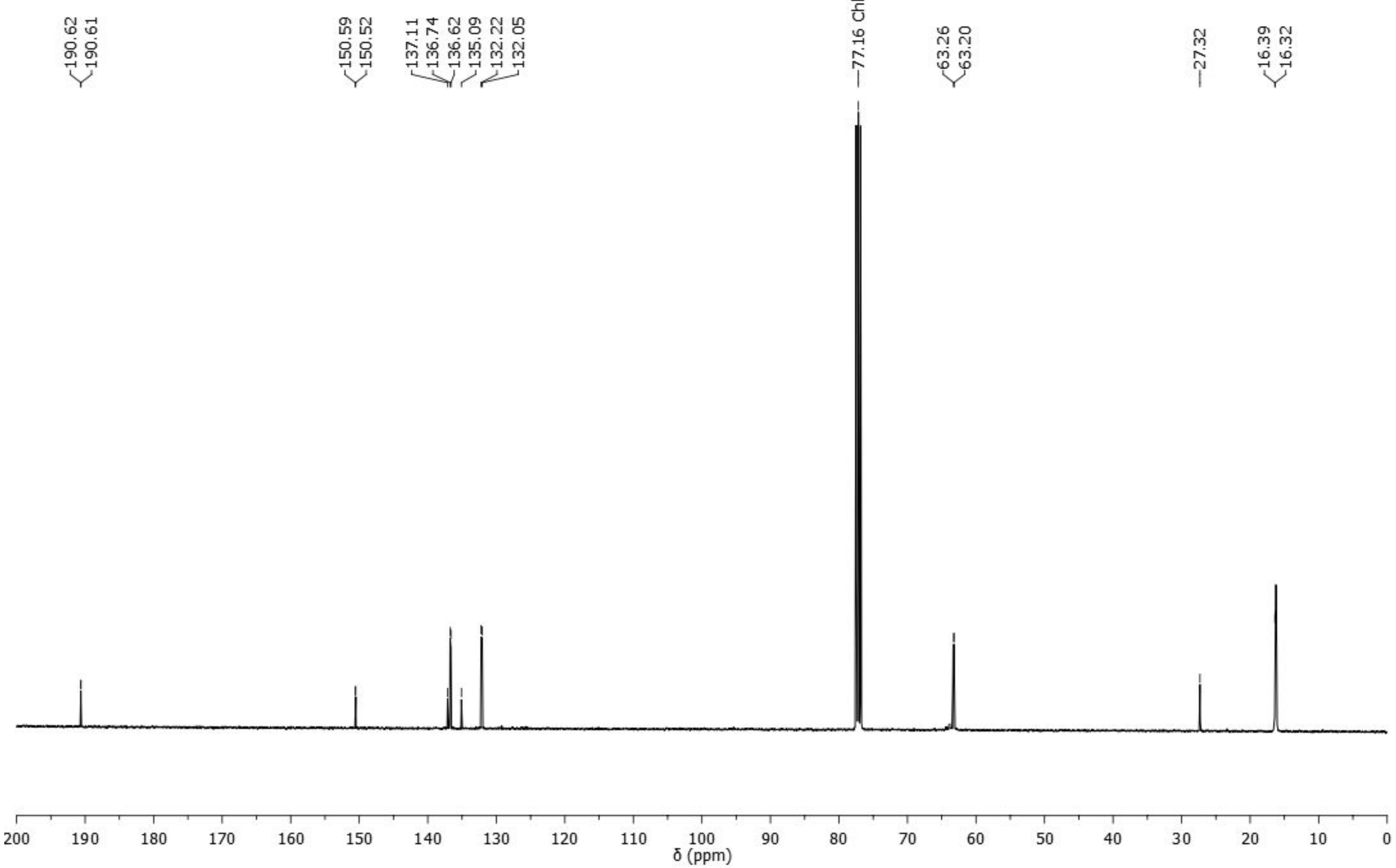

Figure S25. ^{13}C NMR spectrum of **3d**.

^{31}P 162MHz, $CDCl_3$

3d

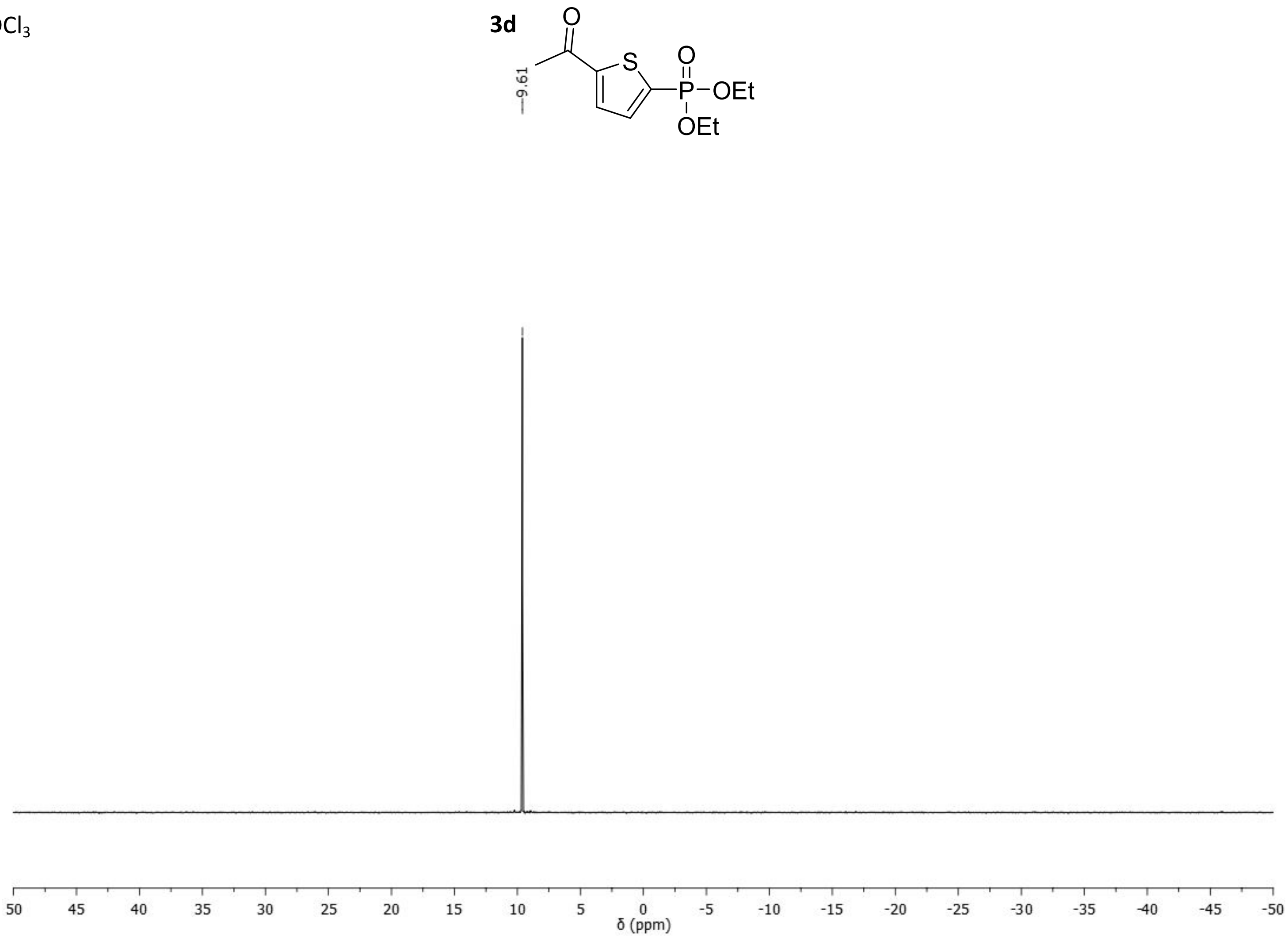

Figure S26. ^{31}P NMR spectrum of **3d**.

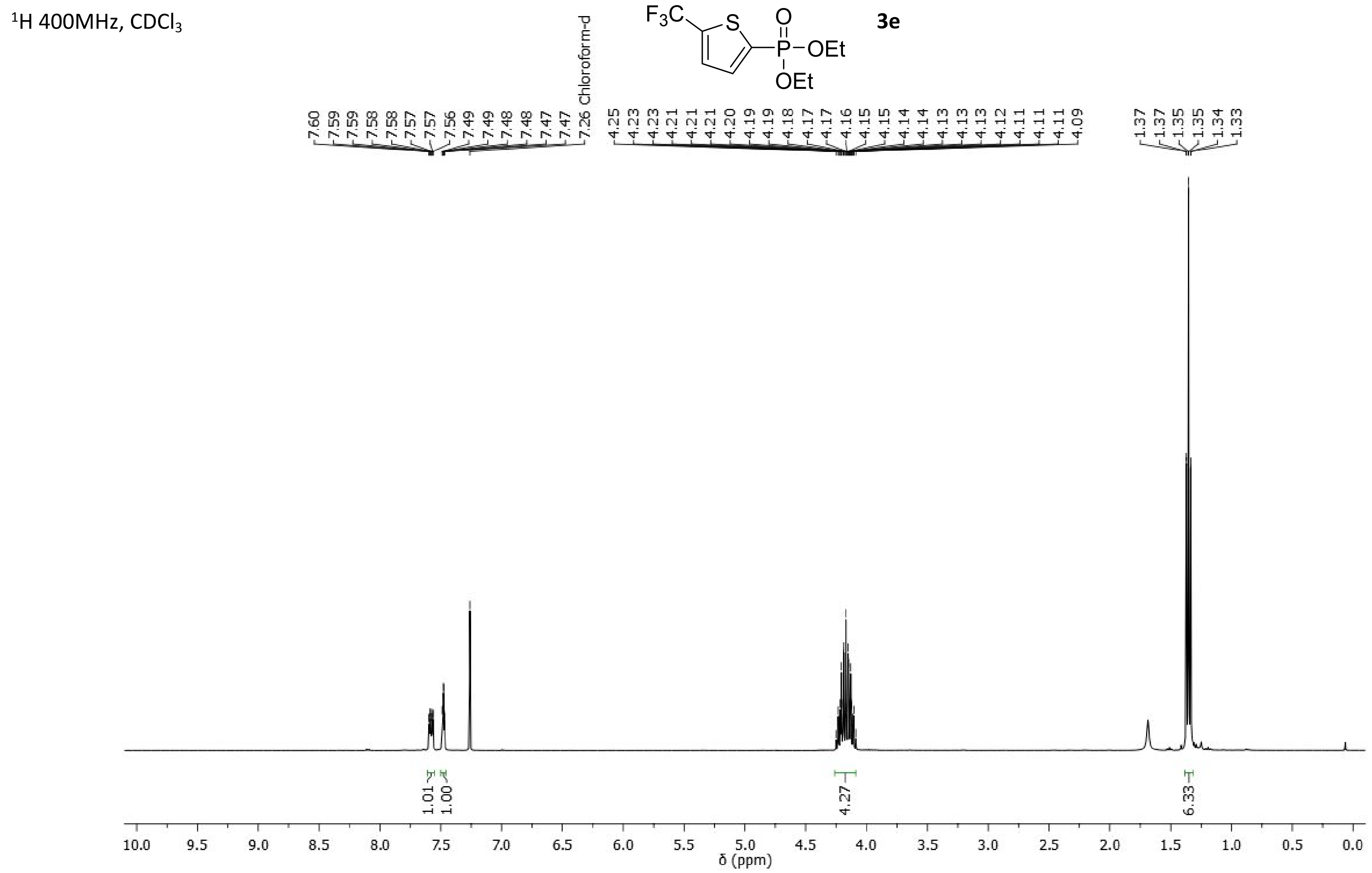

Figure S27. ^{1}H NMR spectrum of **3e**.

^{13}C 101MHz, $CDCl_3$

3e

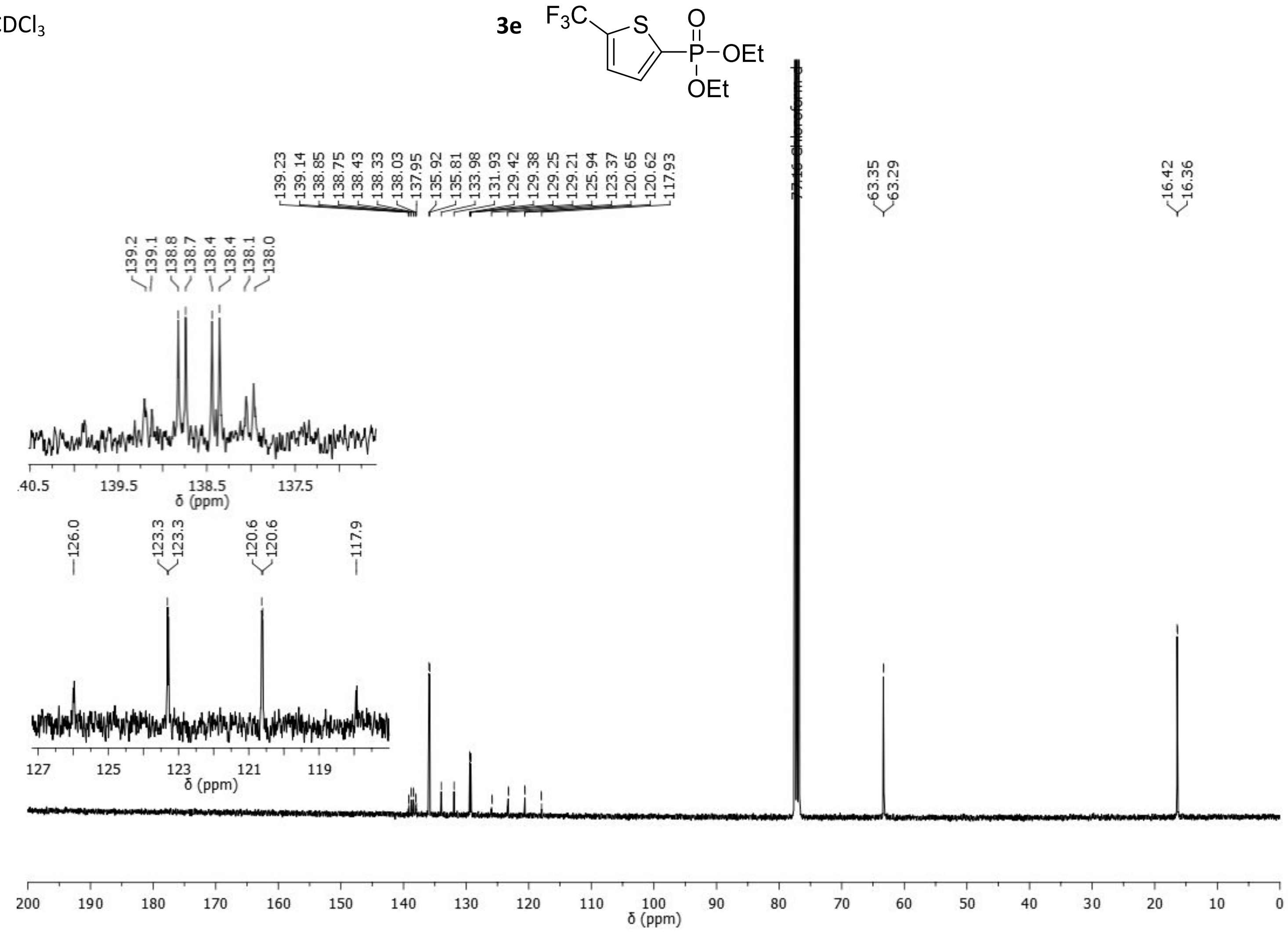

Figure S28. ^{13}C NMR spectrum of **3e**.

^{19}F 376 MHz, $CDCl_3$

3e

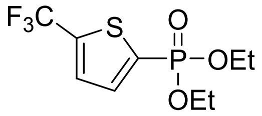

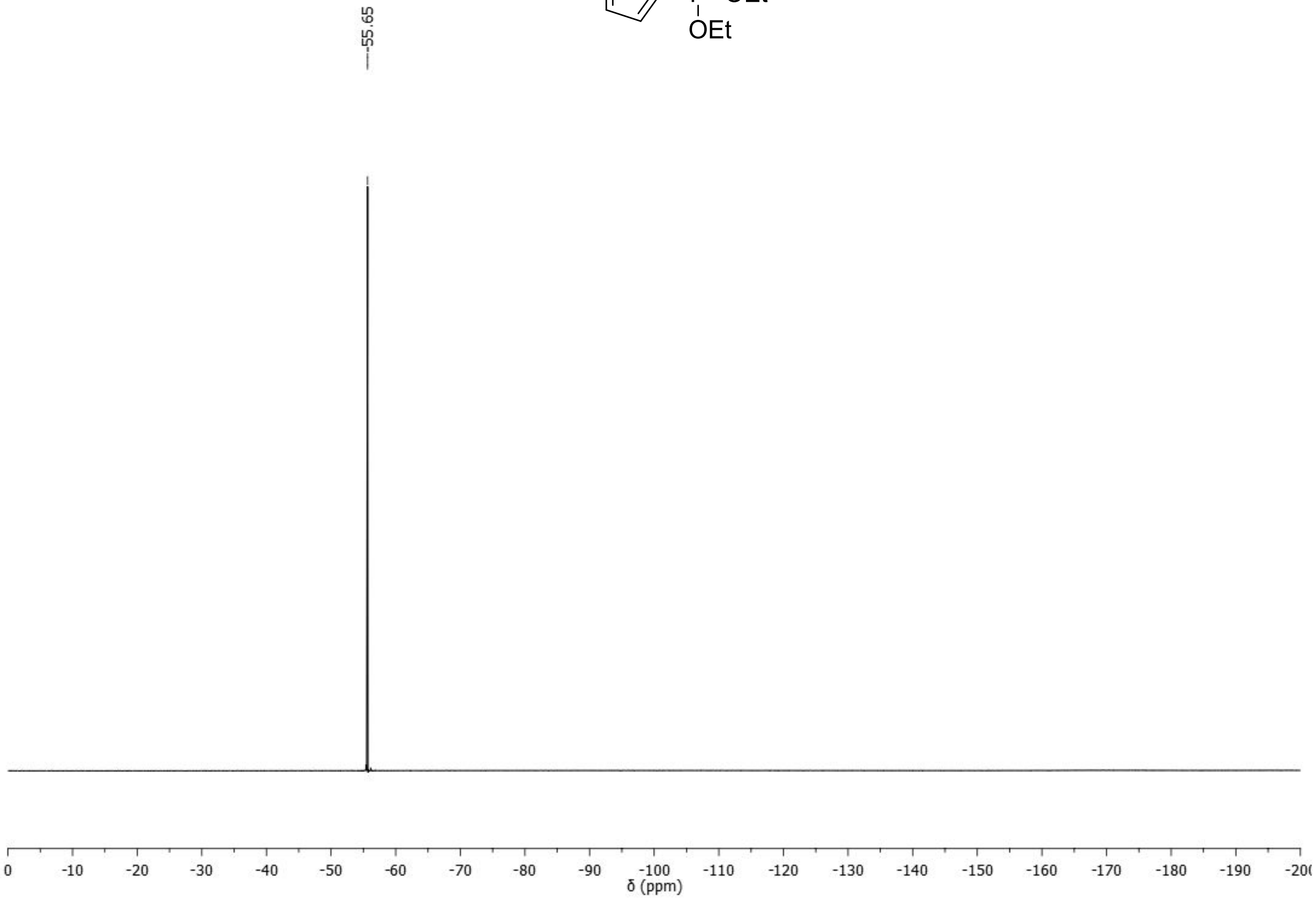

Figure S29. ^{19}F NMR spectrum of **3e**.

^{31}P 162MHz, $CDCl_3$

3e

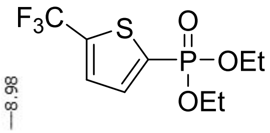

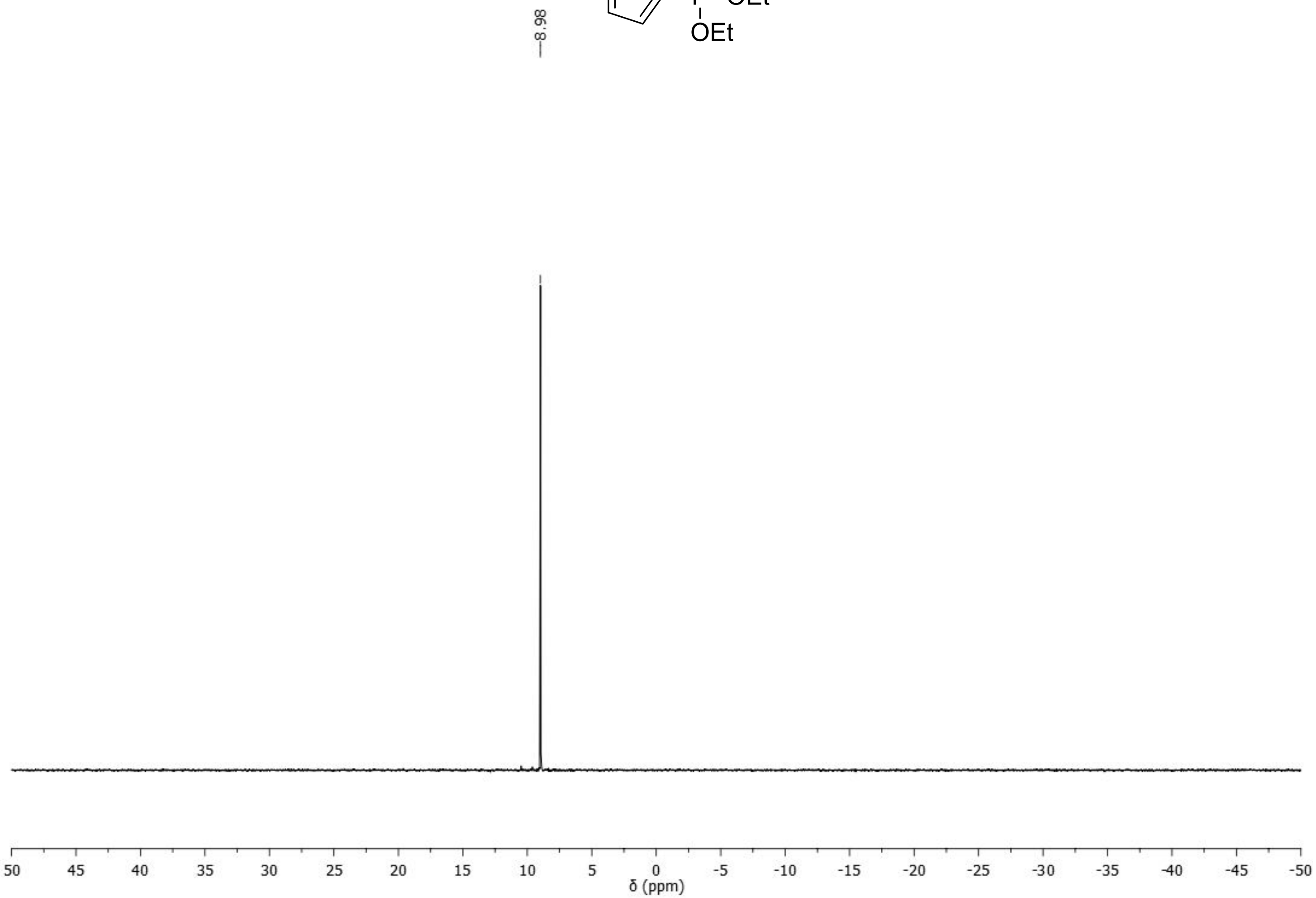

Figure S30. ^{31}P NMR spectrum of **3e**.

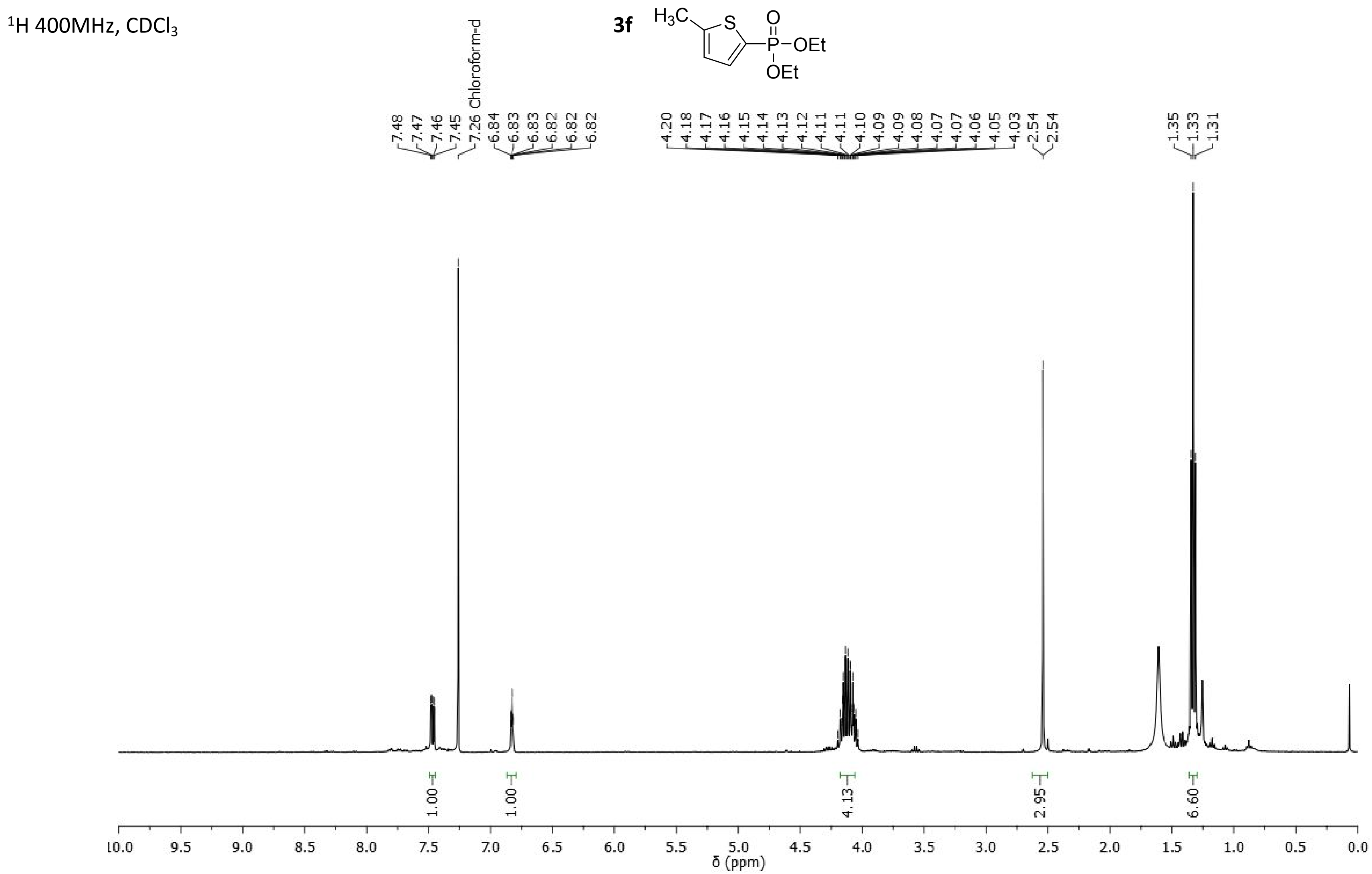

Figure S31. ^{1}H NMR spectrum of **3f**.

^{13}C 101MHz, $CDCl_3$

3f

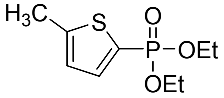

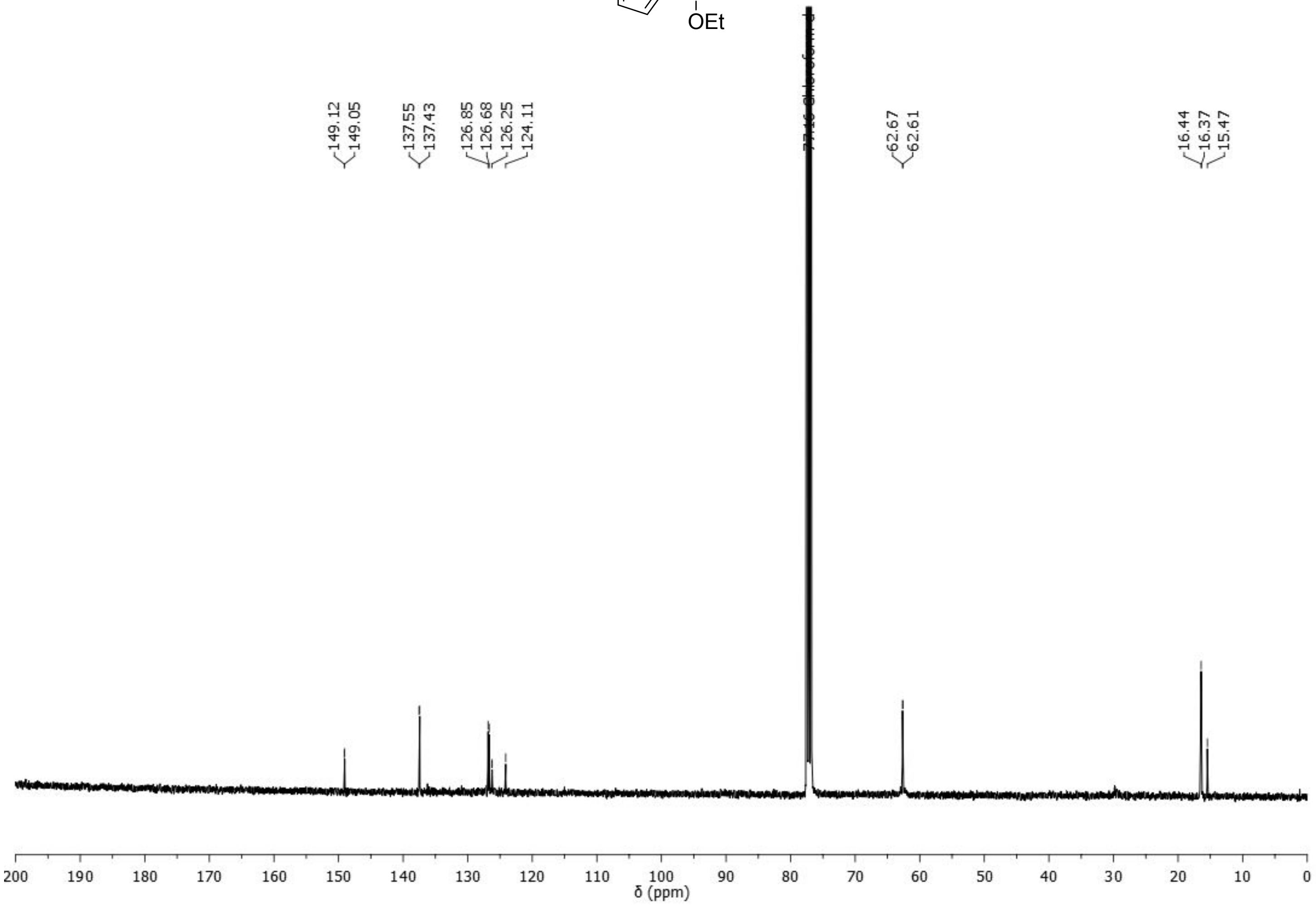

Figure S32. ^{13}C NMR spectrum of **3f**.

^{31}P 162MHz, $CDCl_3$

3f

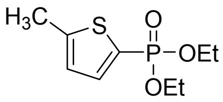

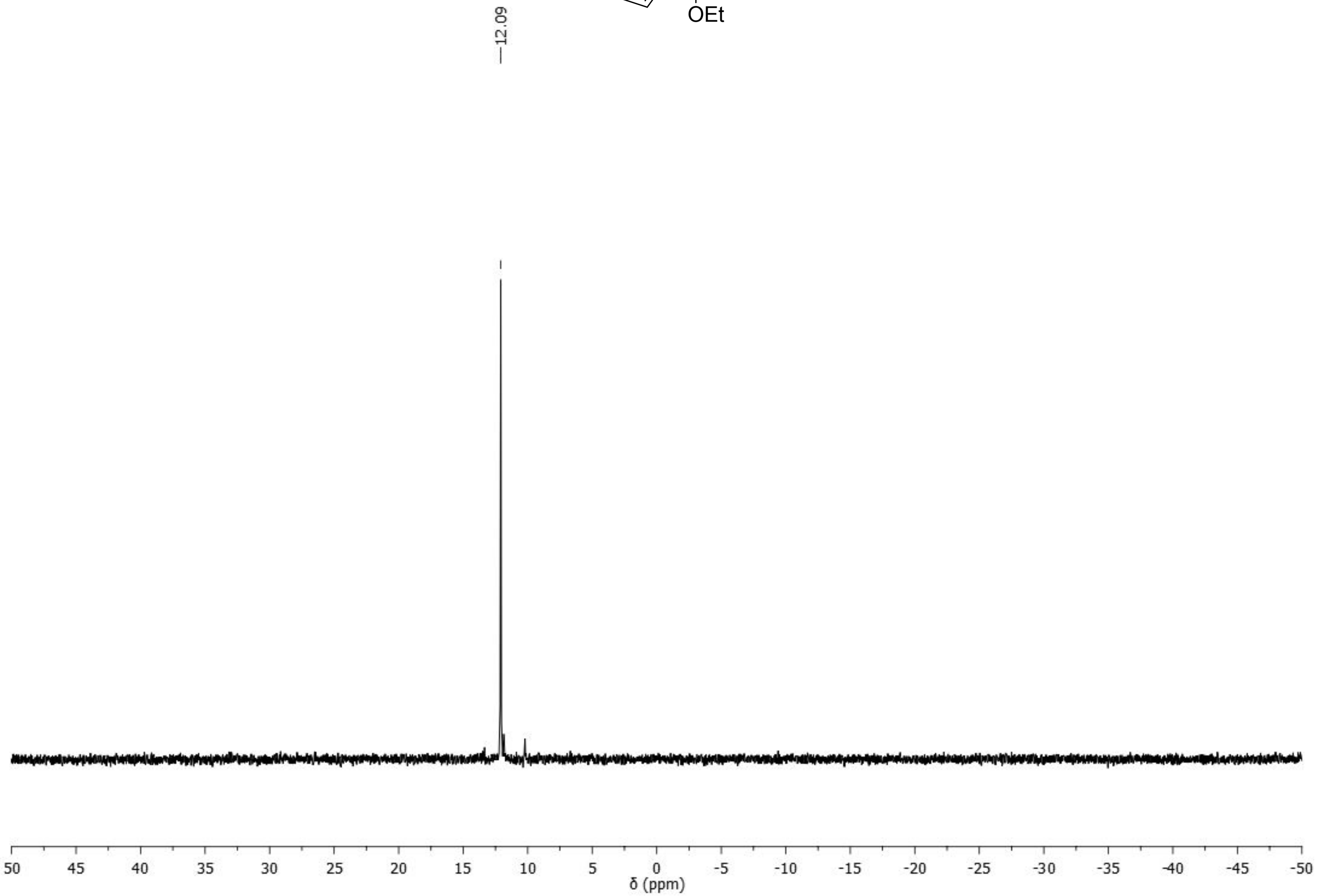

Figure S33. ^{31}P NMR spectrum of **3f**.

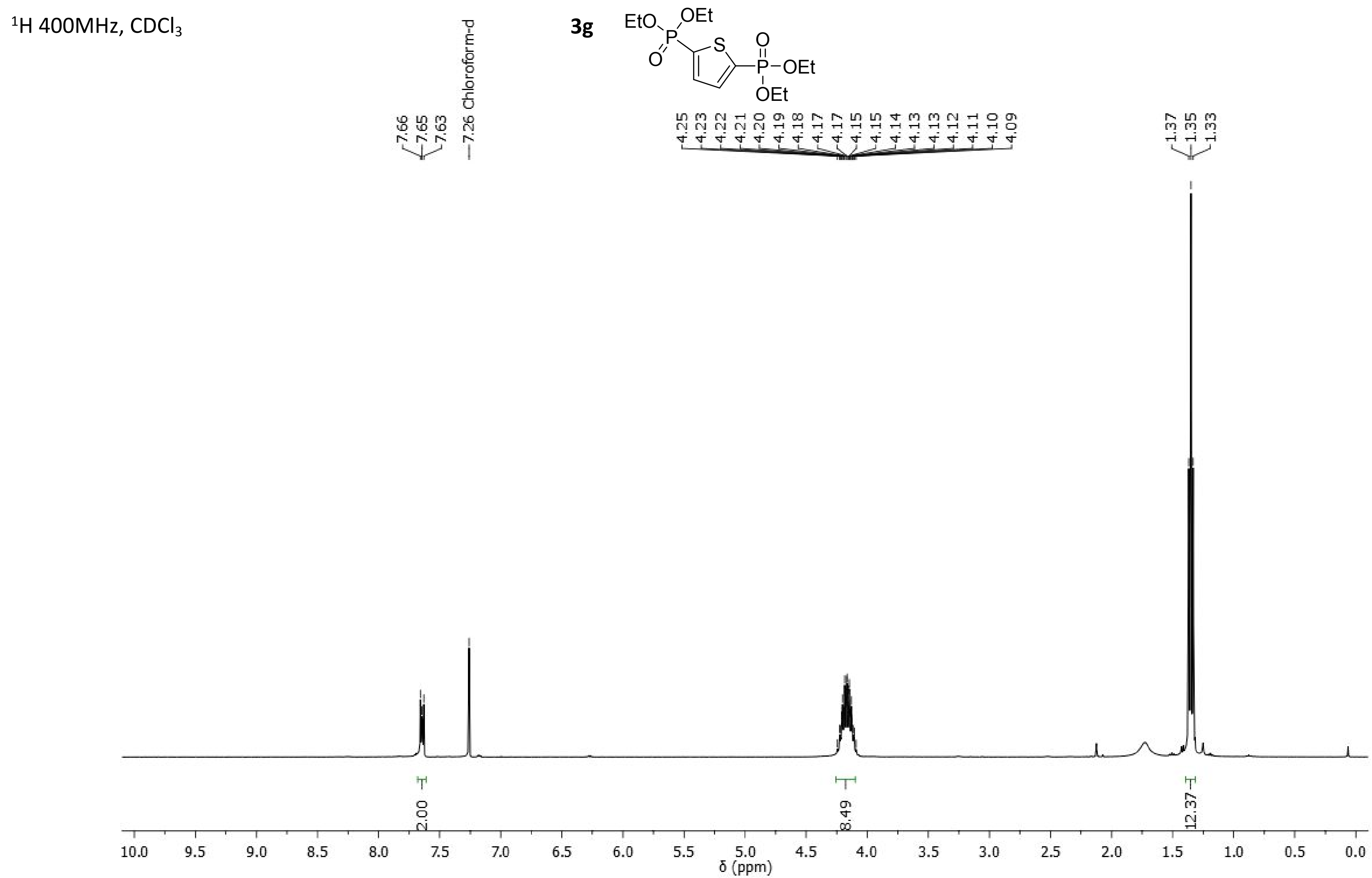

Figure S34. ^{1}H NMR spectrum of **3g**.

^{13}C 101MHz, $CDCl_3$

3g

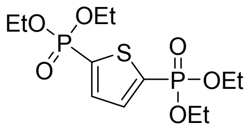

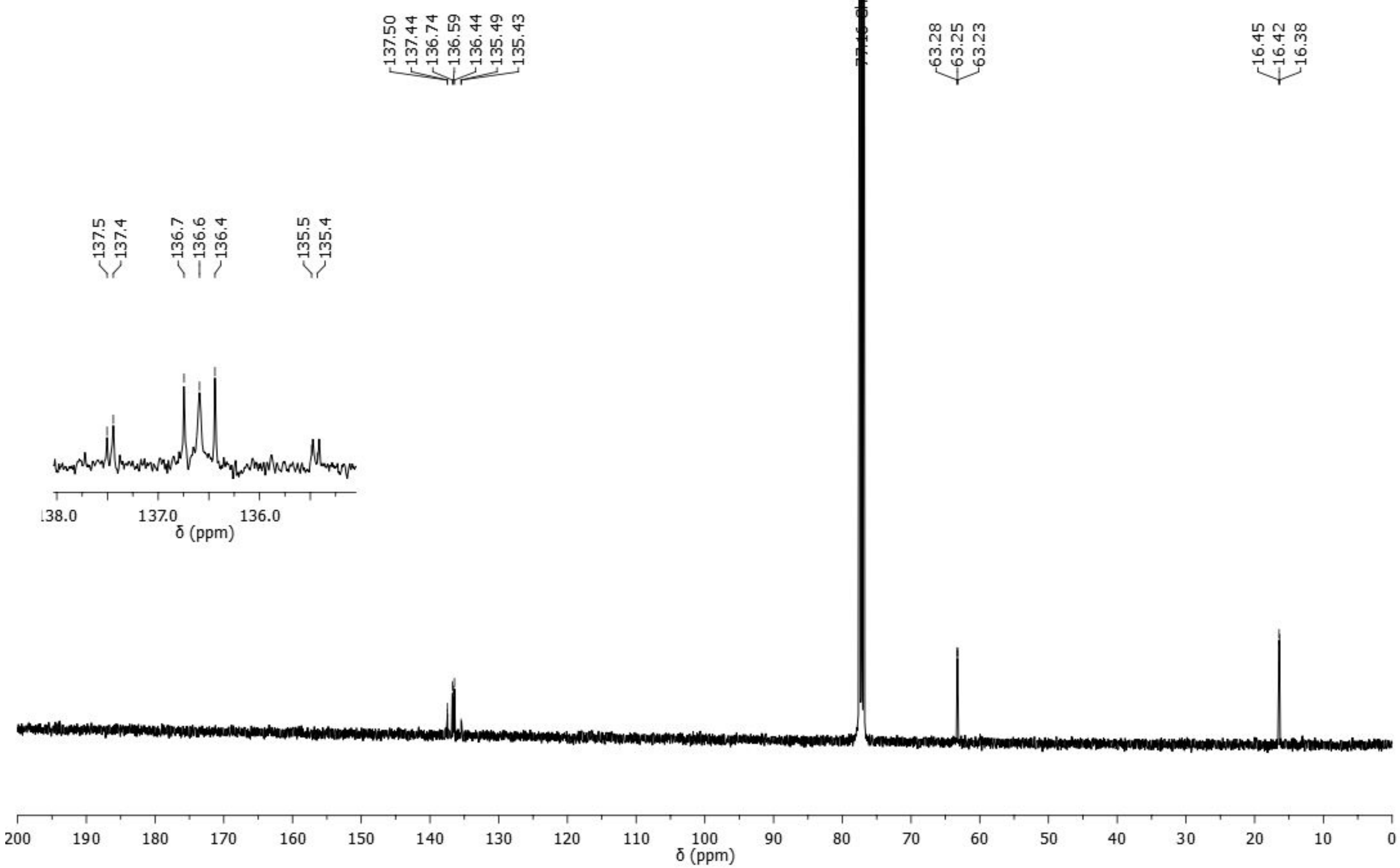

Figure S35. ^{13}C NMR spectrum of **3g**.

^{31}P 162MHz, $CDCl_3$

3g

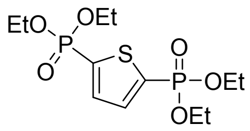

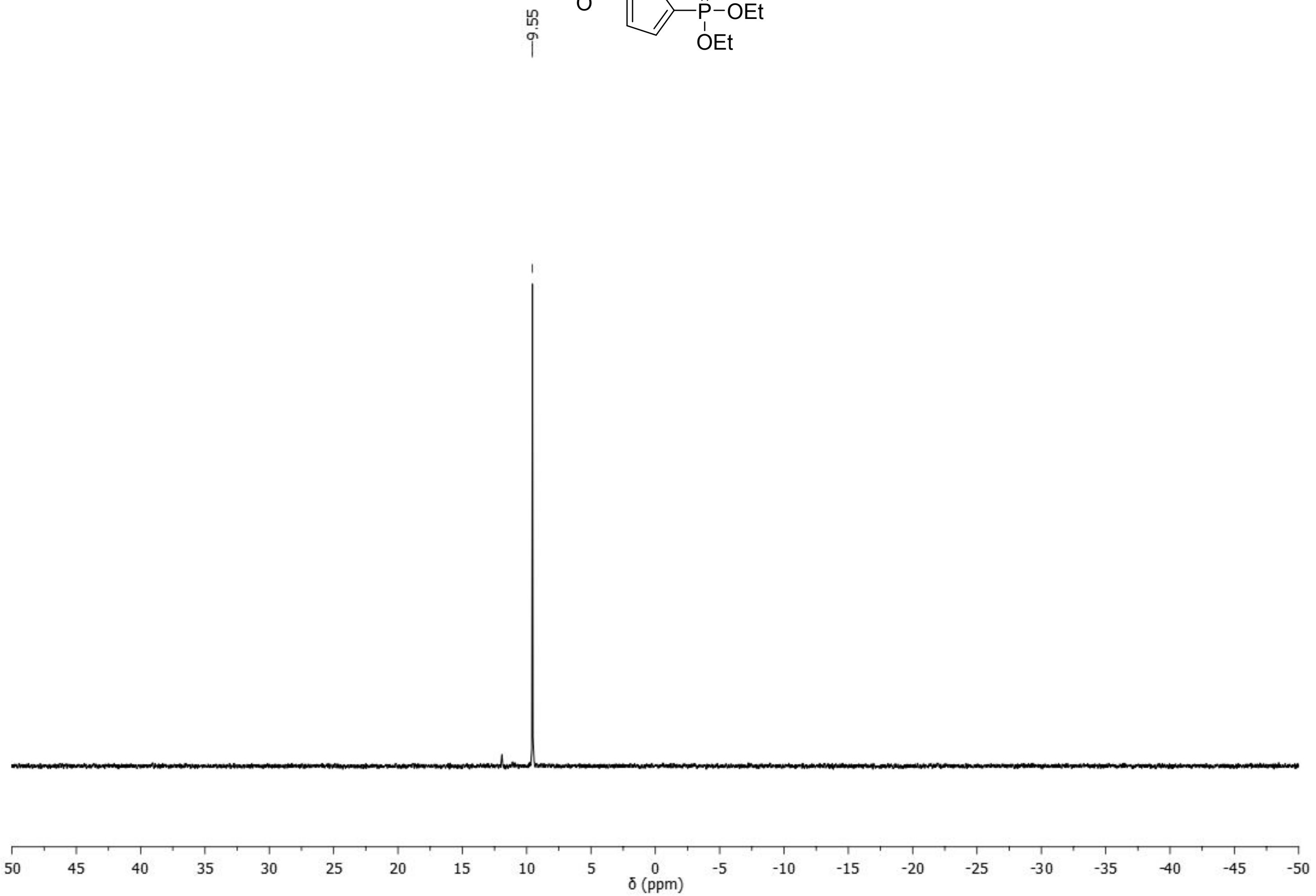

Figure S36. ^{31}P NMR spectrum of **3g**.

^1H 400MHz, $CDCl_3$

3h

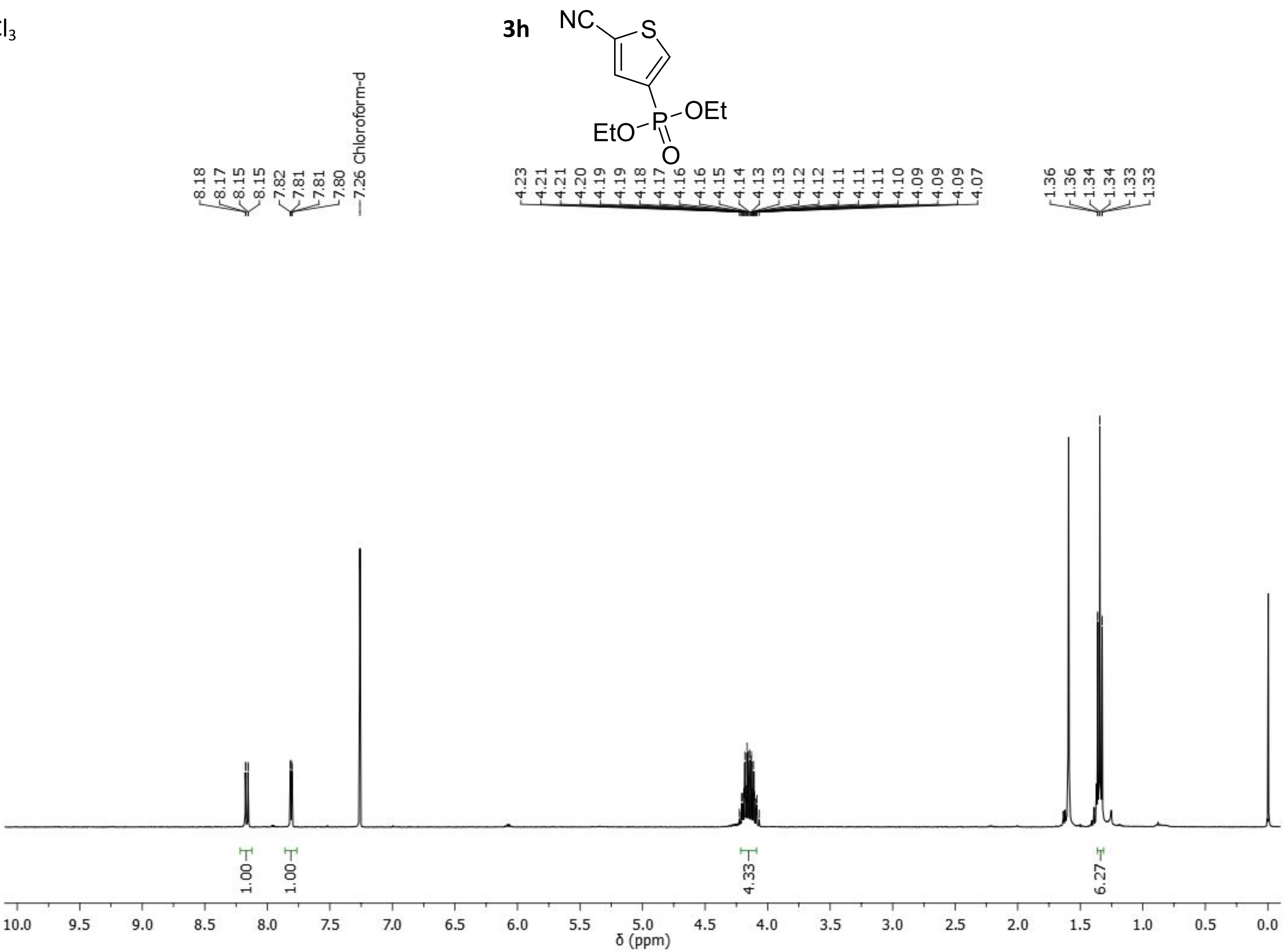

Figure S37. ^1H NMR spectrum of **3h**.

^{13}C 101MHz, $CDCl_3$

3h

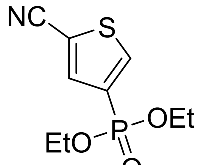

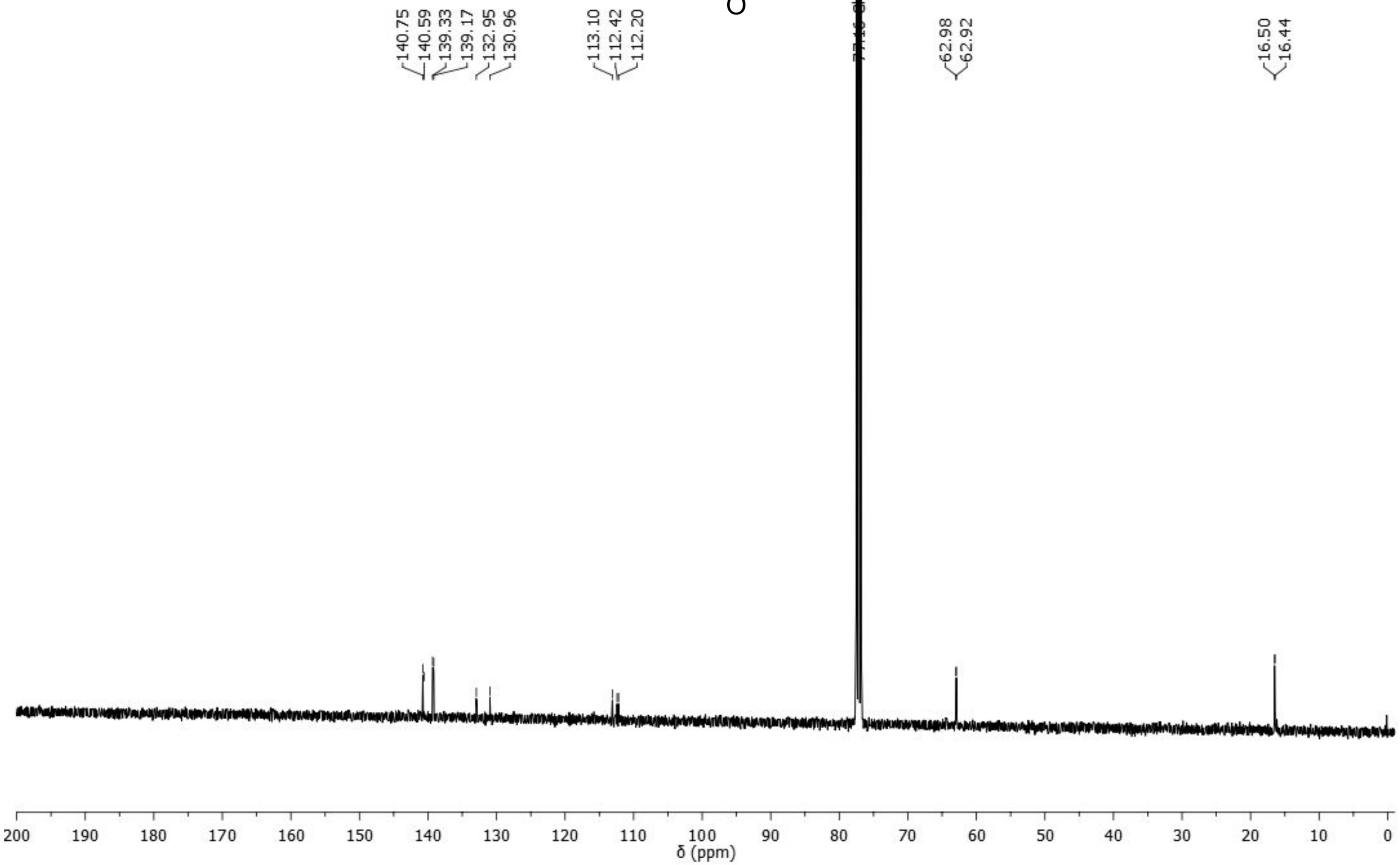

Figure S38. ^{13}C NMR spectrum of **3h.**

^{31}P 162MHz, $CDCl_3$

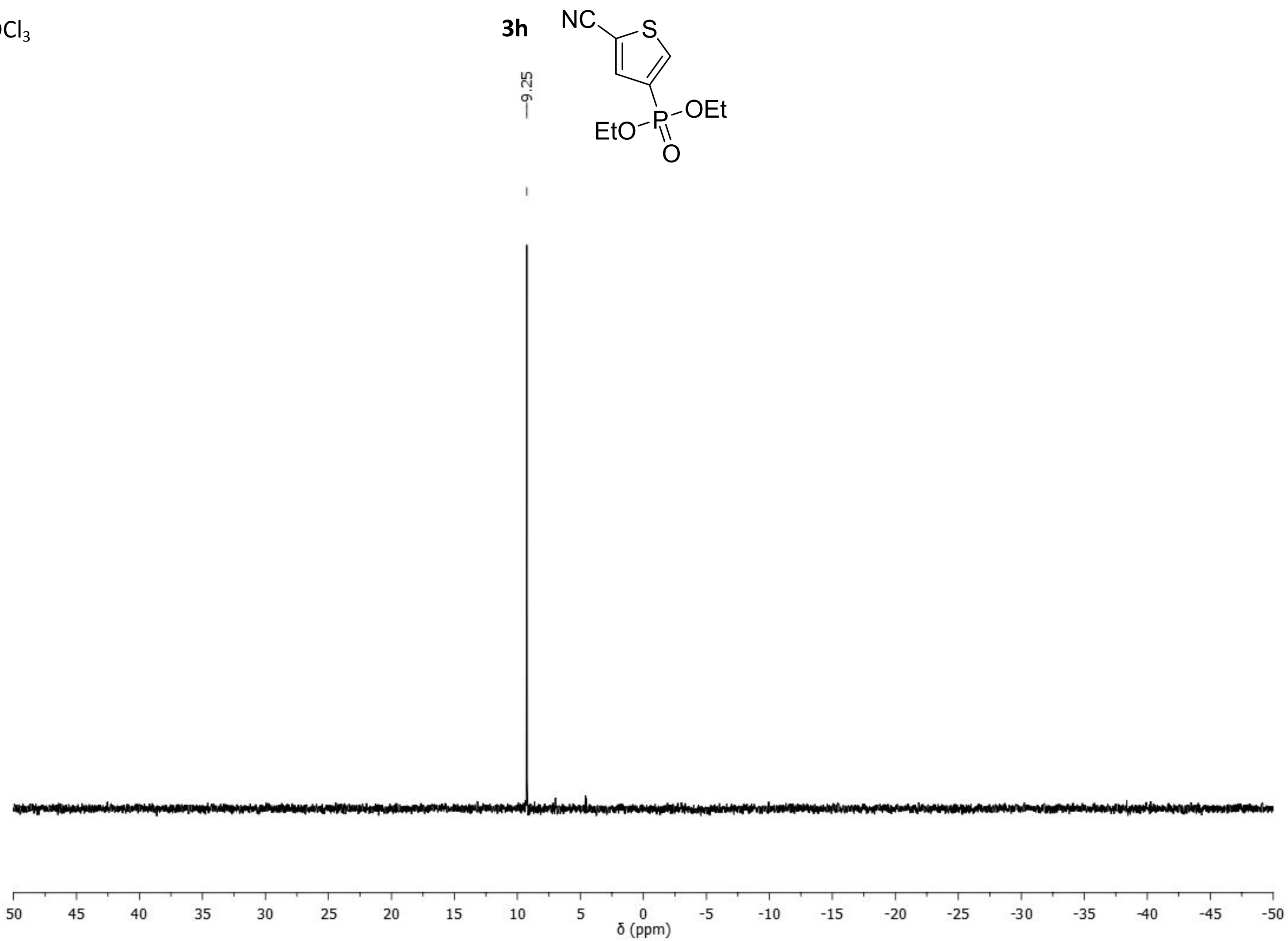

Figure S39. ^{31}P NMR spectrum of **3h.**

^{1}H 400MHz, $CDCl_3$

3i

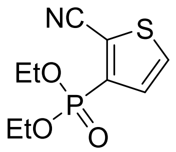

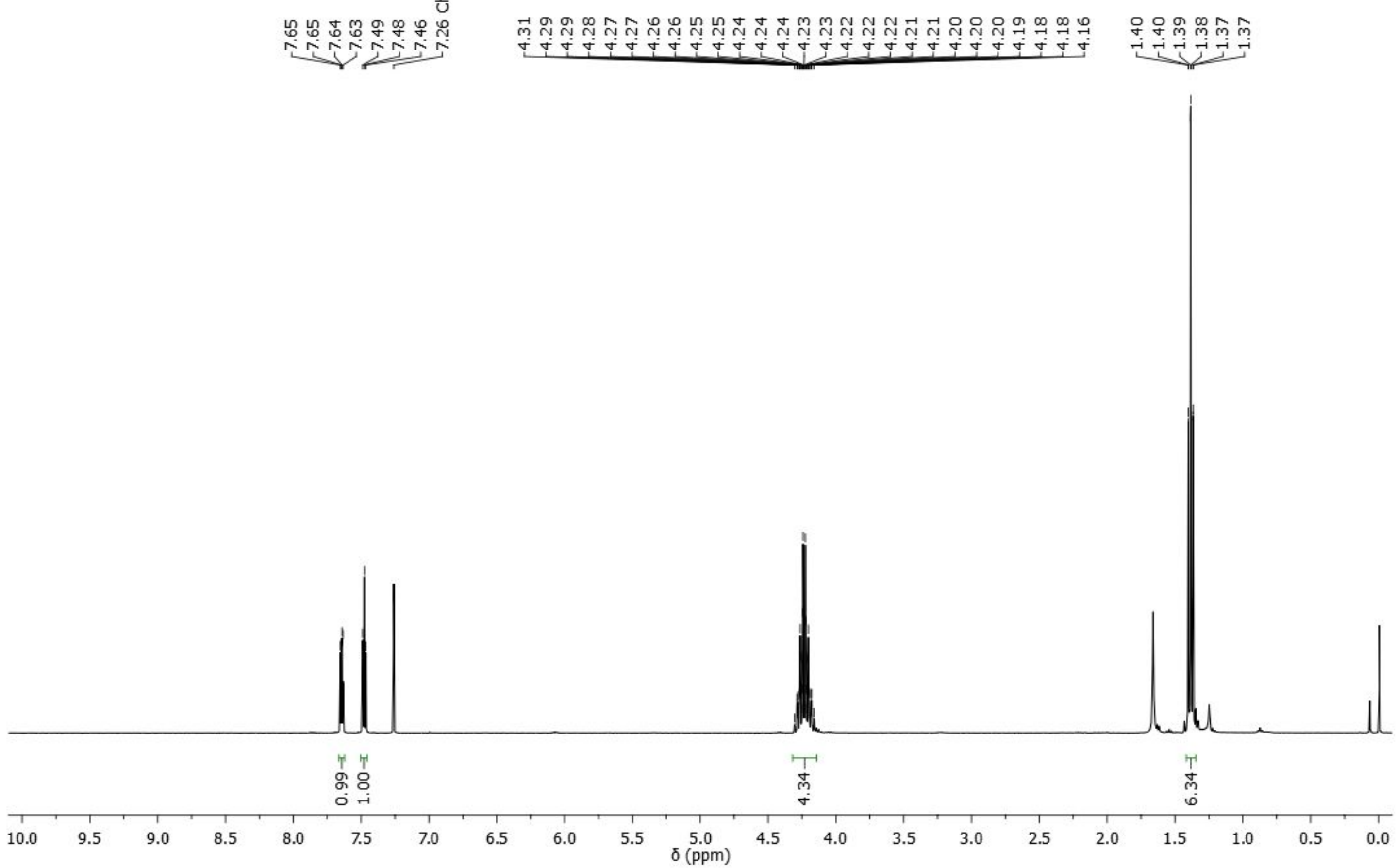

Figure S40. ^{1}H NMR spectrum of **3i.**

^{13}C 101MHz, $CDCl_3$

3i

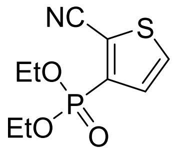

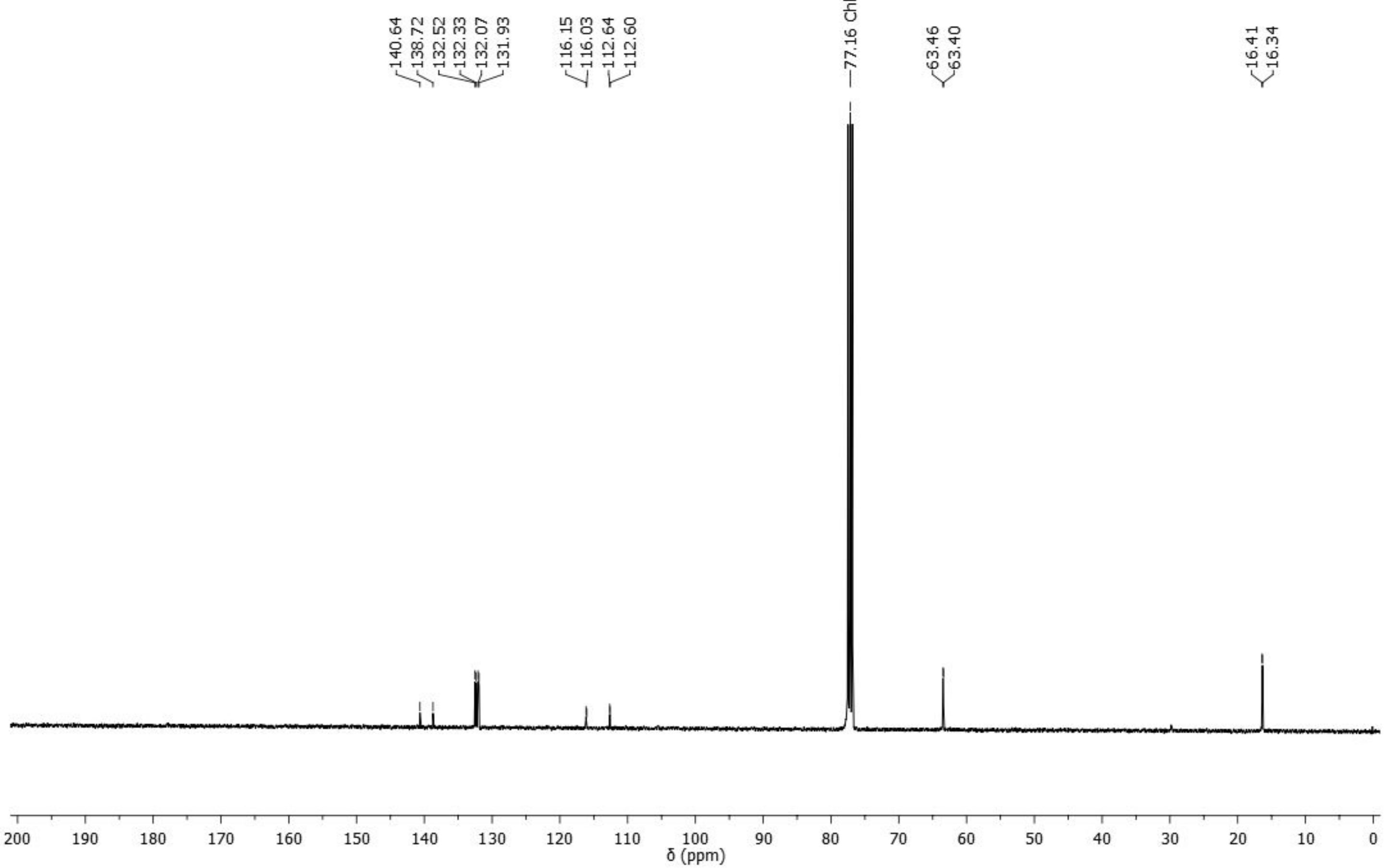

Figure S41. ^{13}C NMR spectrum of **3i.**

^{31}P 162MHz, $CDCl_3$

3i

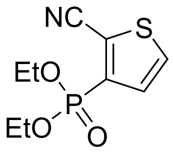

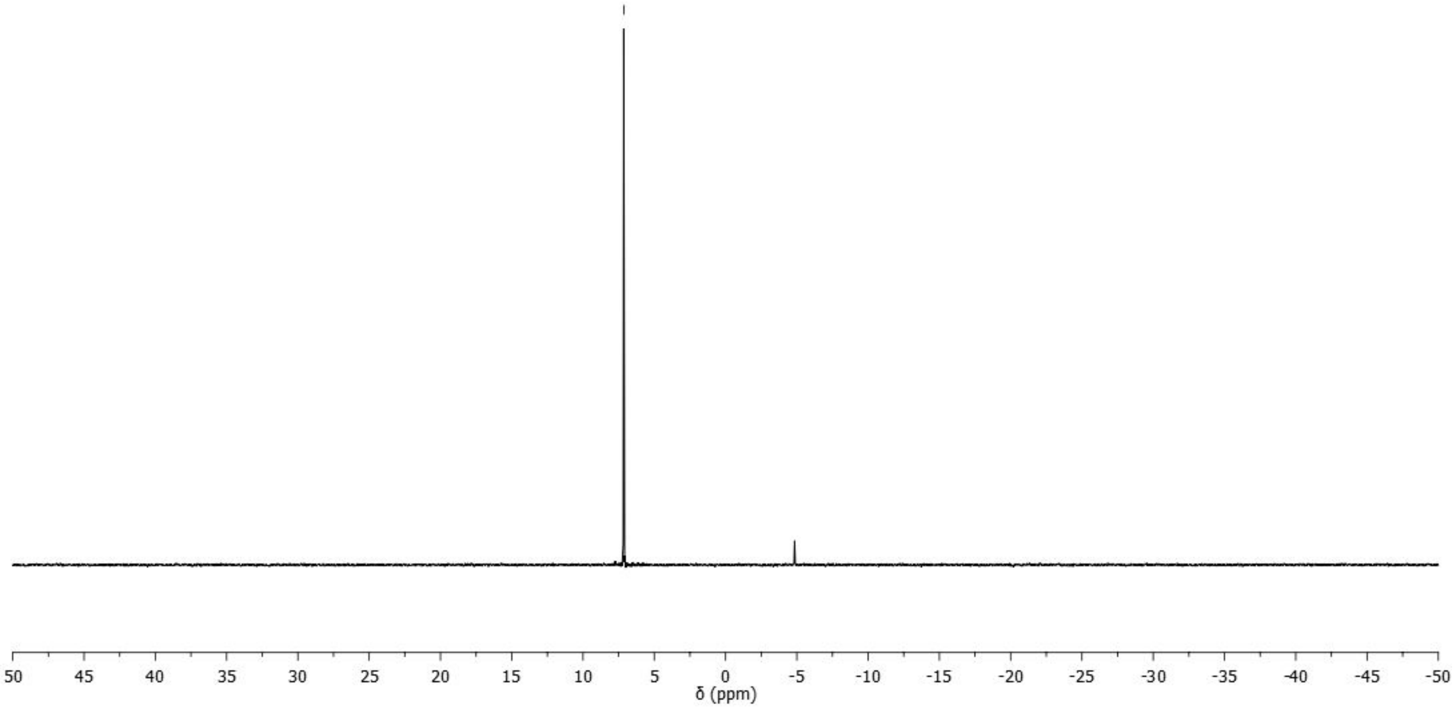

Figure S42. ^{31}P NMR spectrum of **3i.**

^1H 400MHz, $CDCl_3$

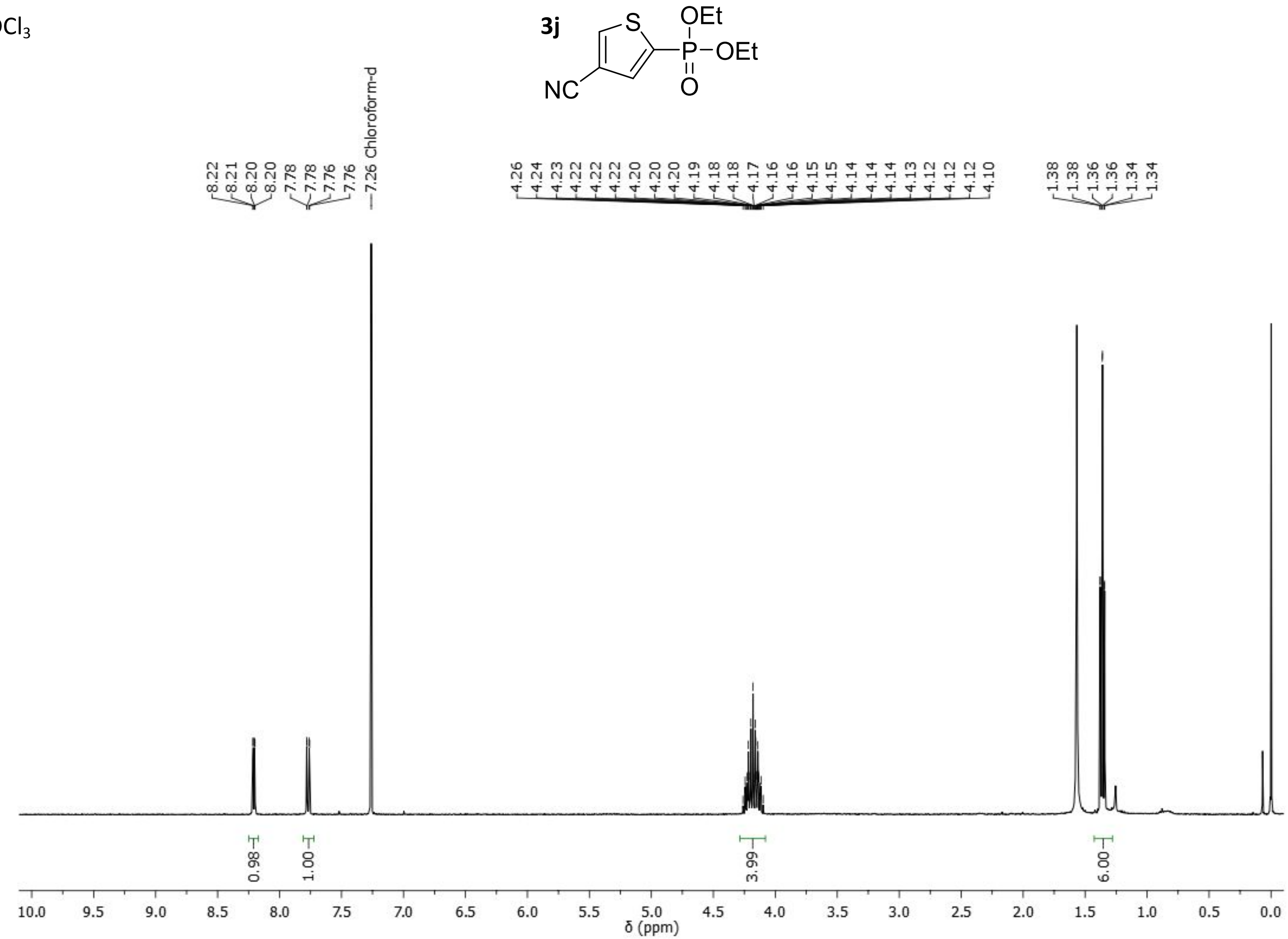

Figure S43. ^1H NMR spectrum of **3j.**

^{13}C 101MHz, $CDCl_3$

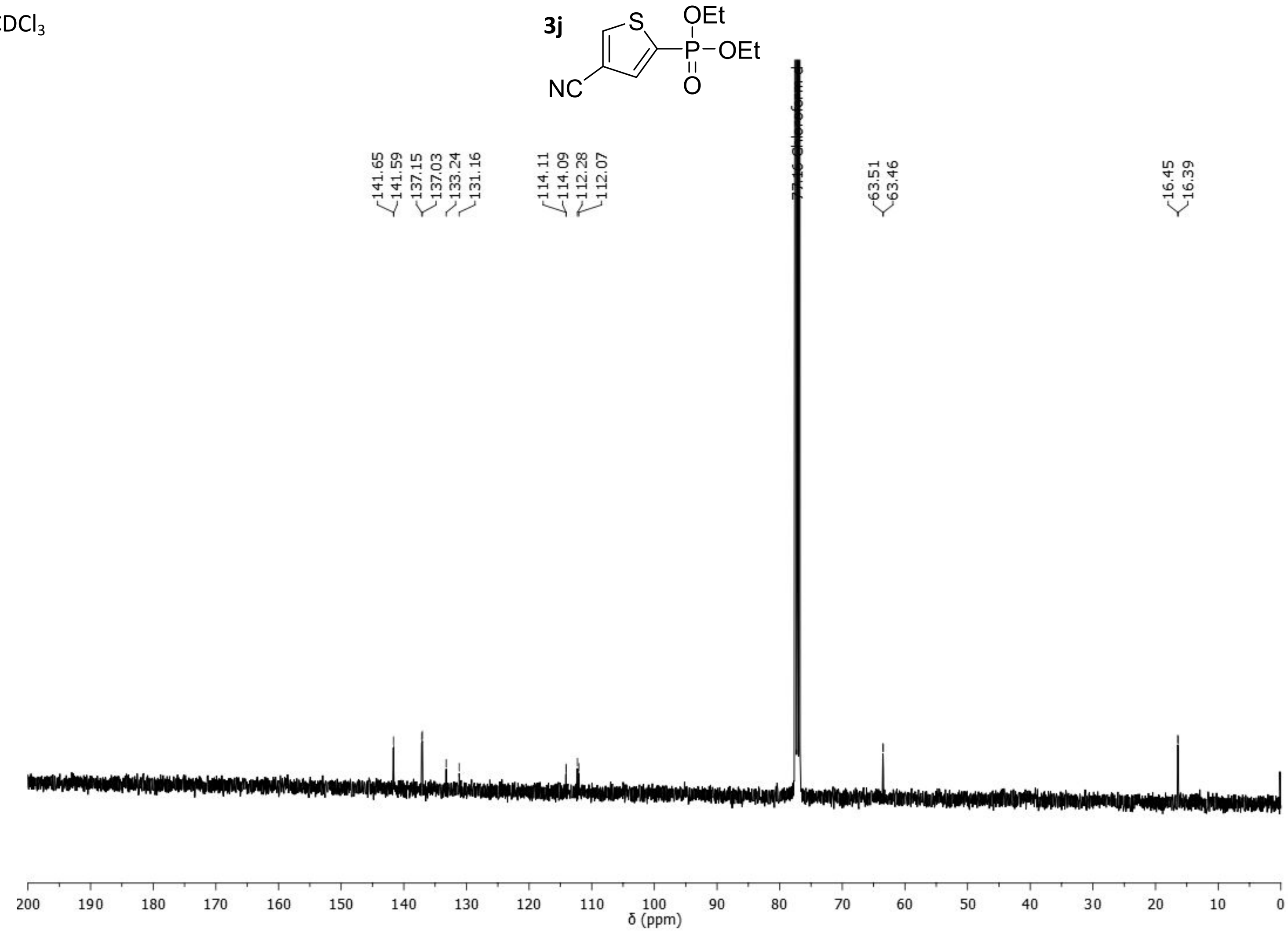

Figure S44. ^{13}C NMR spectrum of **3j.**

^{31}P 162MHz, $CDCl_3$

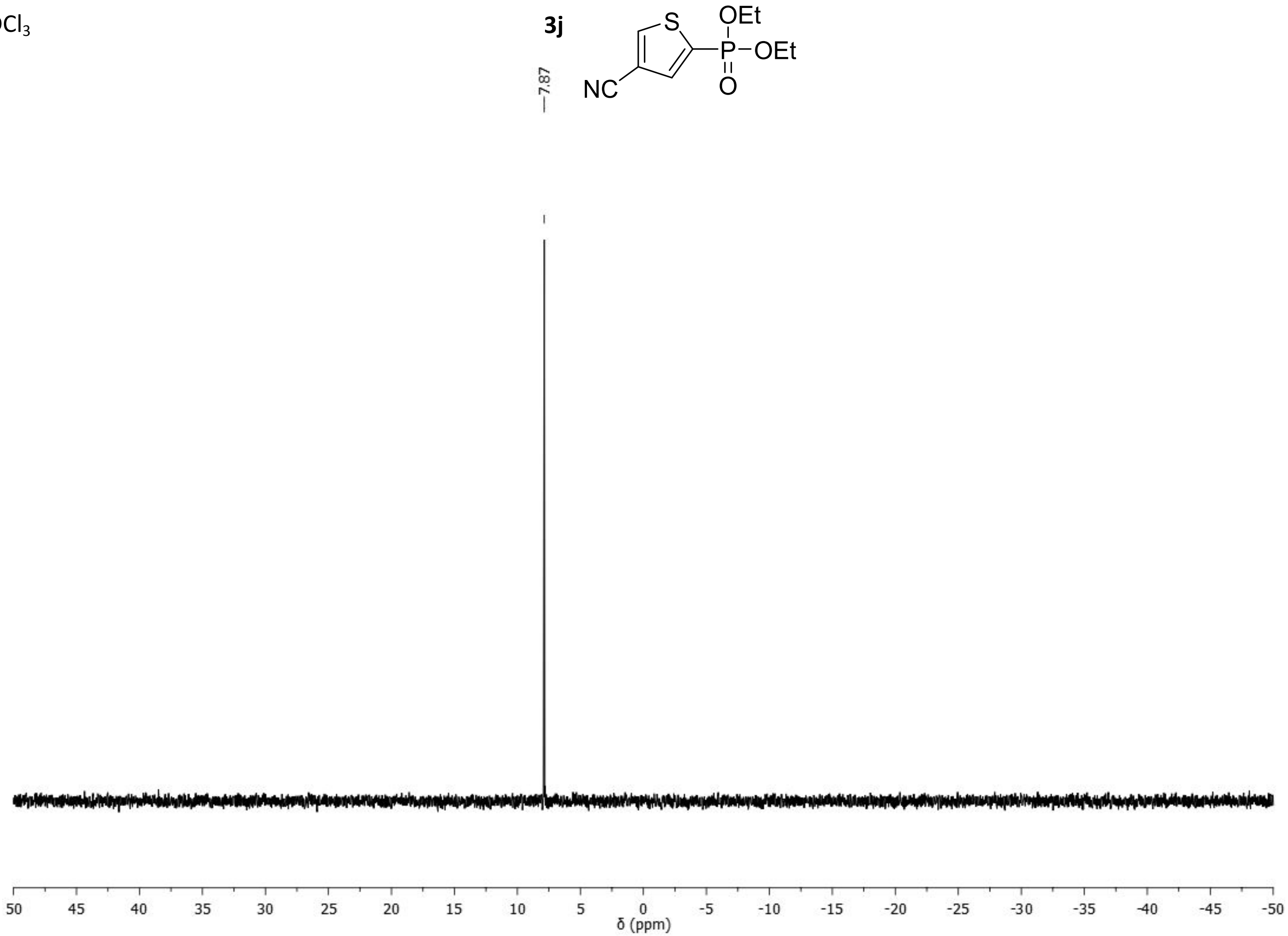

Figure S45. ^{31}P NMR spectrum of **3j.**

^{1}H 400MHz, $CDCl_3$

3k

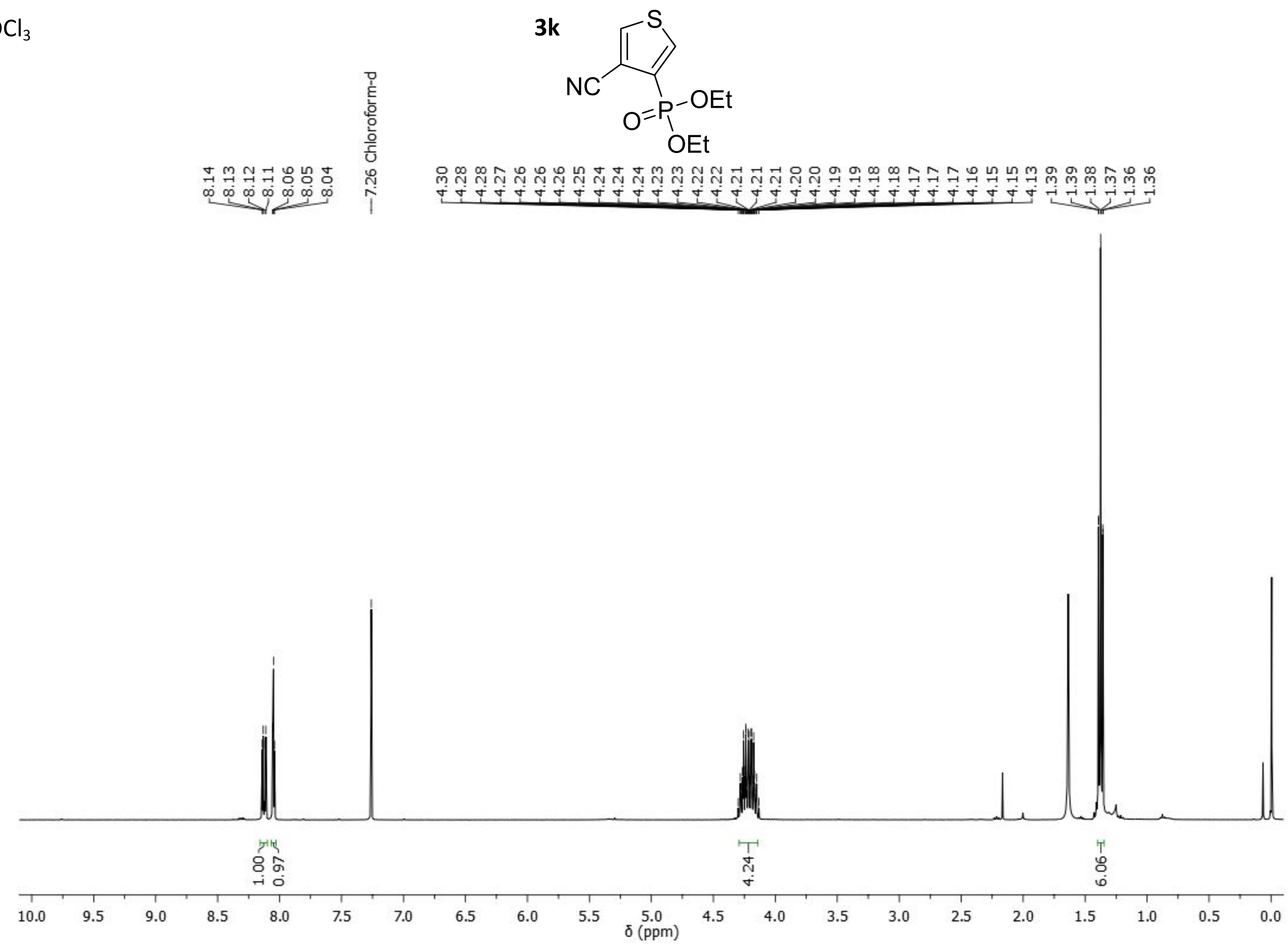

Figure S46. ^{1}H NMR spectrum of **3k.**

^{13}C 101MHz, $CDCl_3$

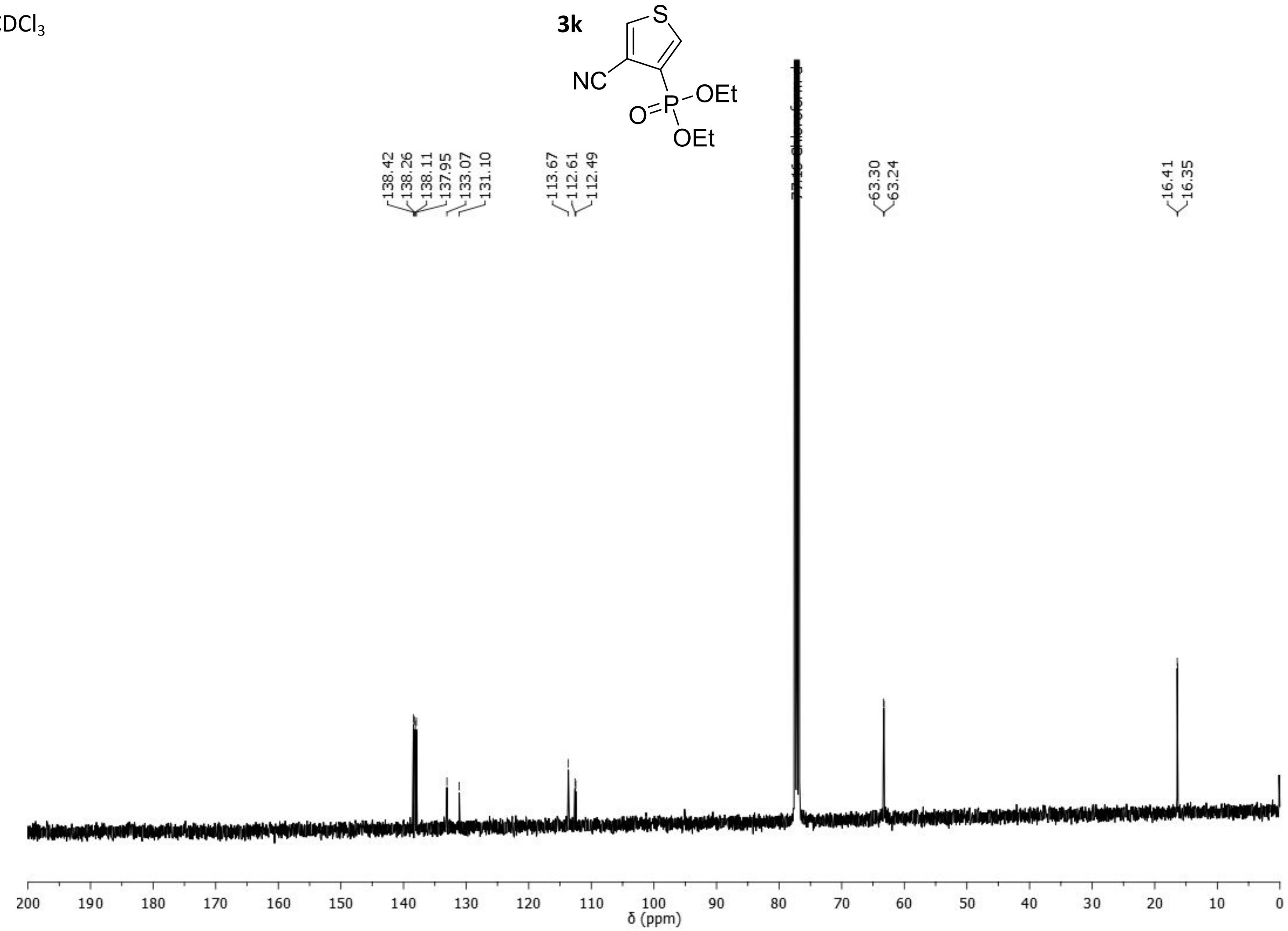

Figure S47. ^{13}C NMR spectrum of **3k.**

^{31}P 162MHz, $CDCl_3$

3k

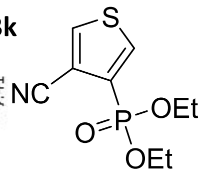

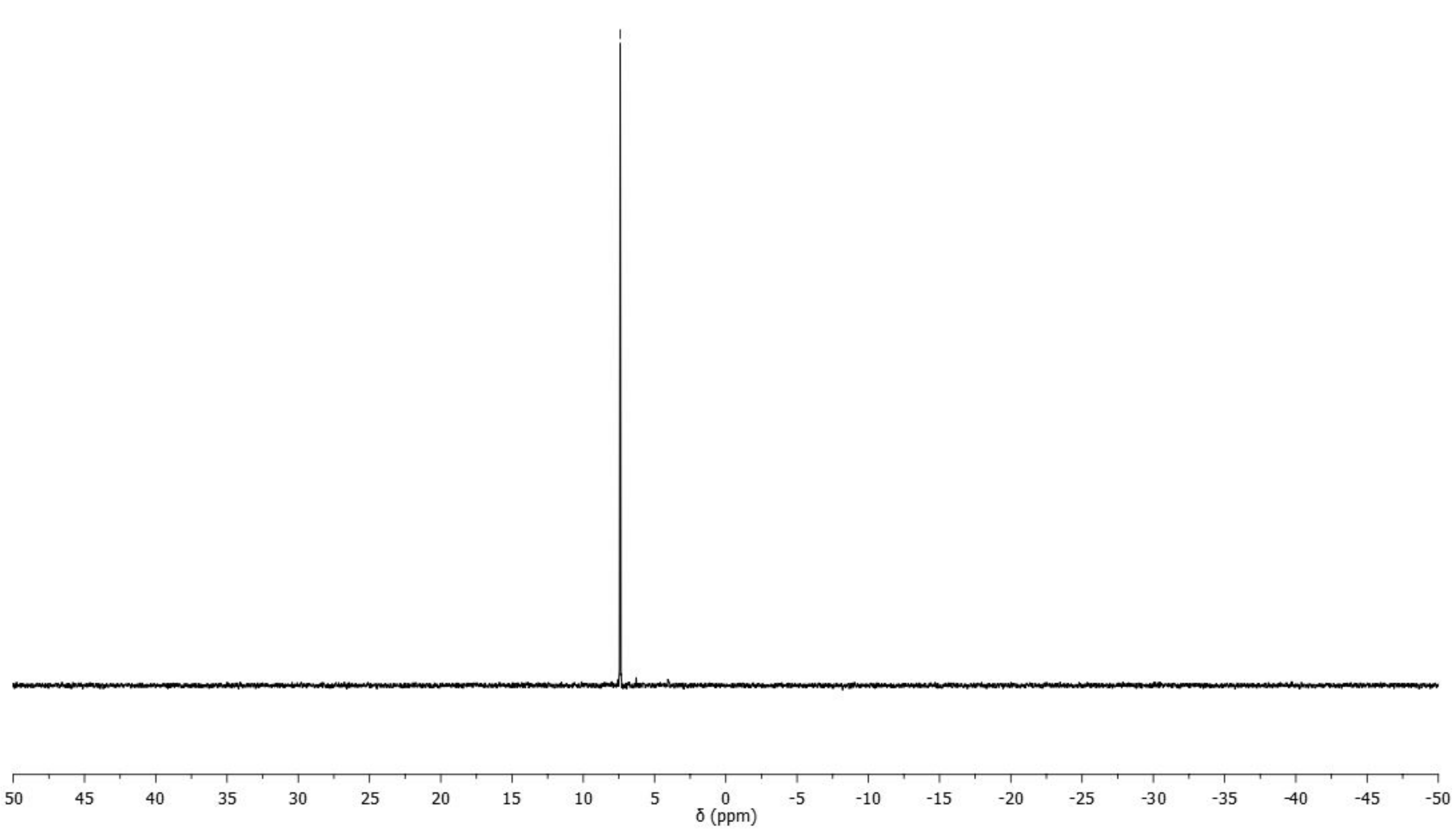

Figure S48. ^{31}P NMR spectrum of **3k.**

^{1}H 400MHz, $CDCl_3$

3l

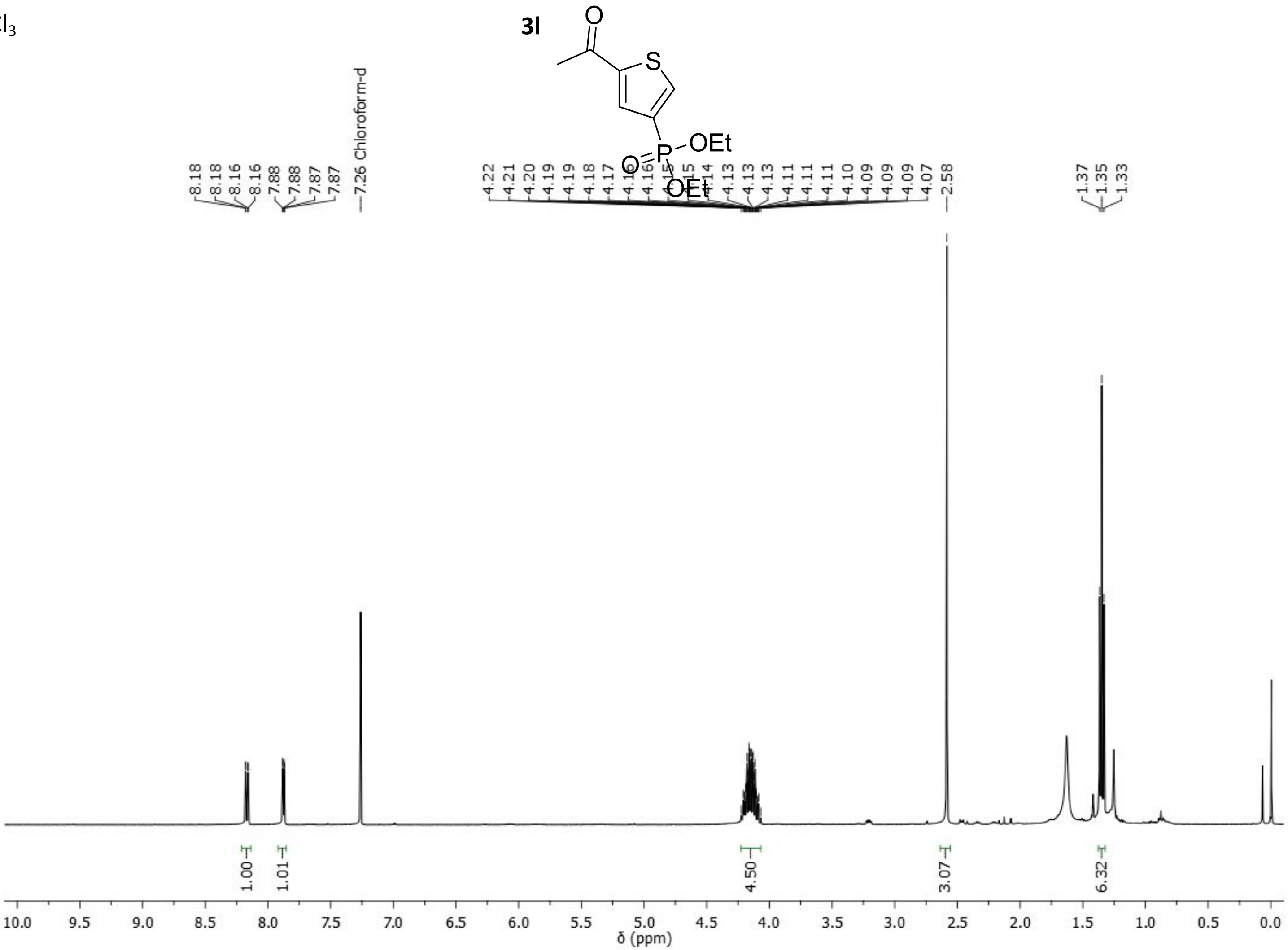

Figure S49. ^{1}H NMR spectrum of **3l.**

^{13}C 101MHz, $CDCl_3$

3l

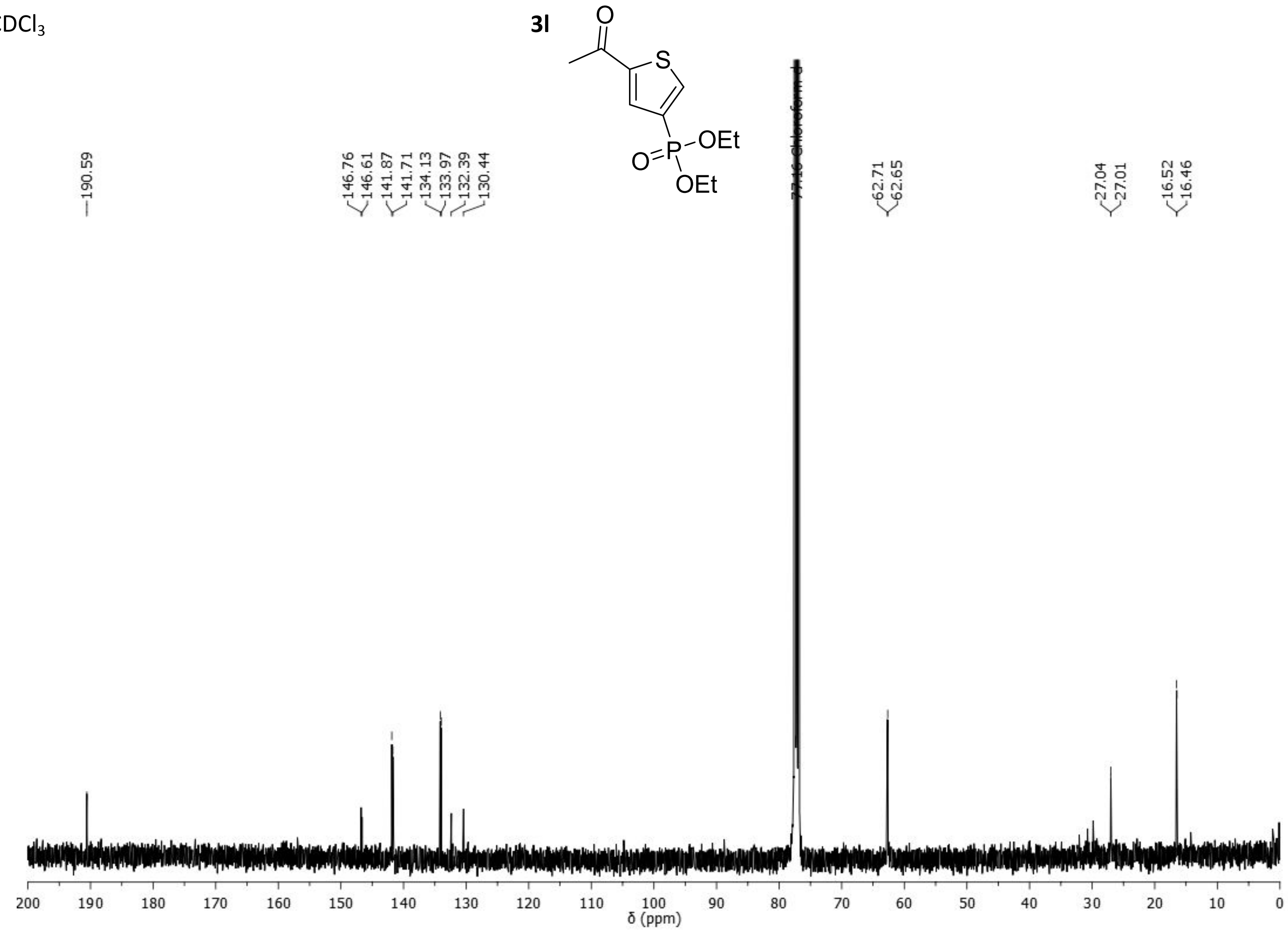

Figure S50. ^{13}C NMR spectrum of **3l.**

^{31}P 162MHz, $CDCl_3$

3l

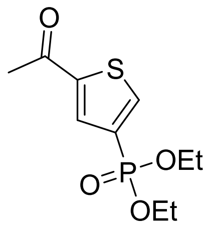

—10.98

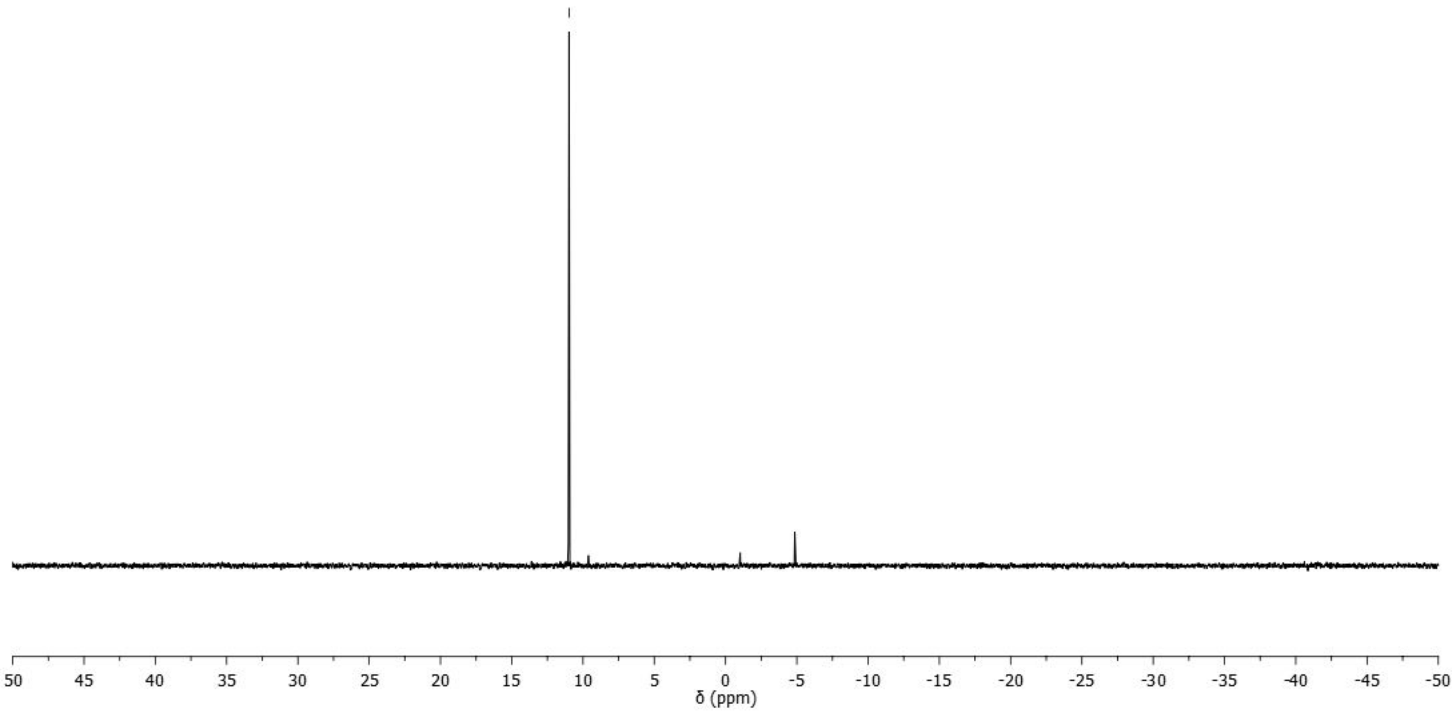

Figure S51. ^{31}P NMR spectrum of **3l.**

^1H 400MHz, $CDCl_3$

3m

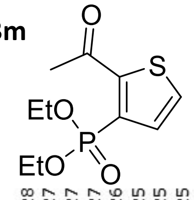

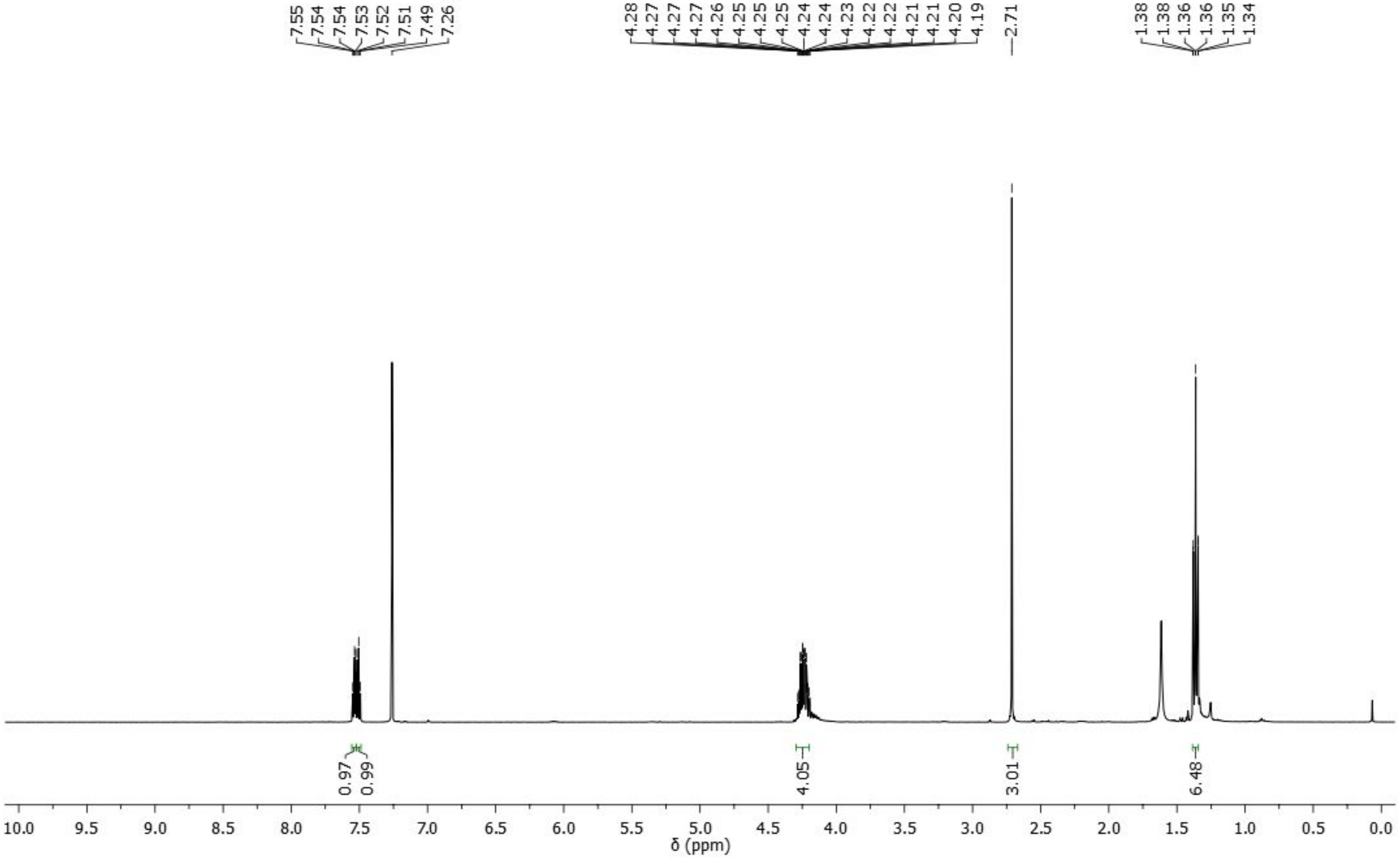

Figure S52. ^1H NMR spectrum of **3m.**

^{13}C 101MHz, $CDCl_3$

3m

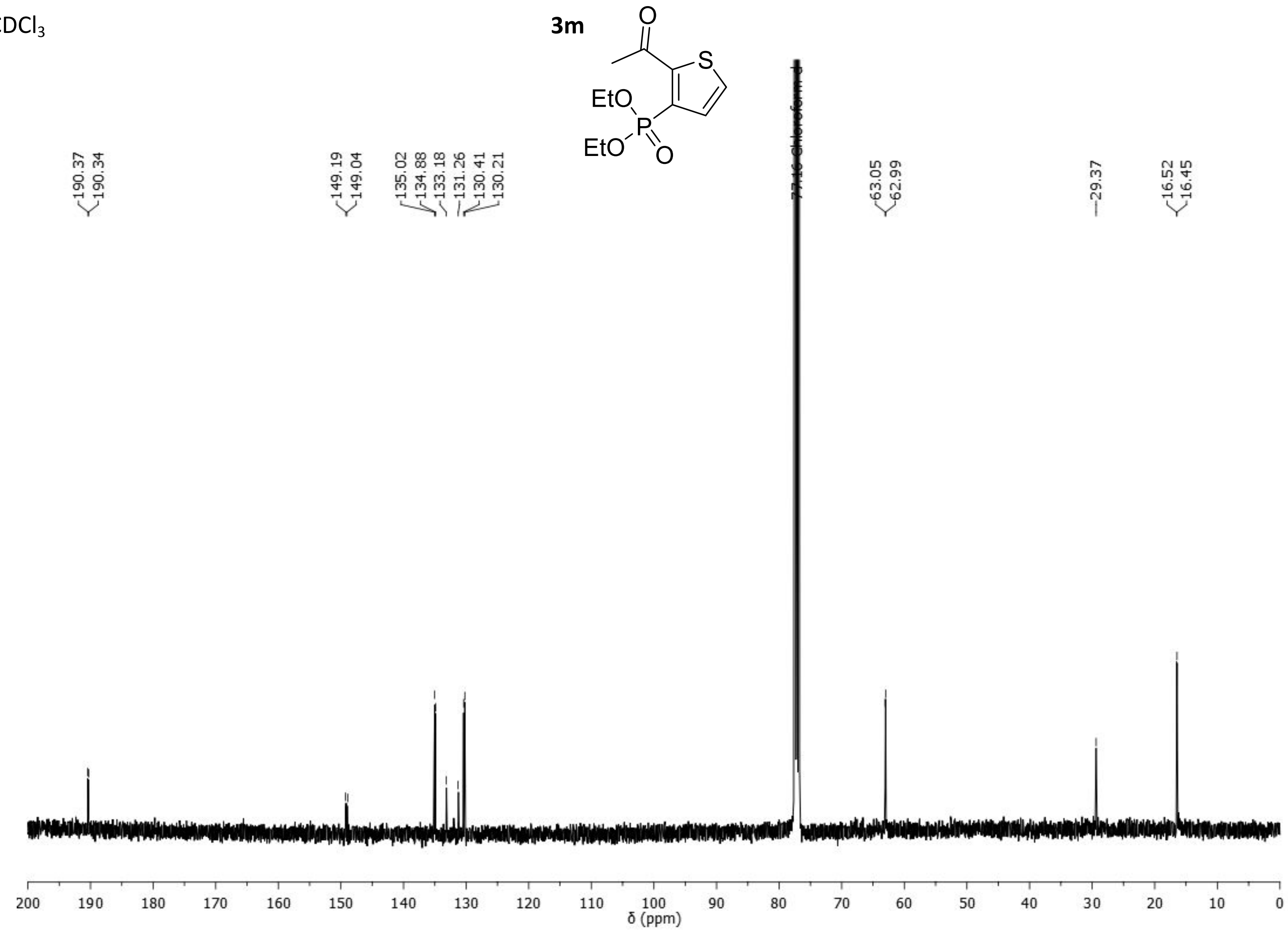

Figure S53. ^{13}C NMR spectrum of **3m.**

^{31}P 162MHz, $CDCl_3$

3m

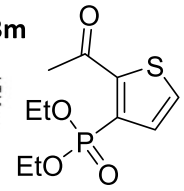

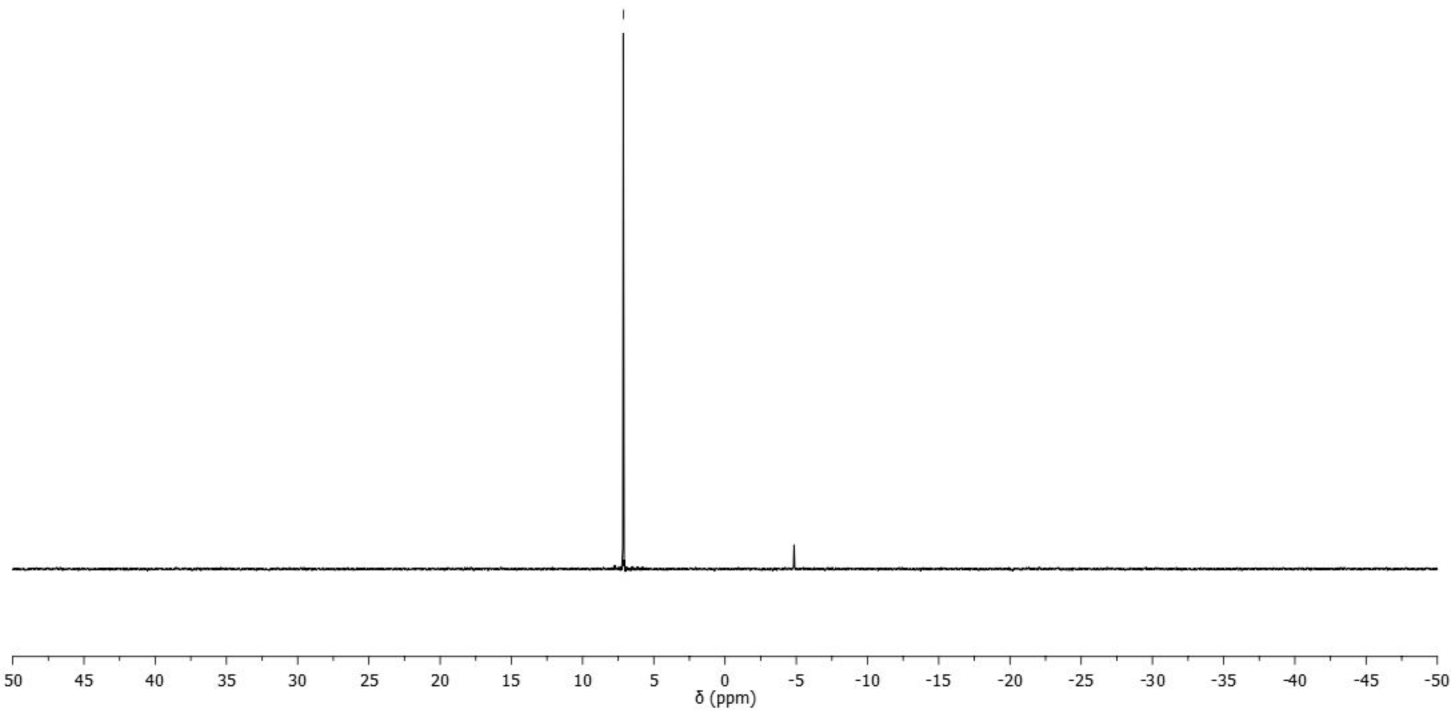

Figure S54. ^{31}P NMR spectrum of **3m.**

^{1}H 400MHz, $CDCl_3$

3n

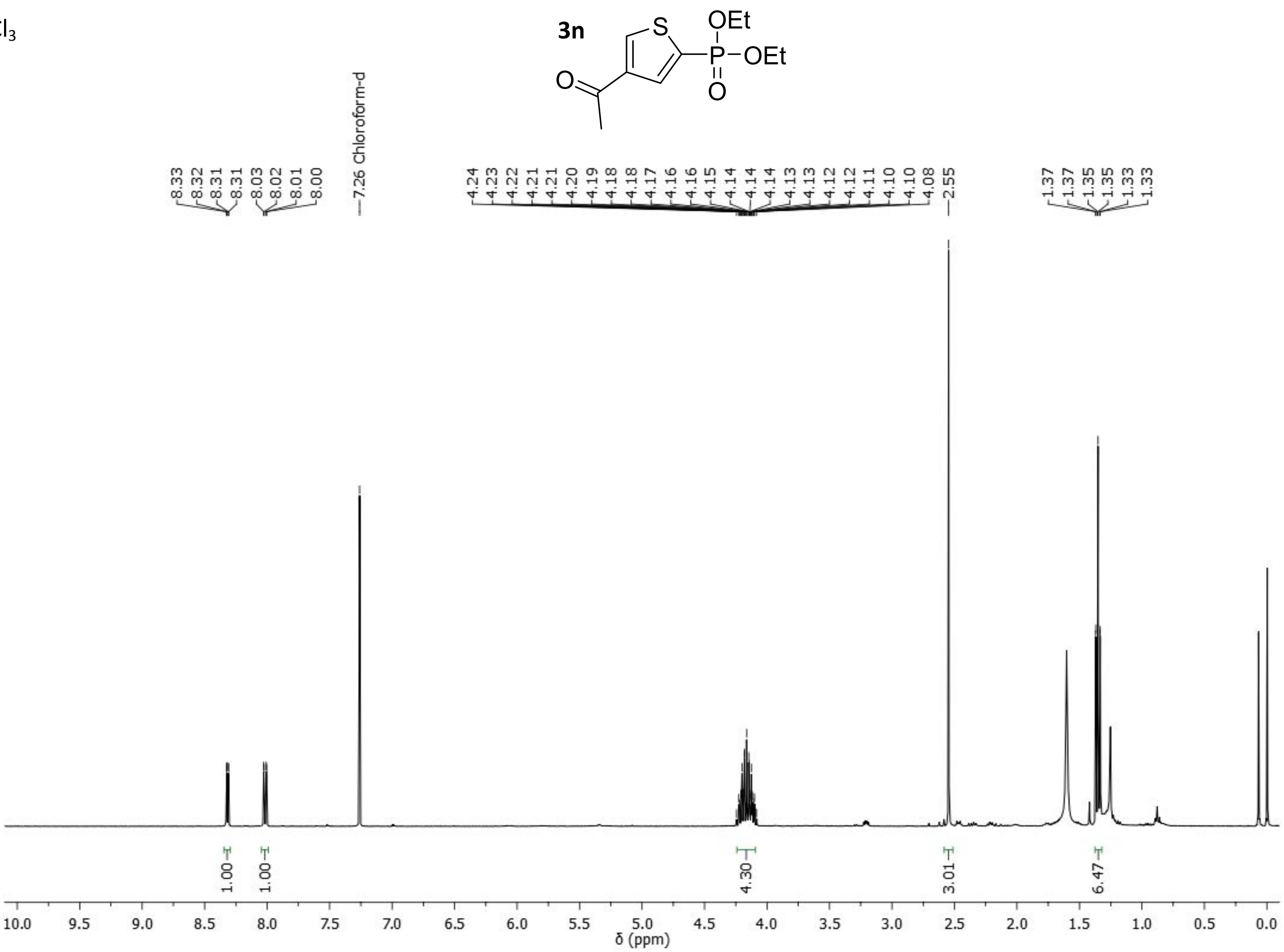

Figure S55. ^{1}H NMR spectrum of **3n.**

^{13}C 101MHz, $CDCl_3$

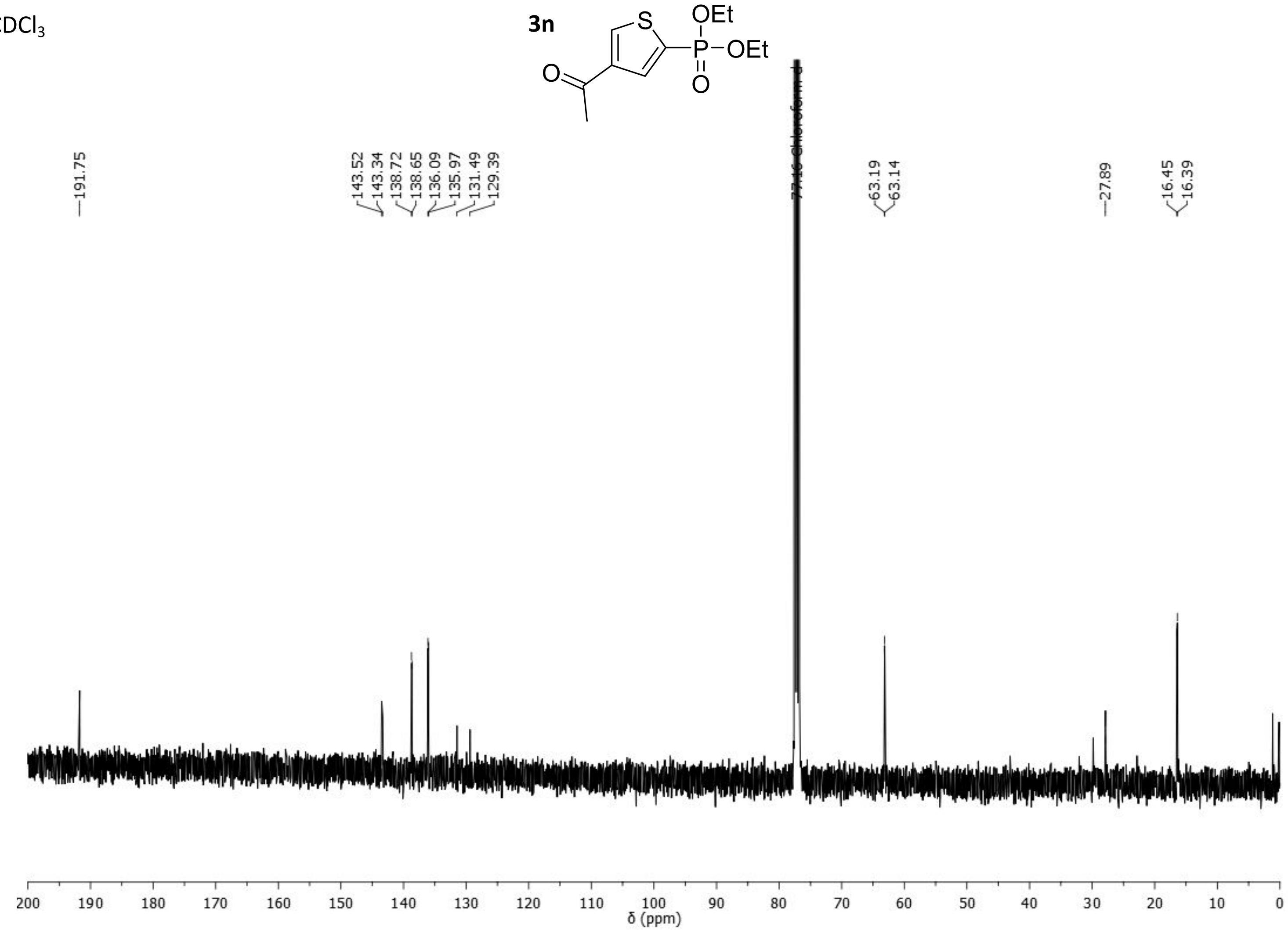

Figure S56. ^{13}C NMR spectrum of **3n.**

^{31}P 162MHz, $CDCl_3$

3n

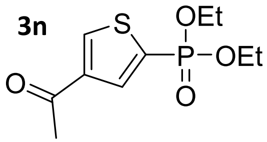

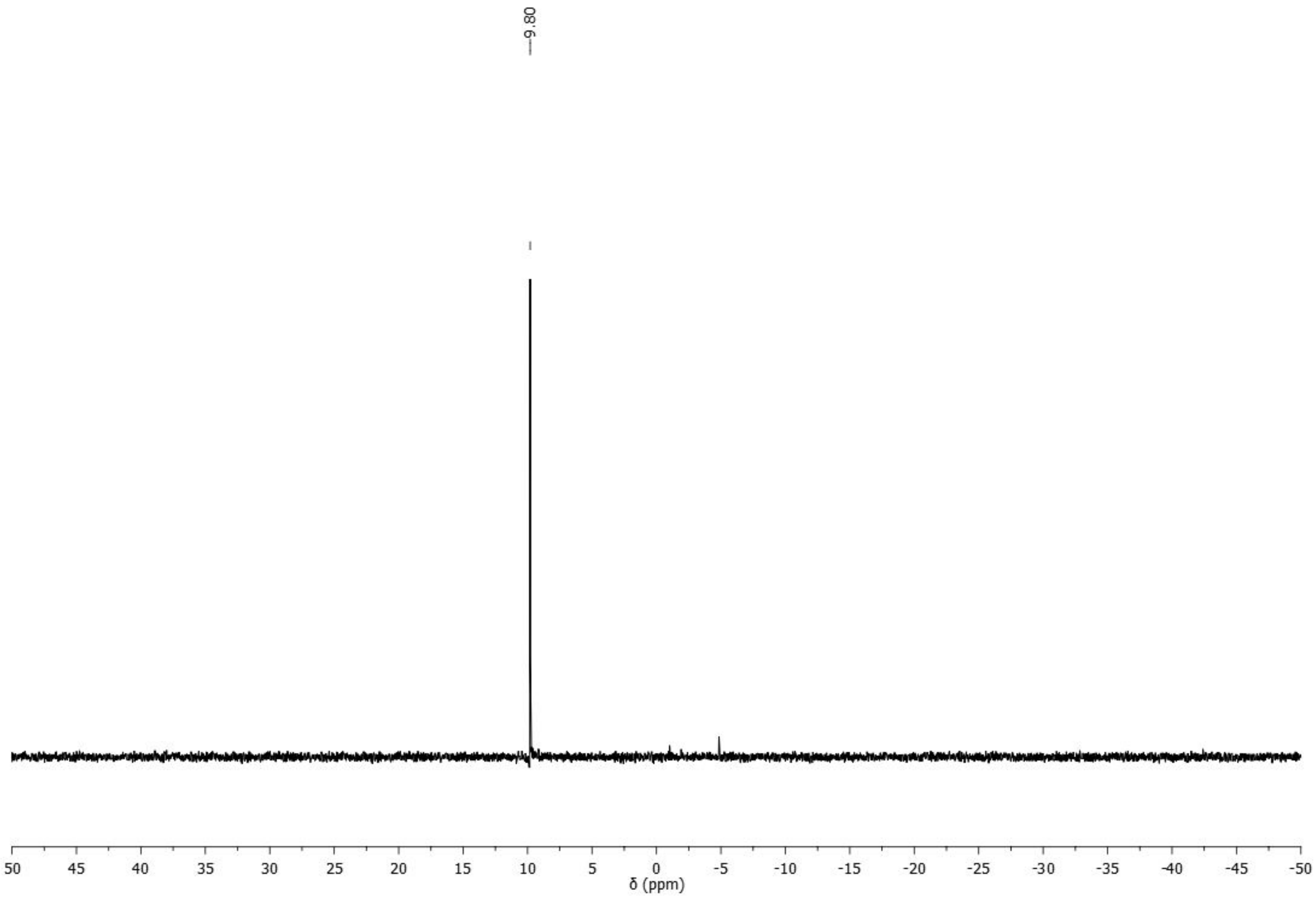

Figure S57. ^{31}P NMR spectrum of **3n.**

^{1}H 400MHz, $CDCl_3$

3o

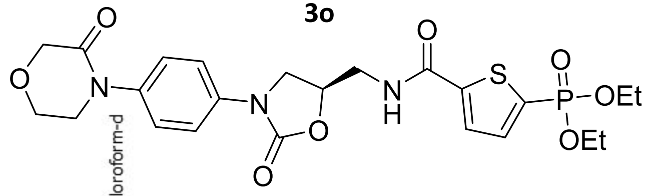

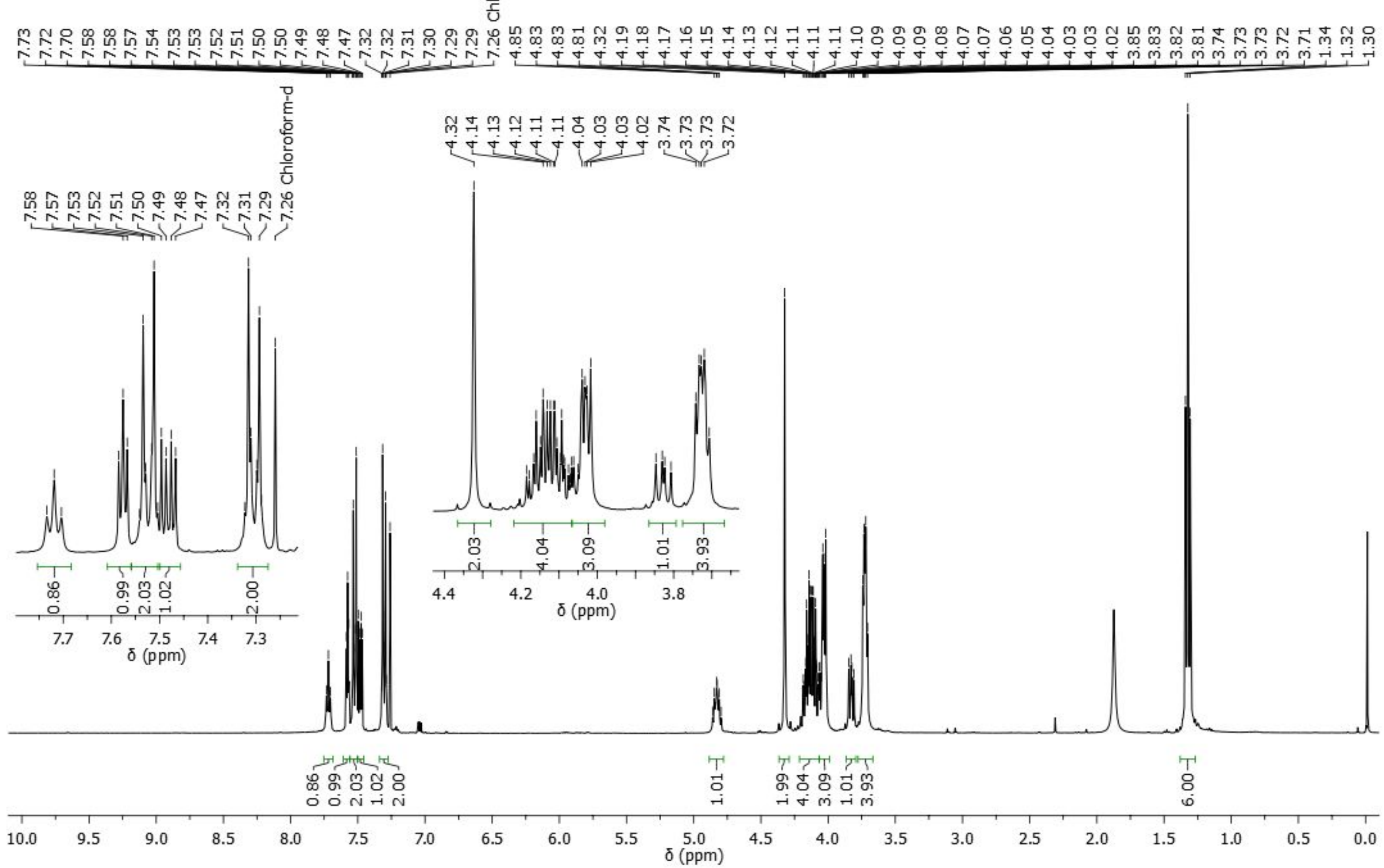

Figure S58. ^{1}H NMR spectrum of **3o.**

^{13}C 101MHz, $CDCl_3$

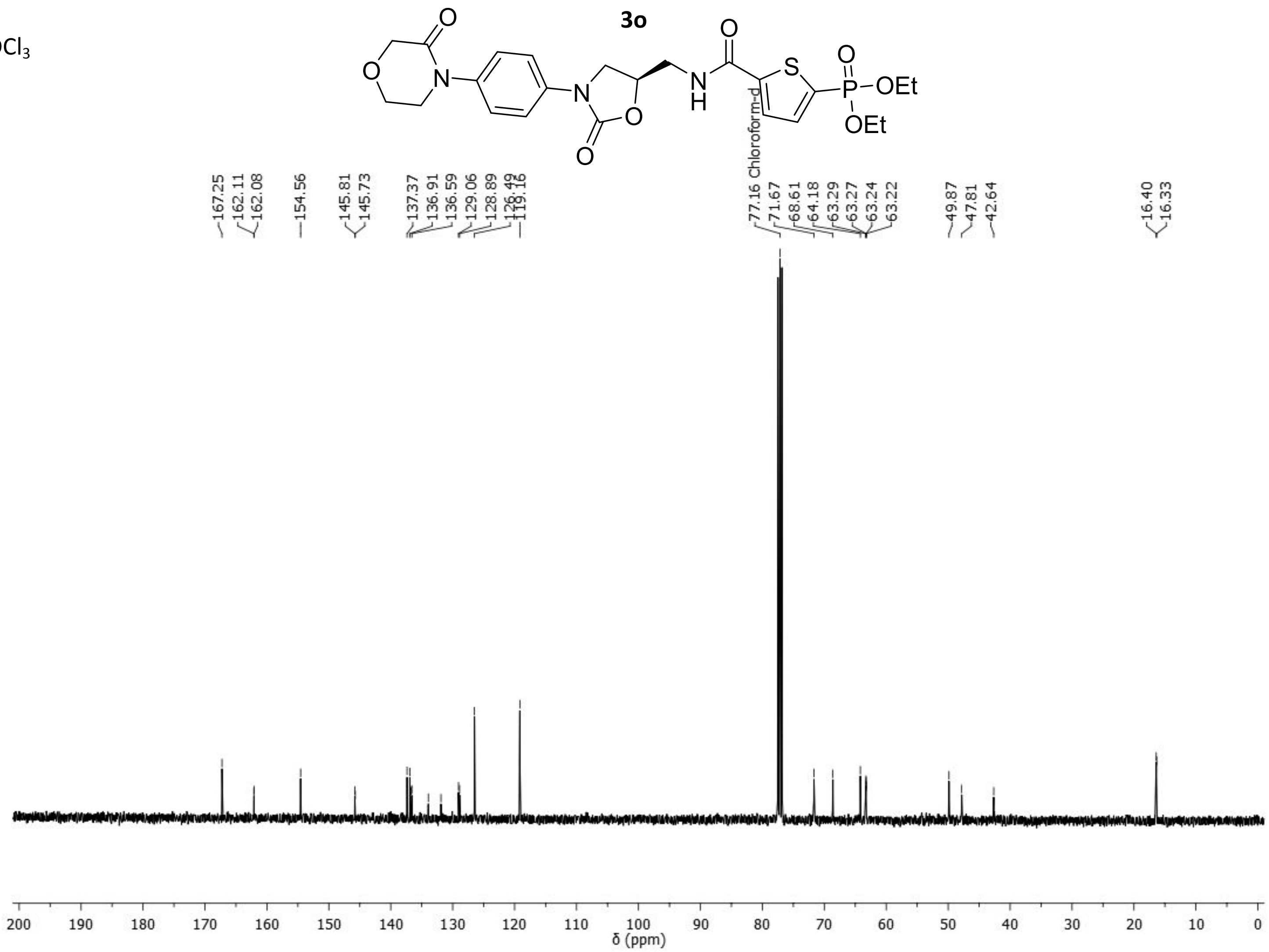

Figure S59. ^{13}C NMR spectrum of **3o.**

^{31}P 162MHz, $CDCl_3$

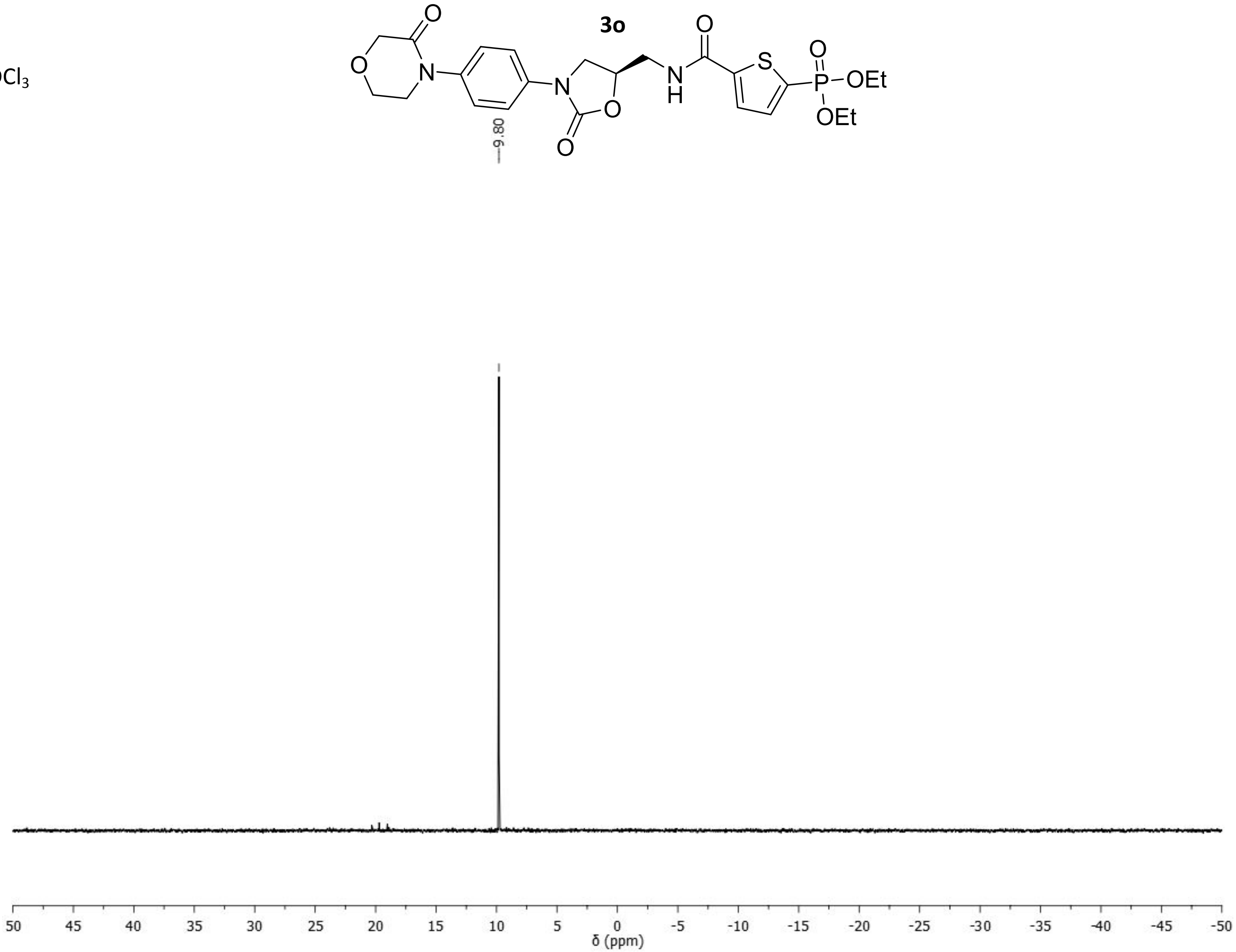

Figure S60. ^{31}P NMR spectrum of **3o.**

^{1}H 400MHz, $CDCl_3$

10a

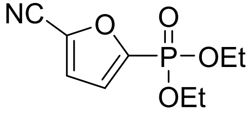

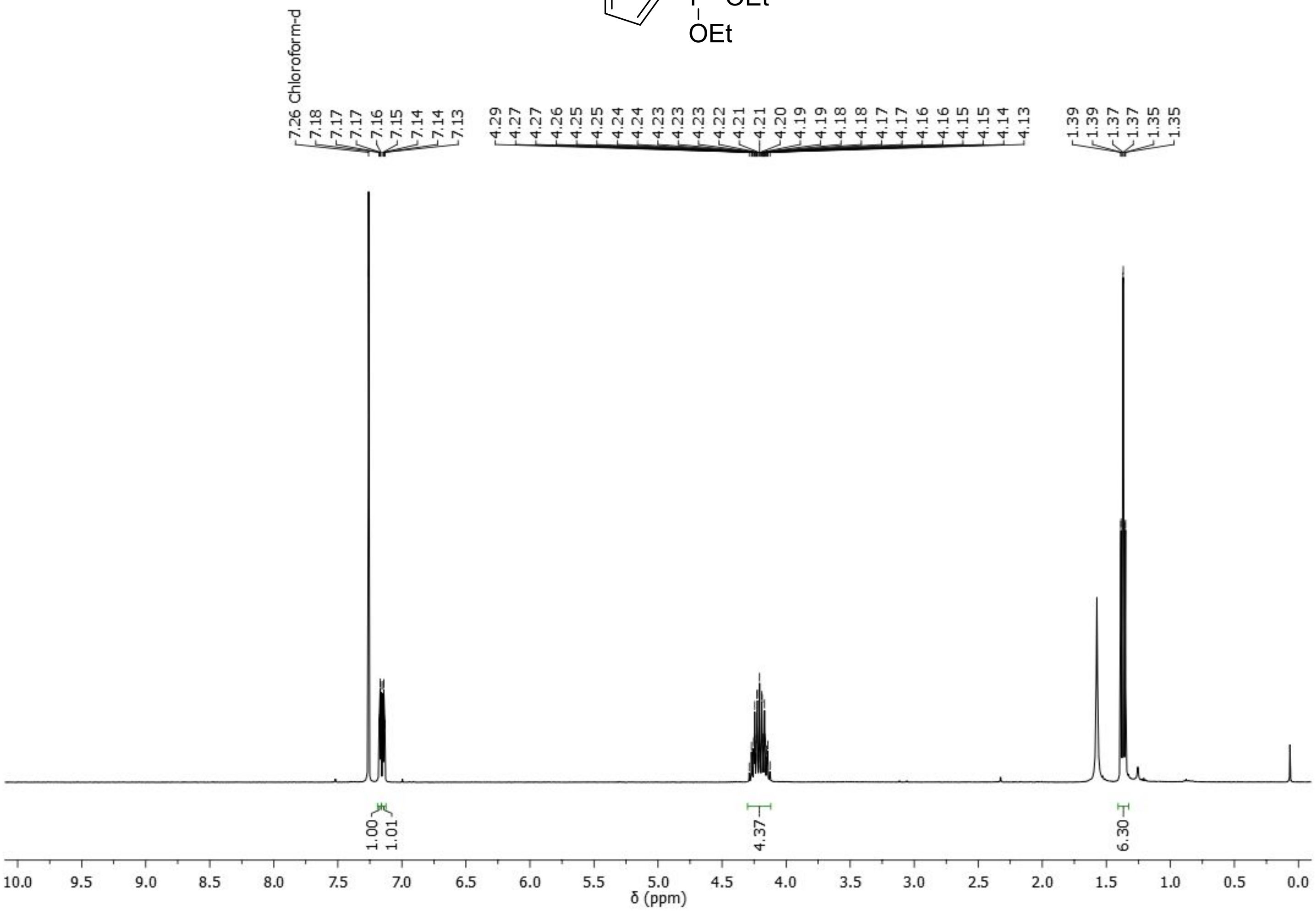

Figure S61. ^{1}H NMR spectrum of **10a.**

^{13}C 101MHz, $CDCl_3$

10a

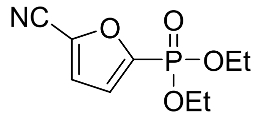

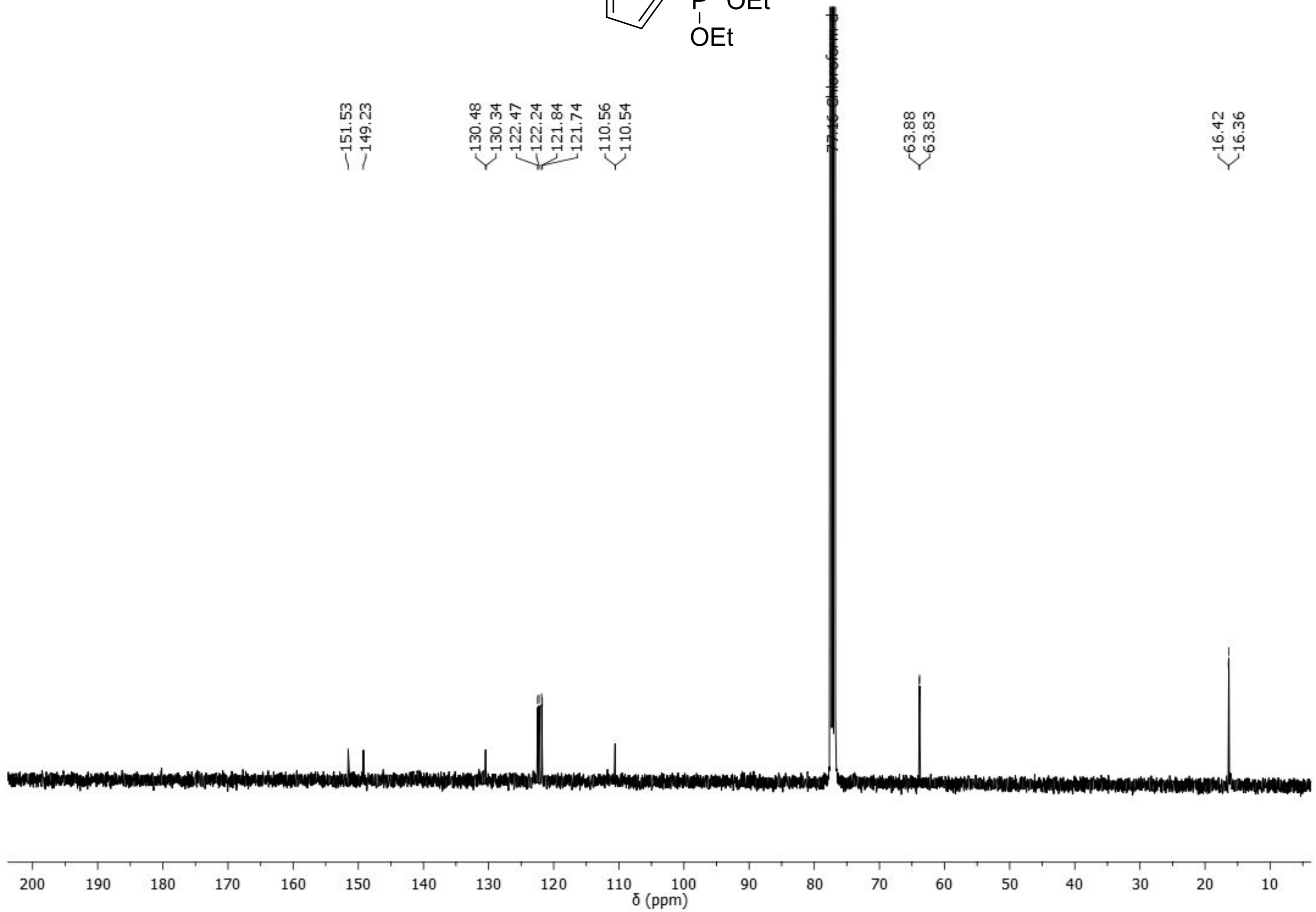

Figure S62. ^{13}C NMR spectrum of **10a.**

^{31}P 162MHz, $CDCl_3$

10a

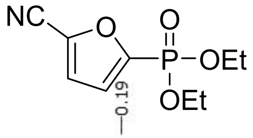

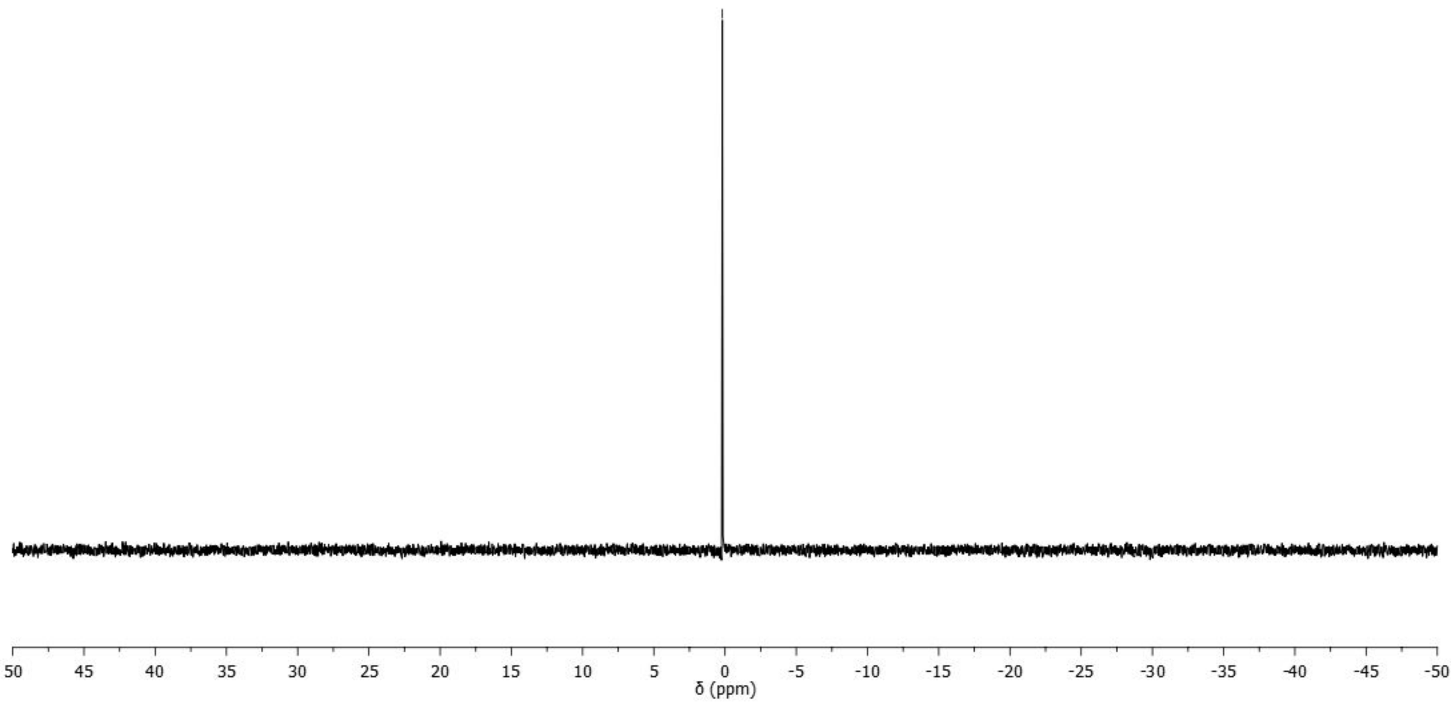

Figure S63. ^{31}P NMR spectrum of **10a.**

^1H 400MHz, $CDCl_3$

10b

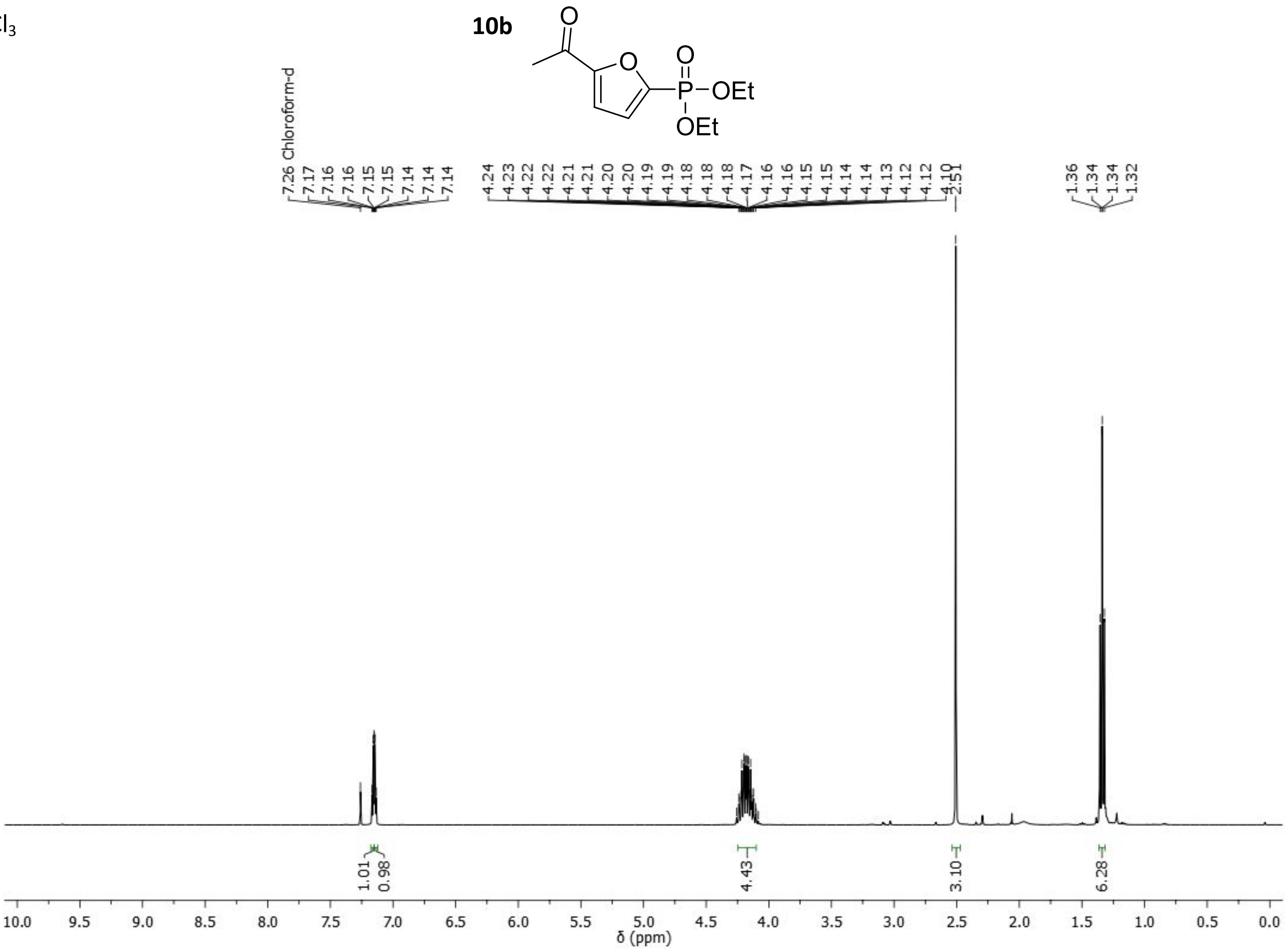

Figure S64. ^1H NMR spectrum of **10b.**

^{13}C 101MHz, $CDCl_3$

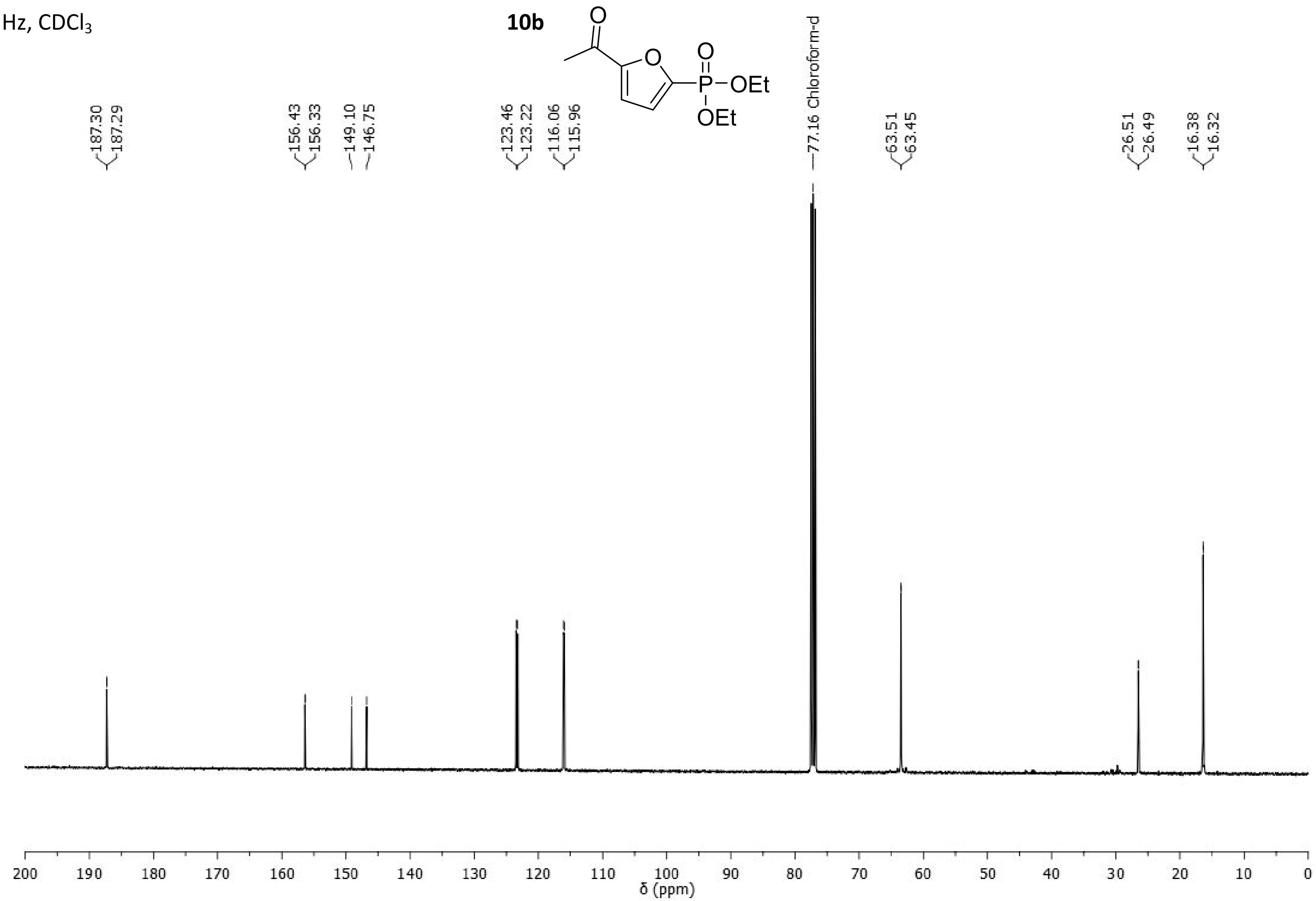

Figure S65. ^{13}C NMR spectrum of **10b.**

^{31}P 162MHz, $CDCl_3$

10b

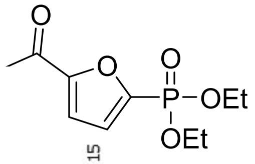

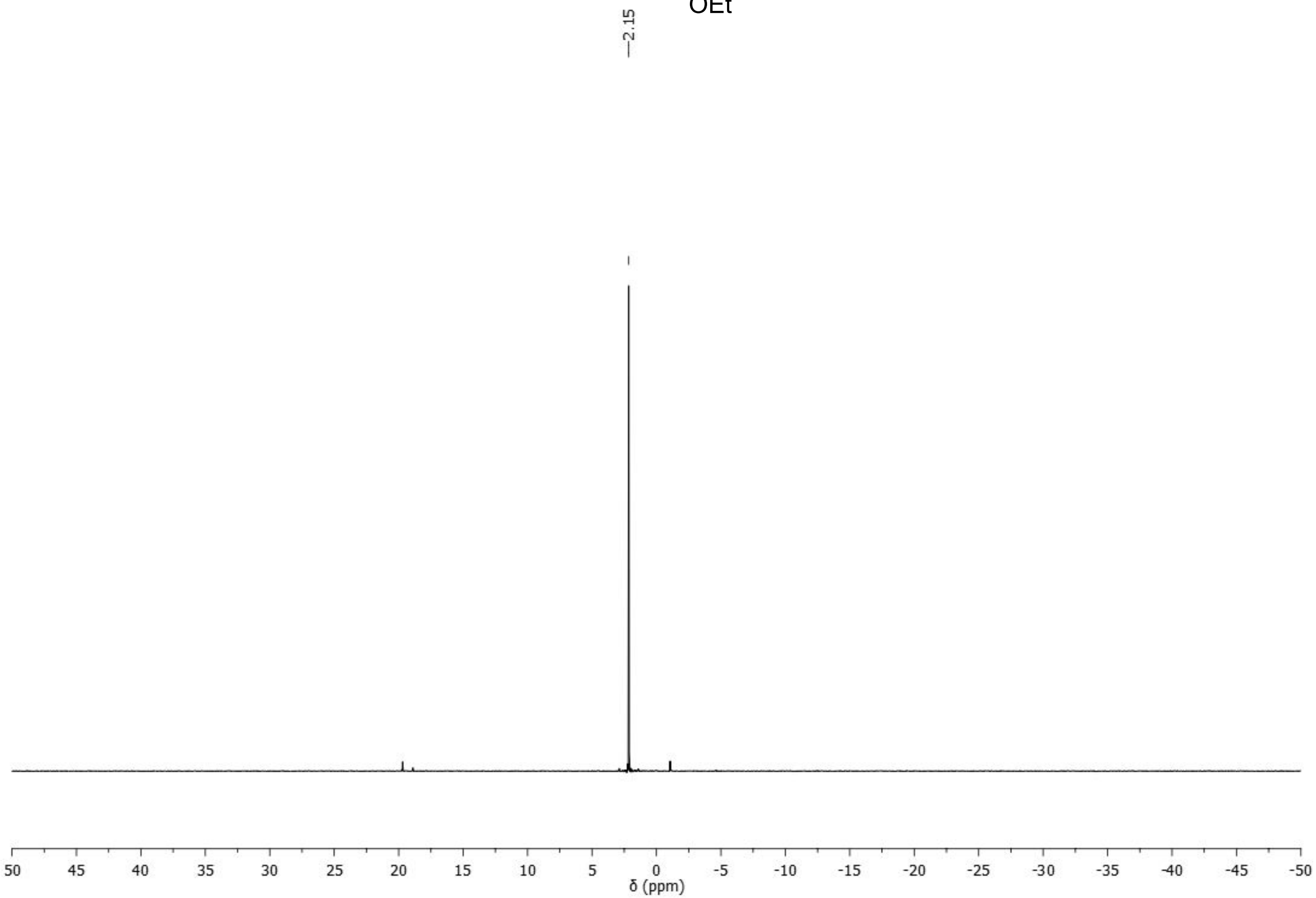

Figure S66. ^{31}P NMR spectrum of **10b.**

^1H 400MHz, $CDCl_3$

10c

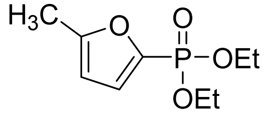

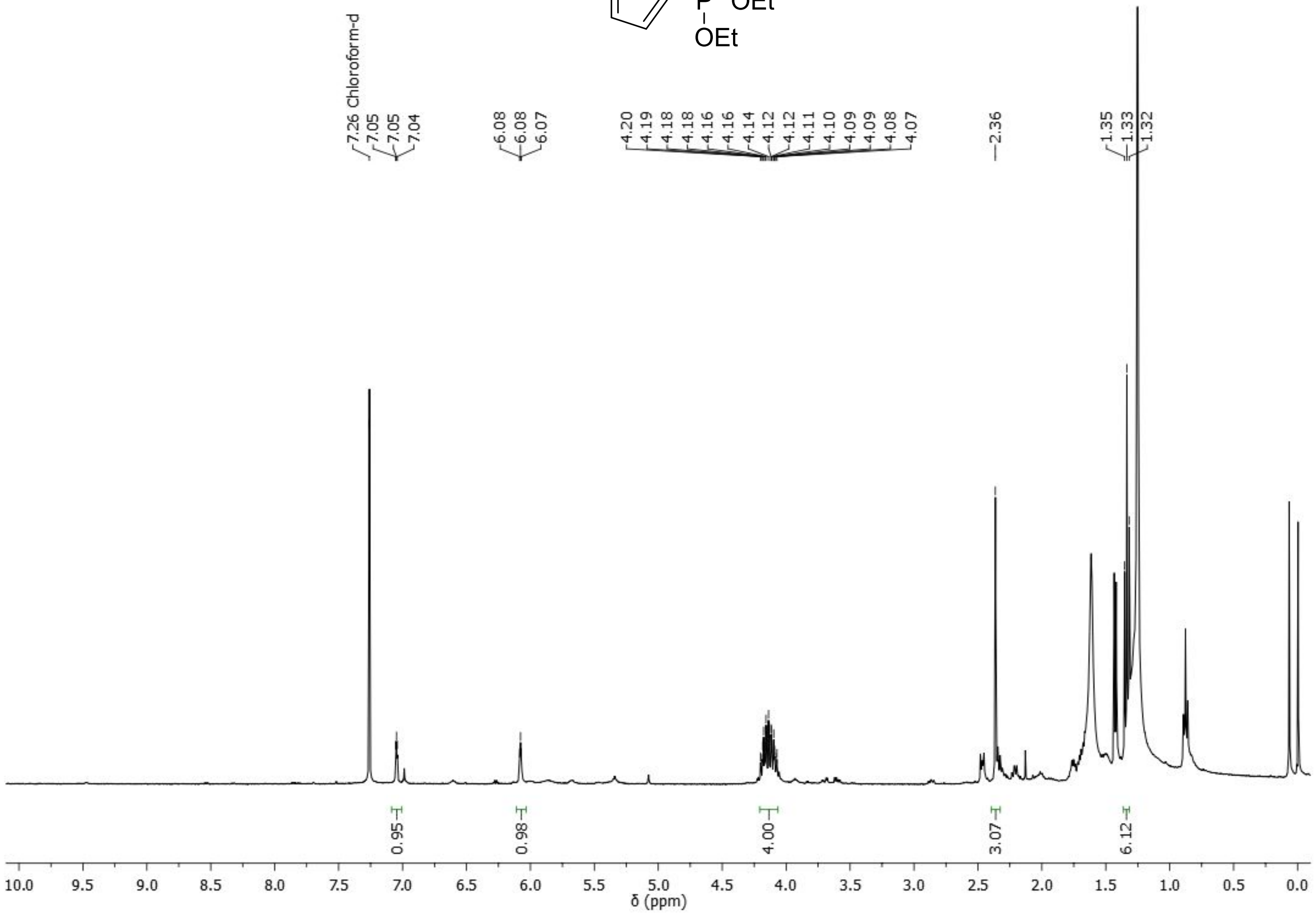

Figure S67. ^1H NMR spectrum of **10c.**

^{13}C 101MHz, $CDCl_3$

10c

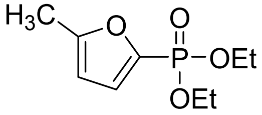

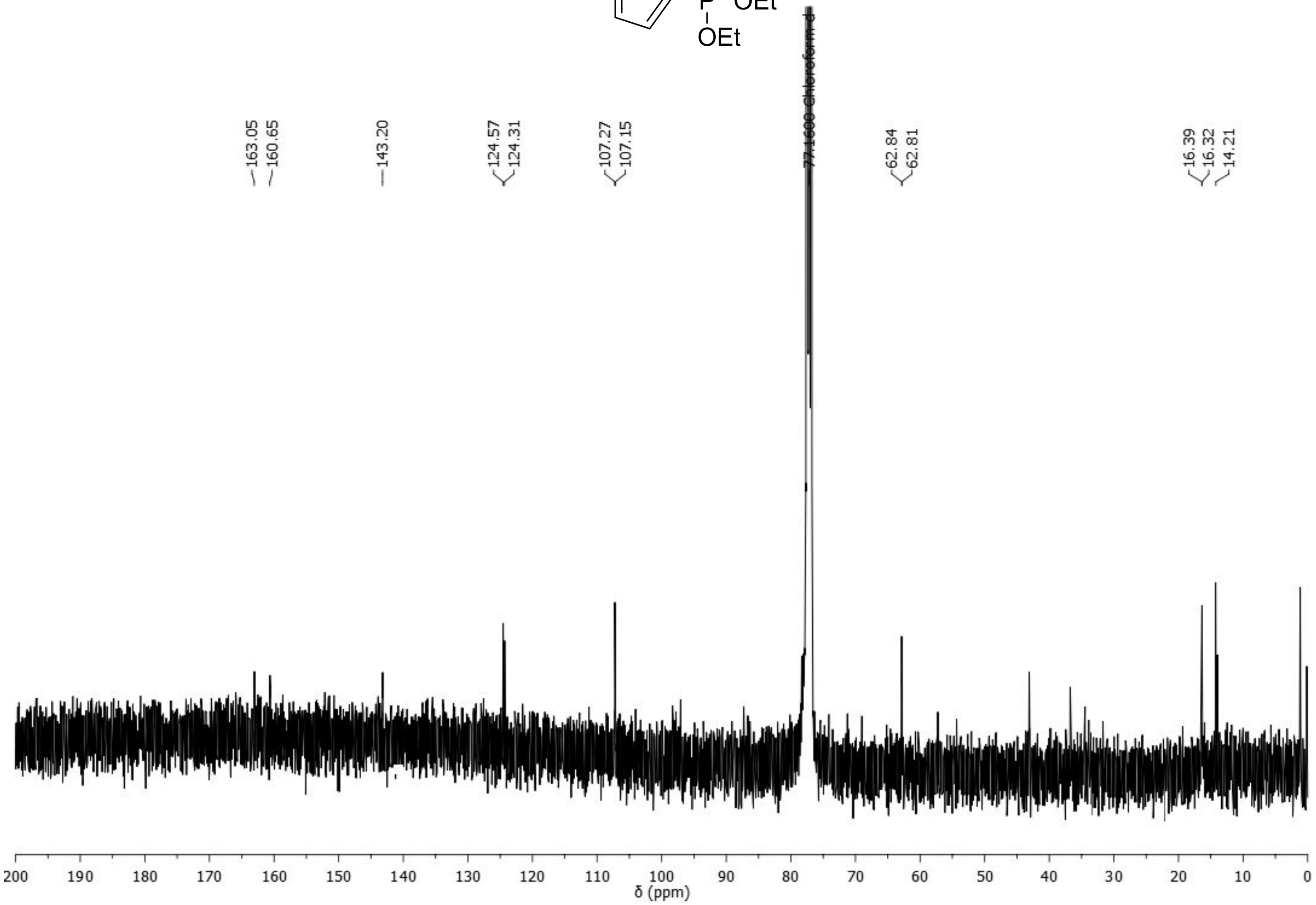

Figure S68. ^{13}C NMR spectrum of **10c.**

^{31}P 162MHz, $CDCl_3$

10c

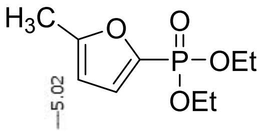

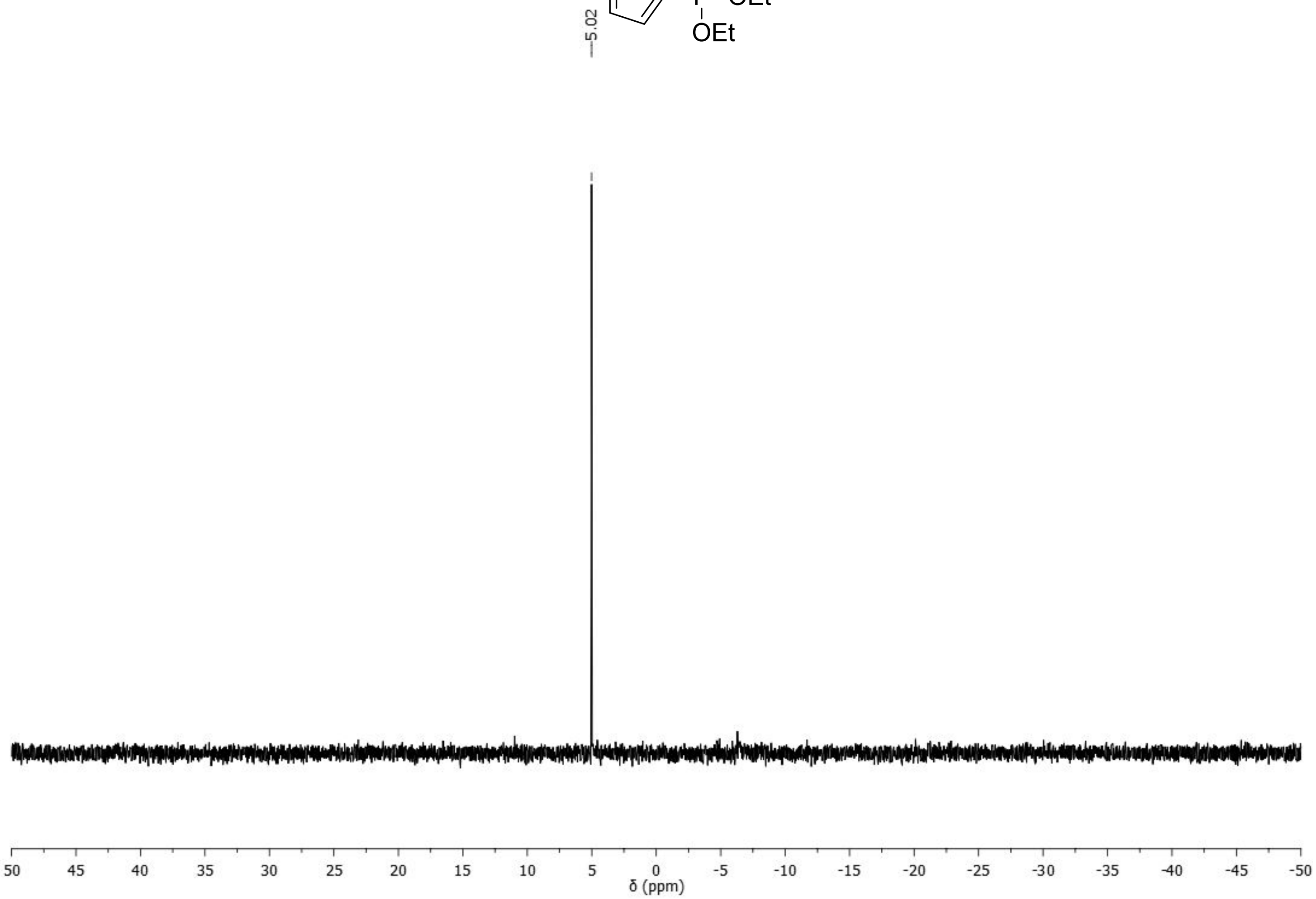

Figure S69. ^{31}P NMR spectrum of **10c.**

^1H 400MHz, $CDCl_3$

11a

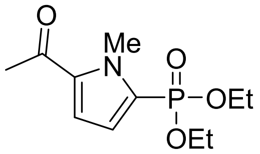

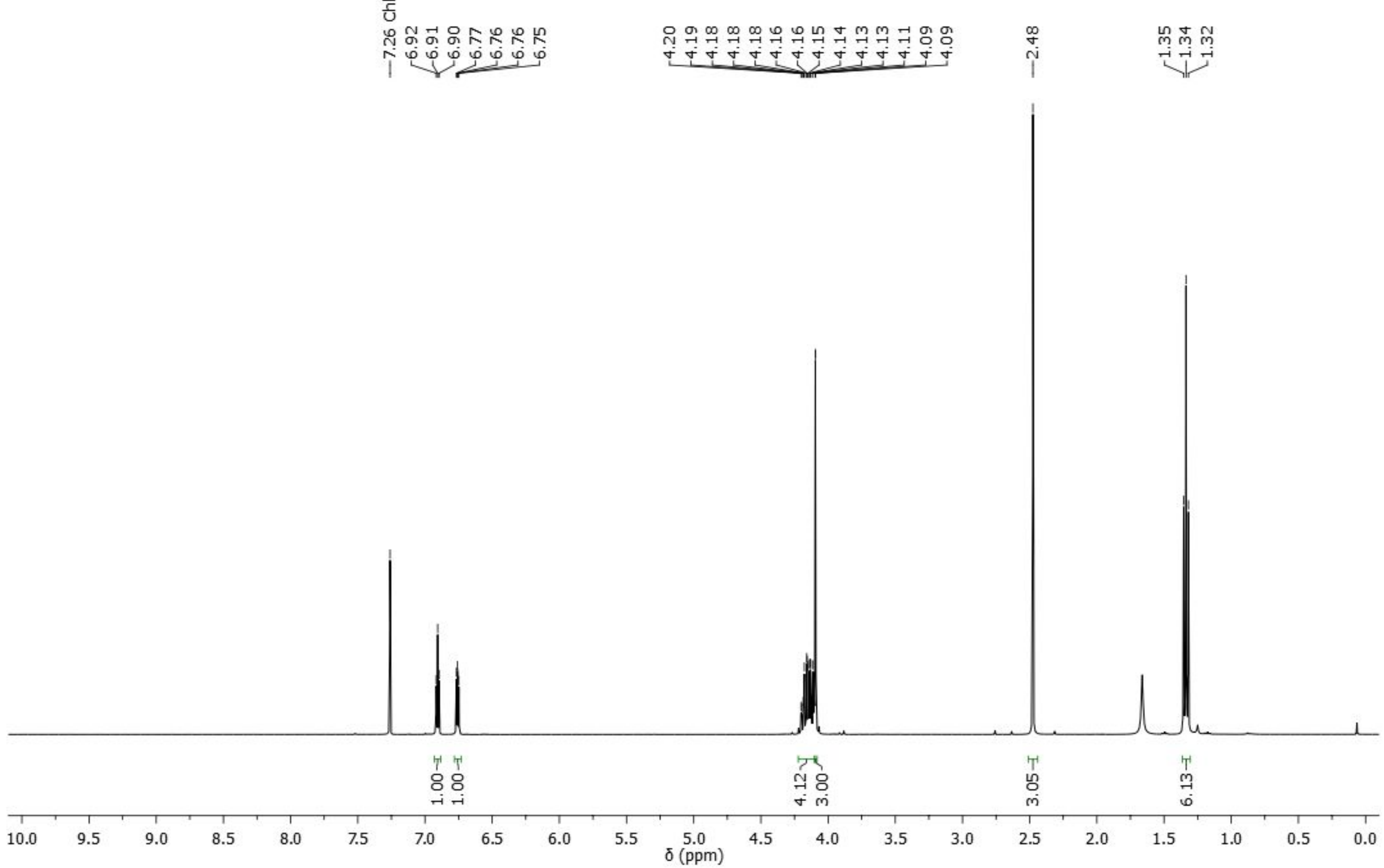

Figure S70. ^1H NMR spectrum of **11a.**

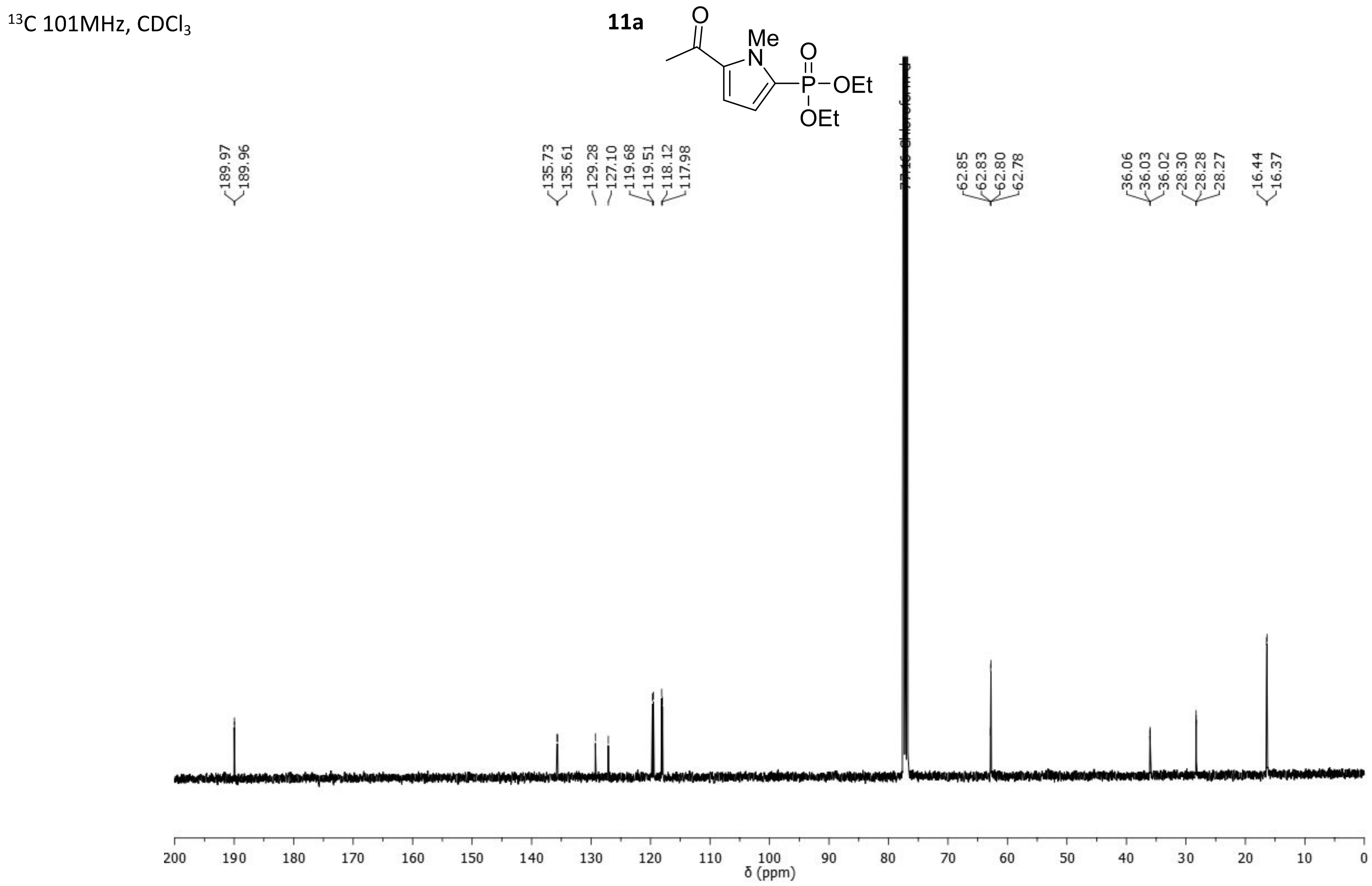

Figure S71. ^{13}C NMR spectrum of **11a.**

^{31}P 162MHz, $CDCl_3$

11a

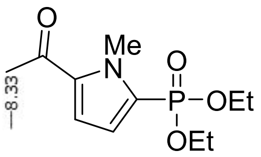

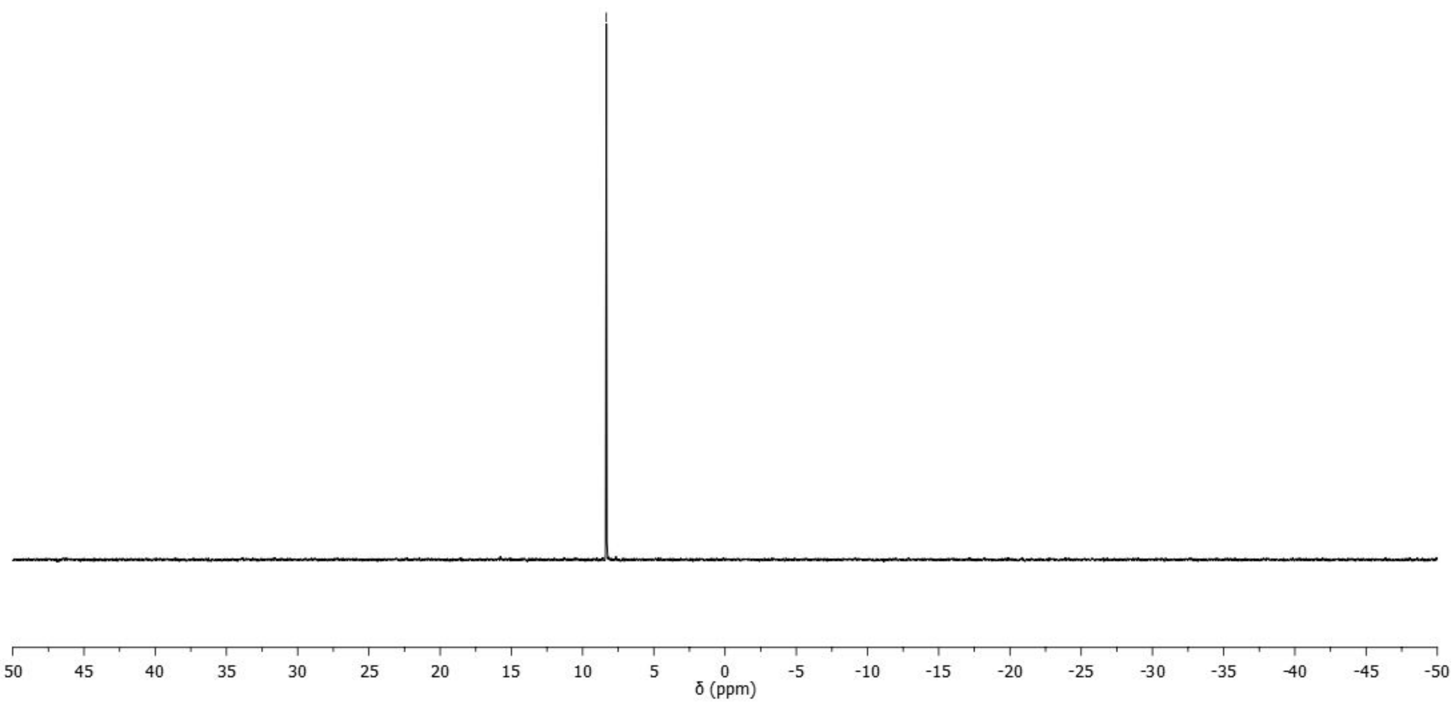

Figure S72. ^{31}P NMR spectrum of **11a.**

^{1}H 400MHz, $CDCl_3$

12a

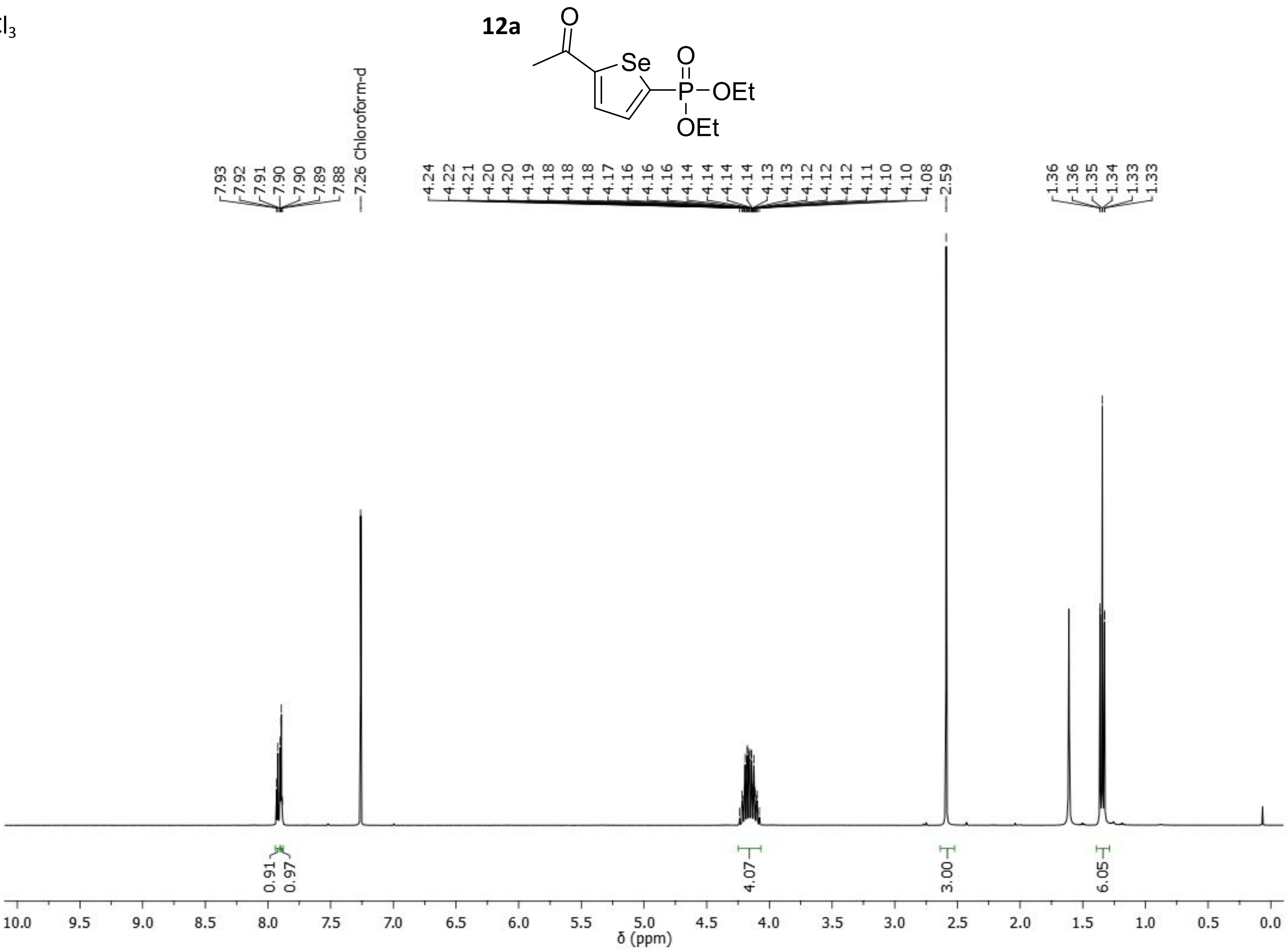

Figure S73. ^{1}H NMR spectrum of **12a.**

^{13}C 101MHz, $CDCl_3$

12a

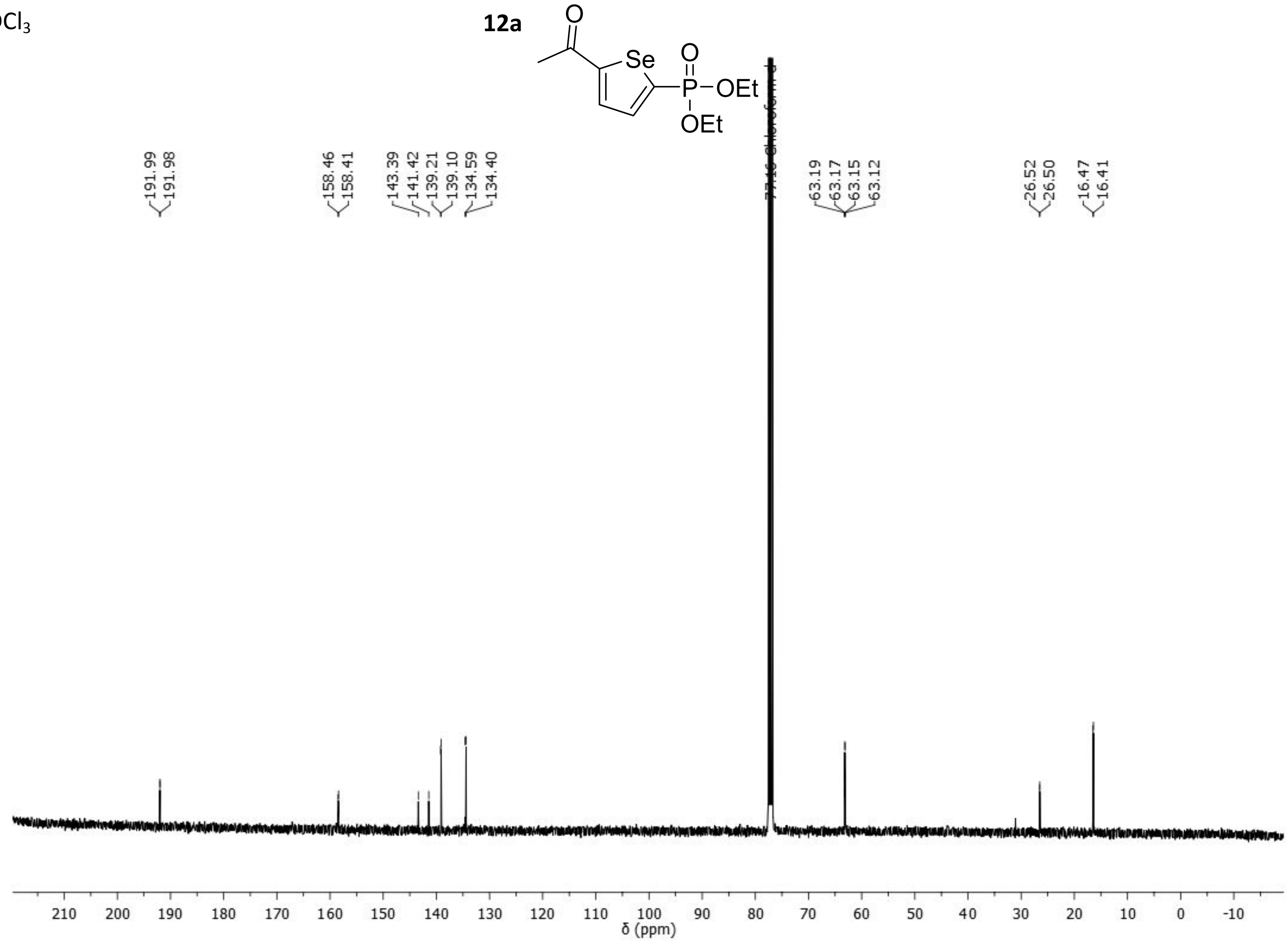

Figure S74. ^{13}C NMR spectrum of **12a.**

^{31}P 162MHz, $CDCl_3$

12a

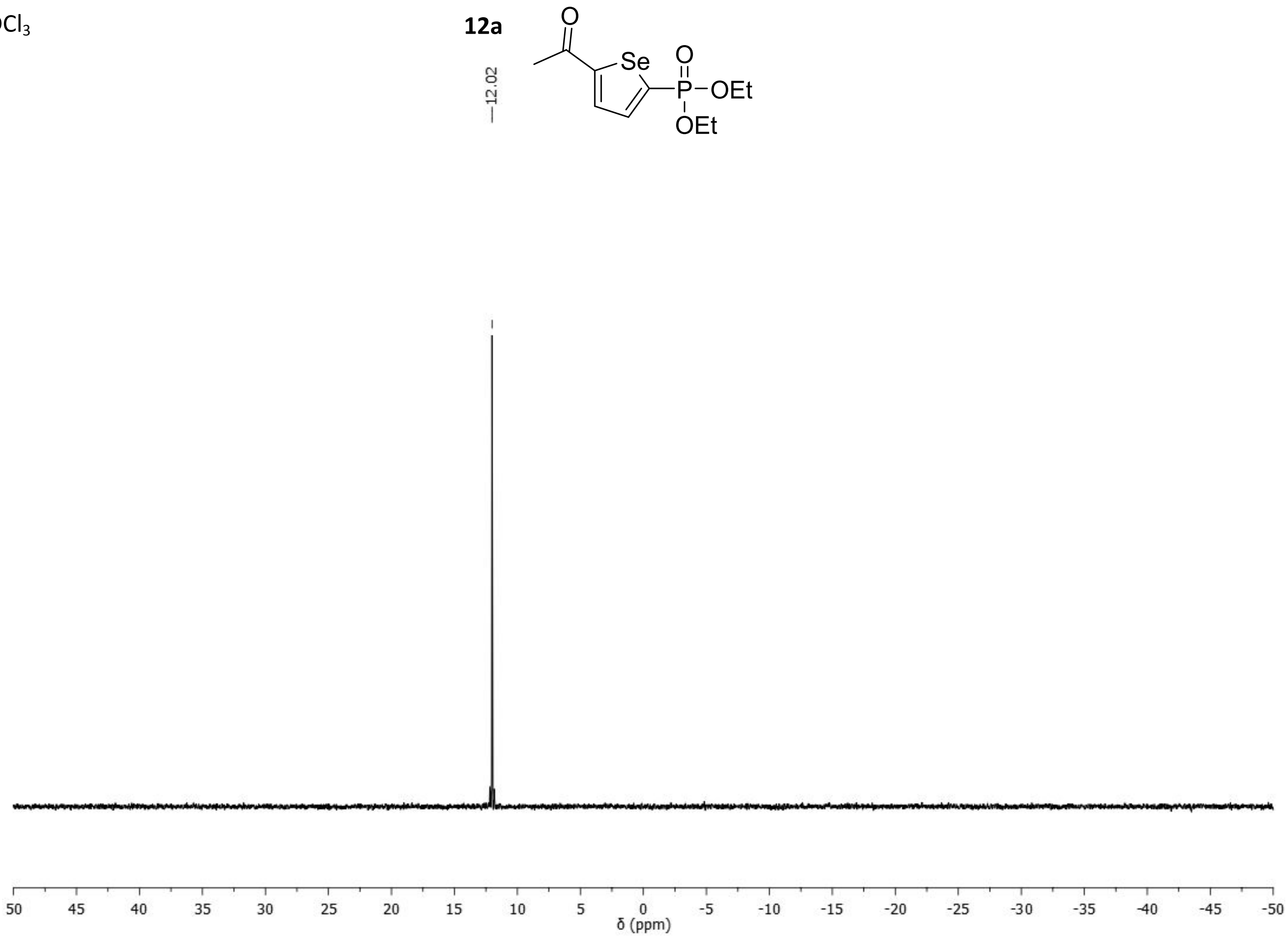

Figure S75. ^{31}P NMR spectrum of **12a.**

^{1}H 400MHz, $CDCl_3$

13a

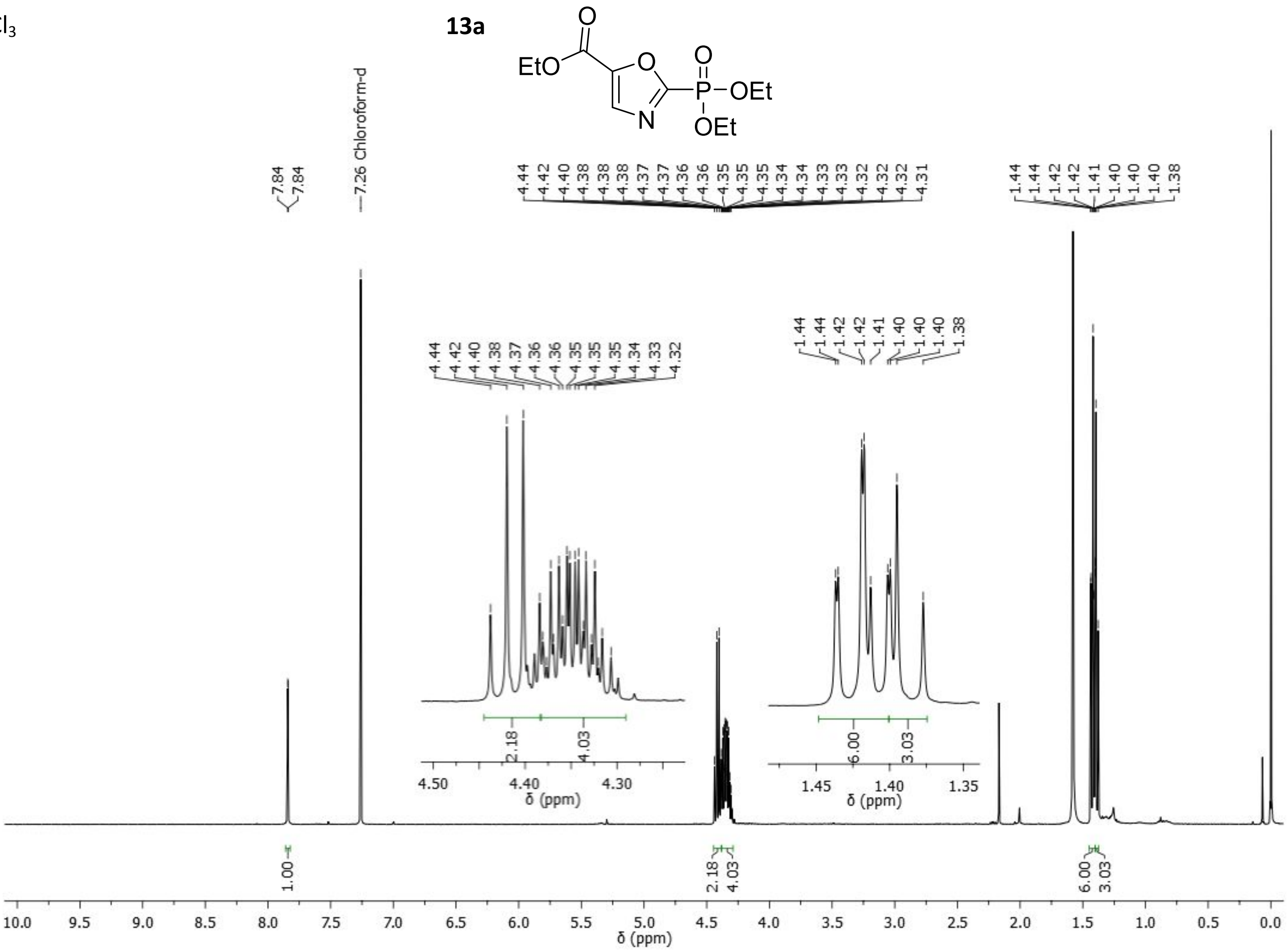

Figure S76. ^{1}H NMR spectrum of **13a.**

^{13}C 101MHz, $CDCl_3$

13a

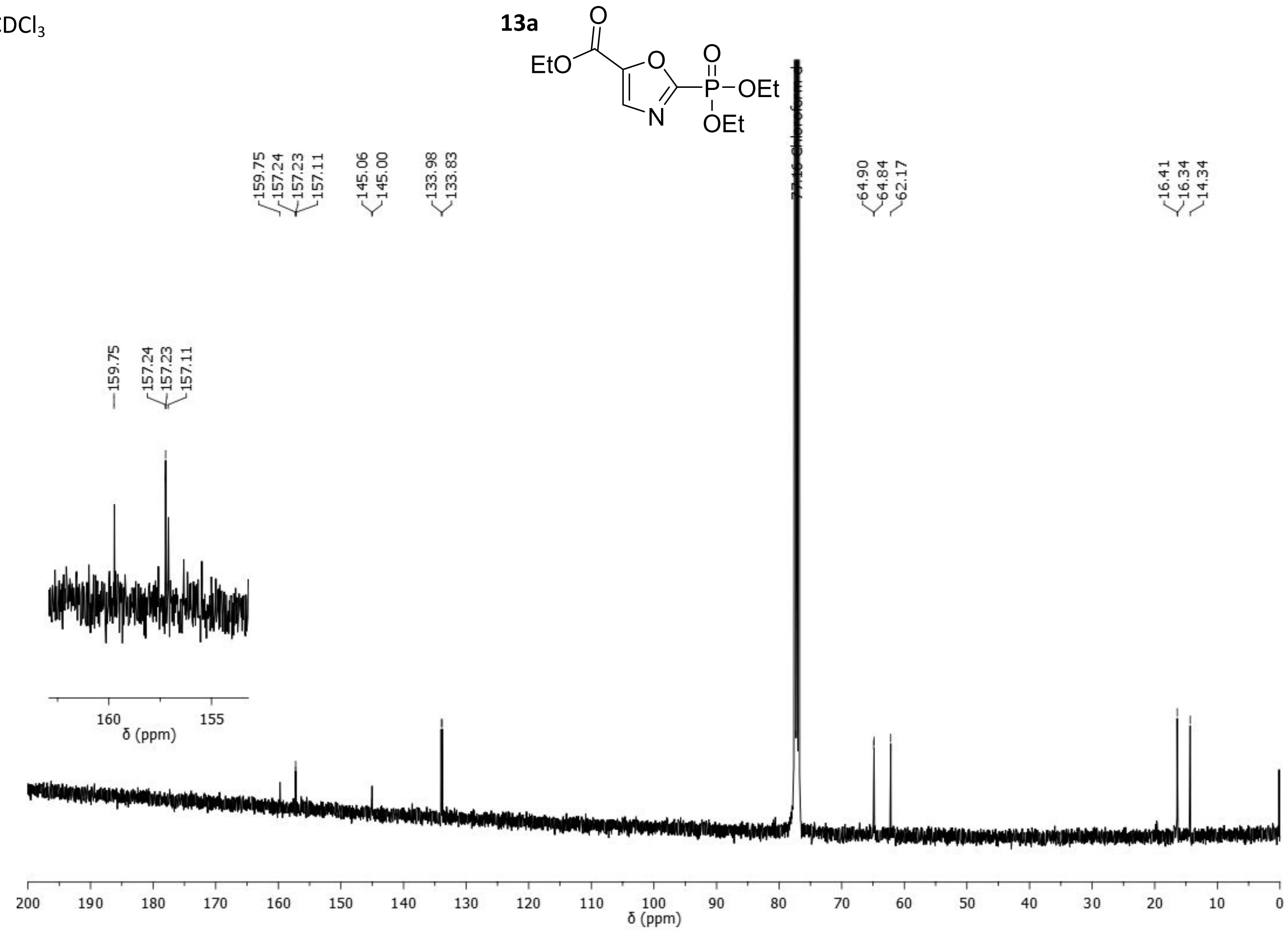

Figure S77. ^{13}C NMR spectrum of **13a.**

^{31}P 162MHz, $CDCl_3$

13a

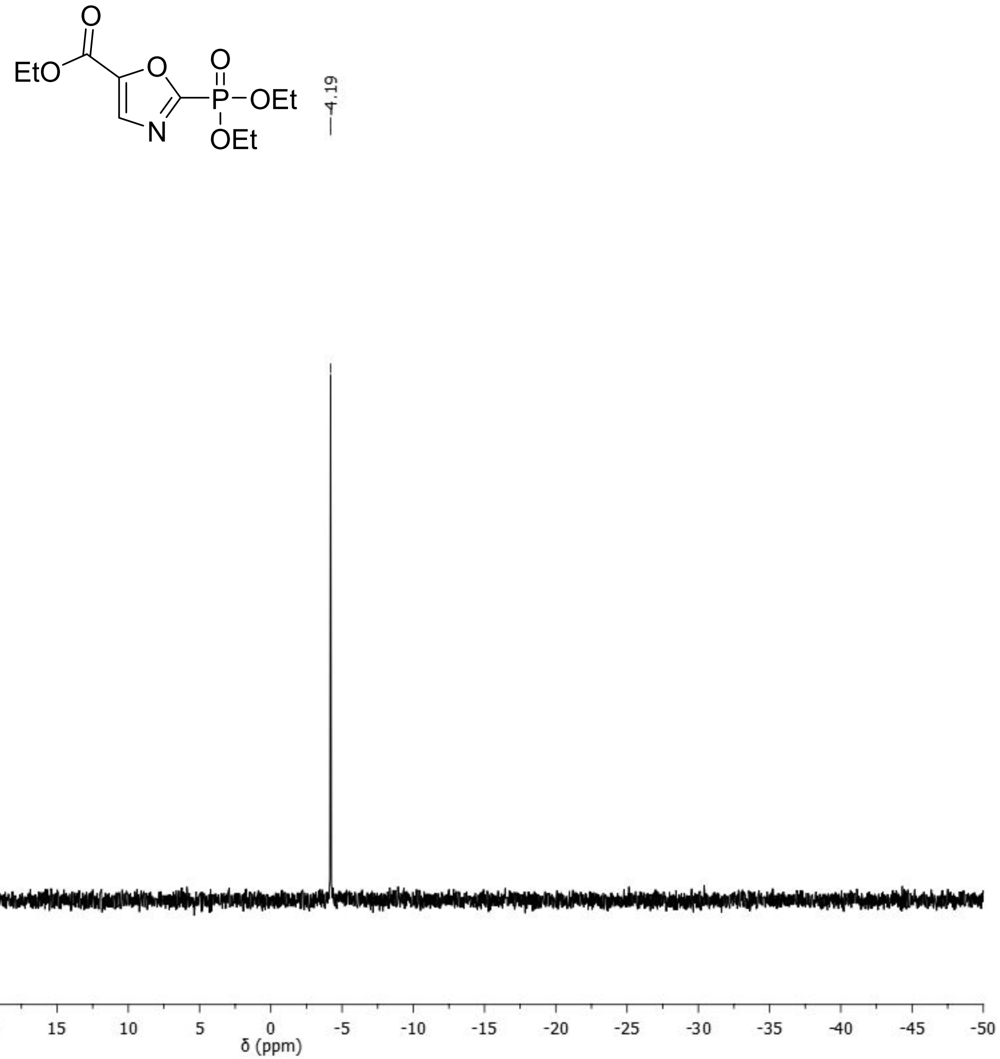

Figure S78. ^{31}P NMR spectrum of **13a.**

^{1}H 400MHz, $CDCl_3$

14a

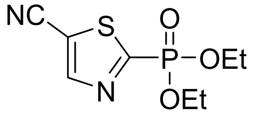

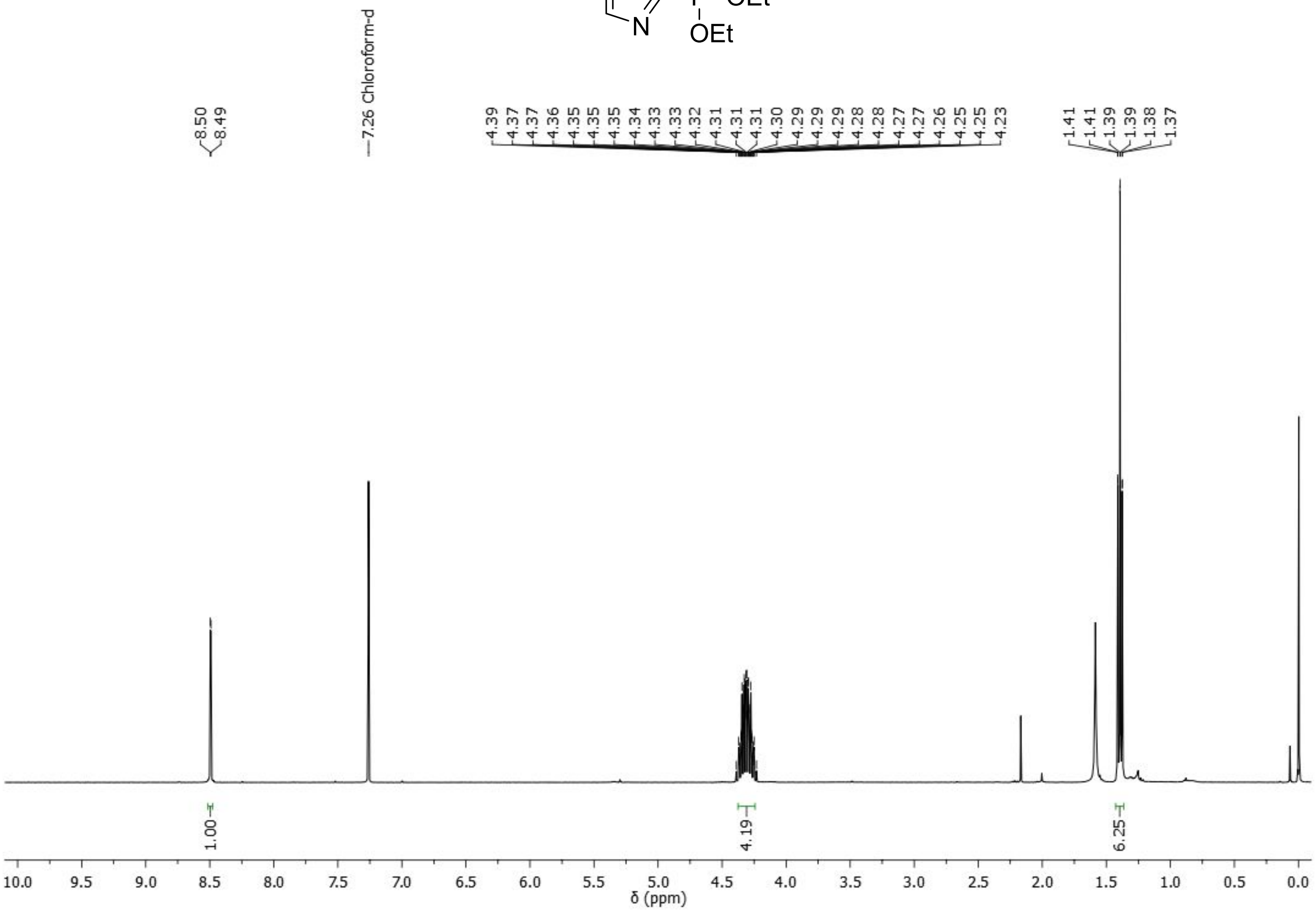

Figure S79. ^{1}H NMR spectrum of **14a.**

^{13}C 101MHz, $CDCl_3$

14a

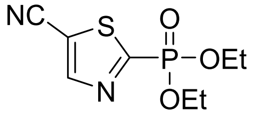

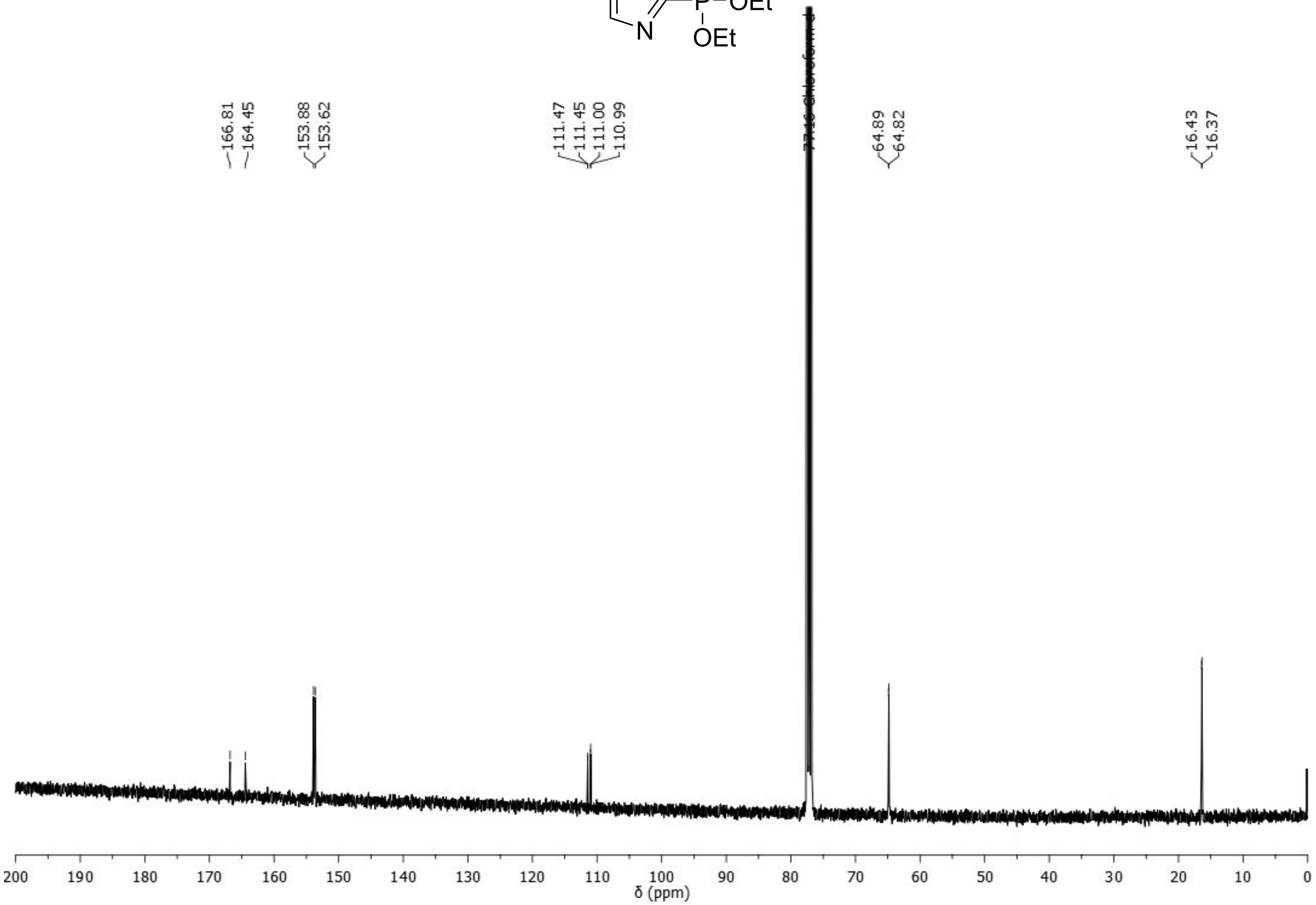

Figure S80. ^{13}C NMR spectrum of **14a.**

^{31}P 162MHz, $CDCl_3$

14a

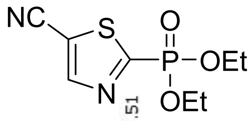

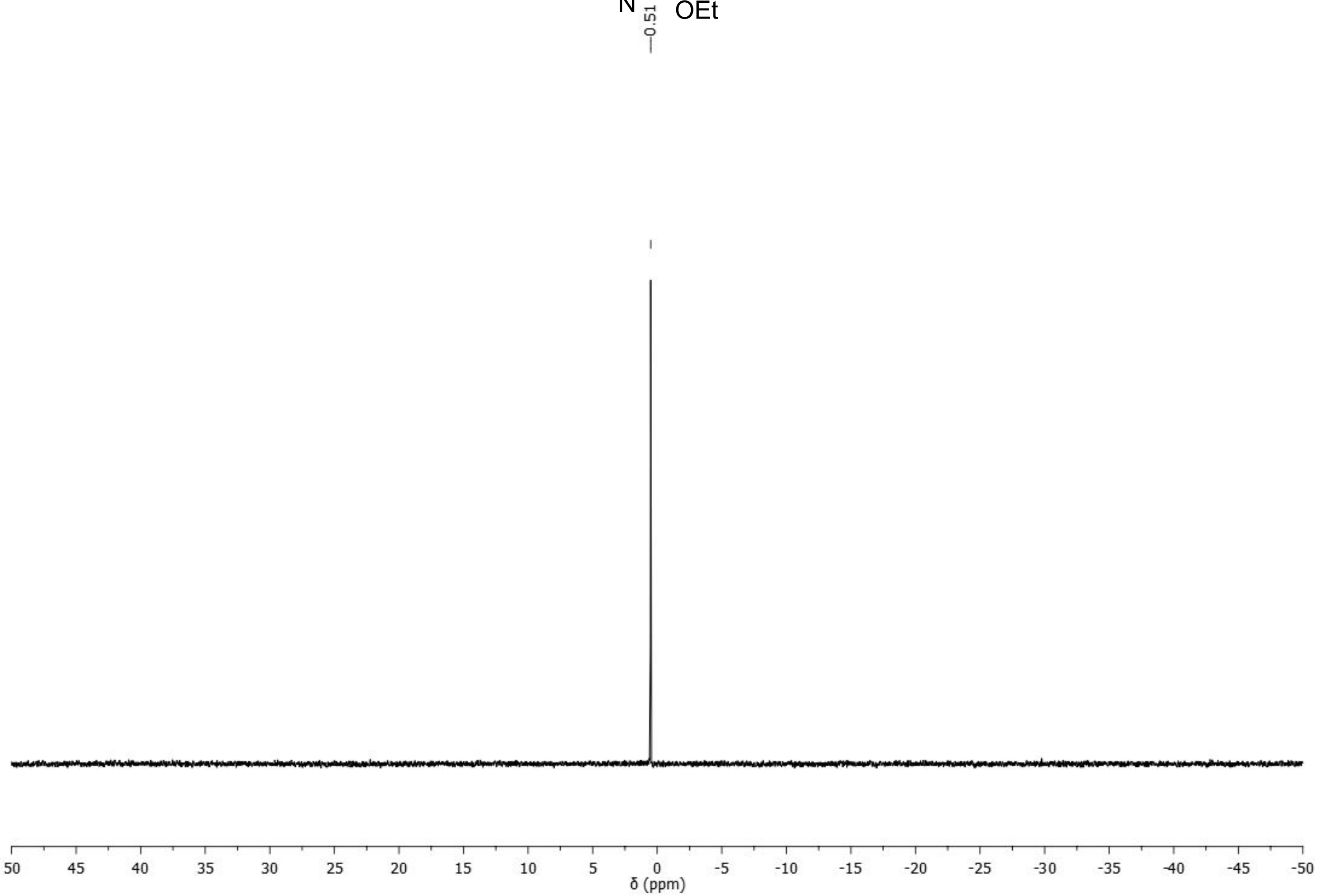

Figure S81. ^{31}P NMR spectrum of **14a.**

^{1}H 400MHz, $CDCl_3$

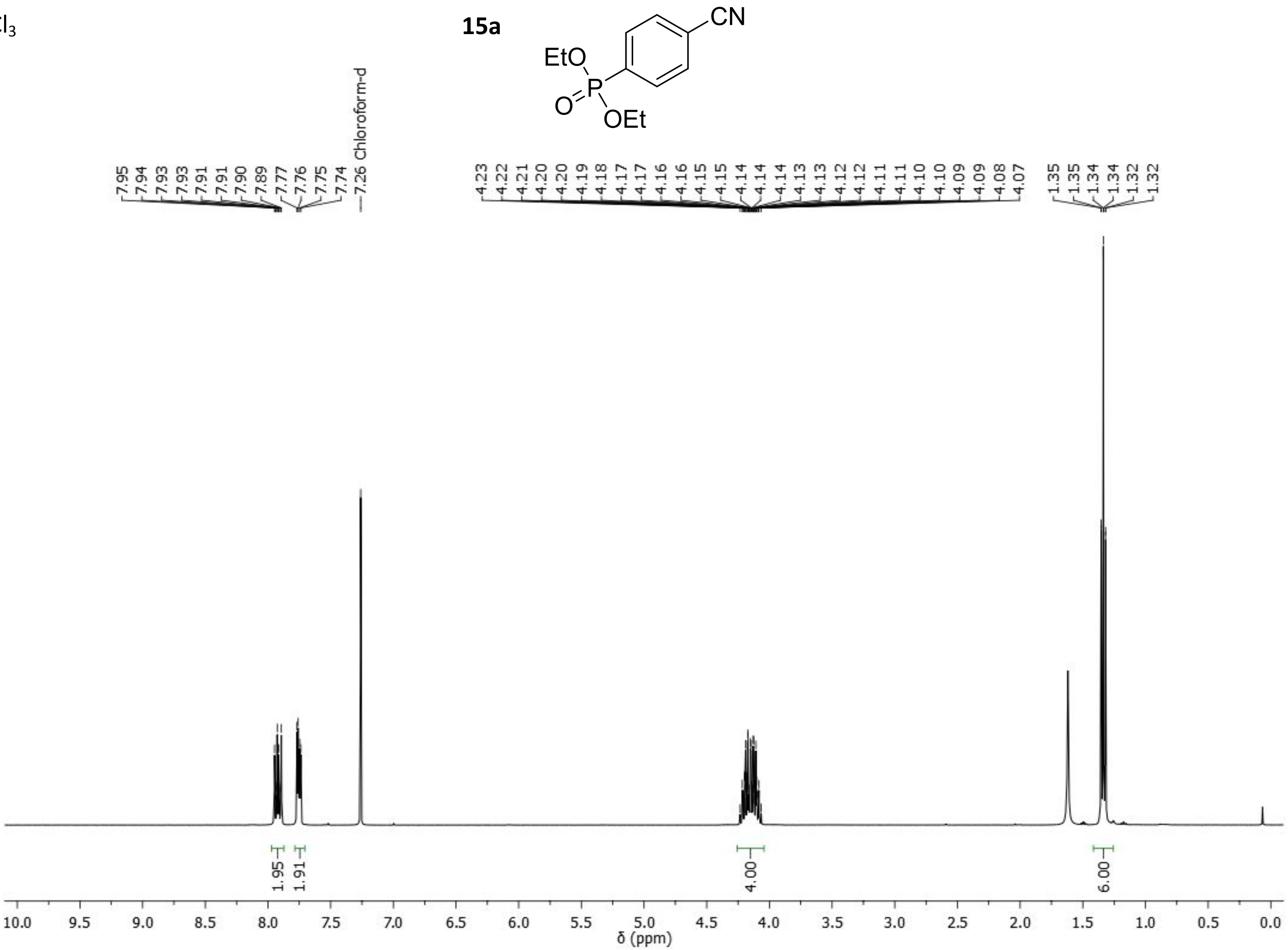

Figure S82. ^{1}H NMR spectrum of **15a.**

^{13}C 101MHz, $CDCl_3$

15a

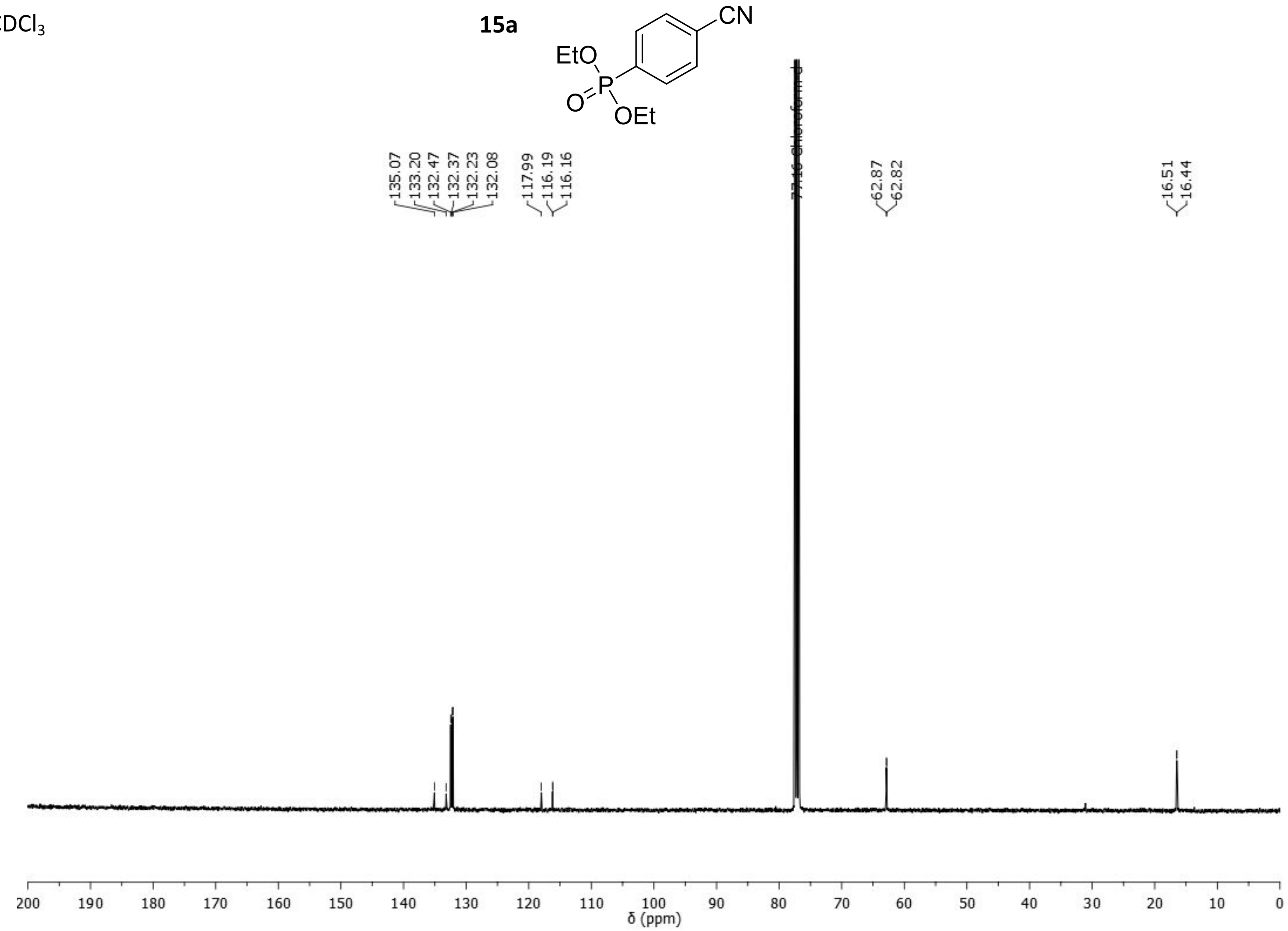

Figure S83. ^{13}C NMR spectrum of **15a.**

^1H 400MHz, $CDCl_3$

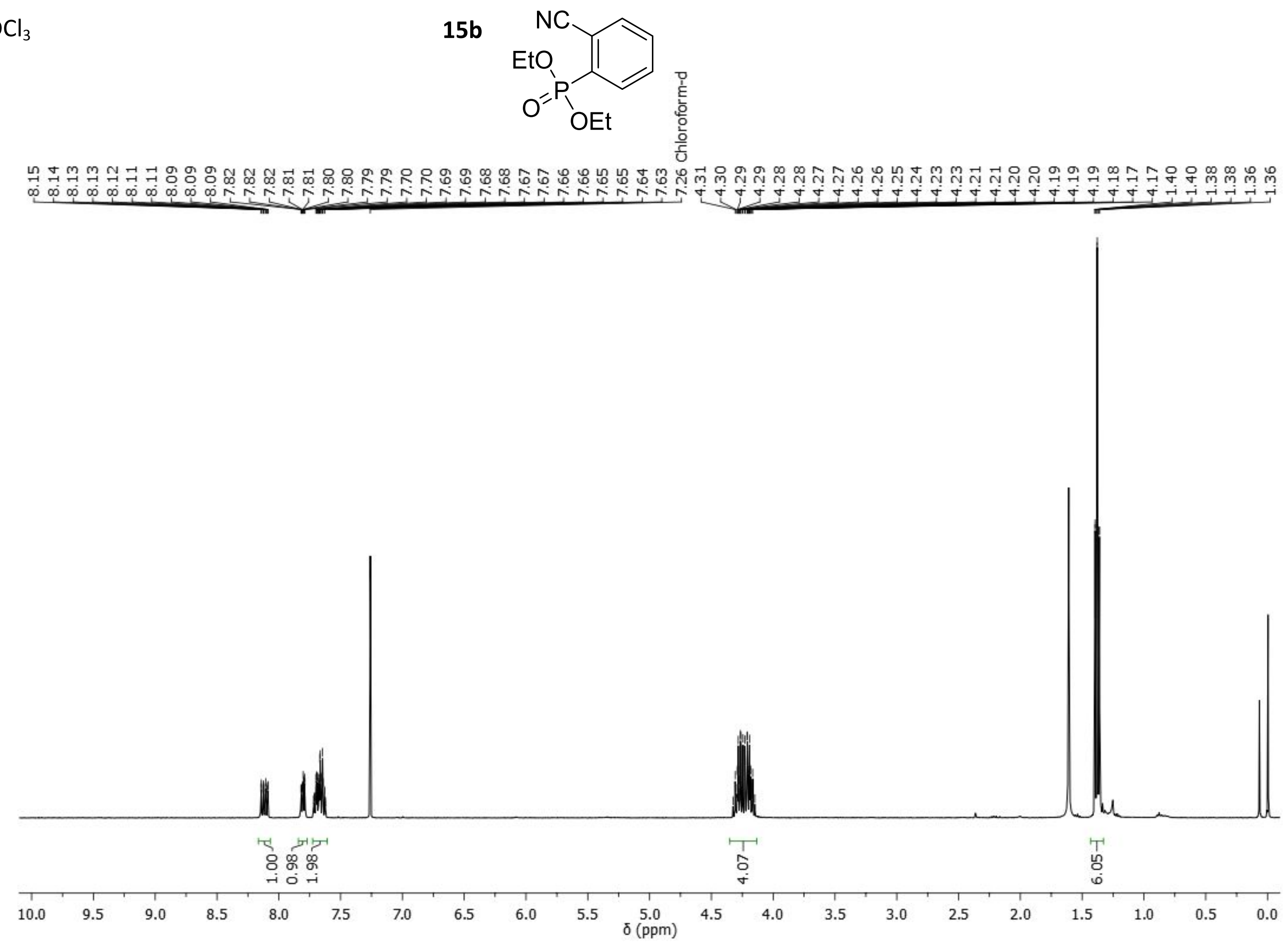

Figure S84. ^1H NMR spectrum of 15b.

^{13}C 101MHz, $CDCl_3$

15b

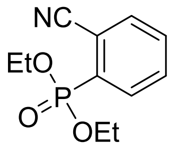

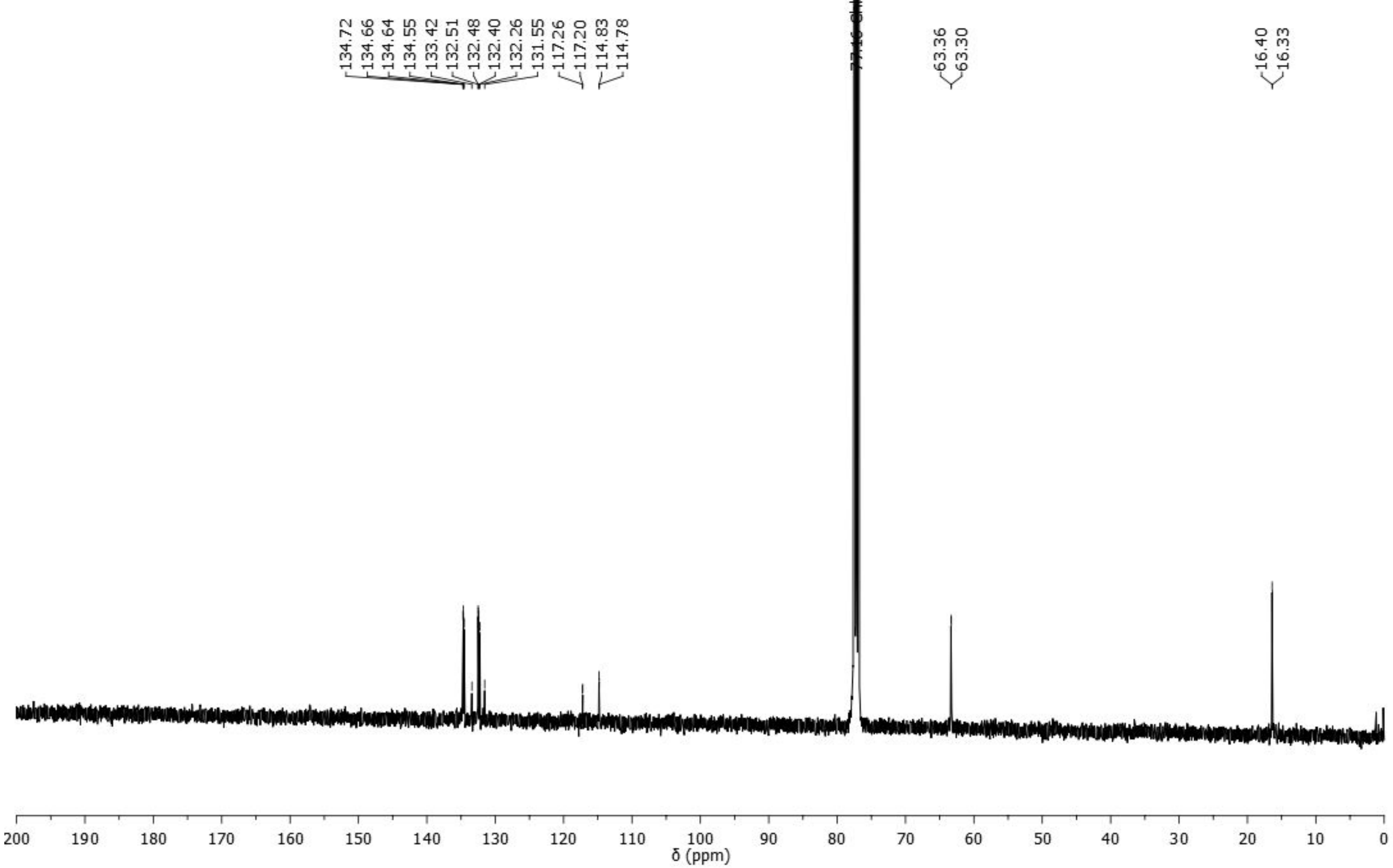

Figure S85. ^{13}C NMR spectrum of **15b.**

^1H 400MHz, $CDCl_3$

15c

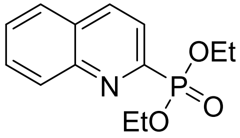

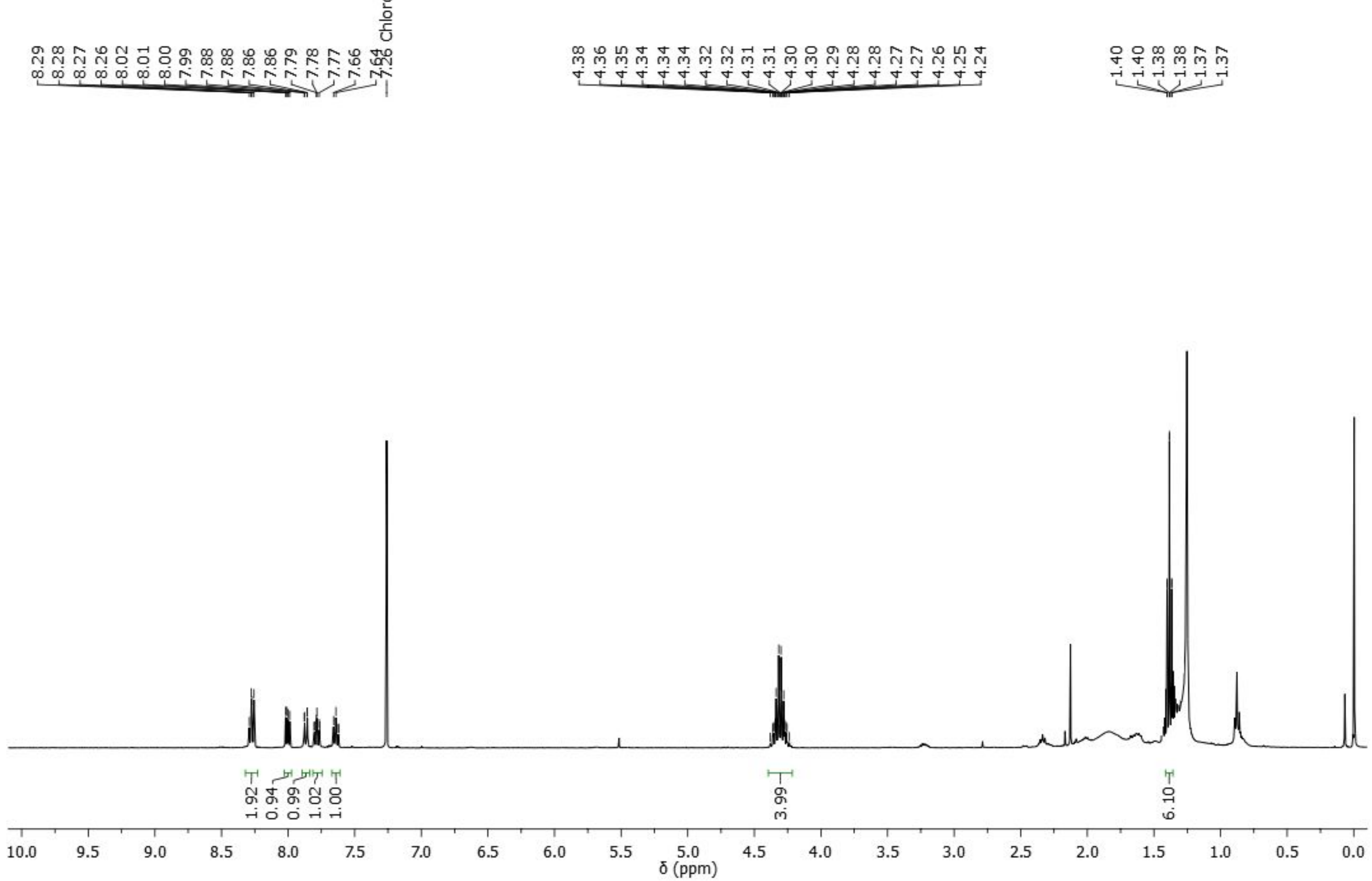

Figure S86. ^1H NMR spectrum of **15c.**

^1H 400MHz, $CDCl_3$

15d

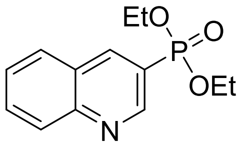

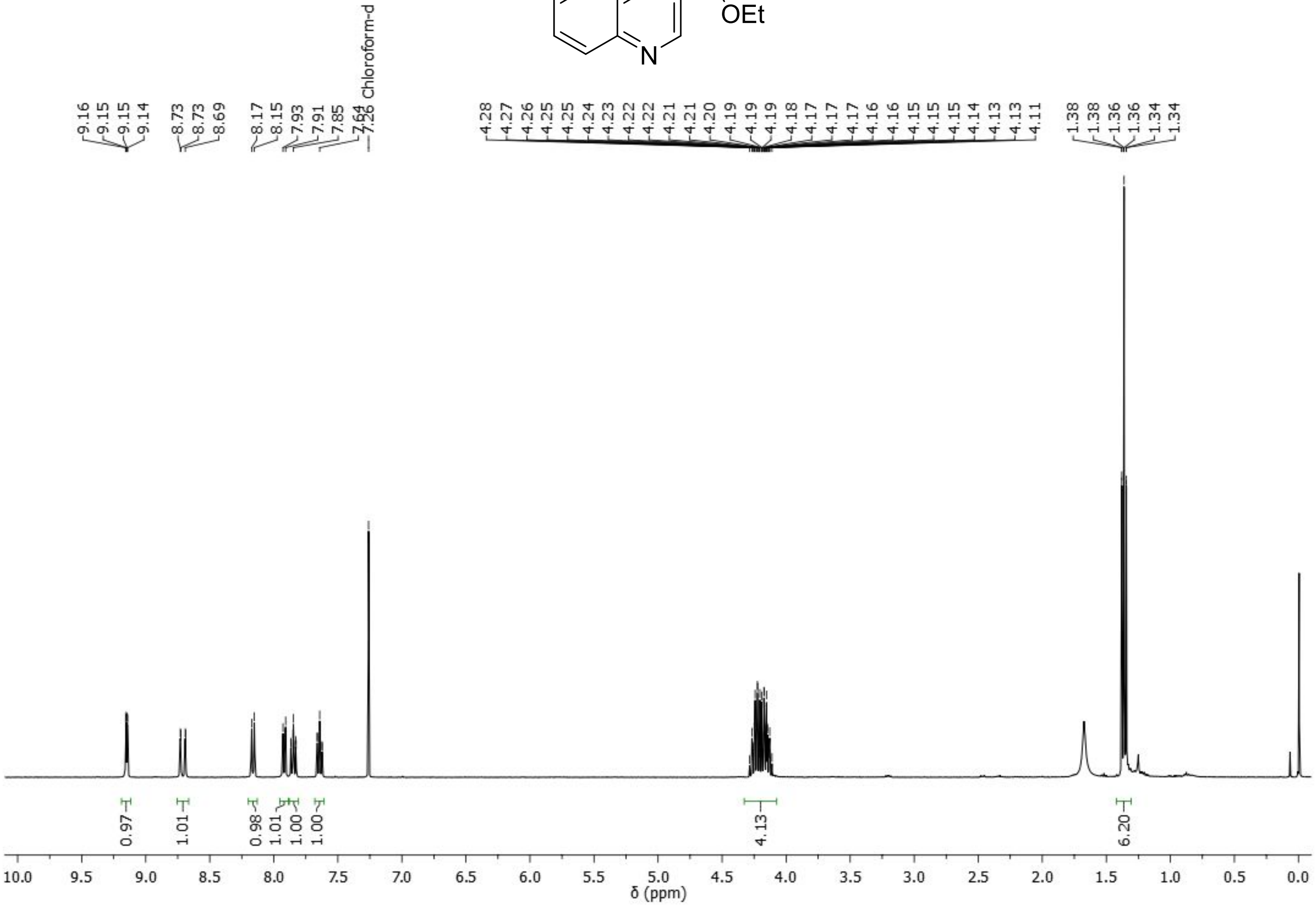

Figure S87. ^1H NMR spectrum of **15d.**

^{13}C 101MHz, $CDCl_3$

15d

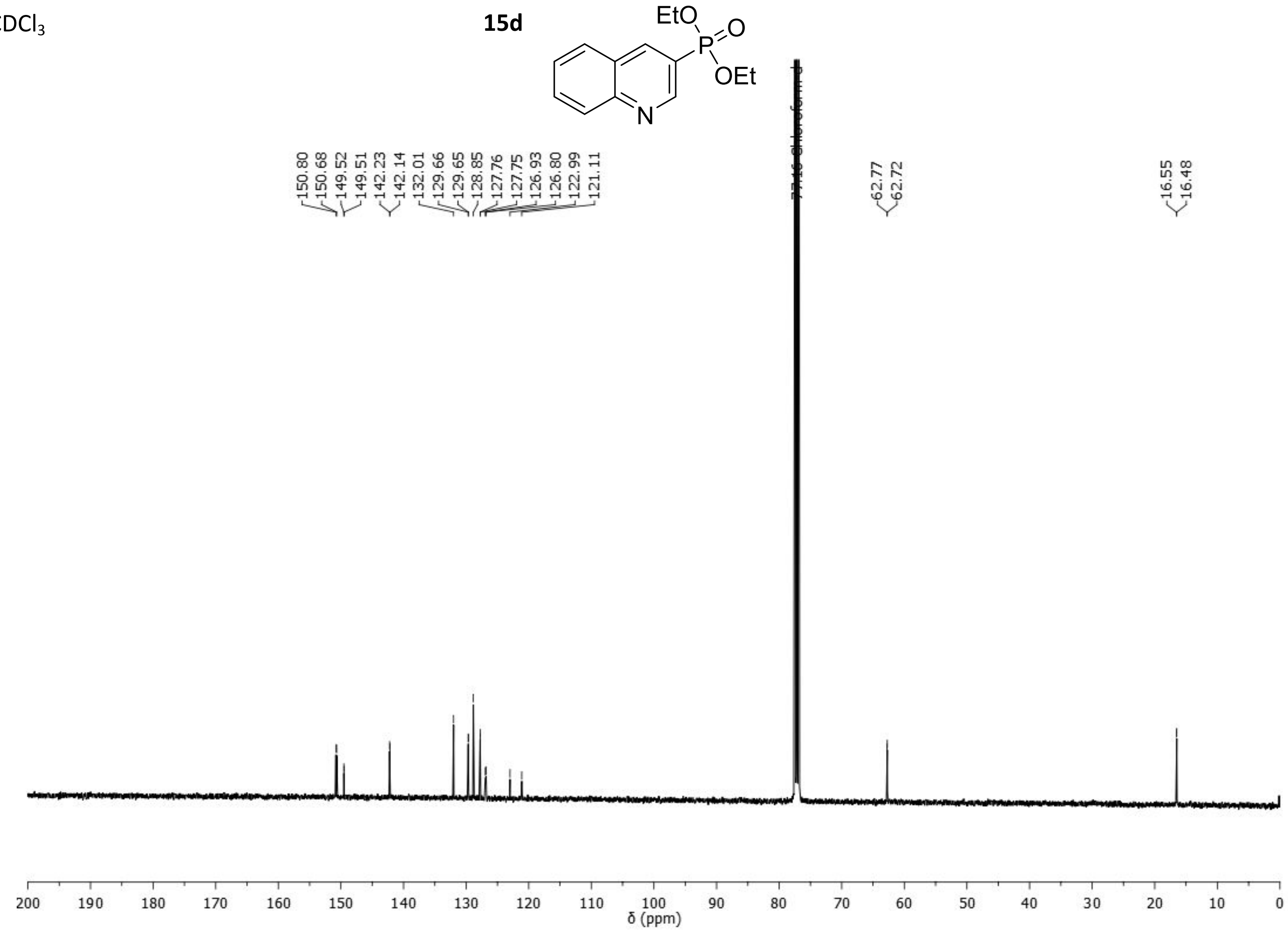

Figure S88. ^{13}C NMR spectrum of **15d.**

15e

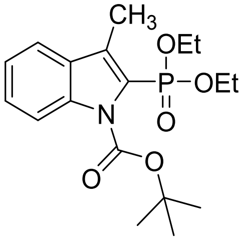

^{1}H 400MHz, $CDCl_3$

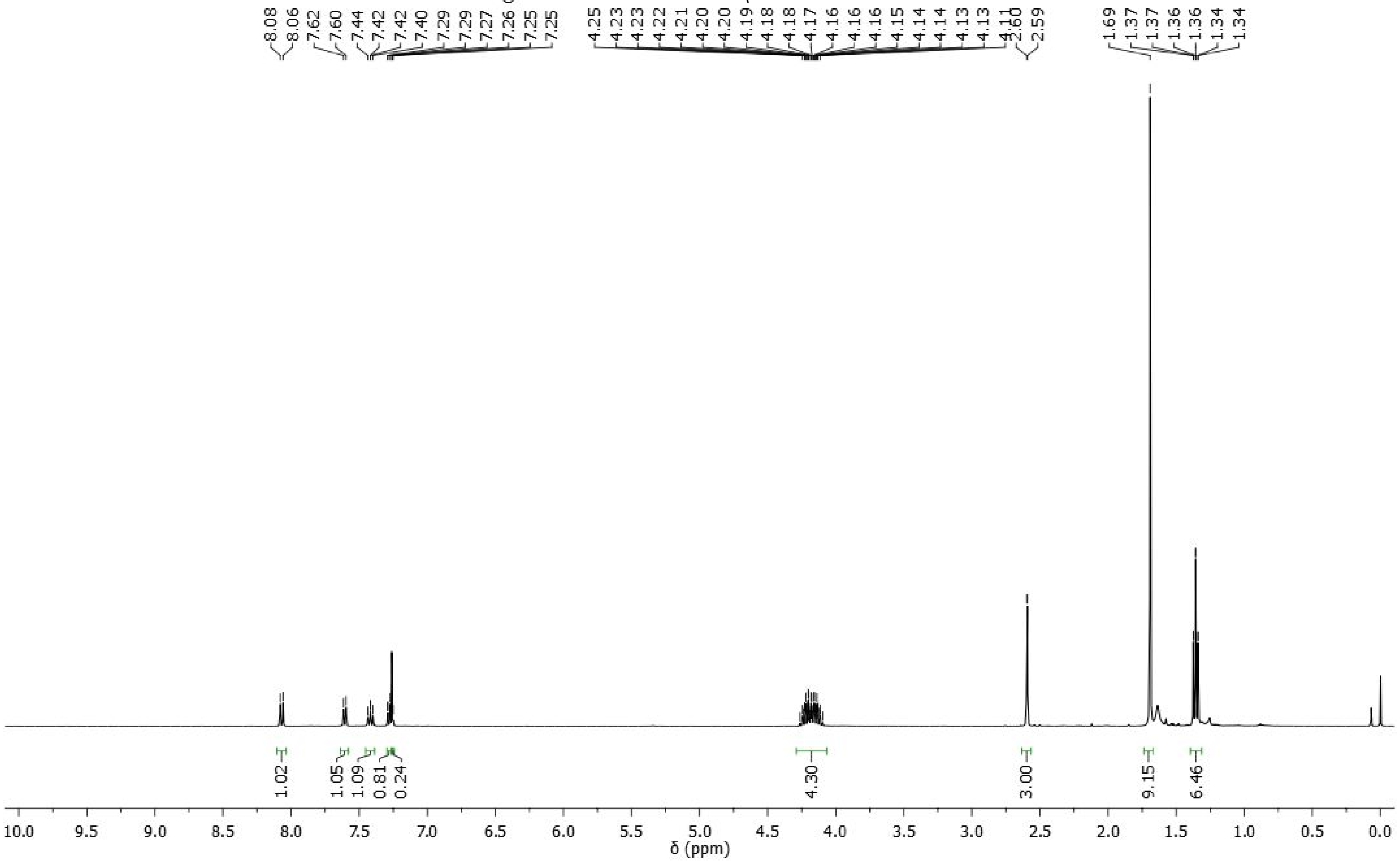

Figure S89. ^{1}H NMR spectrum of **15e.**

^{13}C 101MHz, $CDCl_3$

15e

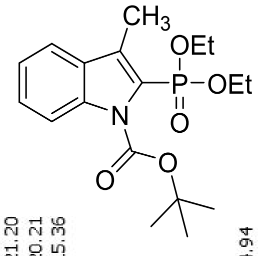

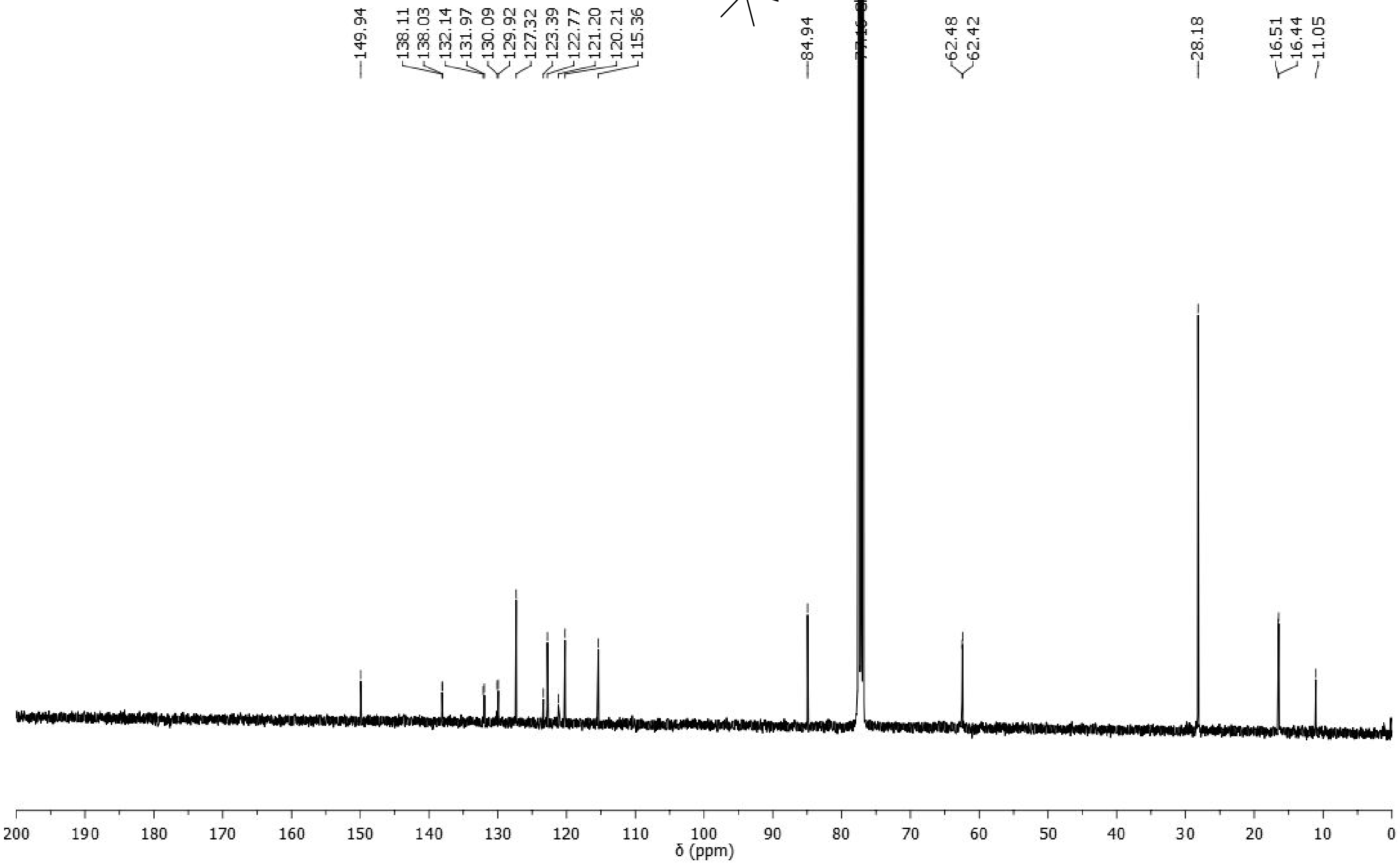

Figure S90. ^{13}C NMR spectrum of **15e**.

^{1}H 400MHz, $CDCl_3$

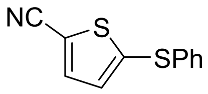

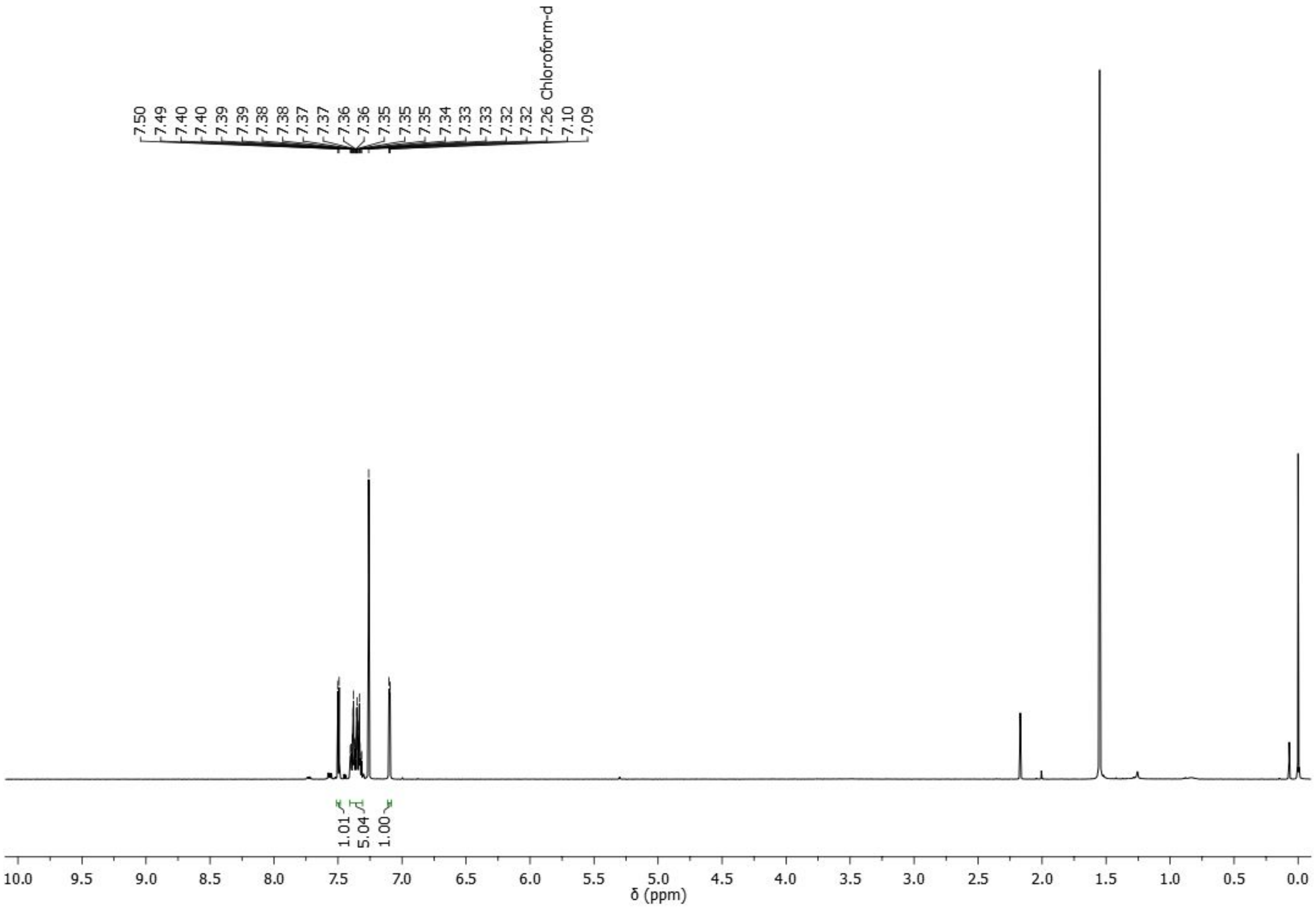

Figure S91. ^{1}H NMR spectrum of **thiophenecarbonitrile phenylsulfide.**

^{13}C 101MHz, $CDCl_3$

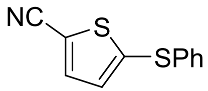

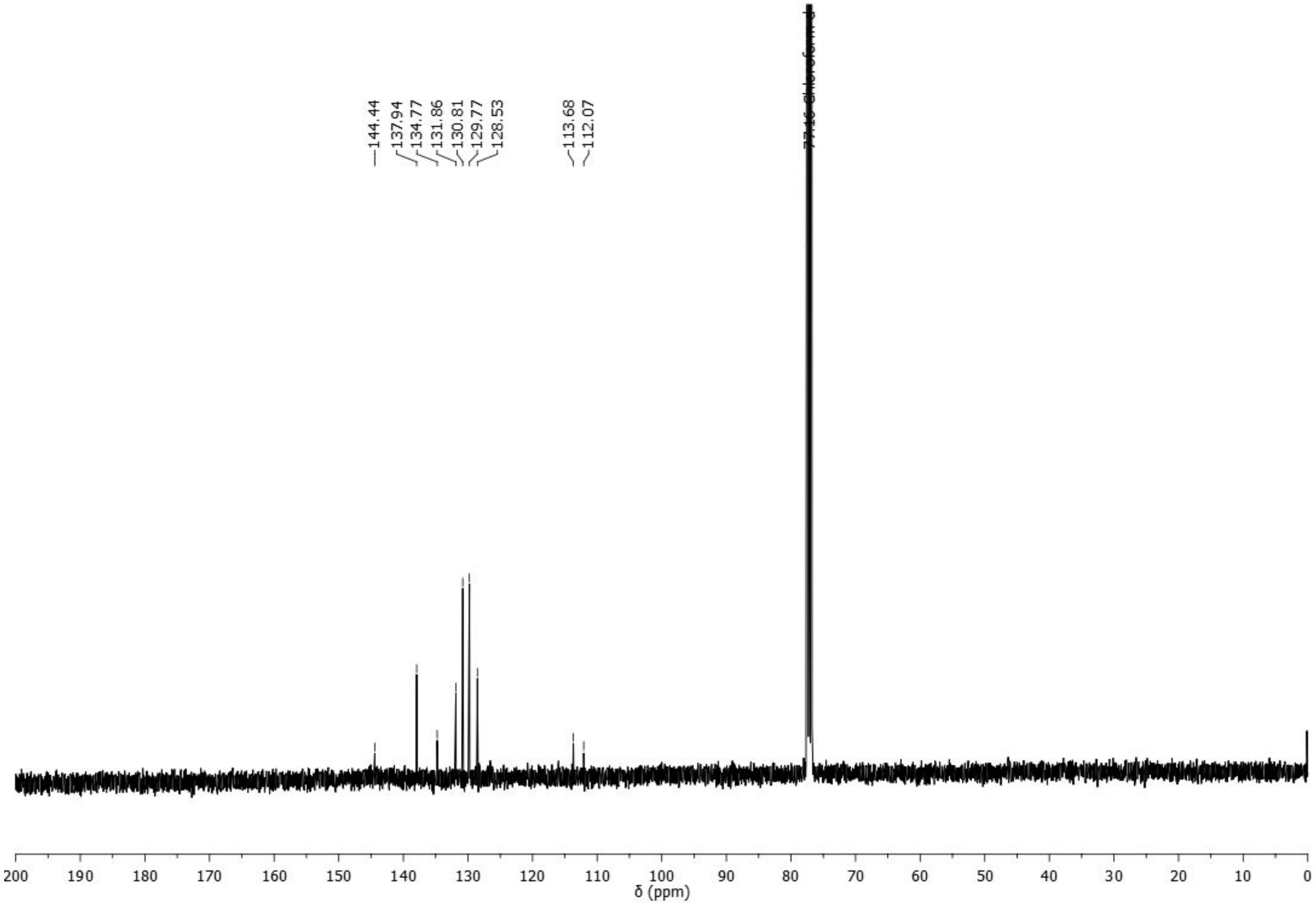

Figure S92. ^{13}C NMR spectrum of **thiophenecarbonitrile phenylsulfide.**
